# Catalytic Asymmetric Synthesis of Tröger’s Base Analogues with Nitrogen Stereocenter

**DOI:** 10.1021/acscentsci.2c01121

**Published:** 2023-01-04

**Authors:** Chun Ma, Yue Sun, Junfeng Yang, Hao Guo, Junliang Zhang

**Affiliations:** † Department of Chemistry, Fudan University, 2005 Songhu Road, Shanghai, 200438, P. R. China; ‡ Zhuhai Fudan Innovation Institute, Zhuhai, 519000, P. R. China; § Fudan Zhangjiang Institute, Shanghai 201203, P. R. China; ∥ School of Chemistry and Chemical Engineering, Henan Normal University, Xinxiang, Henan 453007, P. R. China

## Abstract

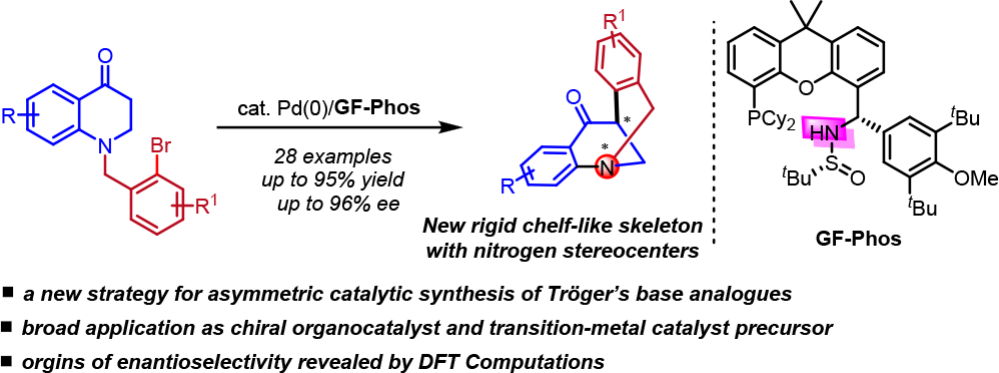

Nitrogen stereocenters are common chiral units in natural products, pharmaceuticals, and chiral catalysts. However, their research has lagged largely behind, compared with carbon stereocenters and other heteroatom stereocenters. Herein, we report an efficient method for the catalytic asymmetric synthesis of Tröger’s base analogues with nitrogen stereocenters via palladium catalysis and home-developed **GF-Phos**. It allows rapid construction of a new rigid cleft-like structure with both a C- and a N-stereogenic center in high efficiency and selectivity. A variety of applications as a chiral organocatalyst and metallic catalyst precursors were demonstrated. Furthermore, DFT calculations suggest that the NH···O hydrogen bonding and weak interaction between the substrate and ligand are crucial for the excellent enantioselectivity control.

## Abstract 2

Catalytic asymmetric synthesis of Tröger’s base analogues with nitrogen stereocenter via Pd catalysis and home-developed GF-Phos and origins of enantioselectivity revealed by DFT computations.

## Introduction

**Scheme 1:**
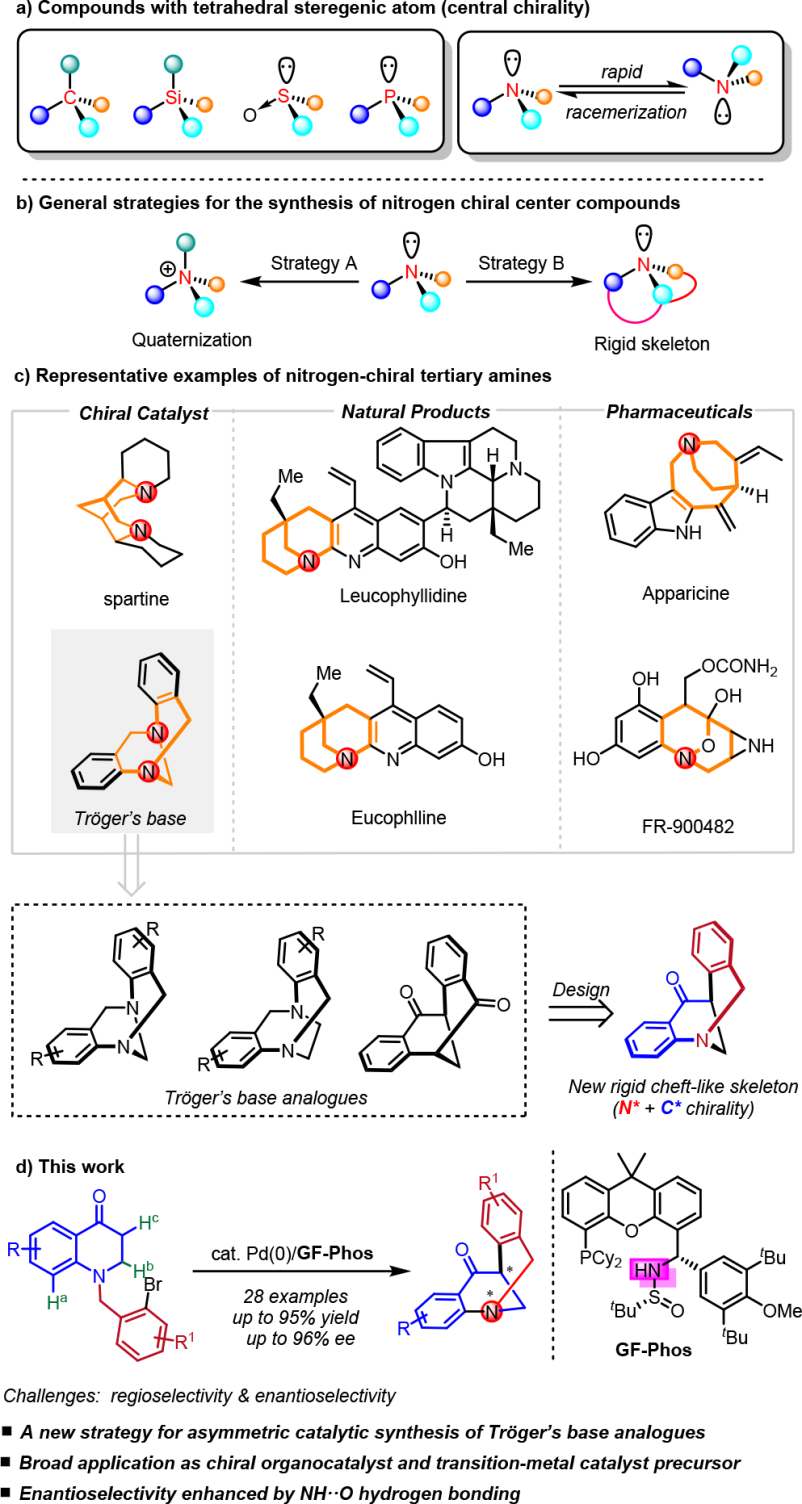
Profile of Compounds with Tetrahedral Stereogenic Atom and Representative Examples of Nitrogen-Chiral Tertiary Amines

Development of efficient and selective processes to access enantioenriched stereocenters are critical objectives in modern synthetic research. Various routes have been established to construct a carbon-stereogenic center, as well as a heteroatom-stereogenic center.^1−8^ In contrast, enantioselective synthesis of nitrogen stereocenters is often challenging because they tend to undergo rapid racemerization due to their low interconversion barrier (Scheme 1a).^9^ In this regard, strategies for enantioselective synthesis of chiral nitrogen-stereogenic center often invlove amine *N*-oxides, *N*-centered metal coordination and *N*-centered quaternary ammonium salts (Scheme 1b).^10−14^ Apart from these methods, another strategy^15^ is to form rigid tertiary amine skeleton which could prevent the inversion of nitrogen’s lone pair. These structures commonly exist in many alkaloids,^16−19^ pharmaceuticals^20,21^ and chiral Lewis base catalysts (Scheme 1c).^22−24^

Among these rigid chiral tertiary amine compounds, one particularly fascinating structure is Tröger’s base. It has two aromatic rings perpendicular to each other which fused to the central bicyclic [3.3.1] framework, enable to form a rigid cleft-like V-shaped scaffold possessing two nitrogen stereocenters.^25,26^ Hence, Tröger’s base has attracted considerable attention due to its application in self-assembly studies, molecular recognition, DNA-interacting probes, as well as Lewis base catalyst in organic synthesis.^22−24,27−32^ However, enantiopure Tröger’s base has rarely been used excessively, as it is configurationally instable under acidic conditions,^33−35^ which limits its wide application. To this end, various Tröger’s base analogues with rigid cleft-like skeleton have been developed by modifying the cleft-like scaffold to increase its stability,^27,28,36−41^ most of which involve multiple-step synthesis or late-stage resolution.

Since the pioneering work by Miura,^42^ Buchwald,^43^ Hartwig,^44^ and others, α-arylation of carbonyls has become one of powerful and useful method in organic synthesis.^42−52^ Recently, Jia, Shi, Zhou, Liu and Gong did seminal contribution in the asymmtric intramolecular α-arylation of ketones, leading various types of bridged bicyclic skeletons.^53−58^ Very recently, our group also disclosed the asymmetric intermolecular α-arylation of acyclic aldehydes and γ-arylation of β,γ-unsaturated butenolides.^59,60^ Along this research line, we wonder whether the rigid cheft-like scaffold could be accessed by the arylation of carbonyl compounds using *N*-benzyl substituted dihydroquinolinone derivatives as starting materials. This approach demonstrates the following advantages: (1) in comparison to Tröger’s base, this designed scaffold is configurationally stable under either acidic or basic conditions; (2) the nitrogen stereocenter, as well as the neighboring carbon stereocenter, is established simultaneously. No late-stage resolution is involved. Nevertheless, several challenges need to be addressed in this scenario, such as the associated debromination, sp^2^ C–H arylation, and sp^3^ C–H arylation, as well as the enantioselectivity control. Herein, we report our progress on the catalytic asymmetric synthesis of a stable Tröger’s base analogue using Pd-catalysis and home-developed **GF-Phos** (Scheme 1d).

## Results and Discussion

At the outset, the *N*-benzyl substituted dihydroquinolinone derivative **1a** designed was selected as the model substrate to verify our hypothesis. As listed in Table 1, a series of commercially available chiral phosphine ligands, such as bidentate P,P-ligands (*R*)-SDP (**L1**), (*R*,*R*)-QuinoxP (**L2**), (*R*)-Segphos (**L3**, **L4**), (*R*)-DIOP (**L5**), (*S*)-Phanephos (**L6**), (*R*, *S*)-Josiphos (**L7**), (*R*,*S*)-PFA (**L8**), and N, P-ligand (*S*,*S*)-PHOX (**L9**), as well as monodentate ligands (*R*)-Antphos (**L10**) and (*R*)-MOP (**L11**, **L12**) were investigated. Unfortunately, most of the commercial ligands (**L1**, **L3**–**L8**, **L10**, **L11**) failed to promote this reaction. Although (*R*,*R*)-QuinoxP (**L2**) and (*S*,*S*)-PHOX (**L9**) could achieve moderate to good enantioselectivity, the reactivity was poor. (*R*)-MOP (**L12**) promoted the reaction well, albeit with miserable enantioselectivity control. These results reveal that the choice of ligand is crucial for the reactivity and selectivity. Recently, Sadphos^61^ developed by our group have shown excellent performance in metal-catalyzed coupling reactions. We next turned to investigate the performance of Sadphos in this reaction. Diphenylphosphine derived Ming-Phos^62^ (**Ming1**–**Ming4**), Xiao-Phos (**Xiao1**, **Xiao2**) and Wei-Phos (**Wei1**, **Wei2**) PC-Phos^63^ (**PC1, PC2**) achieved excellent results in enantioselectivity control, even though the yield was low (Table 1, entry 1 and entry 2). Remarkably, if the nitrogen of **PC2** was masked by the methyl group (**PC3**), the enantioselectivity dropped sharply (Table 1, entry 3). Such results demonstrate that the NH group of the ligand (**PC2**) has a great influence on the enantioselectivity of the reaction. With this result in mind, different R^2^ groups (**PC4**–**PC10**) were subsequently investigated, and the desired product was best furnished in 31% yield and 96% ee (Table 1, entry 10). In most cases, we detected the byproduct **3a**, which might be generated by the β-hydride elimination before the reductive elimination^64^ or the HAT after the oxidative addition.^65^ Considering the importance of bases in metal-catalyzed coupling reactions, we investigated various bases. When CsOAc was used, significant amount of **4a** was detected (Table 1, entry 13), which is produced through the neighboring sp^2^ C–H arylation. However, the whole base screening is unfruitful. At this time, we were reminded that, during the screening of (*R*)-MOP ligands, replacing diphenylphosphine with the more electron-rich dicyclohexylphosphine could greatly increase the yield. In view of this result, we tried the more electron-rich **GF-Phos**.^66^ Gratifyingly, the better yield was obtained (Table 1, entry 15). The yield could be improved further by fine-tuning other parametors, such as solvents, reaction temperature and catalyst loading (see pp S7 and S8 of the Supporting Information). Finally, product **2a** was delivered in good yield (80%) with high enantioselectivity (96% ee) under the optimal reaction conditions.

**Table 1:**
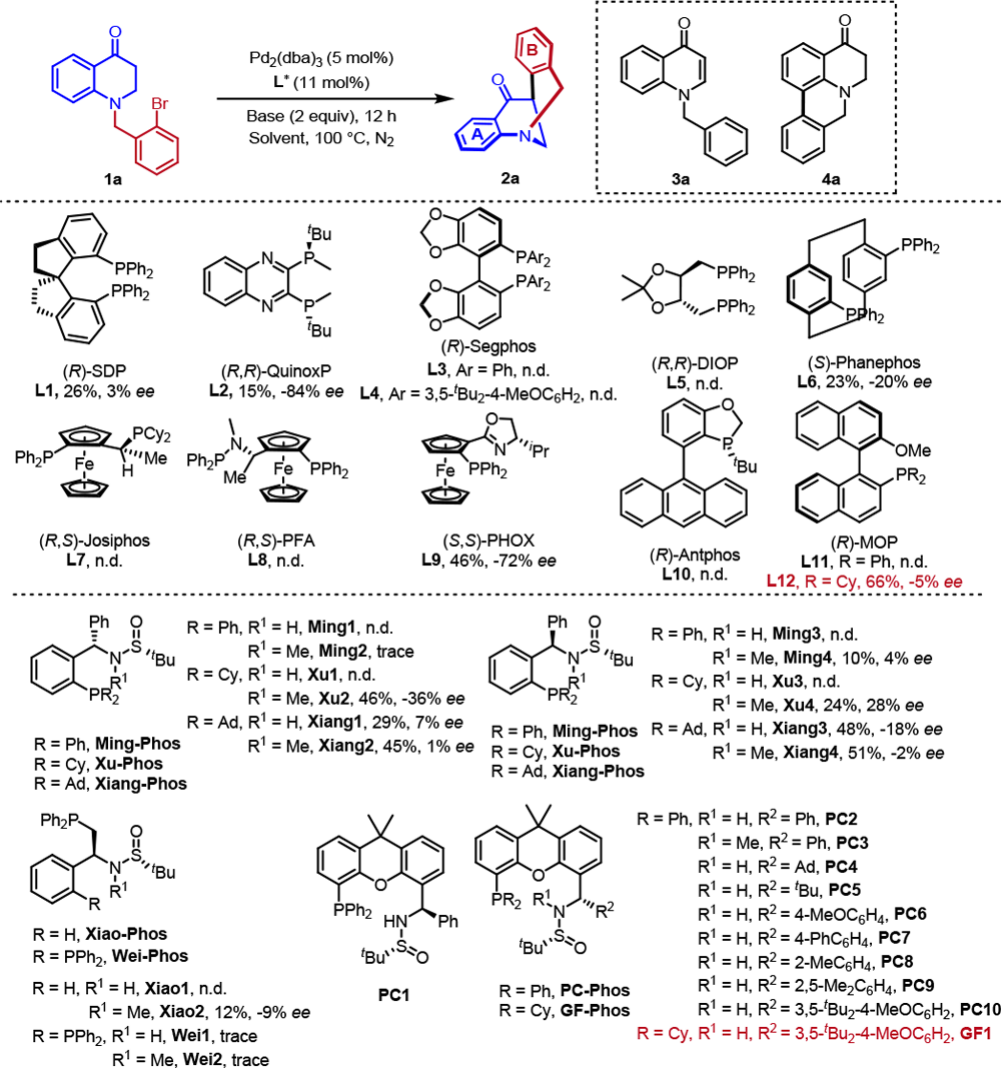
Optimization of Reaction Conditions^a^

entry	L*	base	solvent	yield of **2a** [%]^b^	yield of **3a** (%)^b^	ee (%)^c^
1	**PC1**	Cs_2_CO_3_	toluene	4	4	75
2	**PC2**	Cs_2_CO_3_	toluene	14	18	95
3	**PC3**	Cs_2_CO_3_	toluene	14	n.d.	6
4	**PC4**	Cs_2_CO_3_	toluene	23	n.d.	1
5	**PC5**	Cs_2_CO_3_	toluene	17	n.d.	4
6	**PC6**	Cs_2_CO_3_	toluene	21	n.d.	93
7	**PC7**	Cs_2_CO_3_	toluene	14	10	6
8	**PC8**	Cs_2_CO_3_	toluene	25	3	80
9	**PC9**	Cs_2_CO_3_	toluene	27	3	79
10	**PC10**	Cs_2_CO_3_	toluene	31	17	96
11	**PC10**	K_2_CO_3_	toluene	36	n.d.	88
12	**PC10**	K_3_PO_4_	toluene	34	n.d.	88
13	**PC10**	CsOAc	toluene	n.d.	n.d.	–
14	**PC10**	KHCO_3_	toluene	21	6	74
15	**GF1**	Cs_2_CO_3_	toluene	55	n.d.	97
16^d^	**GF1**	Cs_2_CO_3_	CH_3_CN	80(77)	n.d.	96
17	**GF1**	Cs_2_CO_3_	DMF	58	7	81
18	**GF1**	Cs_2_CO_3_	DMSO	42	8	36

a Reaction conditions: **1a** (0.1 mmol), Pd_2_(dba)_3_ (5 mol %), L* (11 mol %), base (0.2 mmol), solvent (1.0 mL), 100 °C, 12 h.

b Yield was determined by GC using tetradecane as an internal standard.

c Determined by HPLC using a chiral stationary phase.

d The yield of isolated product is shown within the parentheses. DMSO = Dimethyl sulfoxide. DMF = *N,N*-Dimethylformamide. n.d. = not detected.

**Scheme 2:**
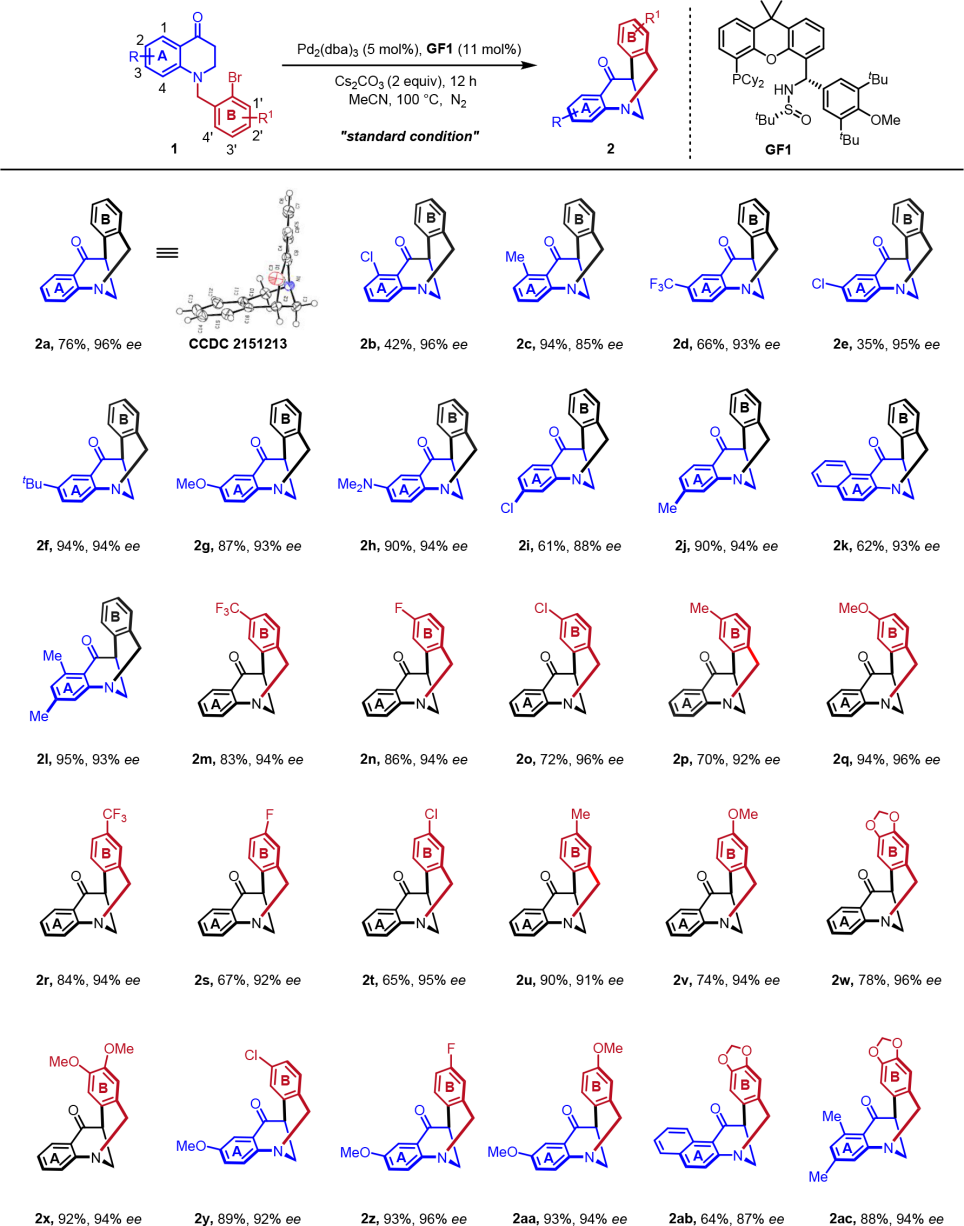
Variation of *N*-Benzyl Substituted Dihydroquinolinone Derivatives **1** Reaction conditions: **1** (0.3 mmol), Cs_2_CO_3_ (2.0 equiv), Pd_2_(dba)_3_ (5 mol %) and **GF1** (11 mol %) in CH_3_CN (3 mL) at 100 °C for 12 h.

With the optimal reaction conditions in hand, the substrate scope of *N*-benzyl substituted dihydroquinolinone derivatives **1** was then examined. As summarized in Scheme 2, a wide range of *N*-benzyl substituted dihydroquinolinone derivatives **1** bearing various R/R^1^ groups could efficiently undergo the intramolecular arylation, generating structurally diverse eight-membered *N*-bridged [3.3.1] ring product **2** containing nitrogen chirality in generally moderate to good yields with high enantioselectivities (35% to 95%, 85% to 96% ee). Specifically, we investigated a series of substituents on the aromatic ring of **A** and **B**. Both electron-withdrawing and electron-donating substituents could be well compatible. Good tolerance of functional groups including fluoro, chloro, methyl, naphthyl, methoxy, trifluoromethyl and amino (**2h**) groups was also observed. The reaction was found to be sensitive to steric influence on the benzene ring. Thus, the presence of a substituent at the 4,1′- or 4′-position in the substrates shuts down the desired reaction. When we used aryl iodide **1ad** and aryl chloride **1ae** instead of aryl bromides, no better results were obtained. (See Supporting Information for more details.) The absolute configuration of **2a** was confirmed by X-ray crystallography analysis, and those of the others were assigned analogously.

**Scheme 3:**
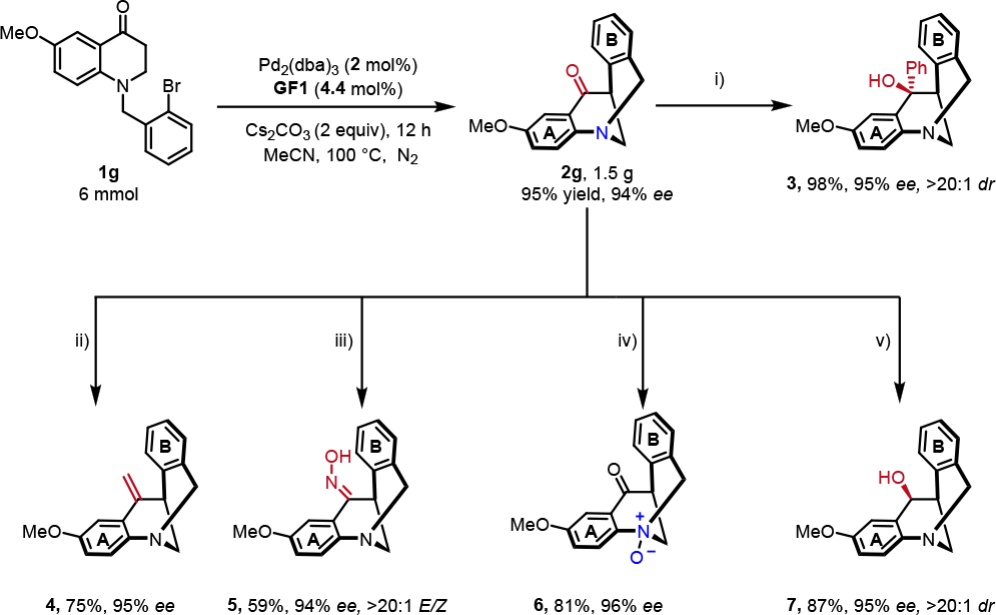
Gram-Scale Reaction and Derivatization of *N*-bridged [3.3.1] Ring Product **2g** Reaction conditions: (i) PhMgBr (1.5 equiv), THF, 0 °C to rt; (ii) PPh_3_MeBr (2.5 equiv), ^*t*^BuOK (2.5 equiv), THF, 0 °C to rt; (iii) HONH_2_·HCl (3.0 equiv), Py (3.0 equiv), Toluene/EtOH, 80 °C; (iv) *m*CPBA (2.2 equiv), DCM, 0 °C to rt; (v) NaBH_4_ (1.2 equiv), MeOH, 0 °C.

The utility of this method to access high-value, chiral building blocks were then demonstrated (Scheme 3). A gram-scale reaction of **1g** with lower catalyst loading (2 mol % of Pd_2_(dba)_3_ and 4.4 mol % of **GF1**) provided the eight-membered *N*-bridged [3.3.1] ring product **2g** (1.5 g) in 95% yield with 94% ee. Chiral *N*-bridged [3.3.1] ring tertiary alcohol **3**(67) and secondary alcohol **7**(68,69) in high yield with chirality retention were synthesized from **2g** by the 1,2-addition of PhMgBr and reduction with NaBH_4_, respectively. Then, the treatment of **2g** with hydroxylamine hydrochloride in heated EtOH/PhMe gave chiral *N*-bridged [3.3.1] ring oxime **5**(70) with perfect *E* selectivity in 59% yield with 94% ee. Next, Wittig reaction of **2g** with Ph_3_PMeBr could be achieved to access chiral *N*-bridged [3.3.1] ring olefin **4**(27) in 75% yield with 95% ee. Additionally, the chiral *N*-bridged [3.3.1] ring *N*-oxide product **6**(71) was successfully delivered by *m*CPBA oxidation in 81% yield and 96% ee.

**Scheme 4:**
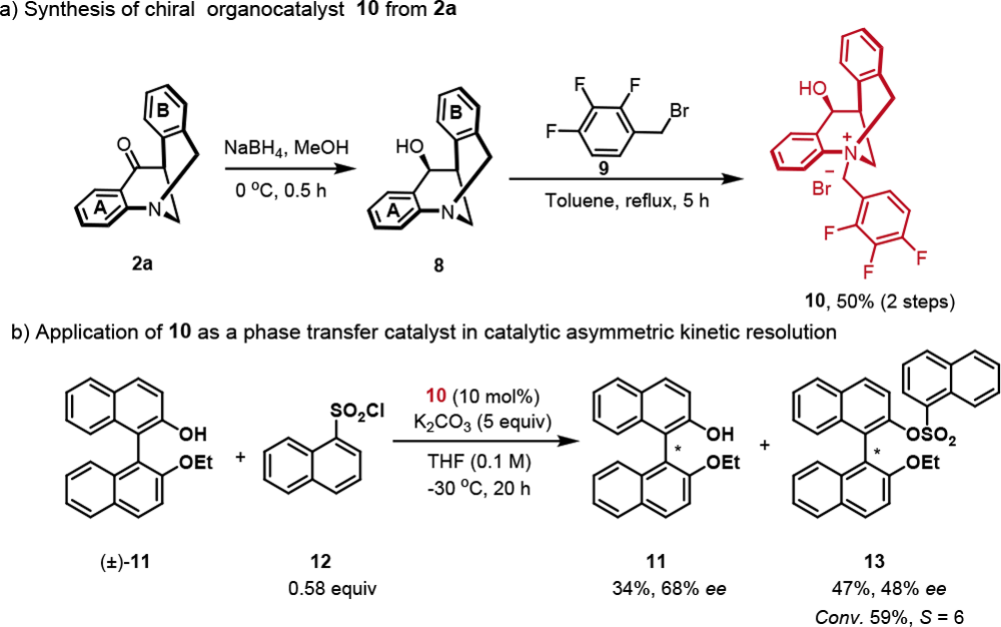
Synthesis of Chiral Organocatalyst **10** and Its Application in Catalytic Kinetic Resolution

As illustrated in Scheme 4, (*S, S*)-**2a** could be utilized for the synthesis of chiral quaternary ammonium salt **10** as a new chiral phase transfer catalyst, via a two-step reaction with a total yield of 50%. Gratifyingly, the application of **10** in catalytic asymmetric kinetic resolution^72^ of ethoxy-protected binaphthol **11** with 1-naphthalenesulfonyl chloride **12** successfully afforded chiral product **13** in 47% yield with 48% ee, and **11** was recovered with moderate enantioselectivity (68% ee, 59% *conv.*, *S* = 6). The above results will enlighten the future application of this new class of chiral phase transfer catalyst in asymmetric catalysis.

**Scheme 5:**
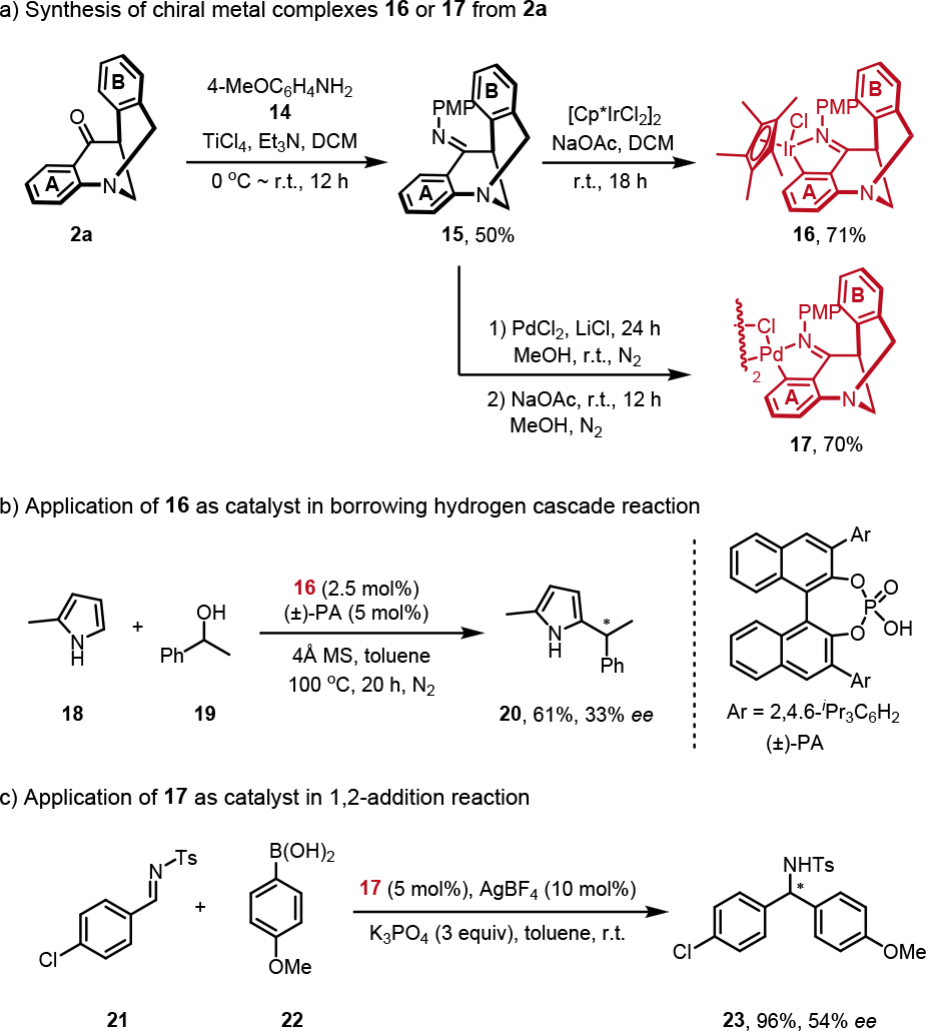
Synthesis of Chiral Metal Complexes **16** or **17** and Their Applications in Borrowing the Hydrogen Cascade Reaction or the 1,2-Addition Reaction

In addition, it is believed that this *N*-bridged [3.3.1] ring product has a rigid V-shaped scaffold, which could be beneficial for asymmetric induction. Thus, we synthesized the Ir and Pd C,N-metallacycles from the corresponding imine **15**. As shown in Scheme 5, the treatment of the imine **15** and [Cp*IrCl_2_]_2_ with NaOAc in CH_2_Cl_2_ successfully afforded the chiral iridium complex **16** in 71% yield, and the treatment of the imine **15** with Li_2_PdCl_4_ in the presence of NaOAc gave the chiral palladium dimer **17** in 70% yield.^70^ These two complexes were then examined in the asymmetric reactions (Scheme 5b). Chiral iridium complex **16** could catalyze the borrowing hydrogen cascade reaction of 2-methylpyrrole **18** with (±)-1-phenylethanol **19**, affording product **20** in moderate yield with some extent of enantio-control.^73^ It should be noted that the enantioselectivity of phosphoric acid did not benefit the enantio-control. Chiral palladium dimer complex **17** was able to catalyze the 1,2-addition reaction of 4-methoxyphenylboronic acid **22** with imine **21**, affording **23** in quantitative yield with moderate enantioselectivity (Scheme 5c).^74,75^ Nonetheless, these preliminary results demonstrate that these rigid chiral scaffolds of *N*-bridged [3.3.1] rings could serve as a new class of chiral metal complexes and could hold promise for more applications in catalytic asymmetric transformations

**Scheme 6:**
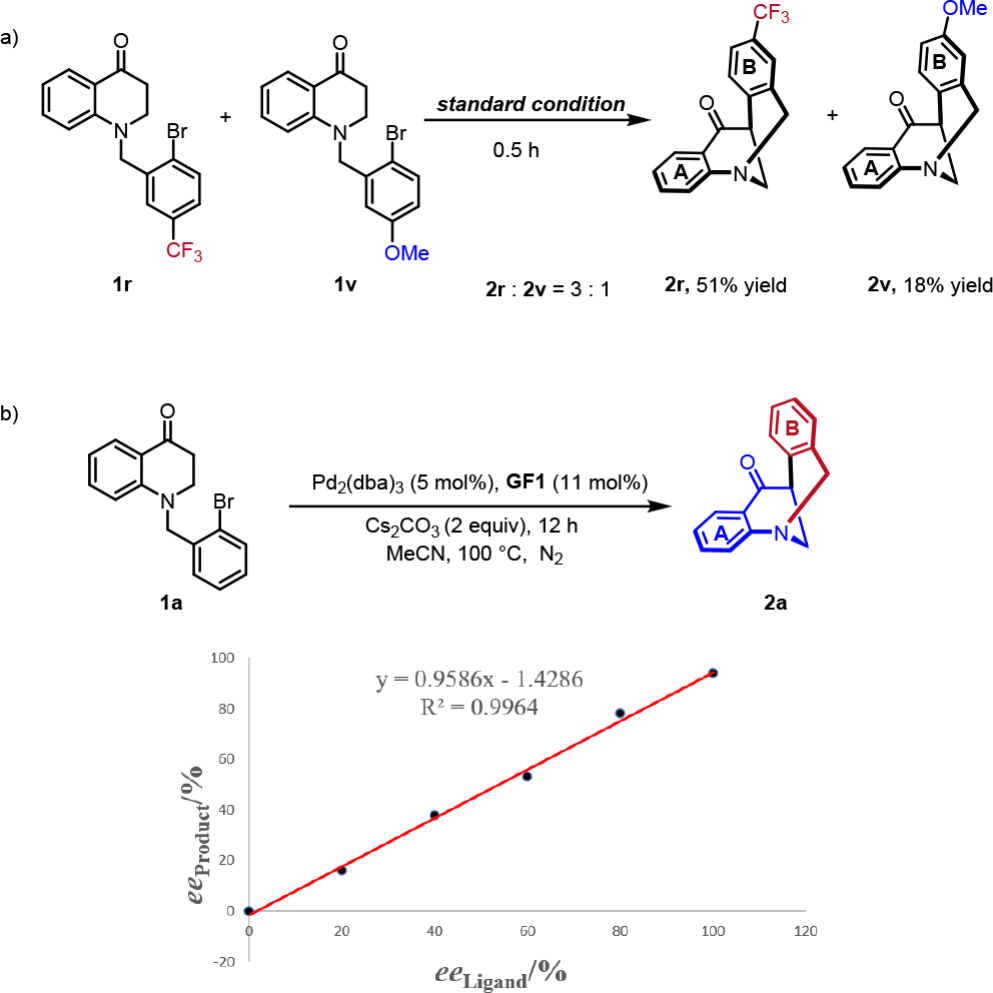
Competitive Experiments and Nonlinear Effects

To gain insight into the mechanism, we first conducted a competitive experiment, which showed that electron-withdrawing substituents on aromatic ring **B** facilitate the reaction **(**Scheme 6a). In addition, a linear relationship between the ee values of **GF1** and product **2a** indicated that one molecule of chiral ligand gets involved in controlling the stereochemistry of this reaction.

**Scheme 7:**
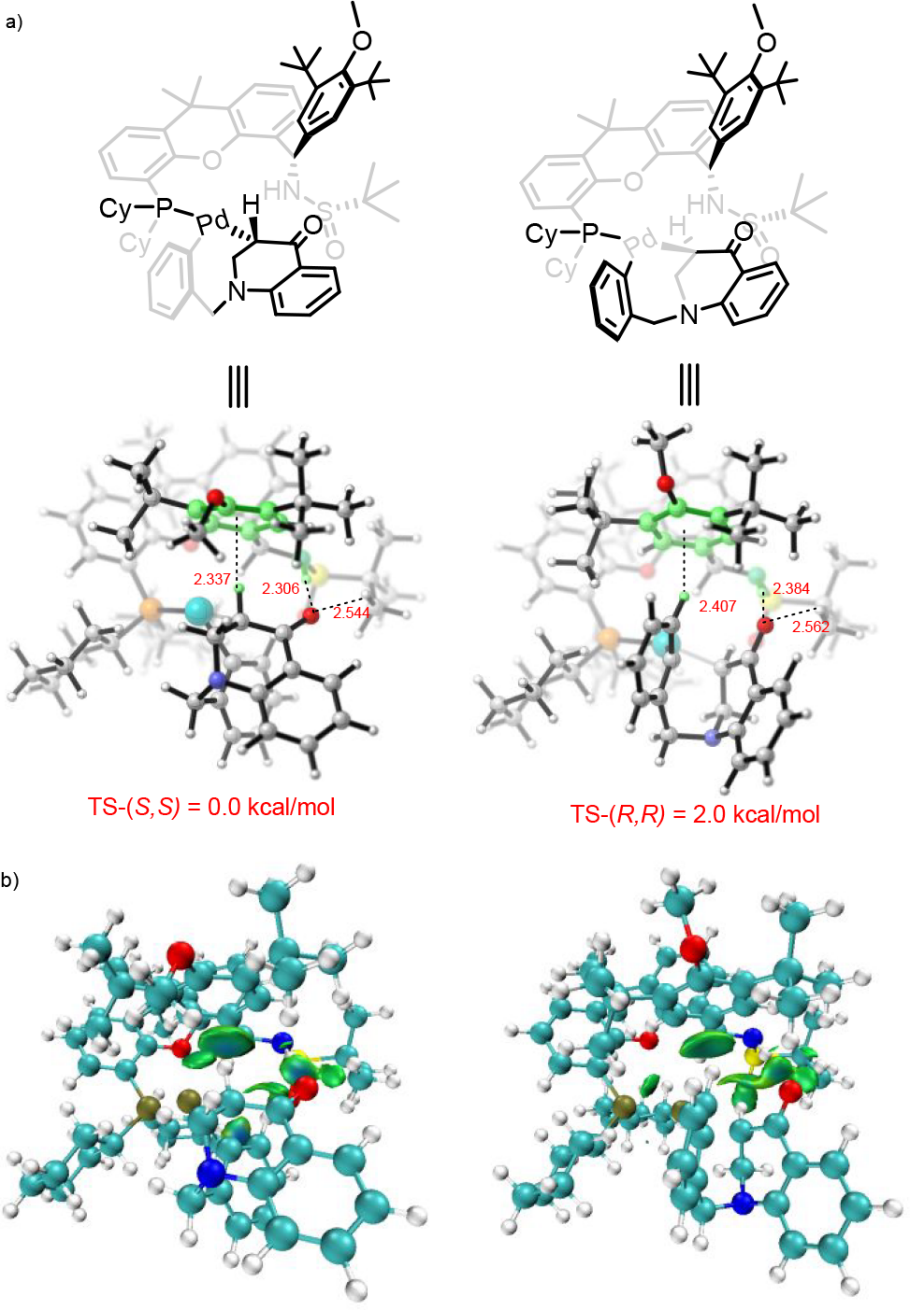
Schematics for the Transition State Structures of Reductive Elimination for Forming (*S,S*)-**2a** and (*R,R*)-**2a** (a) and IGM Analysis Plots (b) For IGM analysis, blue, attraction; green, weak interaction; red, steric effect.

To elucidate the role of ligand in controlling the enantioselectivity, we also conducted DFT calculations on the C–C reductive elimination. The calculation shows that the two transition states differ in free energy of activation by 2.0 kcal/mol favoring the (*S,S*)-product, which is in good agreement with the experiments. Further investigation of the calculated transition-state structures for reductive elimination revealed NH-O hydrogen bond between the oxygen atom in the carbonyl of the substrate fragment and NH group in the ligand. Besides the hydrogen bond, the weak interactions between the substrate C–H with the aromatic ring in the ligand were also observed via independent gradient model (IGM), which displayed blue-green isosurfaces between the aforementioned substrate C–H bonds and the aromatic ring fragment, indicating attractive interactions (Scheme 7a). As for the NH··O hydrogen bond, the contact in the transition state to give the major product enantiomer (TS-(*S,S*), 2.306 Å; Scheme 7a, left) was shorter than that in the transition state to give the minor enantiomer (TS-(*R,R*), 2.384 Å; Scheme 7a, right). Similarly, the same trend was also observed in the distance between the substrate C–H bonds and the aromatic ring aromatic ring fragment in the ligand (2.337 Å for TS-(*S,S*) vs 2.407 Å for TS-(*R,R*)). Similar hydrogen bonding between carbonyl oxygen atoms and other types of CH bonds was also found to be responsible for the high enantioselectivities observed in their Pd-catalyzed asymmetric arylation of silyl ketene acetals, enol ethers and fluorooxindoles.^76−78^ This observation is constant with the experimental result. For example, replacement of the aromatic substituent in the ligand with aliphatic one completely shut down the enantioselectivity (**PC4 and PC5**, Table 1, entries 4 and 5). Similarly, ligand with *N*-Me (**PC3**) also loses control of the enantioselectivity (Table 1, entry 3).

**Scheme 8:**
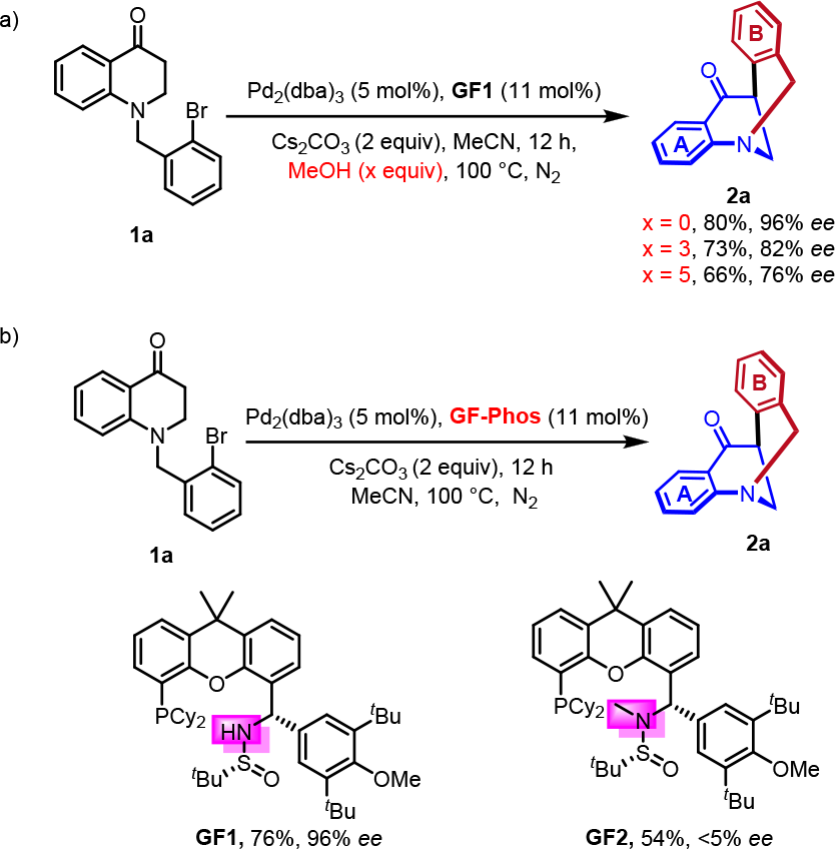
Control Experiments

To further prove these hypotheses, we conducted several control experiments (Scheme 8). First, when the model reaction was performed under standard conditions with the addition of 3 equiv of MeOH, which is known to disrupt hydrogen bonding, the enantioselectivity of the resulting product decreased (from 96% ee to 82% ee), and when the amount of MeOH was increased to 5 equiv, the enantioselectivity further decreased to 76% ee (Scheme 8a). Second, if the *N* atom in the ligand **GF1** was masked with the methyl group, significant loss of enantioselectivity was observed (from 76%, 96% ee to 54%, < 5% ee) (Scheme 8b).

## Conclusion

In conclusion, we have developed an efficient method for the catalytic asymmetric synthesis of rigid cleft-like compounds with nitrogen stereocenters via Pd catalysis and homemade **GF-Phos**. This protocol allows simultaneous construction of a wide range of chiral Tröger’s base analogues, which contain a C- and a N-stereogenic center in high stability, efficiency and selectivity. It provides a new strategy for the catalytic asymmetric synthesis of Tröger’s base analogues from simple starting materials. The synthetic application was demonstrated by its utility as a new class of chiral organocatalysts and chiral transition–metal scaffold precursors in a batch of transformations, including catalytic kinetic resolution of binaphthols, the addition of boronic acids to imines, and the borrowing hydrogen cascade reaction. DFT calculations have revealed that the NH··O hydrogen bonding and weak interaction between the substrate and the ligand are responsible for the high enantioselectivity. This mechanistic insight could stimulate future development for the ligand design. Further investigations on the potential application of the unique chiral scaffold in molecular recognition and material science are in progress and will be reported in due course.

## Supporting Information Available

Experimental procedures, characterization data, NMR spectra, HPLC spectra, density functional theory calculations, and additional data (PDF)
Crystallographic information for (*S*,*S*)-**2a** (CIF)

## Supplementary Material

oc2c01121_si_001.pdf

oc2c01121_si_002.cif
