# Supplementary material for: Catalytic Asymmetric Synthesis of Tröger’s Base Analogues with Nitrogen Stereocenter
Source: ACS Cent Sci. 2023 Jan 4;9(1):64–71. doi: 10.1021/acscentsci.2c01121 (PMC9881208; doi:10.1021/acscentsci.2c01121)
Supplement: Supplementary file 1 — oc2c01121_si_001.pdf [file oc2c01121_si_001.pdf]

Supporting Information

Catalytic Asymmetric Synthesis of Tröger's Base Analogues with Nitrogen Stereocenter

Chun Ma,[a] Yue Sun,[a] Junfeng Yang,[a,c]* Hao Guo,[a,b]*and Junliang Zhang[a,d]*

[a] Department of Chemistry, Fudan University, 2005 Songhu Road, Shanghai, 200438 P. R. China;

[b] Zhuhai Fudan Innovation Institute, Zhuhai, 519000, P. R. China

[c] Fudan Zhangjiang Institute, Shanghai 201203, P. R. China

[d] School of Chemistry and Chemical Engineering, Henan Normal University, Xinxiang, Henan 453007, China.

Junfeng Yang: yangjf@fudan.edu.cn

Hao Guo: hguo@fudan.edu.cn

Junliang Zhang: junliangzhang@fudan.edu.cn

Table of Contents

1. General information ••S3
2. Screening of chiral ligands and condition optimization •••••••••••••••••••••S4
3. General procedure for the synthesis of 1 •••••••••••••••••••••••••••••••••••••S9
4. General procedure for the synthesis of *N*-Me GF-Phos •••••••••••••••••••••S9
5. General procedure for the synthesis of chiral product 2. •••••••••••••••••••S10
6. General procedure for the gram-scale reaction •••••••••••••••••••••••••••••S10
7. General procedure for derivatization of 2g •••••••••••••••••••••••••••••••••S10
8. General procedure for synthesis and application of catalysts •••••••••••••S13
9. Control experiments •••S17
10. Compound characterization •••S20
11. X-ray single crystal data for compounds 2a •••••••••••••••••••••••••••••••S47
12. DFT caculation •••S49
13. ^{1}H, ^{13}C, ^{19}F, ^{31}P NMR Spectra ••S59
14. HPLC Spectra •••S140
15. References ••S178

1. General Information

All reactions were carried out under an atmosphere of argon in sealed tubes with magnetic stirring (speed 1000 rpm). ^{1}H NMR spectra, ^{19}F NMR spectra, ^{31}P NMR spectra, ^{13}C NMR spectra were recorded on a Bruker 400 MHz spectrometer in $CDCl_3$ or DMSO-d_6. All signals are reported in ppm with the internal TMS signal at 7.26 or 2.5 ppm as the internal standard. Data for ^{1}H NMR spectra are reported as follows: chemical shift (ppm, referenced to TMS; s = singlet, d = doublet, t = triplet, dd = doublet of doublets, m = multiplet), coupling constant (Hz), and integration. Data for ^{13}C NMR are reported in terms of chemical shift (ppm) relative to residual solvent peak ($CDCl_3$: 77.0 ppm, DMSO-d_6: 39.5). HRMS (ESI/EI) spectra were recorded on GCQTOF 7200. Reactions were monitored by thin layer chromatography (TLC) using silica gel plates. Flash column chromatography was performed over silica gel (300-400 mesh) or neutral alumina. The enantiomeric excesses of the products were determined by chiral stationary phase HPLC using a Chiralpak AD-H, OD-H, OB-H, IC. All samples tested by chiral stationary phase HPLC were dissolved in *n*-hexane/isopropanol. Optical rotation values were measured with instruments operating at λ = 589 nm, corresponding to the sodium D line at the temperatures indicated. Unless otherwise noted, commercial reagents were purchased from Energy chemical, Bidepharm, Aesar, and other commercial suppliers and were used as received. Chiral ligand **GF-Phos** was synthesized according to the literature.[1] *N*-benzyl substituted dihydroquinolinone derivatives **1** were synthesized according to the literature.[2]

2. Screening of chiral ligands and condition optimization

Table S1. Screening of chiral ligands for coupling reaction[a]

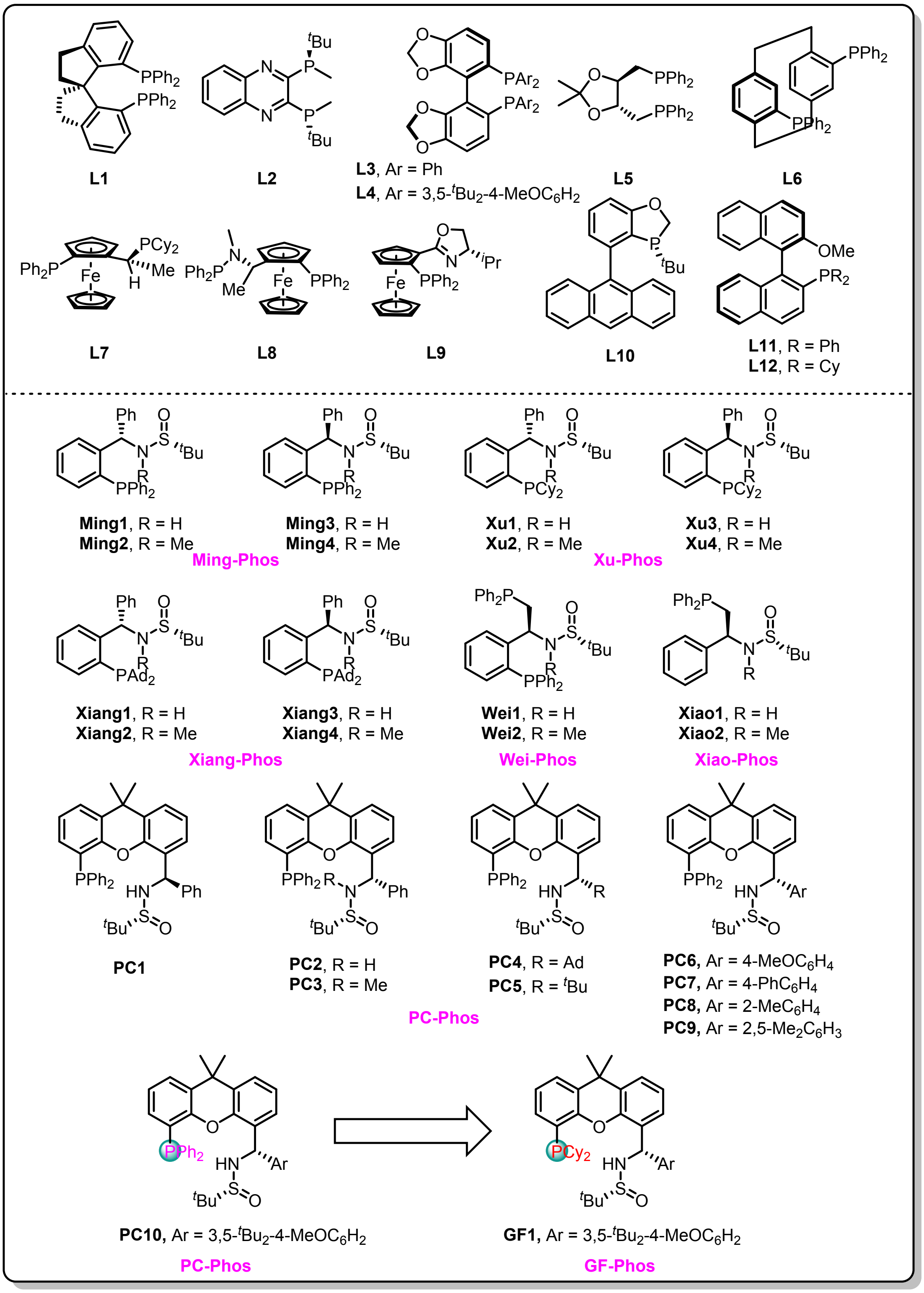

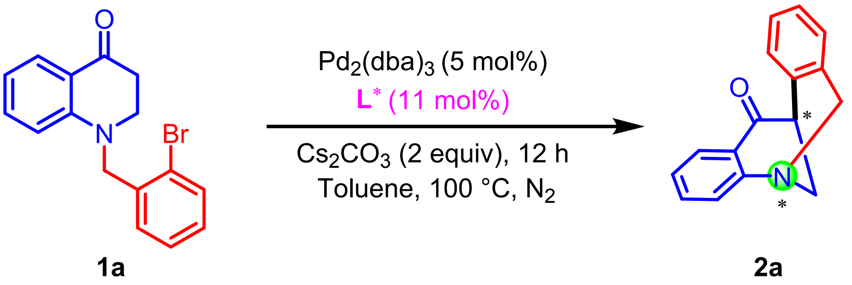

Entry	L*	GC-Yield (%)[b]	*Ee* (%)[c]
1	**L1**	26	3
2	**L2**	15	-84
3	**L3**	n.d.	-
4	**L4**	n.d.	-
5	**L5**	n.d.	-
6	**L6**	23	-20
7	**L7**	n.d.	-
8	**L8**	n.d.	-
9	**L9**	46	-72
10	**L10**	n.d.	-
11	**L11**	n.d.	-
12	**L12**	66	-5
13	**Ming1**	n.d.	-
14	**Ming2**	trace	-
15	**Ming3**	n.d.	-
16	**Ming4**	10	4
17	**Xu1**	n.d.	-
18	**Xu2**	46	-36
19	**Xu3**	n.d.	-
20	**Xu4**	24	28
21	**Xiang1**	29	7
22	**Xiang2**	45	1
23	**Xiang3**	48	-18
24	**Xiang4**	51	-2
25	**Wei1**	trace	-
26	**Wei2**	trace	-
27	**Xiao1**	n. d.	-
28	**Xiao2**	12	-9
29	**PC1**	4	75
30	**PC2**	14	95
31	**PC3**	14	6
32	**PC4**	23	1
33	**PC5**	17	4
34	**PC6**	21	93
35	**PC7**	14	6
36	**PC8**	25	80

37	**PC9**	27	79
38	**PC10**	31	96

[a]Reaction conditions: **1a** (0.1 mmol), Cs_2CO_3 (2.0 equiv), $Pd_2(dba)_3$ (5 mol%) and **L*** (11 mol%) in toluene at 100 °C for 12 h. [b]Determined by GC using tetradecane as an internal standard. [c]The enantiomeric excess (*ee*) was determined by HPLC. n.d. = not detected.

Table S2. Screening of base for coupling reaction[a]

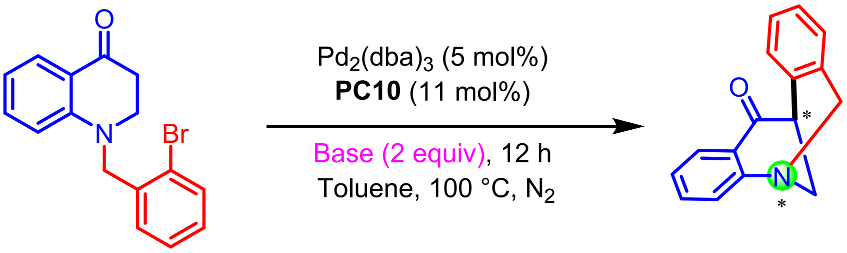

1a **2a**

Entry	Base	GC-Yield (%)[b]	*Ee* (%)[c]
1	Cs_2CO_3	37	95
2	K_2CO_3	36	88
3	NaOAc	n.d.	-
4	NaO*t*Bu	3	15
5	CsOAc	n.d.	-
6	K_3PO_4	34	88
7	KO*t*Bu	n.d.	-
8	HCO_2Na	n.d.	-
9	$KHCO_3$	21	74
10	KOH	21	83
11	Et_3N	n.d.	-
12	DIPEA	n.d.	-
13	DABCO	n.d.	-
14	DBU	n.d.	-
15	*n*Pr_3N	n.d.	-

[a]Reaction conditions: **1a** (0.1 mmol), Base (2.0 equiv), $Pd_2(dba)_3$ (5 mol%) and **PC10** (11 mol%) in toluene at 100 °C for 12 h. [b]Determined by GC using tetradecane as an internal standard. [c]The enantiomeric excess (*ee*) was determined by HPLC. n.d. = not detected.

Table S3. Screening of Pd sources for coupling reaction[a]

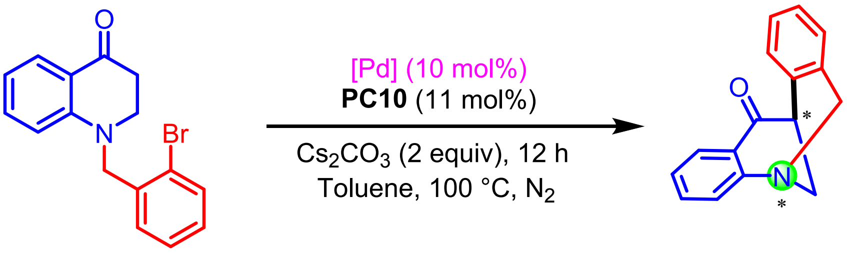

1a **2a**

Entry	Pd sources	GC-Yield (%)[b]	*Ee* (%)[c]
1	$Pd_2(dba)_3$	34	96
2	$Pd_2(dba)_3 \cdot CHCl_3$	39	94

3	$Pd(dba)_2$	39	91
4	$Pd(OAc)_2$	28	93
5	$PdCl_2$	28	89
6	$Pd(TFA)_2$	35	78
7	PdI_2	34	63
8	$PdBr_2$	37	58
9	$Pd(acac)_2$	22	52
10	$[Pd(C_3H_5)Cl]_2$	32	92
11[d]	$Pd_2(dba)_3$	54	97

[a]Reaction conditions: **1a** (0.1 mmol), Cs_2CO_3 (2.0 equiv), [Pd] (5 mol%) and **PC10**(11 mol%) in toluene at 100 °C for 12 h. [b]Determined by GC using tetradecane as an internal standard. [c]The enantiomeric excess (*ee*) was determined by HPLC. [d]Using **GF1** as ligand.

Table S4. Screening of solvent for coupling reaction[a]

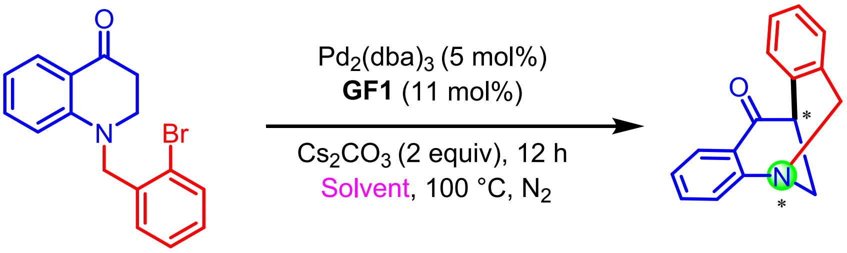

1a **2a**

Entry	Solvent	GC-Yield (%)[b]	*Ee* (%)[c]
1	Toluene	55	97
2	CH_3CN	85	94
3	Dioxane	57	94
4	DCE	83	78
5	DMF	58	81
6	THF	58	96
7	EA	58	96
8	DMSO	42	36
9	MeOH	99	1
10	DPM	n.d.	-

[a]Reaction conditions: **1a** (0.1 mmol), Cs_2CO_3 (2.0 equiv), $Pd_2(dba)_3$ (5 mol%) and **GF1** (11 mol%) in solvent at 100 °C for 12 h. [b]Determined by GC using tetradecane as an internal standard. [c]The enantiomeric excess (*ee*) was determined by HPLC. n.d. = not detected.

Table S5. Screening of temperature for coupling reaction[a]

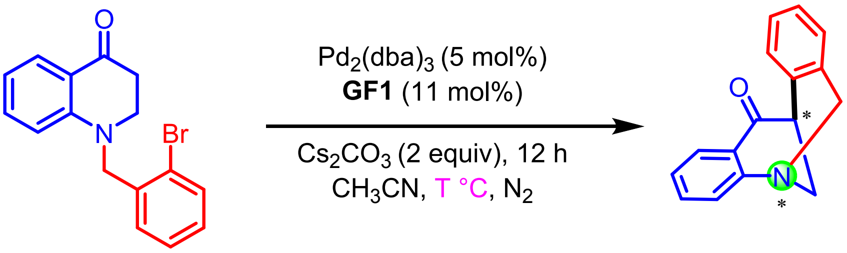

1a **2a**

Entry	T (°C)	GC-Yield (%)[b]	*Ee* (%)[c]

1	100	87	94
2	90	78	94
3	80	68	94
4	70	53	94
5	60	42	93

[a]Reaction conditions: **1a** (0.1 mmol), Cs_2CO_3 (2.0 equiv), $Pd_2(dba)_3$ (5 mol%) and **GF1** (11 mol%) in CH_3CN for 12 h. [b]Determined by GC using tetradecane as an internal standard. [c]The enantiomeric excess (*ee*) was determined by HPLC.

Table S6. Screening of catalyst/ligand loadings for coupling reaction[a]

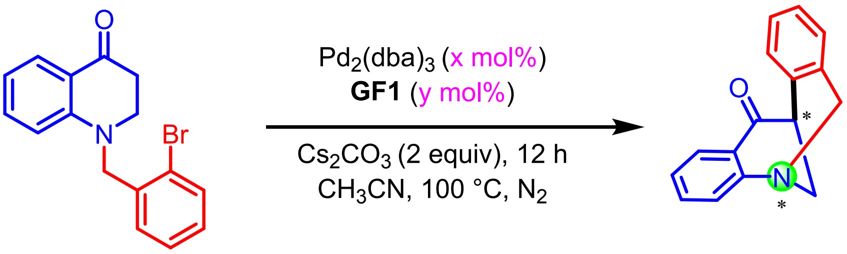

1a **2a**

Entry	x mol%	y mol%	GC-Yield (%)[b]	*Ee* (%)[c]
1	5	5	73	88
2	5	6	74	90
3	5	10	88	93
4	5	11	89	94
5	5	20	89	95
6	5	11	80(77)[d]	96
7	2.5	5.5	66	91
8	1	2.2	27	86

[a]Reaction conditions: **1a** (0.1 mmol), Cs_2CO_3 (2.0 equiv), $Pd_2(dba)_3$ (x mol%) and **GF1** (y mol%) in CH_3CN at 100 °C for 12 h. [b]Determined by GC using tetradecane as an internal standard. [c]The enantiomeric excess (*ee*) was determined by HPLC. [d]Isolated yield.

3. General procedure for the synthesis of 1

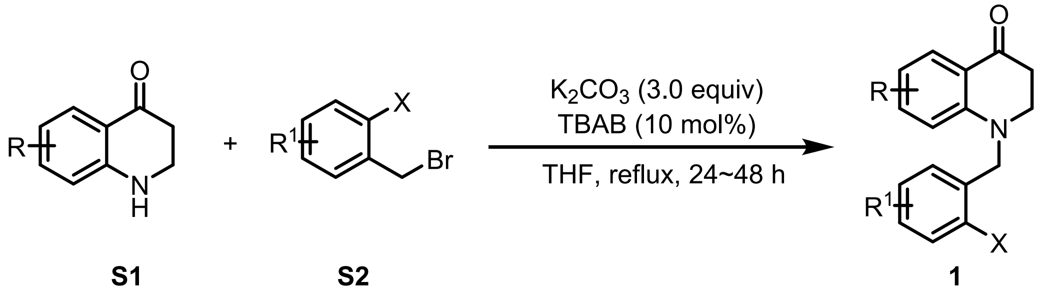

Compounds **S1** (10 mmol), substituted benzyl bromide **S2** (10 mmol), K_2CO_3 (30 mmol) and TBAB (1 mmol) were added to a 100 mL reaction bottle. Then, THF (20 mL) were added to the reaction bottle. The resulting mixture was stirred at 70 °C for 24~48 h in an oil bath. After cooled to room temperature, the mixture was filtered and the solid was washed with EA and further purified by flash column chromatography on silica gel (Petroleum ether: Ethyl acetate) to afford the desired product **1**.

4. General procedure for the synthesis of *N*-Me GF-Phos

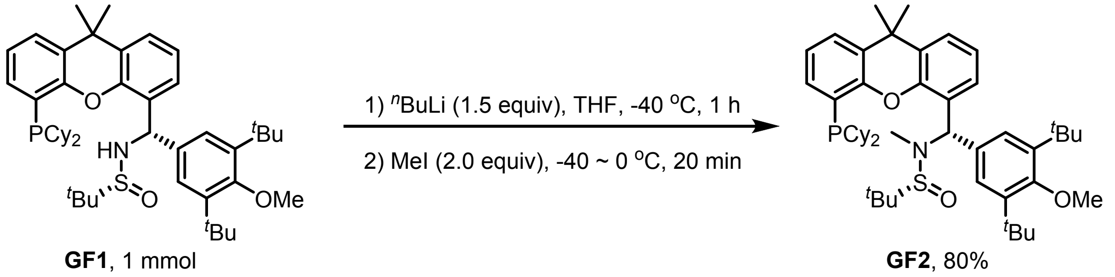

To a solution of **GF1** (1 mmol) in THF (10 mL) was added ^{n}BuLi (1.5 mmol, 0.94 mL, 1.6 M in hexane) dropwise under nitrogen at -40 °C for 1 h. Then, MeI (2 mmol, 0.12 mL) was added to the above system at -40 °C. After the reaction was added, move to 0 °C for 20 minutes. After completion of reaction monitored by TLC analysis, the reaction mixture was quenched by NH_4Cl aqueous solution. The aqueous layer was extracted 3 times with EtOAc, and the combined organic layer was washed with brine, dried over anhydrous Na_2SO_4, and concentrated in vacuo after filtration. The crude product was then purified by flash column chromatography on silica gel (Petroleum ether: Ethyl acetate = 10: 1 to 5: 1) to afford the desired product **GF2**.

5. General procedure for the synthesis of chiral product 2.

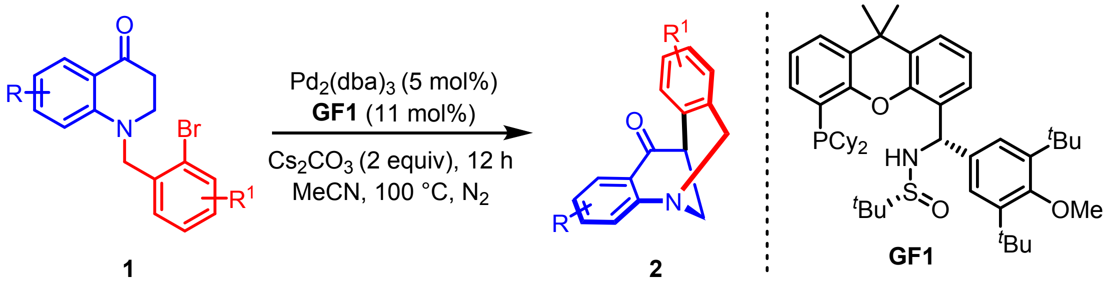

Under a nitrogen atmosphere, compounds **1** (0.3 mmol), $Pd_2(dba)_3$ (5 mol%), **GF1** (11 mol%) and Cs_2CO_3 (0.6 mmol) were added to a dry 10 mL sealed tube. Then, MeCN (3.0 mL) was added to the reaction tube under a nitrogen atmosphere. The resulting mixture was stirred with 1000 rpm at 100 °C in an oil bath. After the reaction was complete as monitored by TLC, the reaction mixture was filtered to remove insoluble and further purified by flash column chromatography on silica gel (Petroleum ether: Ethyl acetate) to afford the desired product **2**.

6. General procedure for the gram-scale reaction

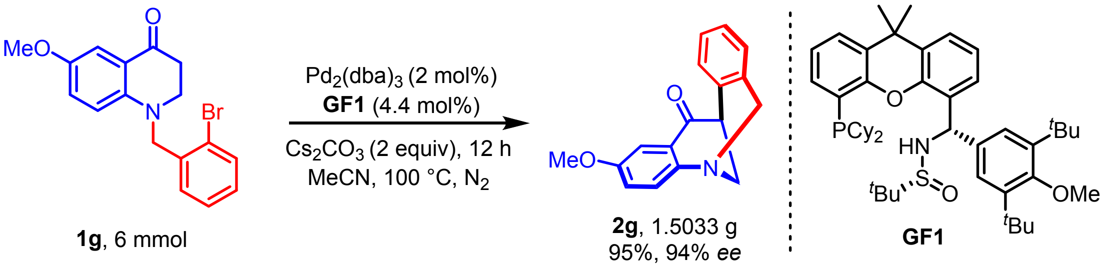

Under a nitrogen atmosphere, compound **1g** (6 mmol, 2.07 g), $Pd_2(dba)_3$ (2 mol%, 110 mg), **GF1** (4.4 mol%, 200 mg) and Cs_2CO_3 (12 mmol, 3.912 g) were added to a dry 125 mL sealed tube. Then, MeCN (60 mL) was added to the reaction tube under a nitrogen atmosphere. The resulting mixture was stirred with 1000 rpm at 100 °C in an oil bath. After the reaction was complete as monitored by TLC, the reaction mixture was filtered to remove insoluble and further purified by flash column chromatography on silica gel (Petroleum ether: Ethyl acetate = 10:1 to 2:1) to afford the desired product **2g**.

7. General procedure for derivatization of 2g[3]

a) The addition reaction of 2g[3a].

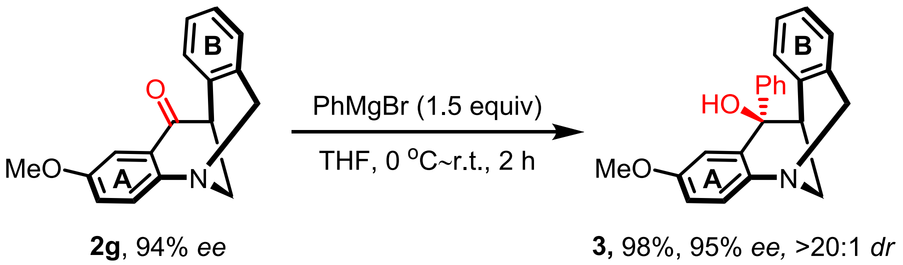

To a solution of **2g** (0.2 mmol, 53 mg) in THF (2 mL) was added PhMgBr (0.3 mmol, 0.3 mL, 1.0 M in THF) dropwise under nitrogen at 0 °C. Then, it was moved to room temperature to continue the reaction for 2 h. After completion of reaction monitored by TLC analysis, the

reaction mixture was quenched by NH_4Cl aqueous solution. The aqueous layer was extracted 3 times with EtOAc and dried over anhydrous Na_2SO_4, and concentrated in vacuo after filtration. The crude product was then purified by flash column chromatography on silica gel (Petroleum ether: Ethyl acetate = 10: 1 to 2: 1) to afford the desired product **3** (67.4 mg, 98%, 95% *ee*, >20:1 *dr*) as a white solid. m.p. 87.8-88.2 °C. **^{1}H NMR** (400 MHz, $CDCl_3$) δ 7.54 (d, *J* = 6.9 Hz, 1H), 7.44 (d, *J* = 7.3 Hz, 2H), 7.40 – 7.34 (m, 2H), 7.33 – 7.26 (m, 2H), 7.26 – 7.21 (m, 1H), 7.14 (d, *J* = 8.3 Hz, 1H), 7.02 (d, *J* = 6.9 Hz, 1H), 6.90 – 6.77 (m, 2H), 4.62 (d, *J* = 16.8 Hz, 1H), 4.21 (d, *J* = 16.8 Hz, 1H), 3.65 (s, 3H), 3.56 (d, *J* = 13.7 Hz, 1H), 3.28 (dd, *J* = 13.7, 3.1 Hz, 1H), 2.99 (s, 1H), 2.29 (s, 1H). **^{13}C NMR** (100 MHz, $CDCl_3$) δ 156.2, 147.6, 142.0, 135.4, 134.5, 133.9, 132.0, 127.9, 127.8, 127.7, 127.3, 126.9, 126.2, 125.4, 116.5, 112.2, 77.7, 60.3, 55.3, 48.3, 42.2. **HPLC conditions**: Daicel Chiralpak OD-H column (hexane/ isopropanol = 90/ 10, flow rate 1.0 mL/min, 254 nm); t_R = 13.448 (major), t_R = 15.873 (minor), 95% *ee*. HRMS (ESI): m/z: Calcd for $(C_{23}H_{21}NO_2+H)^+$ 344.1645, found 344.1643. $[\alpha]_D^{20}$ = +93.617 (c = 0.323, $CHCl_3$).

b) The Wittig reaction of 2g[3b].

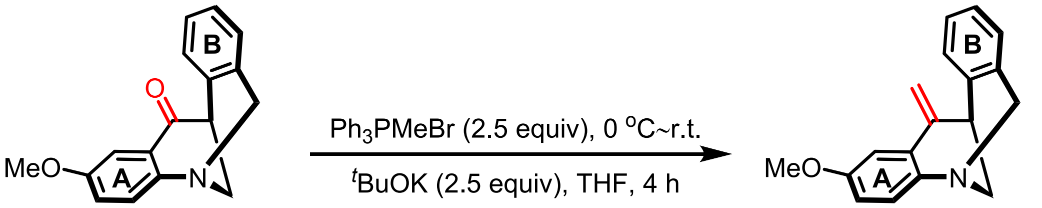

2g, 94% *ee* **4**, 75%, 95% *ee*

To a suspension of methyltriphenylphosphonium briomide (0.5 mmol, 178.5 mg) in dry THF (1 ml) was added a suspension of ^{t}BuOK (0.5 mmol, 56 mg) in dry THF (1 ml), and then the mixture was stirred for 1 h at room temperature. A solution of **2g** (0.2 mmol, 53mg) in dry THF (0.5 ml) was added to the mixture and it was stirred for an additional 4 h at room temperature under nitrogen atmosphere. After completion of reaction monitored by TLC analysis, the reaction mixture was quenched by NH_4Cl aqueous solution. The aqueous layer was extracted 3 times with EtOAc and dried over anhydrous Na_2SO_4, and concentrated in vacuo after filtration. The crude product was then purified by flash column chromatography on silica gel (Petroleum ether: Ethyl acetate = 5: 1) to afford the desired product **4** (39.7 mg, 75%, 95% *ee*) as yellow oil. **^{1}H NMR** (400 MHz, $CDCl_3$) δ 7.26 – 7.20 (m, 1H), 7.19 – 7.01 (m, 4H), 6.95 – 6.88 (m, 1H), 6.85 – 6.74 (m, 1H), 5.48 (s, 1H), 5.21 (s, 1H), 4.64 (d, *J* = 16.8 Hz, 1H), 4.19 (d, *J* = 16.8 Hz, 1H), 3.74 (s, 3H), 3.59 – 3.45 (m, 3H). **^{13}C NMR** (100 MHz, $CDCl_3$) δ 155.7, 145.3, 141.8, 137.5, 132.9, 129.0, 127.5, 127.2, 126.5, 126.4, 126.3, 116.2, 108.1, 106.4, 59.6, 55.4, 51.0, 39.3. **HPLC conditions**: Daicel Chiralpak OD-H column (hexane/ isopropanol = 90/ 10, flow rate 1.0 mL/min, 254 nm); t_R = 8.117 (major), t_R = 13.761 (minor), 95% *ee*. HRMS (ESI): m/z: Calcd for $(C_{18}H_{17}NO+H)^+$ 264.1383, found 264.1388. $[\alpha]_D^{20}$ = +228.129 (c = 0.786, $CHCl_3$).

c) The condensation reaction of 2g[3c].

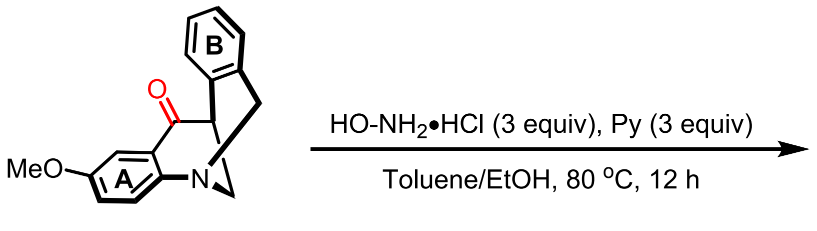

2g, 94% *ee* **5,** 59%, 94% *ee*

A mixture of **2g** (0.2 mmol, 53 mg), $NH_2OH·HCl$ (0.6 mmol, 41.4 mg), and pyridine (0.1 mL) in ethanol and toluene (1 mL, 1/1 v/v) was stirred at 80 °C for 12 h. After it was cooled to room temperature, the mixture was added to ethyl acetate and extracted with HCl (1 N). The organic layer was washed with brine and dried over Na_2SO_4, and concentrated in vacuo after filtration. The crude product was then purified by flash column chromatography on silica gel (Petroleum ether: Ethyl acetate = 5: 1) to afford the desired product **5** (32.9 mg, 59%, 94% *ee*) as a yellow solid. m.p. 201.8-202.4 °C. **^{1}H NMR** (400 MHz, $CDCl_3$) δ 9.05 (s, 1H), 7.63 – 7.50 (m, 1H), 7.35 – 7.30 (m, 1H), 7.21 – 7.12 (m, 3H), 6.98 – 6.86 (m, 2H), 4.78 – 4.66 (m, 2H), 4.20 (d, *J* = 17.0 Hz, 1H), 3.75 (s, 3H), 3.65 – 3.50 (m, 2H). **^{13}C NMR** (100 MHz, $CDCl_3$) δ 156.1, 154.8, 143.1, 134.9, 132.9, 129.7, 127.4, 126.9, 126.6, 126.5, 124.1, 118.7, 106.8, 58.1, 55.4, 49.7, 28.3. **HPLC conditions**: Daicel Chiralpak OD-H column (hexane/ isopropanol = 90/ 10, flow rate 1.0 mL/min, 254 nm); t_R = 10.302 (minor), t_R = 12.033 (major), 94% *ee*. HRMS (ESI): m/z: Calcd for $(C_{17}H_{16}N_2O_2+H)^+$ 281.1285, found 281.1285. $[\alpha]_D^{20}$ = +323.116 (c = 1.18, $CHCl_3$).

d) The oxidation of 2g[3d].

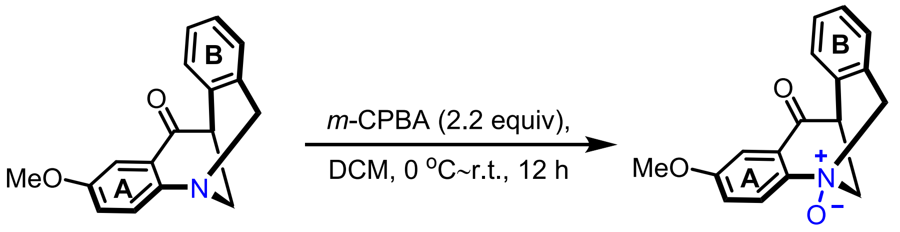

2g, 94% *ee* **6,** 81%, 96% *ee*

To the solution of product **2g** (0.2 mmol, 53 mg) in DCM (5 mL) was added *m*-CPBA (0.44 mmol, 76.1 mg) in an ice bath under magnetic stirring. Then, the resulting mixture was stirred for 12 h at room temperature. After the reaction was complete (monitored by TLC), the crude was diluted with more DCM and quenched with a $NaHCO_3$ aqueous solution. After 2 extractions and removal of water with anhydrous Na_2SO_4, the crude product was then purified by flash column chromatography on silica gel (Dichloromethane: Methanol = 50:1) to afford the desired product **6** (45.3 mg, 81%, 96% *ee*) as a white solid. m.p. 130.0-130.7 °C. **^{1}H NMR** (400 MHz, $CDCl_3$) δ 8.43 (d, *J* = 9.0 Hz, 1H), 7.46 – 7.40 (m, 1H), 7.32 – 7.27 (m, 2H), 7.26 – 7.20 (m, 2H), 7.01 – 6.95 (m, 1H), 5.08 (d, *J* = 16.0 Hz, 1H), 4.79 (d, *J* = 16.0 Hz, 1H), 4.40 (d, *J* = 11.9 Hz, 1H), 4.27 – 4.17 (m, 2H), 3.79 (s, 3H). **^{13}C NMR** (100 MHz, $CDCl_3$) δ 190.2, 160.1, 147.3, 129.9, 129.1, 129.0, 128.9, 127.5, 126.6, 124.6, 124.4, 123.9, 108.4, 75.5, 67.9, 55.8, 50.6. **HPLC conditions**: Daicel Chiralpak IC column (hexane/ isopropanol = 90/ 10, flow rate 1.0 mL/min, 254 nm); t_R = 10.146 (minor), t_R = 14.136 (major), 96% *ee*. HRMS (ESI): m/z: Calcd for $(C_{17}H_{15}NO_3+H)^+$ 282.1125, found 282.1127. $[\alpha]_D^{20}$ = +43.423 (c = 0.432, $CHCl_3$).

(*Note: Determination of ee value of* ***6****: Dissolve* ***6*** *and phenylboronic acid in dichloromethane and stir for 5 minutes, the equivalent is converted into* ***2g****, and then the ee value is determined.*)

e) The reduction of 2g[3e].

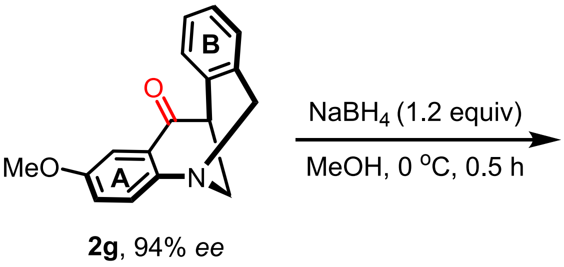

To the solution of product **2g** (0.2 mmol, 53 mg) in MeOH (2 mL) was added $NaBH_4$ (0.24 mmol, 9.2 mg) in an ice bath under magnetic stirring. Then, the resulting mixture was stirred for 0.5 h at this temperature. After completion of reaction monitored by TLC analysis, the reaction mixture was quenched by H_2O. The aqueous layer was extracted 3 times with EtOAc and dried over anhydrous Na_2SO_4, and concentrated in vacuo after filtration. The crude product was then purified by flash column chromatography on silica gel (Petroleum ether: Ethyl acetate = 6: 1 to 1: 1) to afford the desired product **7** (42.2 mg, 79%, 96% *ee*, >20:1 *dr*) as a yellowish solid. m.p. 165.4-165.9 °C. **^{1}H NMR** (400 MHz, $CDCl_3$) δ 7.27 – 7.24 (m, 1H), 7.21 – 7.10 (m, 2H), 7.06 – 6.91 (m, 3H), 6.73 (dd, *J* = 8.7, 2.6 Hz, 1H), 5.01 (d, *J* = 6.2 Hz, 1H), 4.58 (d, *J* = 16.8 Hz, 1H), 4.12 (d, *J* = 16.8 Hz, 1H), 3.70 (s, 3H), 3.44 (s, 2H), 3.03 (d, *J* = 6.0 Hz, 1H), 2.06 (s, 1H). **^{13}C NMR** (100 MHz, $CDCl_3$) δ 156.2, 141.3, 134.2, 133.9, 132.8, 130.9, 127.5, 126.7, 126.2, 125.4, 115.3, 111.2, 71.1, 60.1, 55.3, 50.1, 34.7. **HPLC conditions**: Daicel Chiralpak AD-H column (hexane/ isopropanol = 90/ 10, flow rate 1.0 mL/min, 254 nm); t_R = 14.781 (minor), t_R = 16.561 (major), 95% *ee*. HRMS (ESI): m/z: Calcd for $(C_{17}H_{17}NO_2+H)^+$ 268.1332, found 268.1338. $[\alpha]_D^{20}$ = +48.013 (c = 0.62, $CHCl_3$).

8. General procedure for synthesis and application of catalysts[4]

a) Synthesis of chiral organocatalyst 10 and its application in catalytic kinetic resolution[4a]

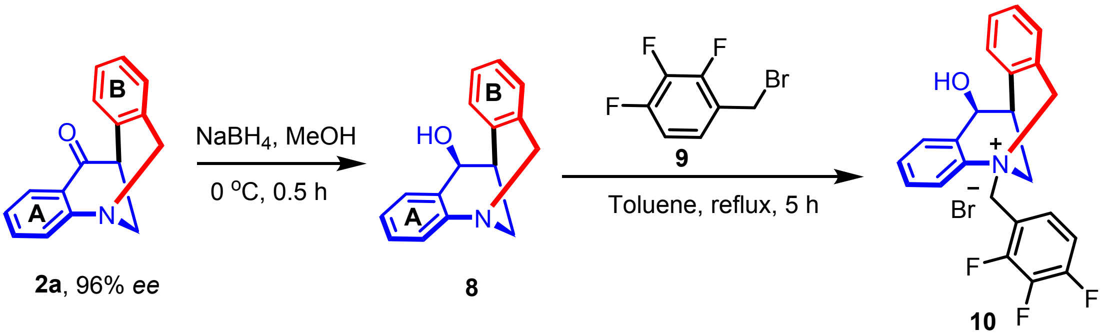

To the solution of product **2a** (1.0 mmol, 235.2 mg) in MeOH (10 mL) was added $NaBH_4$ (1.2 mmol, 45.6 mg) in an ice bath under magnetic stirring. Then, the resulting mixture was stirred for 0.5 h at this temperature. After completion of reaction monitored by TLC analysis, the reaction

mixture was quenched by H_2O. The aqueous layer was extracted 3 times with EtOAc and dried over anhydrous Na_2SO_4, and concentrated in vacuo after filtration. The product **8** was used directly in the next step without purification. Substituted benzyl bromide **9** (1.15 mmol, 260 mg) was added to a solution of **8** in toluene (3 mL). The mixture was heated under reflux for 5 h and then allowed to cool to room temperature. The resulting yellowish solid was collected by filtration and washed with diethyl ether to afford **10** (230 mg, 50% two-step yield) as a yellowish solid. m.p. 122.0-122.8 ^{o}C. **^{1}H NMR** (400 MHz, DMSO-d_6) δ 8.39 (d, *J* = 8.3 Hz, 1H), 7.58 – 7.52 (m, 1H), 7.51 – 7.44 (m, 2H), 7.40 – 7.34 (m, 2H), 7.26 – 7.20 (m, 2H), 7.19 – 7.13 (m, 1H), 7.12 – 7.05 (m, 1H), 5.57 (s, 2H), 5.38 (d, *J* = 15.0 Hz, 1H), 5.10 (dd, *J* = 18.8, 10.1 Hz, 2H), 4.33 – 4.07 (m, 2H), 3.62 (s, 1H), 2.30 (s, 1H). **^{13}C NMR** (100 MHz, DMSO-d_6) δ 137.9, 136.0, 131.6, 130.6, 129.6, 128.9, 128.7, 128.2, 127.7, 127.5, 127.3, 125.7, 125.3, 121.7, 113.2, 113.1, 113.0, 67.4, 67.1, 61.8, 57.0, 36.5.

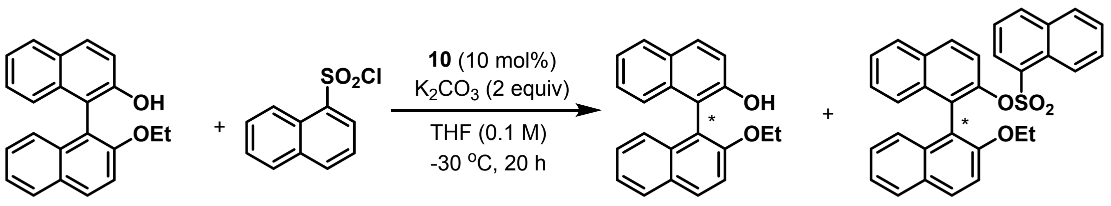

(±)-**11** | **12** 0.58 equiv | **11** 34%, 68% *ee* | **13** 47%, 48% *ee* *Conv.* 59%, *S* = 6

Binaphthol derivative *rac*-**11** (0.1 mmol, 31.4 mg), 1-naphthalenesulfonyl chloride **12** (0.058 mmol, 13.1 mg), chiral quaternary ammonium salt **10** (4.6 mg, 0.01 mmol) and K_2CO_3 (0.2 mmol, 27.6 mg) were added to a reaction tube. Then, THF (1.0 mL) pre-cooled to -30 ^{o}C was added to the reaction mixture, which was stirred at -30 ^{o}C for 20 h. After the completion of the reaction which was indicated by TLC, further purified through preparative thin layer chromatography (Petroleum ether: Dichloromethane = 2: 1) on silica gel to afford pure product **13** (23.7 mg, 47%, 48% *ee*) and recovered **11** (10.6 mg, 34%, 68% *ee*).

2'-ethoxy-[1,1'-binaphthalen]-2-yl naphthalene-1-sulfonate (13):

A white solid. m.p. 137.7-138.2 ^{o}C. **^{1}H NMR** (400 MHz, $CDCl_3$) δ 8.08 – 7.96 (m, 2H), 7.91 (d, *J* = 8.2 Hz, 1H), 7.83 (d, *J* = 9.0 Hz, 1H), 7.74 – 7.67 (m, 2H), 7.67 – 7.62 (m, 1H), 7.47 (d, *J* = 8.1 Hz, 1H), 7.45 – 7.38 (m, 2H), 7.24 (s, 1H), 7.22 – 7.14 (m, 3H), 7.07 – 7.02 (m, 1H), 7.01 – 6.94 (m, 2H), 6.81 (d, *J* = 8.5 Hz, 1H), 6.58 (d, *J* = 9.0 Hz, 1H), 3.84 – 3.69 (m, 2H), 1.03 (t, *J* = 7.0 Hz, 3H). **^{13}C NMR** (100 MHz, $CDCl_3$) δ 153.8, 146.3, 134.5, 133.4 (overlap), 133.3, 132.2, 132.1, 129.6, 129.2, 128.9, 128.1, 128.0, 127.9, 127.8, 127.7, 127.6, 126.7, 126.4, 126.3, 126.2, 126.0, 125.8, 125.0, 124.5, 123.3, 122.9, 122.7, 116.1, 113.2, 64.0, 14.8. **HPLC conditions**: Daicel Chiralpak AD-H column (hexane/ isopropanol = 90/ 10, flow rate 1.0 mL/min, 254 nm); t_R = 9.104 (minor), t_R = 9.765 (major), 48% *ee*. HRMS (ESI): m/z: Calcd for $(C_{32}H_{24}O_4S+Na)^+$ 527.1288, found 527.1301. $[\alpha]_D^{20}$ = -11.16 (c = 0.474, $CHCl_3$).

2'-ethoxy-[1,1'-binaphthalen]-2-ol (11)

A white solid. m.p. 132.9-133.3 ^{o}C. **^{1}H NMR** (400 MHz, $CDCl_3$) δ 8.02 (d, *J* = 9.0 Hz, 1H), 7.94 – 7.87 (m, 3H), 7.47 (d, *J* = 9.1 Hz, 1H), 7.42 – 7.37 (m, 2H), 7.35 – 7.27 (m, 2H), 7.26 – 7.20 (m, 2H), 7.11 (d, *J* = 8.4 Hz, 1H), 5.02 (s, 1H), 4.15 – 4.03 (m, 2H), 1.13 (t, *J* = 7.0 Hz, 3H). **^{13}C**

NMR (100 MHz, $CDCl_3$) δ 155.3, 151.2, 134.1, 133.8, 130.8, 129.6, 129.5, 129.1, 128.1, 128.0, 127.2, 126.2, 125.0, 124.9, 124.2, 123.1, 117.5, 116.4, 115.6, 115.2, 65.2, 14.8. **HPLC conditions**: Daicel Chiralpak AD-H column (hexane/ isopropanol = 90/ 10, flow rate 1.0 mL/min, 254 nm); t_R = 8.825 (minor), t_R = 21.297 (major), 68% *ee*. HRMS (ESI): m/z: Calcd for $(C_{22}H_{18}O_2+Na)^+$ 337.1199, found 337.1191. $[\alpha]_D^{20}$ = -55.408 (c = 0.17, $CHCl_3$).

b) Synthesis of chiral metal complexes 16 or 17 [3c]

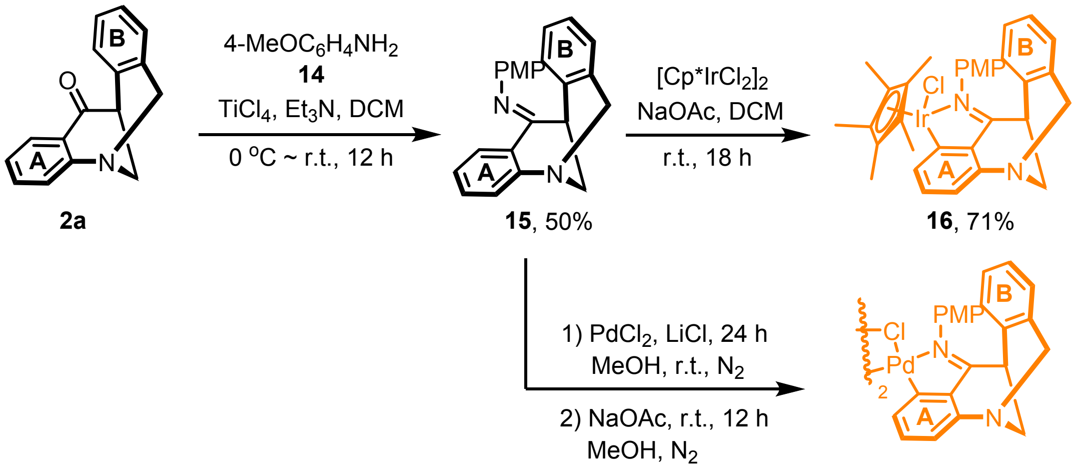

2a **15**, 50% **16**, 71% **17**, 70%

A solution of titanium (IV) chloride in dichloromethane (0.25 mL, 1.0 M in DCM) was added to a mixture of **2a** (1 mmol, 235 mg), 4-methoxyaniline (1.2 mmol, 147.6 mg), triethylamine (1.3 mmol, 0.18 mL), and dichloromethane (5 mL) at 0 °C dropwise. After 10 h, the reaction mixture was quenched with water and extracted with dichloromethane. The combined organic layers were washed with brine, dried over Na_2SO_4, and concentrated. The residue was purified by column chromatography on neutral alumina (Petroleum ether: Ethyl acetate = 5:1) to afford product imine **15** (170 mg, 50%).

Product imine **15** (0.2 mmol, 68 mg), $[Cp^*IrCl_2]_2$ (0.10 mmol, 79.7 mg), and NaOAc (1.0 mmol, 82.0 mg) in dichloromethane (4 mL) were stirred at room temperature for 10 h in air. The residue was purified by column chromatography on silica gel (Petroleum ether: Ethyl acetate = 3:1) to afford **16** (99.8 mg, 71%) as a red solid. **^{1}H NMR** (400 MHz, $CDCl_3$) δ 7.98 (d, *J* = 8.8 Hz, 1H), 7.37 (d, *J* = 7.3 Hz, 1H), 7.17 – 7.08 (m, 2H), 7.07 – 7.02 (m, 1H), 6.99 – 6.92 (m, 2H), 6.92 – 6.85 (m, 1H), 6.80 (d, *J* = 7.6 Hz, 1H), 6.71 (d, *J* = 7.7 Hz, 1H), 6.37 (d, *J* = 7.7 Hz, 1H), 4.66 (d, *J* = 16.9 Hz, 1H), 4.23 (d, *J* = 16.9 Hz, 1H), 4.09 (s, 1H), 3.91 (s, 3H), 3.88 (s, 1H), 3.52 (d, *J* = 12.9 Hz, 1H), 1.43 (s, 15H). **^{13}C NMR** (100 MHz, $CDCl_3$) δ 182.5, 168.5, 157.9, 152.2, 142.9, 139.2, 133.9, 133.7, 132.2, 130.5, 129.0, 128.0, 126.9, 126.6, 126.5, 123.4, 116.3, 115.3, 112.7, 88.7, 57.9, 55.6, 51.7, 35.6, 8.6. HRMS (ESI): m/z: Calcd for $(C_{33}H_{34}ClIrN_2O-Cl)^+$ Calcd for: 667.2297, found 667.2307.

A solution of titanium (IV) chloride in dichloromethane (0.25 mL, 1.0 M in DCM) was added to a mixture of **2a** (1 mmol, 235 mg), 4-methoxyaniline (1.2 mmol, 147.6 mg), triethylamine (1.3 mmol, 0.18 mL), and dichloromethane (5 mL) at 0 °C dropwise. After 10 h, the reaction mixture

was quenched with water and extracted with dichloromethane. The combined organic layers were washed with brine, dried over Na_2SO_4, and concentrated. The residue was purified by column chromatography on neutral alumina (Petroleum ether: Ethyl acetate = 5:1) to afford product imine **15** (170 mg, 50%).

A mixture of $PdCl_2$ (0.50 mmol, 88.7 mg,) and LiCl (1.0 mmol, 43.0 mg,) in methanol (5 mL) was stirred at room temperature for 24 h, and then a solution of product imine **15** (0.5 mmol, 170 mg) in methanol and NaOAc (0.55 mmol, 45.1 mg) was added to the mixture and stirred for another 12 h. The mixture was purified by column chromatography on silica gel (dichloromethane/methanol 50/1) to afford **17** (169 mg, 70%) as a yellow solid. **^{1}H NMR** (400 MHz, $CDCl_3$) δ 7.18 – 6.69 (m, 10H), 6.36 (d, *J* = 7.7 Hz, 1H), 4.60 (d, *J* = 17.0 Hz, 1H), 4.16 (d, *J* = 17.0 Hz, 1H), 3.91 (s, 1H), 3.88 (s, 3H), 3.77 (d, *J* = 13.1 Hz, 1H), 3.42 (dd, *J* = 13.1, 1.9 Hz, 1H). **^{13}C NMR** (100 MHz, $CDCl_3$) δ 182.9, 158.2, 151.8, 138.5, 138.2, 132.5, 132.1, 132.0, 129.0, 128.8, 128.5, 127.5, 127.0, 126.8, 123.7, 119.8, 113.7, 57.0, 55.5, 51.1, 36.3.

c) their application in borrowing hydrogen cascade reaction[4b] or in 1,2-addition reaction[4c].

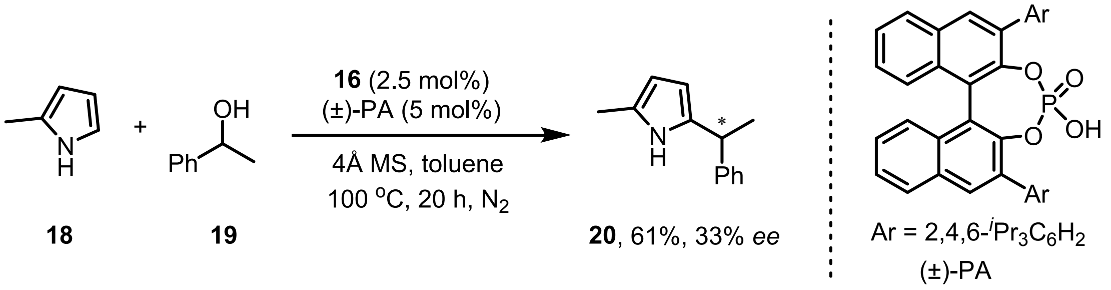

Under a nitrogen atmosphere, chiral iridium complex **16** (0.005 mmol, 3.5 mg), *rac*-PA (0.01 mmol, 7.5 mg) and 4 Å molecular sieves (20 mg) were added to a dry 10 mL sealed tube. Then, 2-methylpyrrole **18** (0.2 mmol, 16.2 mg) and 1-phenylethanol **19** (0.4 mmol, 48.8 mg) will be dissolved in dry toluene (0.5 mL) were added to the reaction tube under a nitrogen atmosphere. The resulting mixture was stirred with 1400 rpm at 100 °C for 20 h in an oil bath. The resulting mixture was cooled to room temperature and then purified by column chromatography (Petroleum ether: Ethyl acetate = 20:1) to provide the desired product **20** (22.7 mg, 61%, 33% *ee*) as colorless oil. **^{1}H NMR** (400 MHz, $CDCl_3$) δ 7.39 (s, 1H), 7.35 – 7.31 (m, 2H), 7.26 – 7.21 (m, 3H), 5.97 – 5.93 (m, 1H), 5.85 – 5.78 (m, 1H), 4.06 (q, *J* = 7.1 Hz, 1H), 2.19 (s, 3H), 1.61 (d, *J* = 7.1 Hz, 3H). **^{13}C NMR** (100 MHz, $CDCl_3$) δ 145.8, 134.7, 128.6, 127.4, 126.8, 126.4, 105.4, 104.8, 38.7, 21.5, 13.0. **HPLC conditions**: Daicel Chiralpak OB-H column (hexane/ isopropanol = 90/ 10, flow rate 1.0 mL/min, 220 nm); t_R = 9.955 (major), t_R = 12.223 (minor), 33% *ee*.

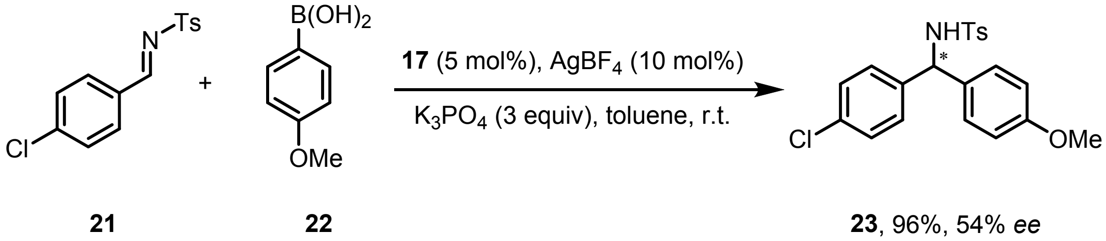

A mixture of imine **21** (0.1 mmol, 29.3 mg), 4-methoxyphenylboronic acid **22** (0.2 mmol, 30.4 mg), chiral palladium complex **17** (0.005 mmol, 4.9 mg), $AgBF_4$ (0.01 mmol, 2.0 mg), and K_3PO_4 (0.3 mmol, 63.6 mg) in toluene (1.0 mL) was stirred at room temperature for 8 h. The mixture was purified by column chromatography on silica gel (Petroleum ether: Ethyl acetate = 10:1) to afford **23** (38.6 mg, 96%, 54% *ee*) as a white solid. m.p. 87.6-87.8 ^{o}C. **^{1}H NMR** (400 MHz, $CDCl_3$) δ 7.55 (d, J = 8.3 Hz, 2H), 7.19 – 7.12 (m, 4H), 7.06 (d, J = 8.4 Hz, 2H), 6.94 (d, J = 8.6 Hz, 2H), 6.77 – 6.67 (m, 2H), 5.49 (d, J = 7.0 Hz, 1H), 5.12 (d, J = 6.9 Hz, 1H), 3.75 (s, 3H), 2.40 (s, 3H). **^{13}C NMR** (100 MHz, $CDCl_3$) δ 159.2, 143.4, 139.2, 137.2, 133.3, 132.3, 129.4, 128.7, 128.6, 128.5, 127.2, 114.1, 60.2, 55.3, 21.5. **HPLC conditions**: Daicel Chiralpak OD-H column (hexane/ isopropanol = 80/ 20, flow rate 1.0 mL/min, 220 nm); t_R = 11.937 (minor), t_R = 15.565 (major), 54% ee.

9. Control experiments

a) Exploring the effect of halogens on the reaction

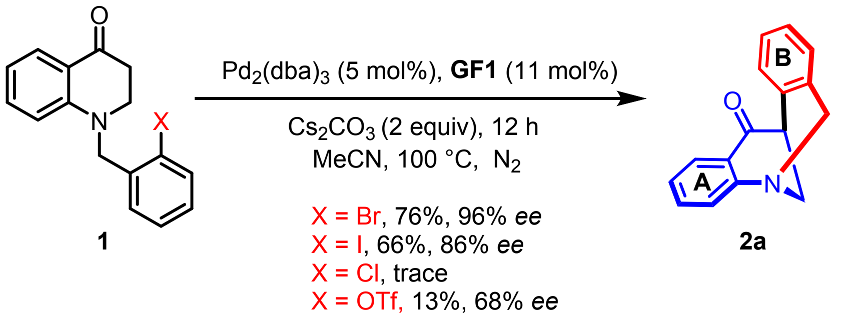

b) Competitive experiment

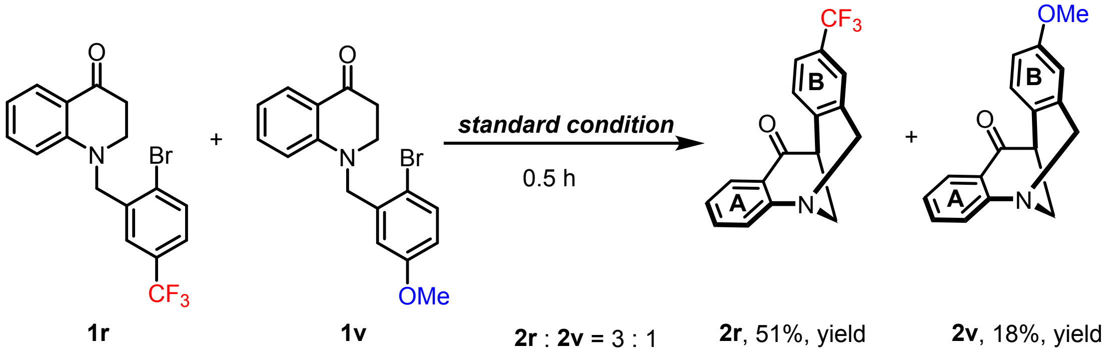

c) Linear relationship between *ee* of GF1 and *ee* of product 2a

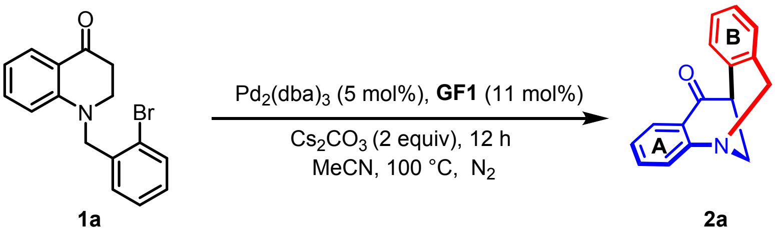

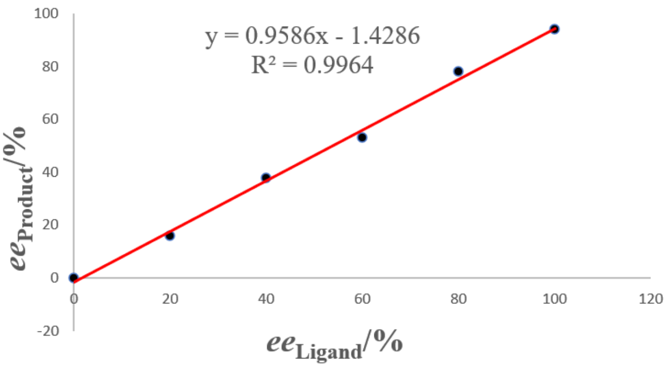

entry	1	2	3	4	5	6
ee of **GF1**	0	20%	40%	60%	80%	100%
ee of **2a**	0	16%	38%	53%	78%	94%

Step: Synthesis the chiral ligands **GF1** and *ent*-**GF1**, accurately weighing them according to the above ratio, then react for 12 hours under standard conditions. The mixture was concentrated in vacuum and the residue was purified by silica gel chromatography using PE: EA = 10: 1 as the eluent to afford the **2a** and detect the *ee* of **2a** by HPLC.

d) Destruction of hydrogen bonding

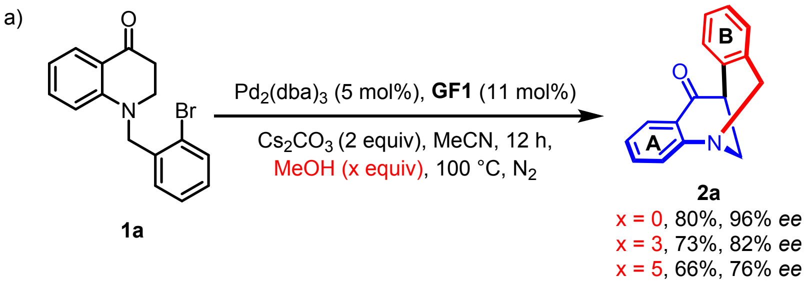

b)

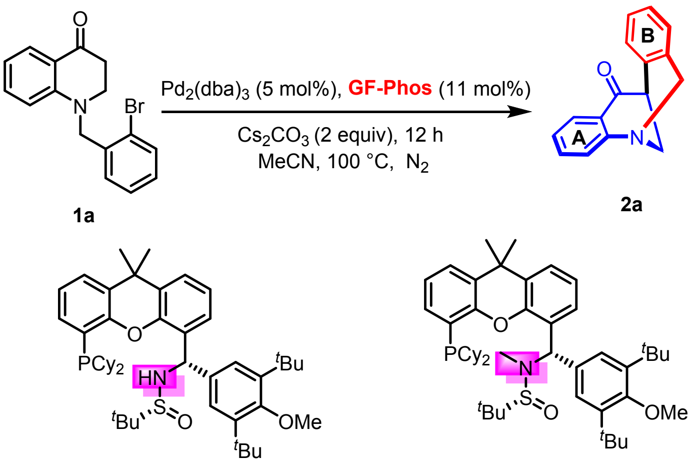

GF1, 76%, 96% *ee*

GF2, 54%, <5% *ee*

10. Compound characterization

1-(2-bromobenzyl)-2,3-dihydroquinolin-4(1*H*)-one (1a)

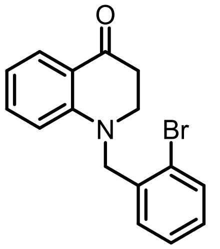

1a

Following the general procedure, flash column chromatography on a silica gel (petroleum ether: ethyl acetate =10: 1) give the product **1a** (1.475 g, 45% yield) as a yellow solid. m.p. 84.1-84.6 ^{o}C. ^{1}**H NMR** (400 MHz, $CDCl_3$) δ 7.96 (dd, *J* = 7.8, 1.4 Hz, 1H), 7.63 (d, *J* = 7.8 Hz, 1H), 7.35 – 7.26 (m, 3H), 7.21 – 7.16 (m, 1H), 6.81 – 6.71 (m, 1H), 6.53 (d, *J* = 8.5 Hz, 1H), 4.59 (s, 2H), 3.73 – 3.63 (m, 2H), 2.86 – 2.77 (m, 2H). ^{13}**C NMR** (100 MHz, $CDCl_3$) δ 193.2, 151.2, 135.6, 135.5, 133.0, 128.8, 128.1, 127.9, 127.6, 122.7, 119.7, 117.2, 113.3, 56.1, 49.6, 38.0. HRMS (EI): m/z: [M]+ Calcd for $C_{16}H_{14}BrNO$: 315.0253, found 315.0253.

1-(2-bromobenzyl)-5-chloro-2,3-dihydroquinolin-4(1*H*)-one (1b)

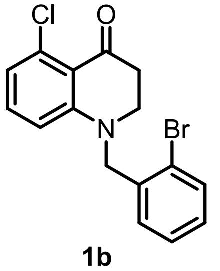

1b

Following the general procedure, flash column chromatography on a silica gel (petroleum ether: ethyl acetate =10: 1) give the product **1b** (0.628 g, 18% yield) as an orange solid. m.p. 85.1-85.9 ^{o}C. ^{1}**H NMR** (400 MHz, $CDCl_3$) δ 7.86 (d, *J* = 8.4 Hz, 1H), 7.62 (d, *J* = 7.8 Hz, 1H), 7.33 – 7.26 (m, 1H), 7.24 – 7.16 (m, 2H), 6.74 – 6.68 (m, 1H), 6.57 – 6.52 (m, 1H), 4.55 (s, 2H), 3.67 – 3.61 (m, 2H), 2.80 – 2.73 (m, 2H). ^{13}**C NMR** (100 MHz, $CDCl_3$) δ 192.1, 151.8, 141.9, 134.9, 133.2, 129.7, 129.1, 127.8, 127.7, 122.9, 118.2, 117.8, 112.9, 55.8, 49.3, 37.6. HRMS (EI): m/z: [M]+ Calcd for $C_{16}H_{13}BrClNO$: 348.9864, found 348.9865.

1-(2-bromobenzyl)-5-methyl-2,3-dihydroquinolin-4(1*H*)-one (1c)

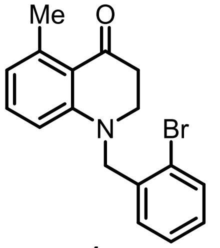

1c

Following the general procedure, flash column chromatography on a silica gel (petroleum ether: ethyl acetate =10: 1) give the product **1c** (0.954 g, 29% yield) as a yellowish solid. m.p. 94.8-95.4 ^{o}C. ^{1}**H NMR** (400 MHz, $CDCl_3$) δ 7.64 (d, *J* = 7.8 Hz, 1H), 7.30 – 7.26 (m, 2H), 7.22 – 7.12 (m,

2H), 6.56 (d, J = 7.3 Hz, 1H), 6.37 (d, J = 8.5 Hz, 1H), 4.59 (s, 2H), 3.72 – 3.62 (m, 2H), 2.86 – 2.78 (m, 2H), 2.67 (s, 3H). **^{13}C NMR** (100 MHz, $CDCl_3$) δ 194.9, 152.3, 142.7, 135.8, 134.1, 133.0, 128.8, 127.8, 127.6, 122.7, 120.9, 118.4, 111.4, 56.9, 49.5, 39.6, 23.7. HRMS (EI): m/z: [M]+ Calcd for $C_{17}H_{16}BrClNO$: 329.0410, found 329.0411.

1-(2-bromobenzyl)-6-(trifluoromethyl)-2,3-dihydroquinolin-4(1*H*)-one (1d)

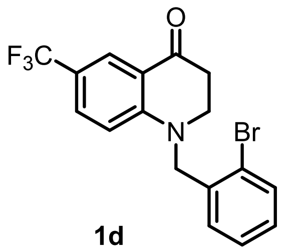

1d

Following the general procedure, flash column chromatography on a silica gel (petroleum ether: ethyl acetate =10: 1) give the product **1d** (0.23 g, 6% yield) as a yellowish solid. m.p. 93.3-94.0 °C. **^{1}H NMR** (400 MHz, $CDCl_3$) δ 8.24 – 8.16 (m, 1H), 7.67 – 7.60 (m, 1H), 7.51 – 7.46 (m, 1H), 7.31 – 7.27 (m, 1H), 7.22 – 7.17 (m, 2H), 6.60 (d, J = 8.9 Hz, 1H), 4.65 (s, 2H), 3.75 – 3.70 (m, 2H), 2.86 – 2.81 (m, 2H). **^{13}C NMR** (100 MHz, $CDCl_3$) δ 192.1, 152.7, 134.7, 133.4, 131.7 (d, J = 3.5 Hz), 129.3, 127.8, 127.6, 126.0 (d, J = 3.8 Hz), 122.9, 119.4, 119.1, 118.8, 113.7, 56.0, 49.3, 37.5. ^{19}F NMR (377 MHz, $CDCl_3$) δ -61.8. HRMS (EI): m/z: [M]+ Calcd for $C_{17}H_{13}BrF_3NO$: 383.0127, found 383.0132.

1-(2-bromobenzyl)-6-chloro-2,3-dihydroquinolin-4(1*H*)-one (1e)

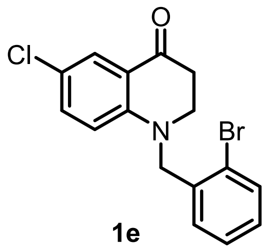

1e

Following the general procedure, flash column chromatography on a silica gel (petroleum ether: ethyl acetate =10: 1) give the product **1e** (0.83 g, 24% yield) as a yellow solid. m.p. 110.2-110.8 °C. **^{1}H NMR** (400 MHz, $CDCl_3$) δ 7.94 – 7.85 (m, 1H), 7.68 – 7.59 (m, 1H), 7.30 – 7.26 (m, 1H), 7.25 – 7.14 (m, 3H), 6.47 (d, J = 9.0 Hz, 1H), 4.57 (s, 2H), 3.71 – 3.63 (m, 2H), 2.83 – 2.76 (m, 2H). **^{13}C NMR** (100 MHz, $CDCl_3$) δ 192.1, 149.7, 135.3, 135.2, 133.3, 129.1, 127.8, 127.7, 127.5, 122.9, 122.8, 120.5, 115.1, 56.2, 49.6, 37.9. HRMS (EI): m/z: [M]+ Calcd for $C_{16}H_{13}BrClNO$: 348.9864, found 348.9862.

1-(2-bromobenzyl)-6-(*tert*-butyl)-2,3-dihydroquinolin-4(1*H*)-one (1f)

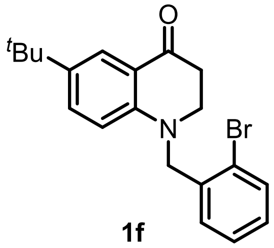

1f

Following the general procedure, flash column chromatography on a silica gel (petroleum ether: ethyl acetate =10: 1) give the product **1f** (1.0 g, 27% yield) as a yellow solid. m.p. 98.0-98.6 °C. **^{1}H NMR** (400 MHz, $CDCl_3$) δ 7.97 (s, 1H), 7.61 (d, *J* = 7.8 Hz, 1H), 7.38 (d, *J* = 8.7 Hz, 1H), 7.34 – 7.26 (m, 2H), 7.19 – 7.14 (m, 1H), 6.49 (d, *J* = 8.8 Hz, 1H), 4.54 (s, 2H), 3.63 (t, *J* = 6.9 Hz, 2H), 2.79 (t, *J* = 6.8 Hz, 2H), 1.28 (s, 9H). **^{13}C NMR** (100 MHz, $CDCl_3$) δ 193.6, 149.5, 140.1, 136.0, 133.3, 133.0, 128.8, 128.1, 127.7, 124.1, 122.8, 119.3, 113.4, 56.3, 49.9, 38.3, 33.9, 31.2. HRMS (EI): m/z: [M]+ Calcd for $C_{20}H_{22}BrNO$: 371.0879, found 371.0880.

1-(2-bromobenzyl)-6-methoxy-2,3-dihydroquinolin-4(1*H*)-one (1g)

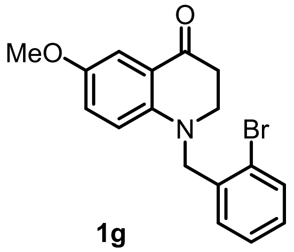

1g

Following the general procedure, flash column chromatography on a silica gel (petroleum ether: ethyl acetate =10: 1) give the product **1g** (1.76 g, 51% yield) as a yellow solid. m.p. 102.0-103.0 °C. **^{1}H NMR** (400 MHz, $CDCl_3$) δ 7.62 (d, *J* = 7.9 Hz, 1H), 7.49 – 7.43 (m, 1H), 7.36 – 7.26 (m, 2H), 7.21 – 7.14 (m, 1H), 7.01 – 6.96 (m, 1H), 6.50 (d, *J* = 9.1 Hz, 1H), 4.54 (s, 2H), 3.80 (s, 3H), 3.63 (t, *J* = 7.0 Hz, 2H), 2.81 (t, *J* = 7.0 Hz, 2H). **^{13}C NMR** (100 MHz, $CDCl_3$) δ 193.2, 151.5, 146.7, 136.0, 133.0, 128.8, 128.1, 127.6, 125.1, 122.7, 119.7, 115.2, 108.9, 56.4, 55.7, 50.1, 38.3. HRMS (EI): m/z: [M]+ Calcd for $C_{17}H_{16}BrNO_2$: 345.0359, found 345.0358.

1-(2-bromobenzyl)-6-(dimethylamino)-2,3-dihydroquinolin-4(1*H*)-one (1h)

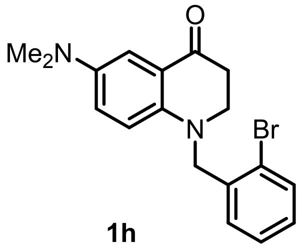

1h

Following the general procedure, flash column chromatography on a silica gel (petroleum ether: ethyl acetate =10: 1) give the product **1h** (0.3 g, 8% yield) as an orange solid. m.p. 98.3-99.0 °C. **^{1}H NMR** (400 MHz, $CDCl_3$) δ 7.60 (dd, *J* = 7.9, 1.0 Hz, 1H), 7.39 – 7.30 (m, 2H), 7.28 – 7.23 (m, 1H), 7.18 – 7.12 (m, 1H), 7.02 – 6.90 (m, 1H), 6.49 (d, *J* = 9.1 Hz, 1H), 4.50 (s, 2H), 3.66 – 3.51 (m, 2H), 2.85 (s, 6H), 2.82 – 2.76 (m, 2H). **^{13}C NMR** (100 MHz, $CDCl_3$) δ 193.8, 144.8, 143.4,

136.4, 133.0, 128.7, 128.3, 127.6, 123.7, 122.8, 120.2, 114.8, 111.2, 56.5, 50.2, 41.7, 38.7. HRMS (EI): m/z: [M]+ Calcd for $C_{18}H_{19}BrN_2O$: 358.0675, found 358.0677.

1-(2-bromobenzyl)-7-chloro-2,3-dihydroquinolin-4(1*H*)-one (1i)

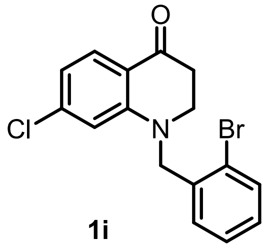

1i

Following the general procedure, flash column chromatography on a silica gel (petroleum ether: ethyl acetate =10: 1) give the product **1i** (0.698 g, 20% yield) as a yellow solid. m.p. 128.0-128.5 °C. **^{1}H NMR** (400 MHz, $CDCl_3$) δ 7.65 – 7.59 (m, 1H), 7.30 – 7.26 (m, 1H), 7.21 – 7.15 (m, 2H), 7.13 – 7.08 (m, 1H), 6.76 – 6.72 (m, 1H), 6.38 (d, J = 8.6 Hz, 1H), 4.58 (s, 2H), 3.71 – 3.66 (m, 2H), 2.86 – 2.81 (m, 2H). **^{13}C NMR** (100 MHz, $CDCl_3$) δ 191.6, 152.7, 135.3, 135.1, 134.2, 133.2, 129.0, 127.8, 127.5, 122.7, 120.5, 116.6, 112.1, 56.9, 49.3, 39.1. HRMS (EI): m/z: [M]+ Calcd for $C_{16}H_{13}BrClNO$: 348.9864, found 348.9865.

1-(2-bromobenzyl)-7-methyl-2,3-dihydroquinolin-4(1*H*)-one (1j)

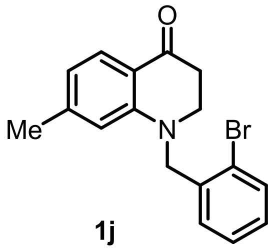

1j

Following the general procedure, flash column chromatography on a silica gel (petroleum ether: ethyl acetate =10: 1) give the product **1j** (0.625 g, 19% yield) as a yellow solid. m.p. 99.4-99.7 °C. **^{1}H NMR** (400 MHz, $CDCl_3$) δ 7.85 (d, J = 8.0 Hz, 1H), 7.62 (d, J = 7.8 Hz, 1H), 7.32 – 7.26 (m, 2H), 7.20 – 7.15 (m, 1H), 6.58 (d, J = 7.9 Hz, 1H), 6.34 (s, 1H), 4.57 (s, 2H), 3.64 – 3.59 (m, 2H), 2.77 – 2.72 (m, 2H), 2.24 (s, 3H). **^{13}C NMR** (100 MHz, $CDCl_3$) δ 192.9, 151.4, 146.6, 135.8, 133.0, 128.8, 128.2, 128.0, 127.7, 122.8, 118.8, 117.7, 113.2, 55.9, 49.6, 37.9, 22.3. HRMS (EI): m/z: [M]+ Calcd for $C_{17}H_{16}BrNO$: 329.0410, found 329.0410.

4-(2-bromobenzyl)-3,4-dihydrobenzo[*f*]quinolin-1(2*H*)-one (1k)

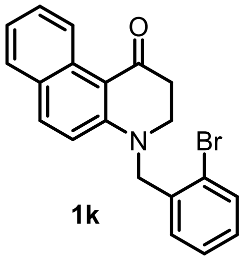

1k

Following the general procedure, flash column chromatography on a silica gel (petroleum ether:

ethyl acetate =10: 1) give the product **1k** (1.576 g, 43% yield) as a yellow solid. m.p. 119.3-120.0 °C. **^{1}H NMR** (400 MHz, $CDCl_3$) δ 9.52 (d, *J* = 8.7 Hz, 1H), 7.71 (d, *J* = 9.3 Hz, 1H), 7.67 – 7.53 (m, 3H), 7.32 – 7.16 (m, 4H), 6.75 (d, *J* = 9.3 Hz, 1H), 4.75 (s, 2H), 3.76 (t, *J* = 7.2 Hz, 2H), 2.89 (t, *J* = 7.2 Hz, 2H). **^{13}C NMR** (100 MHz, $CDCl_3$) δ 193.6, 152.8, 137.0, 135.4, 133.2, 132.8, 129.7, 129.0, 128.1, 127.8, 127.5, 126.9, 125.4, 123.3, 122.6, 114.9, 110.0, 56.8, 49.7, 39.1. HRMS (EI): m/z: [M]+ Calcd for $C_{20}H_{16}BrNO$: 365.0410, found 365.0412.

1-(2-bromobenzyl)-5,7-dimethyl-2,3-dihydroquinolin-4(1*H*)-one (1l)

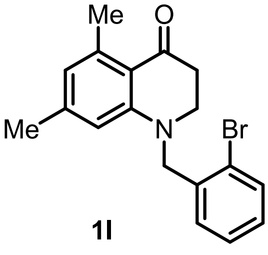

1l

Following the general procedure, flash column chromatography on a silica gel (petroleum ether: ethyl acetate =10: 1) give the product **1l** (0.5145 g, 15% yield) as a yellowish solid. m.p. 137.5-138.2 °C. **^{1}H NMR** (400 MHz, $CDCl_3$) δ 7.61 (d, *J* = 7.8 Hz, 1H), 7.28 – 7.25 (m, 2H), 7.20 – 7.13 (m, 1H), 6.38 (s, 1H), 6.18 (s, 1H), 4.56 (s, 2H), 3.63 – 3.58 (m, 2H), 2.77 – 2.72 (m, 2H), 2.61 (s, 3H), 2.17 (s, 3H). **^{13}C NMR** (100 MHz, $CDCl_3$) δ 194.4, 152.5, 144.9, 142.7, 136.0, 133.0, 128.7, 127.8, 127.6, 122.7, 122.4, 116.3, 111.4, 56.7, 49.4, 39.5, 23.6, 22.0. HRMS (EI): m/z: [M]+ Calcd for $C_{18}H_{18}BrNO$: 343.0566, found 343.0568.

1-(2-bromo-4-(trifluoromethyl)benzyl)-2,3-dihydroquinolin-4(1*H*)-one (1m)

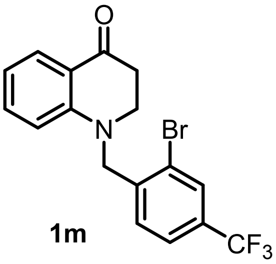

1m

Following the general procedure, flash column chromatography on a silica gel (petroleum ether: ethyl acetate =10: 1) give the product **1m** (0.68 g, 18% yield) as an orange solid. m.p. 100.2-100.8 °C. **^{1}H NMR** (400 MHz, $CDCl_3$) δ 8.02 – 7.92 (m, 1H), 7.88 (s, 1H), 7.53 (d, *J* = 8.1 Hz, 1H), 7.40 (d, *J* = 8.1 Hz, 1H), 7.34 – 7.28 (m, 1H), 6.84 – 6.74 (m, 1H), 6.43 (d, *J* = 8.5 Hz, 1H), 4.60 (s, 2H), 3.75 – 3.63 (m, 2H), 2.85 – 2.78 (m, 2H). **^{13}C NMR** (100 MHz, $CDCl_3$) δ 193.0, 150.9, 140.1, 135.6, 131.4, 131.1, 130.1 (d, *J* = 3.8 Hz), 128.4, 128.2, 124.6 (d, *J* = 3.6 Hz), 122.8, 120.1, 117.8, 113.2, 56.3, 50.1, 38.1. ^{19}F NMR (377 MHz, $CDCl_3$) δ -62.6. HRMS (EI): m/z: [M]+ Calcd for $C_{17}H_{13}BrF_3NO$: 383.0127, found 383.0132.

1-(2-bromo-4-fluorobenzyl)-2,3-dihydroquinolin-4(1*H*)-one (1n)

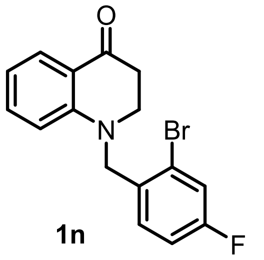
1n

Following the general procedure, flash column chromatography on a silica gel (petroleum ether: ethyl acetate =10: 1) give the product **1n** (0.91 g, 27% yield) as a yellow solid. m.p. 135.5-136.2 °C. **^{1}H NMR** (400 MHz, $CDCl_3$) δ 8.01 – 7.90 (m, 1H), 7.43 – 7.35 (m, 1H), 7.34 – 7.29 (m, 1H), 7.27 – 7.23 (m, 1H), 7.02 – 6.96 (m, 1H), 6.79 – 6.74 (m, 1H), 6.49 (d, *J* = 8.5 Hz, 1H), 4.53 (s, 2H), 3.67 – 3.62 (m, 2H), 2.82 – 2.77 (m, 2H). **^{13}C NMR** (100 MHz, $CDCl_3$) δ 193.2, 161.6 (d, *J* = 248.9 Hz), 151.1, 135.6, 131.6 (d, *J* = 3.4 Hz), 128.9 (d, *J* = 8.2 Hz), 128.3, 122.6 (d, *J* = 9.5 Hz), 120.4 (d, *J* = 24.5 Hz), 120.0, 117.5, 114.8 (d, *J* = 20.8 Hz), 113.3, 55.6, 49.8, 38.1. ^{19}F NMR (377 MHz, $CDCl_3$) δ -113.1. HRMS (EI): m/z: [M]+ Calcd for $C_{16}H_{13}BrFNO$: 333.0159, found 333.016.

1-(2-bromo-4-chlorobenzyl)-2,3-dihydroquinolin-4(1*H*)-one (1o)

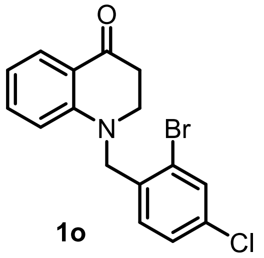
1o

Following the general procedure, flash column chromatography on a silica gel (petroleum ether: ethyl acetate =10: 1) give the product **1o** (0.888 g, 25% yield) as a yellowish solid. m.p. 147.9-148.1 °C. **^{1}H NMR** (400 MHz, $CDCl_3$) δ 7.97 – 7.93 (m, 1H), 7.68 – 7.56 (m, 1H), 7.33 – 7.28 (m, 1H), 7.26 – 7.18 (m, 2H), 6.79 – 6.74 (m, 1H), 6.46 (d, *J* = 8.5 Hz, 1H), 4.52 (s, 2H), 3.67 – 3.63 (m, 2H), 2.82 – 2.77 (m, 2H). **^{13}C NMR** (100 MHz, $CDCl_3$) δ 193.1, 151.0, 135.6, 134.4, 133.8, 132.7, 128.8, 128.3, 127.9, 122.9, 120.0, 117.5, 113.3, 55.8, 49.8, 38.1. HRMS (EI): m/z: [M]+ Calcd for $C_{16}H_{13}BrClNO$: 348.9864, found 348.9867.

1-(2-bromo-4-methylbenzyl)-2,3-dihydroquinolin-4(1*H*)-one (1p)

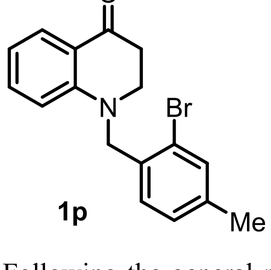
1p

Following the general procedure, flash column chromatography on a silica gel (petroleum ether: ethyl acetate =10: 1) give the product **1p** (0.5 g, 15% yield) as a yellow solid. m.p. 57.5-58.3 °C. **^{1}H NMR** (400 MHz, $CDCl_3$) δ 8.00 – 7.87 (m, 1H), 7.45 (s, 1H), 7.33 – 7.27 (m, 1H), 7.14 (d, *J* =

7.8 Hz, 1H), 7.06 (d, J = 7.8 Hz, 1H), 6.77 – 6.71 (m, 1H), 6.53 (d, J = 8.5 Hz, 1H), 4.54 (s, 2H), 3.67 – 3.61 (m, 2H), 2.81 – 2.75 (m, 2H), 2.33 (s, 3H). **^{13}C NMR** (100 MHz, $CDCl_3$) δ 193.3, 151.3, 139.1, 135.5, 133.5, 132.5, 128.4, 128.2, 127.8, 122.6, 119.8, 117.1, 113.4, 55.7, 49.6, 38.0, 20.6. HRMS (EI): m/z: [M]+ Calcd for $C_{17}H_{16}BrNO$: 329.0410, found 329.0407.

1-(2-bromo-4-methoxybenzyl)-2,3-dihydroquinolin-4(1*H*)-one (1q)

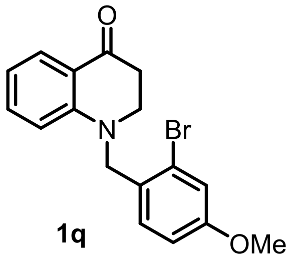

Following the general procedure, flash column chromatography on a silica gel (petroleum ether: ethyl acetate =10: 1) give the product **1q** (2.0 g, 58% yield) as a yellow solid. m.p. 93.0-93.3 °C. **^{1}H NMR** (400 MHz, $CDCl_3$) δ 7.99 – 7.90 (m, 1H), 7.35 – 7.28 (m, 1H), 7.21 – 7.13 (m, 2H), 6.85 – 6.79 (m, 1H), 6.77 – 6.69 (m, 1H), 6.56 (d, J = 8.5 Hz, 1H), 4.51 (s, 2H), 3.79 (s, 3H), 3.66 – 3.58 (m, 2H), 2.81 – 2.74 (m, 2H). **^{13}C NMR** (100 MHz, $CDCl_3$) δ 193.4, 159.4, 151.4, 135.5, 128.7, 128.2, 127.4, 123.1, 119.8, 118.5, 117.1, 113.5, 113.4, 55.5, 55.3, 49.4, 38.1. HRMS (EI): m/z: [M]+ Calcd for $C_{17}H_{16}BrNO_2$: 345.0359, found 345.0357.

1-(2-bromo-5-(trifluoromethyl)benzyl)-2,3-dihydroquinolin-4(1*H*)-one (1r)

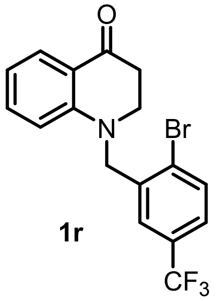

Following the general procedure, flash column chromatography on a silica gel (petroleum ether: ethyl acetate =10: 1) give the product **1r** (1.034 g, 27% yield) as a yellowish solid. m.p. 83.6-84.2 °C. **^{1}H NMR** (400 MHz, $CDCl_3$) δ 8.00 – 7.94 (m, 1H), 7.75 (d, J = 8.2 Hz, 1H), 7.54 (s, 1H), 7.44 (d, J = 8.2 Hz, 1H), 7.36 – 7.30 (m, 1H), 6.82 – 6.76 (m, 1H), 6.47 (d, J = 8.5 Hz, 1H), 4.58 (s, 2H), 3.69 – 3.63 (m, 2H), 2.84 – 2.79 (m, 2H). **^{13}C NMR** (100 MHz, $CDCl_3$) δ 193.0, 151.1, 137.4, 135.6, 133.7, 130.5, 130.2, 128.3, 126.6, 125.7 (d, J = 3.6 Hz), 124.6 (d, J = 3.7 Hz), 120.2, 117.9, 113.2, 56.4, 50.0, 38.1. ^{19}F NMR (377 MHz, $CDCl_3$) δ -62.7. HRMS (EI): m/z: [M]+ Calcd for $C_{17}H_{13}BrF_3NO$: 383.0127, found 383.0127.

1-(2-bromo-5-fluorobenzyl)-2,3-dihydroquinolin-4(1*H*)-one (1s)

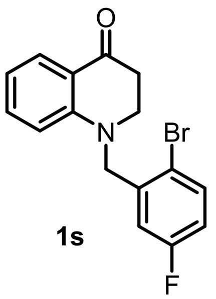

1s

Following the general procedure, flash column chromatography on a silica gel (petroleum ether: ethyl acetate =10: 1) give the product **1s** (0.495 g, 15% yield) as a yellow solid. m.p. 95.6-95.9 ^{o}C. ^{1}**H NMR** (400 MHz, $CDCl_3$) δ 7.96 (d, *J* = 7.6 Hz, 1H), 7.62 – 7.53 (m, 1H), 7.35 – 7.28 (m, 1H), 7.03 (d, *J* = 9.0 Hz, 1H), 6.96 – 6.87 (m, 1H), 6.82 – 6.75 (m, 1H), 6.45 (d, *J* = 8.5 Hz, 1H), 4.52 (s, 2H), 3.67 (t, *J* = 6.9 Hz, 2H), 2.82 (t, *J* = 6.9 Hz, 2H). ^{13}**C NMR** (100 MHz, $CDCl_3$) δ 193.1, 162.4 (d, *J* = 246.2 Hz), 151.0, 138.4, 135.6, 134.3 (d, *J* = 7.8 Hz), 128.3, 120.1, 117.7, 116.4 (d, *J* = 3.6 Hz), 116.0 (d, *J* = 22.5 Hz), 115.2 (d, *J* = 24.1 Hz), 113.3, 56.4, 50.0, 38.1. ^{19}F NMR (377 MHz, $CDCl_3$) δ -113.4. HRMS (EI): m/z: [M]+ Calcd for $C_{16}H_{13}BrFNO$: 333.0159, found 333.0158.

1-(2-bromo-5-chlorobenzyl)-2,3-dihydroquinolin-4(1*H*)-one (1t)

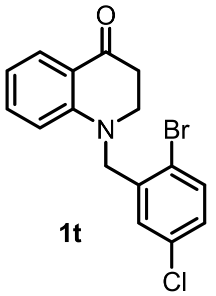

1t

Following the general procedure, flash column chromatography on a silica gel (petroleum ether: ethyl acetate =10: 1) give the product **1t** (0.65 g, 19% yield) as a yellow solid. m.p. 106.8-107.3 ^{o}C. ^{1}**H NMR** (400 MHz, $CDCl_3$) δ 7.96 (dd, *J* = 7.9, 1.6 Hz, 1H), 7.54 (d, *J* = 8.4 Hz, 1H), 7.35 – 7.30 (m, 1H), 7.29 – 7.26 (m, 1H), 7.16 (dd, *J* = 8.4, 2.5 Hz, 1H), 6.82 – 6.75 (m, 1H), 6.46 (d, *J* = 8.5 Hz, 1H), 4.51 (s, 2H), 3.69 – 3.63 (m, 2H), 2.85 – 2.80 (m, 2H). ^{13}**C NMR** (100 MHz, $CDCl_3$) δ 193.1, 151.1, 137.9, 135.6, 134.1, 129.0, 128.3, 127.9, 120.4, 120.1, 117.7, 113.3, 56.4, 50.0, 38.1. HRMS (EI): m/z: [M]+ Calcd for $C_{16}H_{13}BrClNO$: 348.9864, found 348.9861.

1-(2-bromo-5-methylbenzyl)-2,3-dihydroquinolin-4(1*H*)-one (1u)

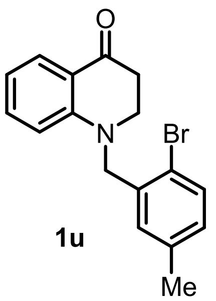

1u

Following the general procedure, flash column chromatography on a silica gel (petroleum ether: ethyl acetate =10: 1) give the product **1u** (0.71 g, 22% yield) as a yellow solid. m.p. 95.3-96.3 °C. **^{1}H NMR** (400 MHz, $CDCl_3$) δ 8.02 – 7.90 (m, 1H), 7.48 (d, *J* = 8.1 Hz, 1H), 7.34 – 7.28 (m, 1H), 7.08 (s, 1H), 6.98 (d, *J* = 8.0 Hz, 1H), 6.78 – 6.71 (m, 1H), 6.54 (d, *J* = 8.5 Hz, 1H), 4.53 (s, 2H), 3.67 – 3.60 (m, 2H), 2.82 – 2.76 (m, 2H), 2.25 (s, 3H). **^{13}C NMR** (100 MHz, $CDCl_3$) δ 193.3, 151.4, 137.7, 135.5, 135.3, 132.8, 129.7, 128.6, 128.1, 119.8, 119.4, 117.1, 113.4, 56.1, 49.6, 38.0, 21.1. HRMS (EI): m/z: [M]+ Calcd for $C_{17}H_{16}BrNO$: 329.0410, found 329.0406.

1-(2-bromo-5-methoxybenzyl)-2,3-dihydroquinolin-4(1*H*)-one (1v)

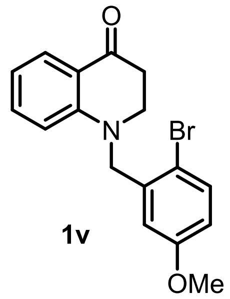

1v

Following the general procedure, flash column chromatography on a silica gel (petroleum ether: ethyl acetate =10: 1) give the product **1v** (1.08 g, 31% yield) as a yellow solid. m.p. 67.3-68.3 °C. **^{1}H NMR** (400 MHz, $CDCl_3$) δ 7.94 (d, *J* = 7.8 Hz, 1H), 7.50 (d, *J* = 8.7 Hz, 1H), 7.34 – 7.28 (m, 1H), 6.87 – 6.82 (m, 1H), 6.77 – 6.70 (m, 2H), 6.52 (d, *J* = 8.5 Hz, 1H), 4.52 (s, 2H), 3.70 (s, 3H), 3.65 (t, *J* = 7.0 Hz, 2H), 2.79 (t, *J* = 7.0 Hz, 2H). **^{13}C NMR** (100 MHz, $CDCl_3$) δ 193.3, 159.3, 151.3, 136.8, 135.6, 133.7, 128.2, 119.8, 117.3, 114.3, 113.7, 113.4, 112.9, 56.2, 55.4, 49.7, 38.1. HRMS (EI): m/z: [M]+ Calcd for $C_{17}H_{16}BrNO_2$: 345.0359, found 345.0357.

1-((6-bromobenzo[*d*][1,3]dioxol-5-yl)methyl)-2,3-dihydroquinolin-4(1*H*)-one (1w)

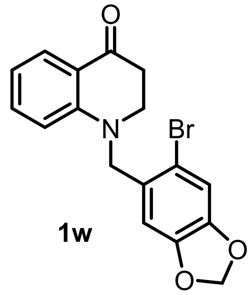

1w

Following the general procedure, flash column chromatography on a silica gel (petroleum ether: ethyl acetate =10: 1) give the product **1w** (0.73 g, 20% yield) as a yellow solid. m.p. 173.6-174.1 °C. **^{1}H NMR** (400 MHz, $CDCl_3$) δ 7.93 (dd, *J* = 7.9, 1.6 Hz, 1H), 7.35 – 7.29 (m, 1H), 7.06 (s, 1H), 6.78 (s, 1H), 6.77 – 6.73 (m, 1H), 6.53 (d, *J* = 8.5 Hz, 1H), 5.95 (s, 2H), 4.46 (s, 2H), 3.65 – 3.60 (m, 2H), 2.81 – 2.75 (m, 2H). **^{13}C NMR** (100 MHz, $CDCl_3$) δ 193.3, 151.3, 147.9, 147.7, 135.6, 129.1, 128.3, 119.9, 117.4, 113.4, 113.1, 112.9, 108.0, 101.9, 56.1, 49.7, 38.1. HRMS (EI): m/z: [M]+ Calcd for $C_{17}H_{14}BrNO_3$: 359.0152, found 359.0150.

1-(2-bromo-4,5-dimethoxybenzyl)-2,3-dihydroquinolin-4(1*H*)-one (1x)

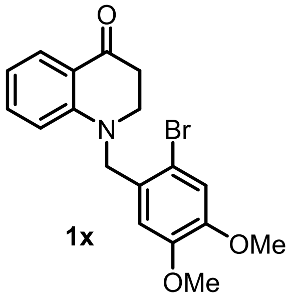

1x

Following the general procedure, flash column chromatography on a silica gel (petroleum ether: ethyl acetate =10: 1) give the product **1x** (1.6 g, 43% yield) as a yellow solid. m.p. 176.1-176.9 ^{o}C. **^{1}H NMR** (400 MHz, $CDCl_3$) δ 7.94 (dd, *J* = 7.8, 1.1 Hz, 1H), 7.37 – 7.29 (m, 1H), 7.07 (s, 1H), 6.78 (s, 1H), 6.77 – 6.70 (m, 1H), 6.61 (d, *J* = 8.5 Hz, 1H), 4.49 (s, 2H), 3.87 (s, 3H), 3.70 (s, 3H), 3.63 – 3.56 (m, 2H), 2.81 – 2.72 (m, 2H). **^{13}C NMR** (100 MHz, $CDCl_3$) δ 193.3, 151.5, 148.9, 148.8, 135.5, 128.2, 127.7, 119.9, 117.4, 115.9, 113.5, 112.8, 111.0, 56.2, 56.0, 55.5, 49.4, 38.1. HRMS (EI): m/z: [M]+ Calcd for $C_{18}H_{18}BrNO_3$: 375.0465, found 375.0466.

1-(2-bromo-4-chlorobenzyl)-6-methoxy-2,3-dihydroquinolin-4(1*H*)-one (1y)

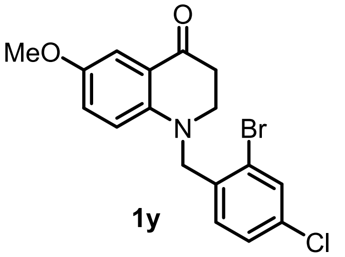

1y

Following the general procedure, flash column chromatography on a silica gel (petroleum ether: ethyl acetate =10: 1) give the product **1y** (1.5 g, 40% yield) as a yellow solid. m.p. 101.8-102.0 ^{o}C. **^{1}H NMR** (400 MHz, $CDCl_3$) δ 7.65 – 7.60 (m, 1H), 7.46 – 7.43 (m, 1H), 7.25 – 7.22 (m, 2H), 6.99 – 6.95 (m, 1H), 6.43 (d, *J* = 9.1 Hz, 1H), 4.47 (s, 2H), 3.78 (s, 3H), 3.62 – 3.57 (m, 2H), 2.81 – 2.77 (m, 2H). **^{13}C NMR** (100 MHz, $CDCl_3$) δ 193.1, 151.8, 146.4, 134.8, 133.8, 132.7, 129.0, 127.9, 125.0, 122.9, 120.1, 115.1, 109.2, 56.2, 55.7, 50.3, 38.4. HRMS (EI): m/z: [M]+ Calcd for $C_{17}H_{15}BrClNO_2$: 378.9969, found 378.9970.

1-(2-bromo-5-fluorobenzyl)-6-methoxy-2,3-dihydroquinolin-4(1*H*)-one (1z)

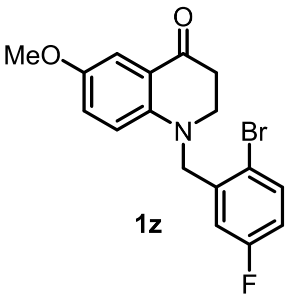

1z

Following the general procedure, flash column chromatography on a silica gel (petroleum ether: ethyl acetate =10: 1) give the product **1z** (1.9 g, 52% yield) as a yellow solid. m.p. 124.0-124.7 ^{o}C. **^{1}H NMR** (400 MHz, $CDCl_3$) δ 7.61 – 7.51 (m, 1H), 7.48 – 7.39 (m, 1H), 7.10 – 7.02 (m, 1H),

7.00 – 6.94 (m, 1H), 6.93 – 6.85 (m, 1H), 6.41 (d, *J* = 9.1 Hz, 1H), 4.46 (s, 2H), 3.78 (s, 3H), 3.65 – 3.58 (m, 2H), 2.84 – 2.77 (m, 2H). **^{13}C NMR** (100 MHz, $CDCl_3$) δ 193.1, 162.4 (d, *J* = 246.1 Hz), 151.9, 146.4, 138.7 (d, *J* = 6.9 Hz), 134.2 (d, *J* = 7.7 Hz), 125.0, 120.1, 116.3 (d, *J* = 3.2 Hz), 116.0 (d, *J* = 22.6 Hz), 115.3 (d, *J* = 24.1 Hz), 115.1, 109.2, 56.7, 55.7, 50.4, 38.4. ^{19}F NMR (377 MHz, $CDCl_3$) δ -113.5. HRMS (EI): m/z: [M]+ Calcd for $C_{17}H_{15}BrFNO_2$: 363.0265, found 363.0265.

1-(2-bromo-5-methoxybenzyl)-6-methoxy-2,3-dihydroquinolin-4(1*H*)-one (1aa)

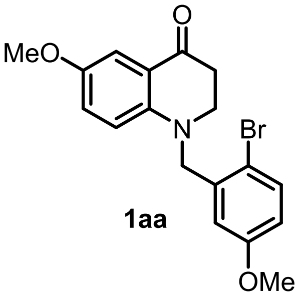

1aa

Following the general procedure, flash column chromatography on a silica gel (petroleum ether: ethyl acetate =10: 1) give the product **1aa** (1.7 g, 45% yield) as a yellow solid. m.p. 96.7-97.3 °C. **^{1}H NMR** (400 MHz, $CDCl_3$) δ 7.48 (d, *J* = 8.7 Hz, 1H), 7.45 – 7.39 (m, 1H), 7.00 – 6.92 (m, 1H), 6.89 – 6.83 (m, 1H), 6.74 – 6.67 (m, 1H), 6.48 (d, *J* = 9.2 Hz, 1H), 4.46 (s, 2H), 3.77 (s, 3H), 3.70 (s, 3H), 3.62 – 3.56 (m, 2H), 2.83 – 2.74 (m, 2H). **^{13}C NMR** (100 MHz, $CDCl_3$) δ 193.2, 159.3, 151.6, 146.7, 137.2, 133.6, 125.1, 119.8, 115.2, 114.4, 113.7, 112.8, 109.0, 56.5, 55.7, 55.4, 50.1, 38.3. HRMS (EI): m/z: [M]+ Calcd for $C_{18}H_{18}BrNO_3$: 375.0465, found 375.0468.

4-((6-bromobenzo[*d*][1,3]dioxol-5-yl)methyl)-3,4-dihydrobenzo[*f*]quinolin-1(2*H*)-one (1ab)

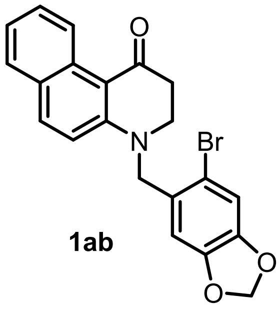

1ab

Following the general procedure, flash column chromatography on a silica gel (petroleum ether: ethyl acetate =10: 1) give the product **1ab** (1.56 g, 38% yield) as a yellow solid. m.p. 178.3-179.0 °C. **^{1}H NMR** (400 MHz, $CDCl_3$) δ 9.50 (d, *J* = 8.7 Hz, 1H), 7.73 (d, *J* = 9.3 Hz, 1H), 7.62 (d, *J* = 8.0 Hz, 1H), 7.60 – 7.55 (m, 1H), 7.33 – 7.27 (m, 1H), 7.09 (s, 1H), 6.77 (d, *J* = 9.3 Hz, 1H), 6.72 (s, 1H), 5.95 (s, 2H), 4.63 (s, 2H), 3.78 – 3.67 (m, 2H), 2.90 – 2.81 (m, 2H). **^{13}C NMR** (100 MHz, $CDCl_3$) δ 193.7, 152.8, 148.0, 147.8, 137.1, 132.8, 129.7, 128.8, 128.2, 127.0, 125.4, 123.4, 114.8, 113.2, 112.7, 110.2, 107.5, 101.9, 56.7, 49.7, 39.1. HRMS (EI): m/z: [M]+ Calcd for $C_{21}H_{16}BrNO_3$: 409.0308, found 409.0306.

1-((6-bromobenzo[*d*][1,3]dioxol-5-yl)methyl)-5,7-dimethyl-2,3-dihydroquinolin-4(1*H*)-one (1ac)

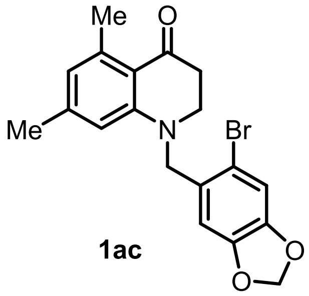

1ac

Following the general procedure, flash column chromatography on a silica gel (petroleum ether: ethyl acetate =10: 1) give the product **1ac** (1.0 g, 26% yield) as a yellow solid. m.p. 123.0-123.4 °C. **^{1}H NMR** (400 MHz, $CDCl_3$) δ 7.05 (s, 1H), 6.77 (s, 1H), 6.38 (s, 1H), 6.18 (s, 1H), 5.95 (s, 2H), 4.43 (s, 2H), 3.60 – 3.52 (m, 2H), 2.76 – 2.69 (m, 2H), 2.60 (s, 3H), 2.19 (s, 3H). **^{13}C NMR** (100 MHz, $CDCl_3$) δ 194.3, 152.4, 147.8, 147.5, 144.9, 142.7, 129.4, 122.5, 116.4, 113.0, 112.7, 111.4, 107.9, 101.7, 56.6, 49.4, 39.5, 23.6, 22.1. HRMS (EI): m/z: [M]+ Calcd for $C_{19}H_{18}BrNO_3$: 387.0465, found 387.0466.

1-(2-iodobenzyl)-2,3-dihydroquinolin-4(1*H*)-one (1ad)

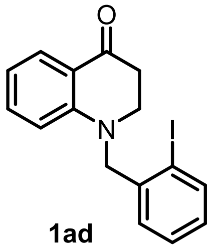

1ad

Following the general procedure, flash column chromatography on a silica gel (petroleum ether: ethyl acetate =10: 1) give the product **1ad** (0.65 g, 18% yield) as a yellow solid. m.p. 85.8-86.3 °C. **^{1}H NMR** (400 MHz, $CDCl_3$) δ 7.95 (dd, *J* = 7.9, 1.7 Hz, 1H), 7.90 (dd, *J* = 7.9, 1.0 Hz, 1H), 7.32 – 7.27 (m, 2H), 7.23 (dd, *J* = 7.6, 1.4 Hz, 1H), 7.03 – 6.98 (m, 1H), 6.77 – 6.71 (m, 1H), 6.49 (d, *J* = 8.5 Hz, 1H), 4.47 (s, 2H), 3.67 – 3.61 (m, 2H), 2.81 – 2.76 (m, 2H). **^{13}C NMR** (100 MHz, $CDCl_3$) δ 193.2, 151.1, 139.7, 138.2, 135.5, 129.1, 128.5, 128.1, 127.6, 119.8, 117.2, 113.4, 97.7, 61.0, 49.6, 38.0. HRMS (EI): m/z: [M]+ Calcd for $C_{16}H_{14}INO$: 363.0115, found 363.0114.

1-(2-chlorobenzyl)-2,3-dihydroquinolin-4(1*H*)-one (1ae)

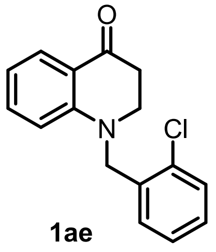

1ae

Following the general procedure, flash column chromatography on a silica gel (petroleum ether: ethyl acetate =10: 1) give the product **1ae** (0.65 g, 24% yield) as a yellow solid. m.p. 93.8-94.2 °C. **^{1}H NMR** (400 MHz, $CDCl_3$) δ 7.94 (d, *J* = 7.8 Hz, 1H), 7.42 (d, *J* = 7.8 Hz, 1H), 7.32 – 7.26 (m, 2H), 7.25 – 7.17 (m, 2H), 6.78 – 6.69 (m, 1H), 6.53 (d, *J* = 8.5 Hz, 1H), 4.61 (s, 2H), 3.65 (t, *J* =

7.0 Hz, 2H), 2.79 (t, J = 7.0 Hz, 2H). **^{13}C NMR** (100 MHz, $CDCl_3$) δ 193.3, 151.3, 135.5, 134.2, 132.9, 129.8, 128.6, 128.2, 127.8, 127.0, 119.8, 117.2, 113.3, 53.5, 49.7, 38.0. HRMS (EI): m/z: [M]+ Calcd for $C_{16}H_{14}ClNO$: 271.0758, found 271.0759.

2-((4-oxo-3,4-dihydroquinolin-1(2*H*)-yl)methyl)phenyl trifluoromethanesulfonate (1af)

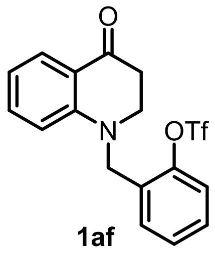

Following the general procedure, flash column chromatography on a silica gel (petroleum ether: ethyl acetate =10: 1) give the product **1af** (1.04 g, 27% yield) as a yellow solid. m.p. 93.2-93.9 °C. **^{1}H NMR** (400 MHz, $CDCl_3$) δ 7.94 (dd, J = 7.8, 1.4 Hz, 1H), 7.44 – 7.28 (m, 5H), 6.80 – 6.71 (m, 1H), 6.56 (d, J = 8.5 Hz, 1H), 4.67 (s, 2H), 3.71 – 3.53 (m, 2H), 2.83 – 2.71 (m, 2H). **^{13}C NMR** (100 MHz, $CDCl_3$) δ 193.0, 151.0, 147.1, 135.5, 130.2, 129.2, 128.7, 128.2, 121.7, 120.1, 120.0, 117.5, 116.9, 113.1, 50.2, 49.7, 37.9. **^{19}F NMR** (377 MHz, $CDCl_3$) δ -73.5. HRMS (EI): m/z: [M]+ Calcd for $C_{17}H_{14}F_3NO_4S$: 385.0590, found 385.0591.

(*R*)-*N*-((*S*)-(3,5-di-*tert*-butyl-4-methoxyphenyl)(5-(dicyclohexylphosphanyl)-9,9-dimethyl-9*H*-xanthen-4-yl)methyl)-*N*,2-dimethylpropane-2-sulfinamide (GF2)

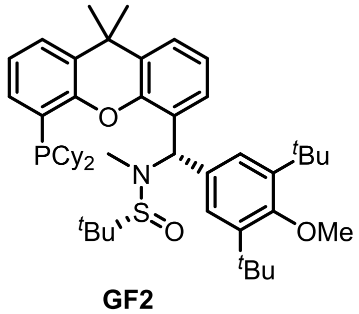

Following the general procedure, flash column chromatography on a silica gel (Petroleum ether: Acetone = 10:1) give the product **GF2** (616.8 mg, 80% yield) as a white solid. m.p. 188.9-189.6 °C. **^{1}H NMR** (400 MHz, $CDCl_3$) δ 7.38 (d, J = 7.5 Hz, 3H), 7.22 (d, J = 7.1 Hz, 1H), 7.15 (s, 2H), 7.14 – 6.96 (m, 2H), 6.81 (s, 1H), 3.64 (s, 3H), 2.57 (s, 3H), 2.03 – 1.77 (m, 3H), 1.73 – 1.65 (m, 3H), 1.62 (s, 3H), 1.56 (s, 3H), 1.51 – 1.39 (m, 5H), 1.35 (s, 19H), 1.33 – 1.28 (m, 3H), 1.24 – 1.15 (m, 2H), 1.14 – 1.09 (m, 9H), 1.09 – 0.76 (m, 6H). **^{13}C NMR** (100 MHz, $CDCl_3$) δ 158.3, 155.1, 149.4, 142.6, 133.1, 131.4, 131.3, 131.2, 131.0, 128.4, 127.7, 127.2, 125.2, 123.8, 123.4 (d, J = 24.9 Hz), 122.5 (d, J = 7.6 Hz), 65.5 (d, J = 5.2 Hz), 64.1, 58.7, 35.7, 34.9, 33.4 (d, J = 16.8 Hz), 32.2, 31.8 (d, J = 14.5 Hz), 30.6, 30.0, 29.6 (d, J = 11.9 Hz), 28.0, 26.4 (d, J = 25.7 Hz), 23.9. **^{31}P NMR** (162 MHz, $CDCl_3$) δ -20.1. HRMS (ESI): m/z: Calcd for $(C_{48}H_{70}NO_3PS+Na)^+$ 794.4706, found 794.4724.

(5*S*,11*S*)-6,11-dihydro-12*H*-5,11-methanodibenzo[*b*,*f*]azocin-12-one (2a)

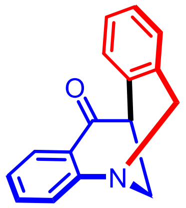

2a

Following the general procedure, preparative thin layer chromatography on a silica gel (petroleum ether: ethyl acetate =4: 1) to give the product **2a** (53.5 mg, 76% yield) as a yellow solid. m.p. 97.2-98.1 °C. **^{1}H NMR** (400 MHz, $CDCl_3$) δ 7.86 (d, *J* = 7.7 Hz, 1H), 7.49 – 7.41 (m, 1H), 7.30 (d, *J* = 7.3 Hz, 1H), 7.23 – 7.12 (m, 3H), 7.10 – 7.02 (m, 1H), 6.94 (d, *J* = 7.2 Hz, 1H), 4.80 (d, *J* = 16.9 Hz, 1H), 4.29 (d, *J* = 17.0 Hz, 1H), 4.04 (d, *J* = 13.5 Hz, 1H), 3.69 – 3.64 (m, 1H), 3.48 (s, 1H). **^{13}C NMR** (100 MHz, $CDCl_3$) δ 195.2, 154.3, 134.9, 132.5, 131.9, 129.4, 128.4, 127.5, 127.1, 126.8, 125.2, 125.0, 124.1, 57.5, 50.3, 45.0. **HPLC conditions**: Daicel Chiralpak OD-H column (hexane/ isopropanol = 90/ 10, flow rate 1.0 mL/min, 254 nm); t_R = 9.362 (major), t_R = 10.146 (minor), 96% *ee*. HRMS (ESI): m/z: Calcd for $(C_{16}H_{13}NO+H)^+$ 236.1070, found 236.1073. $[\alpha]_D^{20}$ = +390.044 (c = 1.038, $CHCl_3$).

(5*S*,11*S*)-1-chloro-6,11-dihydro-12*H*-5,11-methanodibenzo[*b*,*f*]azocin-12-one (2b)

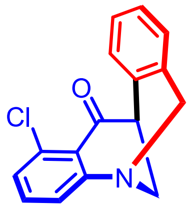

2b

Following the general procedure, preparative thin layer chromatography on a silica gel (petroleum ether: ethyl acetate =4: 1) to give the product **2b** (33.5 mg, 42% yield) as a white solid. m.p. 101.7-102.6 °C. **^{1}H NMR** (400 MHz, $CDCl_3$) δ 7.79 (d, *J* = 8.4 Hz, 1H), 7.28 (d, *J* = 7.5 Hz, 1H), 7.25 – 7.15 (m, 3H), 7.04 (d, *J* = 8.4 Hz, 1H), 6.96 (d, *J* = 7.3 Hz, 1H), 4.79 (d, *J* = 17.0 Hz, 1H), 4.27 (d, *J* = 17.0 Hz, 1H), 4.02 (d, *J* = 13.5 Hz, 1H), 3.67 (d, *J* = 13.7 Hz, 1H), 3.48 (s, 1H). **^{13}C NMR** (100 MHz, $CDCl_3$) δ 194.2, 155.4, 140.8, 132.3, 131.6, 129.5, 128.9, 128.6, 127.4, 126.9, 125.1, 124.8, 123.8, 57.6, 50.3, 44.8. **HPLC conditions**: Daicel Chiralpak OD-H column (hexane/ isopropanol = 90/ 10, flow rate 1.0 mL/min, 254 nm); t_R = 8.634 (major), t_R = 10.453 (minor), 96% *ee*. HRMS (ESI): m/z: Calcd for $(C_{16}H_{12}ClNO+H)^+$ 270.0680, found 270.0681. $[\alpha]_D^{20}$ = +310.535 (c = 0.578, $CHCl_3$).

(5*S*,11*S*)-1-methyl-6,11-dihydro-12*H*-5,11-methanodibenzo[*b*,*f*]azocin-12-one (2c)

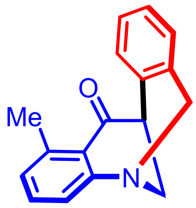

2c

Following the general procedure, preparative thin layer chromatography on a silica gel (petroleum ether: ethyl acetate =4: 1) to give the product **2c** (69.9 mg, 94% yield) as a white solid. m.p. 113.5-114.0 °C. **^{1}H NMR** (400 MHz, $CDCl_3$) δ 7.35 – 7.26 (m, 2H), 7.24 – 7.13 (m, 2H), 7.09 (d, *J* = 8.0 Hz, 1H), 6.95 (d, *J* = 7.1 Hz, 1H), 6.84 (d, *J* = 7.4 Hz, 1H), 4.79 (d, *J* = 16.9 Hz, 1H), 4.33 (d, *J* = 16.9 Hz, 1H), 4.03 (d, *J* = 13.4 Hz, 1H), 3.64 (dd, *J* = 13.3, 1.4 Hz, 1H), 3.45 (s, 1H), 2.58 (s, 3H). **^{13}C NMR** (100 MHz, $CDCl_3$) δ δ 196.9, 155.7, 142.5, 133.6, 132.6, 132.5, 129.3, 128.2, 127.7, 127.0, 126.6, 123.4, 123.2, 58.1, 49.9, 46.7, 23.0. **HPLC conditions**: Daicel Chiralpak OD-H column (hexane/ isopropanol = 90/ 10, flow rate 1.0 mL/min, 254 nm); t_R = 6.912 (major), t_R = 7.829 (minor), 85% *ee*. HRMS (ESI): m/z: Calcd for $(C_{17}H_{15}NO+H)^+$ 250.1226, found 250.1232. $[\alpha]_D^{20}$ = +533.346 (c = 1.054, $CHCl_3$).

(5*S*,11*S*)-2-(trifluoromethyl)-6,11-dihydro-12*H*-5,11-methanodibenzo[*b*,*f*]azocin-12-one (2d)

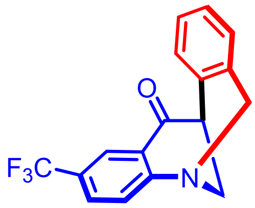

2d

Following the general procedure, preparative thin layer chromatography on a silica gel (petroleum ether: ethyl acetate =4: 1) to give the product **2d** (59.7 mg, 66% yield) as a yellow solid. m.p. 57.2-57.5 °C. **^{1}H NMR** (400 MHz, $CDCl_3$) δ 8.16 (s, 1H), 7.66 (d, *J* = 8.3 Hz, 1H), 7.35 – 7.28 (m, 2H), 7.24 – 7.18 (m, 2H), 6.96 (d, *J* = 7.3 Hz, 1H), 4.84 (d, *J* = 16.9 Hz, 1H), 4.33 (d, *J* = 16.9 Hz, 1H), 4.04 (d, *J* = 13.6 Hz, 1H), 3.71 (d, *J* = 13.6 Hz, 1H), 3.55 (s, 1H). **^{13}C NMR** (100 MHz, $CDCl_3$) δ 193.8, 157.2, 132.2, 131.3, 131.0 (dd, *J* = 6.6, 3.2 Hz), 129.5, 128.7, 127.5, 126.8, 125.7, 125.2, 125.11 (overlap), 125.0, 57.5, 50.0, 44.6. **^{19}F NMR** (377 MHz, $CDCl_3$) δ -62.5. **HPLC conditions**: Daicel Chiralpak OD-H column (hexane/ isopropanol = 90/ 10, flow rate 1.0 mL/min, 254 nm); t_R = 7.710 (major), t_R = 11.166 (minor), 93% *ee*. HRMS (ESI): m/z: Calcd for $(C_{17}H_{12}F_3NO+H)^+$ 304.0944, found 304.0936. $[\alpha]_D^{20}$ = +420.443 (c = 1.154, $CHCl_3$).

(5*S*,11*S*)-2-chloro-6,11-dihydro-12*H*-5,11-methanodibenzo[*b*,*f*]azocin-12-one (2e)

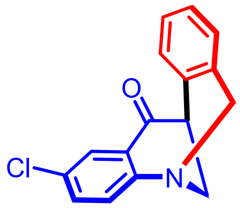

2e

Following the general procedure, preparative thin layer chromatography on a silica gel (petroleum ether: ethyl acetate =4: 1) to give the product **2e** (28 mg, 35% yield) as yellow oil. **^{1}H NMR** (400 MHz, $CDCl_3$) δ 7.92 – 7.72 (m, 1H), 7.41 – 7.37 (m, 1H), 7.31 – 7.26 (m, 1H), 7.25 – 7.20 (m, 1H), 7.20 – 7.15 (m, 2H), 6.95 (d, *J* = 7.3 Hz, 1H), 4.79 (d, *J* = 17.0 Hz, 1H), 4.24 (d, *J* = 16.9 Hz, 1H), 4.01 (dt, *J* = 13.6, 1.5 Hz, 1H), 3.67 (dd, *J* = 13.5, 2.5 Hz, 1H), 3.49 (s, 1H). **^{13}C NMR** (100 MHz, $CDCl_3$) δ 194.0, 152.7, 134.8, 132.2, 131.5, 129.8, 129.5, 128.7, 127.41, 127.1, 126.9, 126.6, 126.4, 57.5, 50.2, 44.5. **HPLC conditions**: Daicel Chiralpak IC column (hexane/ isopropanol = 90/ 10, flow rate 1.0 mL/min, 254 nm); t_R = 7.012 (minor), t_R = 7.410 (major), 95% *ee*. HRMS (ESI): m/z: Calcd for $(C_{16}H_{12}ClNO+H)^+$ 270.0680, found 270.0684. $[\alpha]_D^{20}$ = +514.293 (c = 0.34, $CHCl_3$).

(5*S*,11*S*)-2-(*tert*-butyl)-6,11-dihydro-12*H*-5,11-methanodibenzo[*b*,*f*]azocin-12-one (2f)

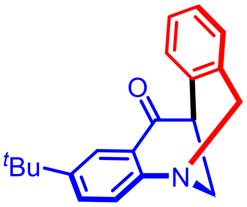

2f

Following the general procedure, preparative thin layer chromatography on a silica gel (petroleum ether: ethyl acetate =4: 1) to give the product **2f** (81.8 mg, 94% yield) as yellow oil. **^{1}H NMR** (400 MHz, $CDCl_3$) δ 7.94 – 7.81 (m, 1H), 7.56 – 7.48 (m, 1H), 7.31 (d, *J* = 7.3 Hz, 1H), 7.23 – 7.13 (m, 3H), 6.95 (d, *J* = 7.1 Hz, 1H), 4.79 (d, *J* = 17.0 Hz, 1H), 4.27 (d, *J* = 17.0 Hz, 1H), 4.04 (d, *J* = 13.4 Hz, 1H), 3.65 (d, *J* = 13.4 Hz, 1H), 3.48 (s, 1H), 1.27 (s, 9H). **^{13}C NMR** (100 MHz, $CDCl_3$) δ 195.6, 151.9, 147.1, 132.7, 132.6, 132.2, 129.4, 128.3, 127.1, 126.8, 124.7, 124.6, 123.6, 57.4, 50.5, 45.1, 34.4, 31.1. **HPLC conditions**: Daicel Chiralpak IG column (hexane/ isopropanol = 90/ 10, flow rate 1.0 mL/min, 254 nm); t_R = 8.233 (minor), t_R = 9.329 (major), 94% *ee*. HRMS (ESI): m/z: Calcd for $(C_{20}H_{21}NO+H)^+$ 292.1696, found 292.1703. $[\alpha]_D^{20}$ = +353.320 (c = 1.35, $CHCl_3$).

(5*S*,11*S*)-2-methoxy-6,11-dihydro-12*H*-5,11-methanodibenzo[*b*,*f*]azocin-12-one (2g)

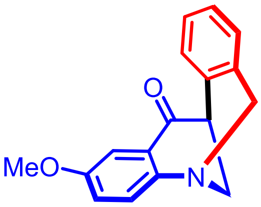

2g

Following the general procedure, preparative thin layer chromatography on a silica gel (petroleum ether: ethyl acetate =4: 1) to give the product **2g** (68.9 mg, 87% yield) as yellow oil. **^{1}H NMR** (400 MHz, $CDCl_3$) δ 7.36 – 7.26 (m, 2H), 7.22 – 7.12 (m, 3H), 7.07 – 7.01 (m, 1H), 6.93 (d, *J* = 7.2 Hz, 1H), 4.75 (d, *J* = 17.0 Hz, 1H), 4.20 (d, *J* = 17.0 Hz, 1H), 4.02 (d, *J* = 13.4 Hz, 1H), 3.73 (s, 3H), 3.64 (d, *J* = 13.4 Hz, 1H), 3.46 (s, 1H). **^{13}C NMR** (100 MHz, $CDCl_3$) δ 195.3, 156.2, 147.8, 132.4, 131.9, 129.2, 128.4, 127.1, 126.8, 126.3, 125.6, 123.7, 108.5, 57.3, 55.5, 50.6, 44.8.

HPLC conditions: Daicel Chiralpak IC column (hexane/ isopropanol = 90/ 10, flow rate 1.0 mL/min, 254 nm); t_R = 10.558 (minor), t_R = 14.867 (major), 93% *ee*. HRMS (ESI): m/z: Calcd for $(C_{17}H_{15}NO_2+Na)^+$ 288.0995, found 288.1003. $[\alpha]_D^{20}$ = +366.636 (c = 1.098, $CHCl_3$).

(5*S*,11*S*)-2-(dimethylamino)-6,11-dihydro-12*H*-5,11-methanodibenzo[*b*,*f*]azocin-12-one (2h)

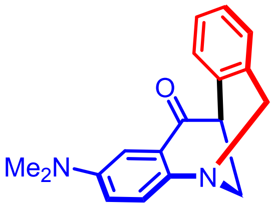

2h

Following the general procedure, preparative thin layer chromatography on a silica gel (petroleum ether: ethyl acetate =4: 1) to give the product **2h** (75.2 mg, 90% yield) as a yellow solid. m.p. 107.3-108.0 °C. **^{1}H NMR** (400 MHz, $CDCl_3$) δ 7.30 (d, *J* = 7.2 Hz, 1H), 7.21 – 7.08 (m, 4H), 6.98 – 6.87 (m, 2H), 4.74 (d, *J* = 17.0 Hz, 1H), 4.18 (d, *J* = 17.0 Hz, 1H), 4.02 (d, *J* = 13.3 Hz, 1H), 3.62 (dd, *J* = 13.3, 1.5 Hz, 1H), 3.45 (s, 1H), 2.86 (s, 6H). **^{13}C NMR** (100 MHz, $CDCl_3$) δ 195.9, 147.6, 144.2, 132.6, 132.1, 129.2, 128.2, 126.9, 126.8, 125.6, 125.4, 120.5, 109.2, 57.3, 50.7, 45.1, 40.7. **HPLC conditions**: Daicel Chiralpak AD-H column (hexane/ isopropanol = 90/ 10, flow rate 1.0 mL/min, 254 nm); t_R = 11.968 (minor), t_R = 13.600 (major), 94% *ee*. HRMS (ESI): m/z: Calcd for $(C_{18}H_{18}N_2O+H)^+$ 279.1492, found 279.1496. $[\alpha]_D^{20}$ = +332.061 (c = 1.504, $CHCl_3$).

(5*S*,11*S*)-3-chloro-6,11-dihydro-12*H*-5,11-methanodibenzo[*b*,*f*]azocin-12-one (2i)

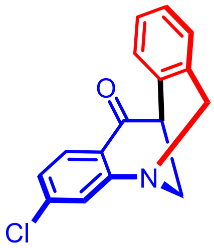

2i

Following the general procedure, preparative thin layer chromatography on a silica gel (petroleum ether: ethyl acetate =4: 1) to give the product **2i** (49 mg, 61% yield) as a yellow solid. m.p. 132.8-133.4 °C. **^{1}H NMR** (400 MHz, $CDCl_3$) δ 7.36 – 7.26 (m, 2H), 7.24 – 7.17 (m, 2H), 7.14 (d, *J* = 8.2 Hz, 1H), 7.05 (d, *J* = 7.8 Hz, 1H), 6.95 (d, *J* = 7.2 Hz, 1H), 4.79 (d, *J* = 16.9 Hz, 1H), 4.33 (d, *J* = 16.9 Hz, 1H), 4.02 (d, *J* = 13.5 Hz, 1H), 3.63 (dd, *J* = 13.5, 1.4 Hz, 1H), 3.49 (s, 1H). **^{13}C NMR** (100 MHz, $CDCl_3$) δ 193.1, 156.8, 135.4, 133.8, 132.2, 131.8, 129.5, 128.4, 127.5, 127.3, 126.6, 124.1, 121.9, 58.2, 49.5, 46.0. **HPLC conditions**: Daicel Chiralpak OD-H column (hexane/ isopropanol = 90/ 10, flow rate 1.0 mL/min, 254 nm); t_R = 11.444 (major), t_R = 13.799 (minor), 88% *ee*. HRMS (ESI): m/z: Calcd for $(C_{16}H_{12}ClNO+Na)^+$ 292.0500, found 292.0505. $[\alpha]_D^{20}$ = +610.005 (c = 0.966, $CHCl_3$).

(5*S*,11*S*)-3-methyl-6,11-dihydro-12*H*-5,11-methanodibenzo[*b*,*f*]azocin-12-one (2j)

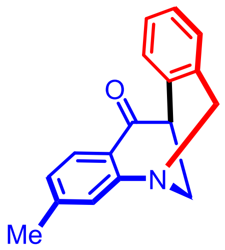

2j

Following the general procedure, preparative thin layer chromatography on a silica gel (petroleum ether: ethyl acetate =4: 1) to give the product **2j** (67.6 mg, 90% yield) as a white solid. m.p. 132.8-133.4 °C. **^{1}H NMR** (400 MHz, $CDCl_3$) δ 7.76 (d, *J* = 7.9 Hz, 1H), 7.29 (d, *J* = 7.2 Hz, 1H), 7.23 – 7.12 (m, 2H), 7.01 (s, 1H), 6.94 (d, *J* = 7.2 Hz, 1H), 6.88 (d, *J* = 7.9 Hz, 1H), 4.78 (d, *J* = 16.9 Hz, 1H), 4.28 (d, *J* = 16.9 Hz, 1H), 4.02 (d, *J* = 13.4 Hz, 1H), 3.66 (dd, *J* = 13.4, 1.2 Hz, 1H), 3.45 (s, 1H), 2.34 (s, 3H). **^{13}C NMR** (100 MHz, $CDCl_3$) δ 195.1, 154.4, 146.1, 132.6, 132.2, 129.3, 128.3, 127.4, 127.1, 126.8, 125.4, 125.2, 122.9, 57.5, 50.5, 45.0, 21.8. **HPLC conditions**: Daicel Chiralpak OD-H column (hexane/ isopropanol = 90/ 10, flow rate 1.0 mL/min, 254 nm); t_R = 8.615 (minor), t_R = 9.664 (major), 94% *ee*. HRMS (ESI): m/z: Calcd for $(C_{17}H_{15}NO+Na)^+$ 272.1046, found 272.1050. $[\alpha]_D^{20}$ = +284.547 (c = 0.986, $CHCl_3$).

(7*S*,13*S*)-8,13-dihydro-14*H*-7,13-methanobenzo[*f*]naphtho[2,1-*b*]azocin-14-one (2k)

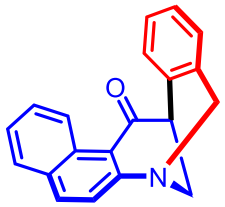

2k

Following the general procedure, preparative thin layer chromatography on a silica gel (petroleum ether: ethyl acetate =4: 1) to give the product **2k** (53.4 mg, 62% yield) as a yellow solid. m.p. > 250 °C. **^{1}H NMR** (400 MHz, $CDCl_3$) δ 9.43 (d, *J* = 8.7 Hz, 1H), 7.87 (d, *J* = 8.8 Hz, 1H), 7.69 (d, *J* = 8.0 Hz, 1H), 7.63 – 7.54 (m, 1H), 7.44 – 7.34 (m, 2H), 7.31 (d, *J* = 8.8 Hz, 1H), 7.22 – 7.12 (m, 2H), 6.95 (d, *J* = 7.2 Hz, 1H), 4.85 (d, *J* = 16.8 Hz, 1H), 4.52 (d, *J* = 16.8 Hz, 1H), 4.22 (d, *J* = 13.2 Hz, 1H), 3.73 (d, *J* = 13.2 Hz, 1H), 3.52 (s, 1H). **^{13}C NMR** (100 MHz, $CDCl_3$) δ 197.1, 156.3, 136.1, 133.0, 132.2, 132.0, 131.0, 129.4, 129.3, 128.4, 128.3, 127.3, 126.7, 126.5, 125.2, 124.0, 117.2, 56.9, 50.1, 47.0. **HPLC conditions**: Daicel Chiralpak OD-H column (hexane/ isopropanol = 90/ 10, flow rate 1.0 mL/min, 254 nm); t_R = 9.345 (major), t_R = 11.803 (minor), 92% *ee*. HRMS (ESI): m/z: Calcd for $(C_{20}H_{15}NO+Na)^+$ 308.1046, found 308.1042. $[\alpha]_D^{20}$ = +873.9 (c = 1.04, $CHCl_3$).

(5*S*,11*S*)-1,3-dimethyl-6,11-dihydro-12*H*-5,11-methanodibenzo[*b*,*f*]azocin-12-one (2l)

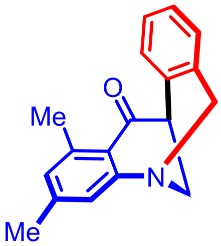

2l

Following the general procedure, preparative thin layer chromatography on a silica gel (petroleum ether: ethyl acetate =4: 1) to give the product **2l** (74.9 mg, 95% yield) as a yellow solid. m.p. 119.4-120.1 °C. **^{1}H NMR** (400 MHz, $CDCl_3$) δ 7.30 (d, J = 7.3 Hz, 1H), 7.21 – 7.13 (m, 2H), 6.95 (d, J = 7.1 Hz, 1H), 6.90 (s, 1H), 6.67 (s, 1H), 4.78 (d, J = 16.9 Hz, 1H), 4.33 (d, J = 16.9 Hz, 1H), 4.00 (d, J = 13.3 Hz, 1H), 3.61 (d, J = 13.3 Hz, 1H), 3.42 (s, 1H), 2.55 (s, 3H), 2.28 (s, 3H). **^{13}C NMR** (100 MHz, $CDCl_3$) δ 196.6, 155.7, 144.5, 142.3, 132.8, 132.7, 129.2, 128.9, 128.0, 127.0, 126.5, 123.5, 121.0, 58.1, 49.9, 46.7, 22.9, 21.5. **HPLC conditions**: Daicel Chiralpak AD-H column (hexane/ isopropanol = 90/ 10, flow rate 1.0 mL/min, 254 nm); t_R = 5.338 (minor), t_R = 6.638 (major), 93% *ee*. HRMS (ESI): m/z: Calcd for $(C_{18}H_{17}NO+Na)^+$ 286.1202, found 286.1204. $[\alpha]_D^{20}$ = +357.448 (c = 1.454, $CHCl_3$).

(5*S*,11*S*)-9-(trifluoromethyl)-6,11-dihydro-12*H*-5,11-methanodibenzo[*b*,*f*]azocin-12-one (2m)

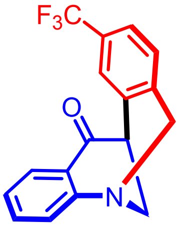

2m

Following the general procedure, preparative thin layer chromatography on a silica gel (petroleum ether: ethyl acetate =4: 1) to give the product **2m** (75.1 mg, 83% yield) as a yellowish solid. m.p. 122.9-123.5 °C. **^{1}H NMR** (400 MHz, $CDCl_3$) δ 7.87 (dd, J = 7.8, 1.5 Hz, 1H), 7.57 (s, 1H), 7.51 – 7.43 (m, 2H), 7.22 (dd, J = 8.1, 0.7 Hz, 1H), 7.13 – 7.06 (m, 2H), 4.81 (d, J = 17.4 Hz, 1H), 4.34 (d, J = 17.3 Hz, 1H), 4.08 (dt, J = 13.6, 1.5 Hz, 1H), 3.67 (dd, J = 13.6, 2.4 Hz, 1H), 3.54 (s, 1H). **^{13}C NMR** (100 MHz, $CDCl_3$) δ 194.3, 154.1, 136.7, 135.3, 132.9, 129.8, 129.4, 127.7, 127.4, 126.3 (q, J = 3.7 Hz), 125.1 (dd, J = 8.9, 3.7 Hz), 125.0, 124.5, 57.4, 50.1, 44.9. ^{19}F NMR (377 MHz, $CDCl_3$) δ -62.6. **HPLC conditions**: Daicel Chiralpak OD-H column (hexane/ isopropanol = 90/ 10, flow rate 1.0 mL/min, 254 nm); t_R = 8.198 (major), t_R = 9.171 (minor), 94% *ee*. HRMS (ESI): m/z: Calcd for $(C_{17}H_{12}F_3NO+Na)^+$ 326.0763, found 326.0756. $[\alpha]_D^{20}$ = +338.84 (c = 0.474, $CHCl_3$).

(5*S*,11*S*)-9-fluoro-6,11-dihydro-12*H*-5,11-methanodibenzo[*b*,*f*]azocin-12-one (2n)

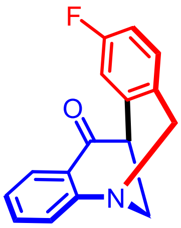

2n

Following the general procedure, preparative thin layer chromatography on a silica gel (petroleum ether: ethyl acetate =4: 1) to give the product **2n** (65.1 mg, 86% yield) as a yellow solid. m.p. 103.5-104.1 °C. **^{1}H NMR** (400 MHz, $CDCl_3$) δ 7.94 – 7.77 (m, 1H), 7.51 – 7.41 (m, 1H), 7.20 (d, *J* = 8.1 Hz, 1H), 7.10 – 7.04 (m, 1H), 7.01 (d, *J* = 8.9 Hz, 1H), 6.89 (d, *J* = 6.2 Hz, 2H), 4.74 (d, *J* = 16.8 Hz, 1H), 4.26 (d, *J* = 16.8 Hz, 1H), 4.02 (d, *J* = 13.5 Hz, 1H), 3.63 (dd, *J* = 13.5, 2.3 Hz, 1H), 3.44 (s, 1H). **^{13}C NMR** (100 MHz, $CDCl_3$) δ 194.6, 161.5 (d, *J* = 244.8 Hz), 154.2, 135.1, 133.8 (d, *J* = 7.3 Hz), 128.3 (d, *J* = 8.0 Hz), 127.9 (d, *J* = 3.0 Hz), 127.5, 125.1, 125.0, 124.3, 115.8 (d, *J* = 21.2 Hz), 115.6 (d, *J* = 21.4 Hz), 57.1, 50.0, 45.0. ^{19}F NMR (377 MHz, $CDCl_3$) δ -114.9. **HPLC conditions**: Daicel Chiralpak OD-H column (hexane/ isopropanol = 90/ 10, flow rate 1.0 mL/min, 254 nm); t_R = 9.066 (major), t_R = 9.519 (minor), 94% *ee*. HRMS (ESI): m/z: Calcd for $(C_{16}H_{12}FNO+Na)^+$ 276.0795, found 276.0797. $[\alpha]_D^{20}$ = +351.298 (c = 1.16, $CHCl_3$).

(5*S*,11*S*)-9-chloro-6,11-dihydro-12*H*-5,11-methanodibenzo[*b*,*f*]azocin-12-one (2o)

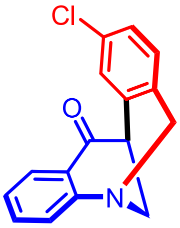

2o

Following the general procedure, preparative thin layer chromatography on a silica gel (petroleum ether: ethyl acetate =4: 1) to give the product **2o** (58.3 mg, 72% yield) as a yellow solid. m.p. 108.2-109.1 °C. **^{1}H NMR** (400 MHz, $CDCl_3$) δ 7.86 (dd, *J* = 7.8, 1.3 Hz, 1H), 7.51 – 7.41 (m, 1H), 7.35 – 7.27 (m, 1H), 7.22 – 7.14 (m, 2H), 7.11 – 7.05 (m, 1H), 6.87 (d, *J* = 8.2 Hz, 1H), 4.74 (d, *J* = 17.0 Hz, 1H), 4.25 (d, *J* = 17.0 Hz, 1H), 4.03 (d, *J* = 13.6 Hz, 1H), 3.63 (dd, *J* = 13.6, 2.3 Hz, 1H), 3.44 (s, 1H). **^{13}C NMR** (100 MHz, $CDCl_3$) δ 194.5, 154.1, 135.1, 133.7, 132.8, 130.8, 129.1, 128.6, 128.1, 127.6, 125.1, 125.0, 124.3, 57.1, 50.0, 44.8. **HPLC conditions**: Daicel Chiralpak IC column (hexane/ isopropanol = 90/ 10, flow rate 1.0 mL/min, 254 nm); t_R = 8.667 (major), t_R = 11.656 (minor), 96% *ee*. HRMS (ESI): m/z: Calcd for $(C_{16}H_{12}ClNO+Na)^+$ 292.0500, found 292.0498. $[\alpha]_D^{20}$ = +217.718 (c = 0.974, $CHCl_3$).

(5*S*,11*S*)-9-methyl-6,11-dihydro-12*H*-5,11-methanodibenzo[*b*,*f*]azocin-12-one (2p)

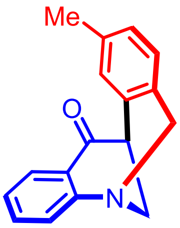

2p

Following the general procedure, preparative thin layer chromatography on a silica gel (petroleum ether: ethyl acetate =4: 1) to give the product **2p** (52 mg, 70% yield) as colorless oil. **^{1}H NMR** (400 MHz, $CDCl_3$) δ 7.87 (d, *J* = 7.8 Hz, 1H), 7.50 – 7.39 (m, 1H), 7.20 (d, *J* = 8.1 Hz, 1H), 7.12 (s, 1H), 7.09 – 7.04 (m, 1H), 7.02 (d, *J* = 7.7 Hz, 1H), 6.83 (d, *J* = 7.7 Hz, 1H), 4.76 (d, *J* = 16.8 Hz, 1H), 4.25 (d, *J* = 16.8 Hz, 1H), 4.04 (d, *J* = 13.5 Hz, 1H), 3.65 (d, *J* = 13.5 Hz, 1H), 3.44 (s, 1H), 2.27 (s, 3H). **^{13}C NMR** (100 MHz, $CDCl_3$) δ 195.4, 154.4, 136.9, 134.9, 131.7, 129.8, 129.3, 129.2, 127.5, 126.7, 125.3, 125.0, 124.1, 57.4, 50.4, 45.0, 20.9. **HPLC conditions**: Daicel Chiralpak OD-H column (hexane/ isopropanol = 90/ 10, flow rate 1.0 mL/min, 254 nm); t_R = 6.974 (major), t_R = 7.380 (minor), 92% *ee*. HRMS (ESI): m/z: Calcd for $(C_{17}H_{15}NO+Na)^+$ 272.1046, found 272.1051. $[\alpha]_D^{20}$ = +331.515 (c = 0.43, $CHCl_3$).

(5*S*,11*S*)-9-methoxy-6,11-dihydro-12*H*-5,11-methanodibenzo[*b*,*f*]azocin-12-one (2q)

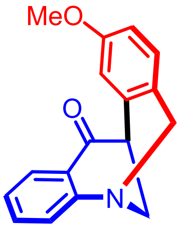

2q

Following the general procedure, preparative thin layer chromatography on a silica gel (petroleum ether: ethyl acetate =4: 1) to give the product **2q** (74.7 mg, 94% yield) as yellow oil. **^{1}H NMR** (400 MHz, $CDCl_3$) δ 7.86 (dd, *J* = 7.8, 1.5 Hz, 1H), 7.47 – 7.41 (m, 1H), 7.20 (d, *J* = 7.8 Hz, 1H), 7.08 – 7.03 (m, 1H), 6.85 – 6.80 (m, 2H), 6.76 (dd, *J* = 8.4, 2.6 Hz, 1H), 4.74 (d, *J* = 16.6 Hz, 1H), 4.23 (d, *J* = 16.5 Hz, 1H), 4.01 (d, *J* = 13.5 Hz, 1H), 3.74 (s, 3H), 3.66 – 3.61 (m, 1H), 3.43 (s, 1H). **^{13}C NMR** (100 MHz, $CDCl_3$) δ 195.1, 158.5, 154.4, 134.9, 132.9, 127.7, 127.4, 125.2, 125.0, 124.2, 124.0, 115.4, 113.3, 57.1, 55.2, 50.2, 45.3. **HPLC conditions**: Daicel Chiralpak AD-H column (hexane/ isopropanol = 90/ 10, flow rate 1.0 mL/min, 254 nm); t_R = 8.766 (minor), t_R = 10.017 (major), 96% *ee*. HRMS (ESI): m/z: Calcd for $(C_{17}H_{15}NO_2+Na)^+$ 288.0995, found 288.0992. $[\alpha]_D^{20}$ = +285.691 (c = 1.496, $CHCl_3$).

(5*S*,11*S*)-8-(trifluoromethyl)-6,11-dihydro-12*H*-5,11-methanodibenzo[*b*,*f*]azocin-12-one (2r)

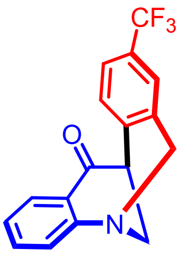

2r

Following the general procedure, preparative thin layer chromatography on a silica gel (petroleum ether: ethyl acetate =4: 1) to give the product **2r** (76.8 mg, 84% yield) as a yellow solid. m.p. 105.8-106.4 °C. **^{1}H NMR** (400 MHz, $CDCl_3$) δ 7.86 (dd, *J* = 7.8, 1.4 Hz, 1H), 7.51 – 7.45 (m, 1H), 7.42 (s, 2H), 7.25 – 7.19 (m, 2H), 7.11 – 7.06 (m, 1H), 4.81 (d, *J* = 17.1 Hz, 1H), 4.34 (d, *J* = 17.1 Hz, 1H), 4.07 (d, *J* = 13.6 Hz, 1H), 3.67 (dd, *J* = 13.6, 2.3 Hz, 1H), 3.55 (s, 1H). **^{13}C NMR** (100 MHz, $CDCl_3$) δ 194.3, 154.1, 136.0, 135.3, 133.4, 130.7, 130.4, 129.9, 127.6, 125.1, 125.0, 124.5, 123.9 (dd, *J* = 7.5, 3.6 Hz), 123.8 (dd, *J* = 7.8, 3.9 Hz), 57.3, 50.0, 45.0. ^{19}F NMR (377 MHz, $CDCl_3$) δ -62.8. **HPLC conditions**: Daicel Chiralpak OD-H column (hexane/ isopropanol = 90/ 10, flow rate 1.0 mL/min, 254 nm); t_R = 8.026 (major), t_R = 8.580 (minor), 94% *ee*. HRMS (ESI): m/z: Calcd for $(C_{17}H_{12}F_3NO+Na)^+$ 326.0763, found 326.0758. $[\alpha]_D^{20}$ = +294.406 (c = 1.44, $CHCl_3$).

(5*S*,11*S*)-8-fluoro-6,11-dihydro-12*H*-5,11-methanodibenzo[*b*,*f*]azocin-12-one (2s)

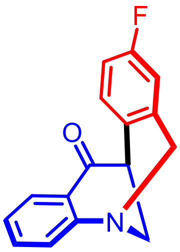

2s

Following the general procedure, preparative thin layer chromatography on a silica gel (petroleum ether: ethyl acetate =4: 1) to give the product **2s** (51 mg, 67% yield) as yellow oil. **^{1}H NMR** (400 MHz, $CDCl_3$) δ 7.86 (d, *J* = 7.8 Hz, 1H), 7.50 – 7.42 (m, 1H), 7.29 – 7.24 (m, 1H), 7.20 (d, *J* = 8.1 Hz, 1H), 7.12 – 7.04 (m, 1H), 6.92 – 6.79 (m, 1H), 6.66 (d, *J* = 8.7 Hz, 1H), 4.76 (d, *J* = 17.1 Hz, 1H), 4.26 (d, *J* = 17.1 Hz, 1H), 4.04 (d, *J* = 13.5 Hz, 1H), 3.62 (d, *J* = 13.5 Hz, 1H), 3.45 (s, 1H). **^{13}C NMR** (100 MHz, $CDCl_3$) δ 195.0, 162.7 (d, *J* = 246.2 Hz), 154.1, 135.1, 134.5 (d, *J* = 6.5 Hz), 130.9 (d, *J* = 7.9 Hz), 127.6 (d, *J* = 3.0 Hz), 127.5, 125.2, 125.0, 124.4, 114.4 (d, *J* = 21.5 Hz), 113.3 (d, *J* = 21.3 Hz), 57.6, 50.3, 44.3. ^{19}F NMR (377 MHz, $CDCl_3$) δ -113.0. **HPLC conditions**: Daicel Chiralpak OD-H column (hexane/ isopropanol = 90/ 10, flow rate 1.0 mL/min, 254 nm); t_R = 8.645 (major), t_R = 10.291 (minor), 92% *ee*. HRMS (ESI): m/z: Calcd for $(C_{16}H_{12}FNO+Na)^+$ 276.0795, found 276.0796. $[\alpha]_D^{20}$ = +384.242 (c = 0.762, $CHCl_3$).

(5*S*,11*S*)-8-chloro-6,11-dihydro-12*H*-5,11-methanodibenzo[*b*,*f*]azocin-12-one (2t)

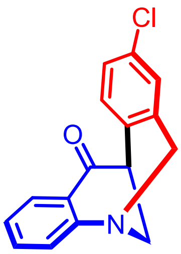

2t

Following the general procedure, preparative thin layer chromatography on a silica gel (petroleum ether: ethyl acetate =4: 1) to give the product **2t** (52.6 mg, 65% yield) as a white solid. m.p. 88.8-89.4 °C. **^{1}H NMR** (400 MHz, $CDCl_3$) δ 7.86 (d, *J* = 7.8 Hz, 1H), 7.51 – 7.43 (m, 1H), 7.24 – 7.18 (m, 2H), 7.13 (d, *J* = 8.2 Hz, 1H), 7.11 – 7.06 (m, 1H), 6.94 (s, 1H), 4.74 (d, *J* = 17.1 Hz, 1H), 4.24 (d, *J* = 17.0 Hz, 1H), 4.04 (d, *J* = 13.5 Hz, 1H), 3.62 (dd, *J* = 13.5, 1.8 Hz, 1H), 3.45 (s, 1H). **^{13}C NMR** (100 MHz, $CDCl_3$) δ 194.7, 154.1, 135.1, 134.2, 134.1, 130.6, 130.4, 127.6, 127.4, 126.8, 125.1, 125.0, 124.4, 57.3, 50.2, 44.4. **HPLC conditions**: Daicel Chiralpak OD-H column (hexane/ isopropanol = 90/ 10, flow rate 1.0 mL/min, 254 nm); t_R = 9.035 (major), t_R = 10.220 (minor), 95% *ee*. HRMS (ESI): m/z: Calcd for $(C_{16}H_{12}ClNO+Na)^+$ 292.0500, found 292.0497. $[\alpha]_D^{20}$ = +595.730 (c = 0.744, $CHCl_3$).

(5*S*,11*S*)-8-methyl-6,11-dihydro-12*H*-5,11-methanodibenzo[*b*,*f*]azocin-12-one (2u)

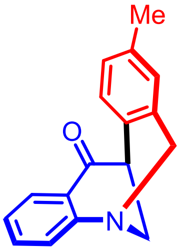

2u

Following the general procedure, preparative thin layer chromatography on a silica gel (petroleum ether: ethyl acetate =4: 1) to give the product **2u** (66.9 mg, 90% yield) as yellow oil. **^{1}H NMR** (400 MHz, $CDCl_3$) δ 7.87 (dd, *J* = 7.8, 1.5 Hz, 1H), 7.47 – 7.42 (m, 1H), 7.22 – 7.17 (m, 2H), 7.08 – 7.03 (m, 1H), 6.98 (d, *J* = 7.9 Hz, 1H), 6.76 (s, 1H), 4.77 (d, *J* = 16.9 Hz, 1H), 4.25 (d, *J* = 16.8 Hz, 1H), 4.03 (dt, *J* = 13.5, 1.5 Hz, 1H), 3.65 (dd, *J* = 13.4, 2.5 Hz, 1H), 3.45 (s, 1H), 2.24 (s, 3H). **^{13}C NMR** (100 MHz, $CDCl_3$) δ 195.3, 154.4, 138.2, 134.8, 132.2, 129.2, 128.9, 128.0, 127.5, 127.3, 125.3, 125.0, 124.1, 57.6, 50.5, 44.6, 21.0. **HPLC conditions**: Daicel Chiralpak AD-H column (hexane/ isopropanol = 90/ 10, flow rate 1.0 mL/min, 254 nm); t_R = 9.571 (minor), t_R = 10.604 (major), 91% *ee*. HRMS (ESI): m/z: Calcd for $(C_{17}H_{15}NO+Na)^+$ 272.1046, found 272.1047. $[\alpha]_D^{20}$ = +575.096 (c = 0.846, $CHCl_3$).

(5*S*,11*S*)-8-methoxy-6,11-dihydro-12*H*-5,11-methanodibenzo[*b*,*f*]azocin-12-one (2v)

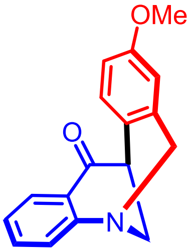

2v

Following the general procedure, preparative thin layer chromatography on a silica gel (petroleum ether: ethyl acetate =4: 1) to give the product **2v** (59.1 mg, 74% yield) as brown oil. **^{1}H NMR** (400 MHz, $CDCl_3$) δ 7.86 (d, *J* = 7.8 Hz, 1H), 7.47 – 7.41 (m, 1H), 7.22 – 7.17 (m, 2H), 7.09 – 7.03 (m, 1H), 6.72 (d, *J* = 8.4 Hz, 1H), 6.47 (s, 1H), 4.76 (d, *J* = 16.9 Hz, 1H), 4.25 (d, *J* = 16.9 Hz, 1H), 4.02 (d, *J* = 13.3 Hz, 1H), 3.71 (s, 3H), 3.63 (d, *J* = 13.6 Hz, 1H), 3.42 (s, 1H). **^{13}C NMR** (100 MHz, $CDCl_3$) δ 195.4, 159.7, 154.2, 134.8, 133.6, 130.3, 127.5, 125.3, 124.9, 124.2, 123.9, 113.2, 111.6, 57.8, 55.1, 50.6, 44.2. **HPLC conditions**: Daicel Chiralpak OD-H column (hexane/ isopropanol = 90/ 10, flow rate 1.0 mL/min, 254 nm); t_R = 9.114 (major), t_R = 11.665 (minor), 94% *ee*. HRMS (ESI): m/z: Calcd for $(C_{17}H_{15}NO_2+Na)^+$ 288.0995, found 288.0999. $[\alpha]_D^{20}$ = +490.913 (c = 1.22, $CHCl_3$).

(6*S*,12*S*)-5,12-dihydro-11*H*-6,12-methano[1,3]dioxolo[4',5':4,5]benzo[1,2-*f*]benzo[*b*]azocin-11-one (2w)

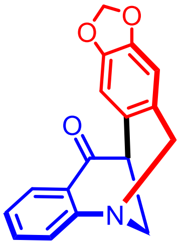

2w

Following the general procedure, preparative thin layer chromatography on a silica gel (petroleum ether: ethyl acetate =4: 1) to give the product **2w** (65.7 mg, 78% yield) as a yellow solid. m.p. 161.3-161.6 °C. **^{1}H NMR** (400 MHz, $CDCl_3$) δ 7.86 (d, *J* = 7.7 Hz, 1H), 7.49 – 7.41 (m, 1H), 7.18 (d, *J* = 8.1 Hz, 1H), 7.10 – 7.04 (m, 1H), 6.74 (s, 1H), 6.36 (s, 1H), 5.87 (s, 1H), 5.80 (s, 1H), 4.69 (d, *J* = 16.7 Hz, 1H), 4.14 (d, *J* = 16.8 Hz, 1H), 3.96 (d, *J* = 13.4 Hz, 1H), 3.59 (dd, *J* = 13.4, 1.6 Hz, 1H), 3.32 (s, 1H). **^{13}C NMR** (100 MHz, $CDCl_3$) δ 195.2, 154.2, 148.0, 146.7, 134.8, 127.4, 125.3, 125.2, 125.0, 124.8, 124.2, 108.9, 106.5, 100.9, 57.7, 50.3, 44.8. **HPLC conditions**: Daicel Chiralpak AD-H column (hexane/ isopropanol = 90/ 10, flow rate 1.0 mL/min, 254 nm); t_R = 12.653 (minor), t_R = 17.745 (major), 96% *ee*. HRMS (ESI): m/z: Calcd for $(C_{17}H_{13}NO_3+Na)^+$ 302.0788, found 302.0785. $[\alpha]_D^{20}$ = +390.108 (c = 1.298, $CHCl_3$).

(5*S*,11*S*)-8,9-dimethoxy-6,11-dihydro-12*H*-5,11-methanodibenzo[*b*,*f*]azocin-12-one (2x)

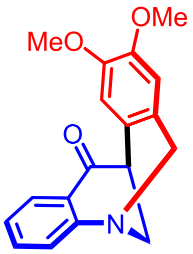

2x

Following the general procedure, preparative thin layer chromatography on a silica gel (petroleum ether: ethyl acetate =4: 1) to give the product **2x** (81 mg, 92% yield) as yellow oil. **^{1}H NMR** (400 MHz, $CDCl_3$) δ 7.85 (d, J = 7.8 Hz, 1H), 7.48 – 7.39 (m, 1H), 7.18 (d, J = 8.1 Hz, 1H), 7.09 – 7.02 (m, 1H), 6.75 (s, 1H), 6.40 (s, 1H), 4.73 (d, J = 16.6 Hz, 1H), 4.18 (d, J = 16.7 Hz, 1H), 3.99 (d, J = 13.3 Hz, 1H), 3.82 (s, 3H), 3.75 (s, 3H), 3.61 (d, J = 13.4 Hz, 1H), 3.35 (s, 1H). **^{13}C NMR** (100 MHz, $CDCl_3$) δ 195.4, 154.3, 149.4, 148.1, 134.8, 127.4, 125.3, 124.9, 124.1, 124.0, 123.7, 111.5, 109.1, 57.3, 55.9, 55.7, 50.4, 44.4. **HPLC conditions**: Daicel Chiralpak OD-H column (hexane/ isopropanol = 90/ 10, flow rate 1.0 mL/min, 254 nm); t_R = 13.688 (major), t_R = 17.456 (minor), 94% *ee*. HRMS (ESI): m/z: Calcd for $(C_{18}H_{17}NO_3+Na)^+$ 318.1101, found 318.1094. $[\alpha]_D^{20}$ = +435.468 (c = 1.578, $CHCl_3$).

(5*S*,11*S*)-9-chloro-2-methoxy-6,11-dihydro-12*H*-5,11-methanodibenzo[*b*,*f*]azocin-12-one (2y)

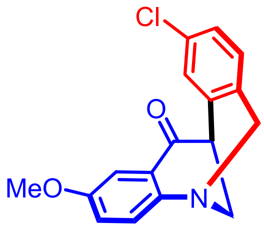

2y

Following the general procedure, preparative thin layer chromatography on a silica gel (petroleum ether: ethyl acetate =4: 1) to give the product **2y** (79.7 mg, 89% yield) as yellow oil. **^{1}H NMR** (400 MHz, $CDCl_3$) δ 7.34 – 7.27 (m, 2H), 7.17 – 7.09 (m, 2H), 7.07 – 7.01 (m, 1H), 6.85 (d, J = 8.2 Hz, 1H), 4.68 (d, J = 17.1 Hz, 1H), 4.16 (d, J = 17.0 Hz, 1H), 4.00 (d, J = 13.5 Hz, 1H), 3.74 (s, 3H), 3.59 (dd, J = 13.5, 2.4 Hz, 1H), 3.41 (s, 1H). **^{13}C NMR** (100 MHz, $CDCl_3$) δ 194.6, 156.3, 147.6, 133.7, 132.7, 130.8, 129.0, 128.6, 128.2, 126.3, 125.4, 123.9, 108.4, 56.8, 55.5, 50.3, 44.5. **HPLC conditions**: Daicel Chiralpak AD-H column (hexane/ isopropanol = 90/ 10, flow rate 1.0 mL/min, 254 nm); t_R = 9.847 (minor), t_R = 11.720 (major), 92% *ee*. HRMS (ESI): m/z: Calcd for $(C_{17}H_{14}ClNO_2+H)^+$ 300.0786, found 300.0779. $[\alpha]_D^{20}$ = +186.259 (c = 1. 504, $CHCl_3$).

(5*S*,11*S*)-8-fluoro-2-methoxy-6,11-dihydro-12*H*-5,11-methanodibenzo[*b*,*f*]azocin-12-one (2z)

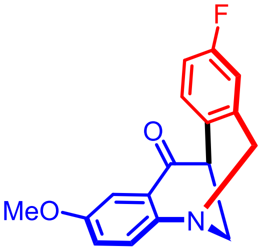

2z

Following the general procedure, preparative thin layer chromatography on a silica gel (petroleum ether: ethyl acetate =4: 1) to give the product **2z** (79.1 mg, 93% yield) as a yellow solid. m.p. 121.5-121.8 °C. **^{1}H NMR** (400 MHz, $CDCl_3$) δ 7.36 – 7.28 (m, 1H), 7.28 – 7.23 (m, 1H), 7.13 (d, *J* = 8.8 Hz, 1H), 7.05 (dd, *J* = 8.8, 2.8 Hz, 1H), 6.91 – 6.81 (m, 1H), 6.65 (d, *J* = 8.6 Hz, 1H), 4.71 (d, *J* = 17.1 Hz, 1H), 4.17 (d, *J* = 17.1 Hz, 1H), 4.01 (d, *J* = 13.5 Hz, 1H), 3.74 (s, 3H), 3.59 (d, *J* = 13.4 Hz, 1H), 3.43 (s, 1H). **^{13}C NMR** (100 MHz, $CDCl_3$) δ 195.0, 162.7 (d, *J* = 246.2 Hz), 156.3, 147.5, 134.5 (d, *J* = 6.5 Hz), 130.8 (d, *J* = 7.9 Hz), 127.6 (d, *J* = 2.9 Hz), 126.3, 125.5, 123.8, 114.3 (d, *J* = 21.4 Hz), 113.4 (d, *J* = 21.1 Hz), 108.5, 57.3, 55.5, 50.6, 44.0. ^{19}F NMR (377 MHz, $CDCl_3$) δ -112.9. **HPLC conditions**: Daicel Chiralpak OD-H column (hexane/ isopropanol = 90/ 10, flow rate 1.0 mL/min, 254 nm); t_R = 9.688 (major), t_R = 11.403 (minor), 96% *ee*. HRMS (ESI): m/z: Calcd for $(C_{17}H_{14}FNO_2+H)^+$ 284.1081, found 284.1079. $[\alpha]_D^{20}$ = +383.798 (c = 1.612, $CHCl_3$).

(5*S*,11*S*)-2,8-dimethoxy-6,11-dihydro-12*H*-5,11-methanodibenzo[*b*,*f*]azocin-12-one (2aa)

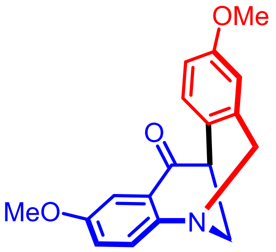

2aa

Following the general procedure, preparative thin layer chromatography on a silica gel (petroleum ether: ethyl acetate =4: 1) to give the product **2aa** (82.2 mg, 93% yield) as yellow oil. **^{1}H NMR** (400 MHz, $CDCl_3$) δ 7.34 – 7.29 (m, 1H), 7.19 (d, *J* = 8.5 Hz, 1H), 7.12 (d, *J* = 8.8 Hz, 1H), 7.02 (dd, *J* = 8.8, 2.7 Hz, 1H), 6.72 (dd, *J* = 8.4, 1.7 Hz, 1H), 6.46 (s, 1H), 4.71 (d, *J* = 16.9 Hz, 1H), 4.16 (d, *J* = 17.0 Hz, 1H), 3.99 (d, *J* = 13.4 Hz, 1H), 3.73 (s, 3H), 3.69 (s, 3H), 3.60 (dd, *J* = 13.4, 1.4 Hz, 1H), 3.39 (s, 1H). **^{13}C NMR** (100 MHz, $CDCl_3$) δ 195.4, 159.7, 156.2, 147.6, 133.5, 130.2, 126.2, 125.7, 123.9, 123.5, 113.2, 111.6, 108.5, 57.5, 55.4, 55.1, 50.8, 43.9. **HPLC conditions**: Daicel Chiralpak OD-H column (hexane/ isopropanol = 90/ 10, flow rate 1.0 mL/min, 254 nm); t_R = 11.588 (major), t_R = 13.213 (minor), 94% *ee*. HRMS (ESI): m/z: Calcd for $(C_{18}H_{17}NO_3+Na)^+$ 318.1101, found 318.1097. $[\alpha]_D^{20}$ = +491.564 (c = 1.644, $CHCl_3$).

(7*S*,14*S*)-8,14-dihydro-15*H*-7,14-methano[1,3]dioxolo[4',5':4,5]benzo[1,2-*f*]naphtho[2,1-*b*]azocin-15-one (2ab)

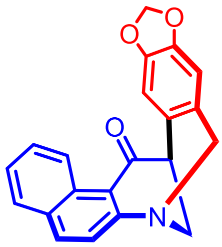

2ab

Following the general procedure, preparative thin layer chromatography on a silica gel (petroleum ether: ethyl acetate =4: 1) to give the product **2ab** (63 mg, 64% yield) as a yellow solid. m.p. > 250 °C. **^{1}H NMR** (400 MHz, $CDCl_3$) δ 9.40 (d, *J* = 8.7 Hz, 1H), 7.87 (d, *J* = 8.8 Hz, 1H), 7.69 (d, *J* = 8.0 Hz, 1H), 7.61 – 7.56 (m, 1H), 7.42 – 7.37 (m, 1H), 7.28 (d, *J* = 8.8 Hz, 1H), 6.81 (s, 1H), 6.38 (s, 1H), 5.87 (s, 1H), 5.77 (s, 1H), 4.76 (d, *J* = 16.6 Hz, 1H), 4.38 (d, *J* = 16.6 Hz, 1H), 4.14 (d, *J* = 13.1 Hz, 1H), 3.66 (d, *J* = 13.0 Hz, 1H), 3.36 (s, 1H). **^{13}C NMR** (100 MHz, $CDCl_3$) δ 197.3, 156.1, 147.9, 146.7, 136.0, 131.9, 131.0, 129.2, 128.2, 126.4, 126.0, 125.2, 124.9, 123.8, 117.1, 109.0, 106.4, 100.9, 56.9, 50.1, 46.8. **HPLC conditions**: Daicel Chiralpak OD-H column (hexane/ isopropanol = 90/ 10, flow rate 1.0 mL/min, 254 nm); t_R = 14.213 (major), t_R = 16.509 (minor), 87% *ee*. HRMS (ESI): m/z: Calcd for $(C_{21}H_{15}NO_3+Na)^+$ 352.0944, found 352.0935. $[\alpha]_D^{20}$ = +822.615 (c = 1.34, $CHCl_3$).

(6*S*,12*S*)-8,10-dimethyl-5,12-dihydro-11*H*-6,12-methano[1,3]dioxolo[4',5':4,5]benzo[1,2-*f*]benzo[*b*]azocin-11-one (2ac)

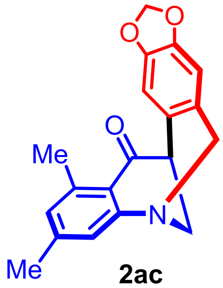

2ac

Following the general procedure, preparative thin layer chromatography on a silica gel (petroleum ether: ethyl acetate =4: 1) to give the product **2ac** (80.1 mg, 88% yield) as a yellow solid. m.p. 132.5-133.2 °C. **^{1}H NMR** (400 MHz, $CDCl_3$) δ 6.87 (s, 1H), 6.75 (s, 1H), 6.68 (s, 1H), 6.38 (s, 1H), 5.87 (s, 1H), 5.81 (s, 1H), 4.68 (d, *J* = 16.7 Hz, 1H), 4.19 (d, *J* = 16.7 Hz, 1H), 3.93 (d, *J* = 13.3 Hz, 1H), 3.54 (d, *J* = 13.2 Hz, 1H), 3.26 (s, 1H), 2.55 (s, 3H), 2.28 (s, 3H). **^{13}C NMR** (100 MHz, $CDCl_3$) δ 196.7, 155.6, 147.7, 146.6, 144.5, 142.4, 129.0, 125.8, 125.5, 123.5, 121.0, 108.9, 106.4, 100.8, 58.2, 50.0, 46.6, 22.9, 21.5. **HPLC conditions**: Daicel Chiralpak AD-H column (hexane/ isopropanol = 90/ 10, flow rate 1.0 mL/min, 254 nm); t_R = 9.566 (minor), t_R = 11.598 (major), 94% *ee*. HRMS (ESI): m/z: Calcd for $(C_{19}H_{17}NO_3+Na)^+$ 330.1101, found 330.1098. $[\alpha]_D^{20}$ = +314.341 (c = 1.25, $CHCl_3$).

11. X-ray single crystal data for compounds 2a

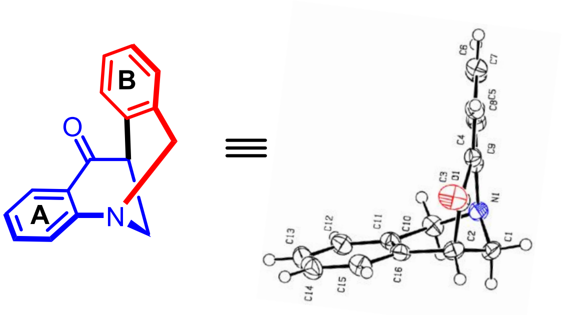

(*S*,*S*)-2a CCDC 2151213

The single-crystal of **2a** was grown from the mixed solution of dichloromethane and hexane (V/V = 1:3).

Table 1. Crystal data and structure refinement for ga_210325b_a.

Identification code	ga_210325b_a
Empirical formula	$C_{16}H_{13}NO$
Formula weight	235.27
Temperature	173(2) K
Wavelength	1.34138 Å
Crystal system	Monoclinic
Space group	$P2_1$
Unit cell dimensions	a = 9.7345(3) Å
	b = 12.3631(4) Å
	c = 10.0830(3) Å
Volume	1170.39(6) Å^3
Z	4
Density (calculated)	1.335 Mg/m^3
Absorption coefficient	0.419 mm^{-1}
F(000)	496
Crystal size	0.230 x 0.160 x 0.050 mm^3
Theta range for data collection	3.955 to 59.291°.
Index ranges	-12<=h<=12, -15<=k<=15, -12<=l<=12
Reflections collected	15318
Independent reflections	5140 [R(int) = 0.0362]
Completeness to theta = 53.594°	99.9 %
Absorption correction	Semi-empirical from equivalents
Max. and min. transmission	0.752 and 0.681
Refinement method	Full-matrix least-squares on F^2
Data / restraints / parameters	5140 / 1 / 325

Goodness-of-fit on F^2	1.062
Final R indices [I>2sigma(I)]	R1 = 0.0426, wR2 = 0.1124
R indices (all data)	R1 = 0.0449, wR2 = 0.1145
Absolute structure parameter	0.03(14)
Extinction coefficient	n/a
Largest diff. peak and hole	0.284 and -0.163 e.Å^{-3}

12. DFT Calculations

All the density functional theory (DFT) calculations were performed using Gaussian 09 program.[5] Geometry optimizations were performed with the M06-L functional[6] using a combined basis set (Lanl2DZ[7] for Palladium and 6-31G(d) basis[8] for the other atoms). Harmonic frequency calculations were performed for each stationary point to ensure that it is either an energy minimum (no imaginary frequency) or a transition state (only one imaginary frequency). For each transition state, intrinsic reaction coordinate (IRC) analysis was performed to ensure that it connects the correct reactant and product. The single-point energy calculations were further performed with the M06-L functional and a combined basis set (Lanl2DZ for Palladium and the 6-311+G(d,p) basis set[9] for all other atoms), using a self-consistent reaction field (SCRF) method called IEFPCM[10] in order to obtain energies in solution. The single-point energies corrected by the thermal correction to Gibbs free energies (TCG, obtained from frequency calculations) were used as the Gibbs free energies reported in this work, corresponding to the reference state of 1 mol/L, 298.15 K. The independent gradient model (IGM) was conducted with Multiwfn and VMD.[11] The 3-D images of the calculated structures were prepared using CYLview.[12]

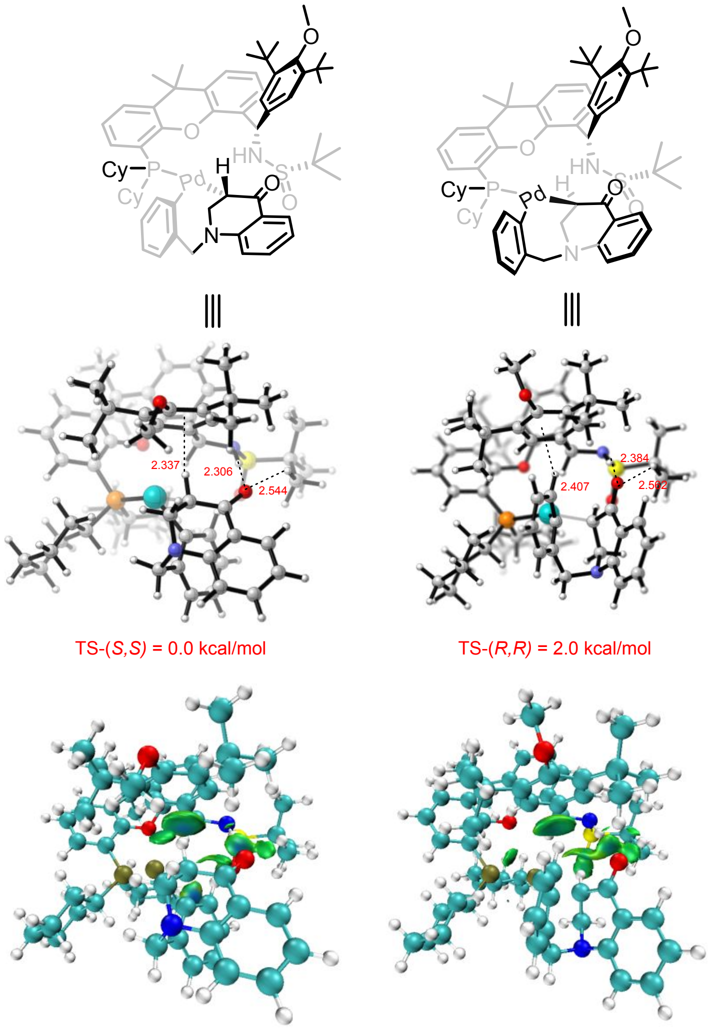

Figure S1. Optimized structures and IGM analysis of the transition states TS-(*S,S*) and TS-(*R,R*) for forming (*S,S*)-**2a** and (*R,R*)-**2a**. For IGM analysis, blue, attraction; green, weak interaction; red,

steric effect.

We have conducted DFT calculations on the C-C reductive elimination to understand the role of the ligand on enantioselectivity. The calculation is performed at the M06-L/ Lanl2DZ-6-311+G(d,p)// M06-L / Lanl2DZ -6-31G(d) level of theory. The solvent effect (acetonitrile) was also taken into account by using IEFPCM method. Reductive elimination from the Pd(II) intermediate could deliver the arylation product with the transition state (TS-(*S,S*) and TS-(*R,R*)) at 0 and 2.0 kcal/mol respectively. the IGM analysis showed that NH··O hydrogen bond between the NH of the ligand and the carbonyl group of the substrate play a significant role on lowering the energy of the transition state.

Table S7. Energy data (hartrees).

Geometry	$E_{M06\text{-}L/6\text{-}31G(d)}$	TCG	$E_{M06\text{-}L/6\text{-}311+G(d,p)}$	TCG+$E_{M06\text{-}L/6\text{-}311+G(d,p)}$	TCG+ $G_{(corr\text{-}B3\text{-}LYP)}$	Imaginary Frequency
TS-(*S*,*S*)	-3725.81804080	1.176358	-3726.62755614	-3725.451198	0	-293.41
TS-(*R*,*R*)	-3725.81445038	1.176145	-3726.62420390	-3725.448059	2.0	-270.56

TS-(*S*,*S*)

C	-3.26615200	5.04140700	11.08086000
C	-2.54742800	6.26674300	11.65170400
C	-2.34923400	7.32017700	10.56536700
C	-1.58829800	6.75275600	9.37146900
C	-2.26180400	5.49664100	8.82918500
C	-2.45216000	4.45553600	9.92572600
C	-5.54851700	4.41084500	12.72948700
C	-5.71027800	5.89410400	13.07986400
C	-7.18534200	6.26333900	13.22153300
C	-7.89959600	5.36407900	14.22190800
C	-7.76912300	3.90036700	13.81934600
C	-6.30601900	3.49179400	13.69546100
C	-0.45720500	4.29652700	14.63672200
C	-0.95779900	4.75560400	15.85586700
C	-2.33047400	4.86500600	16.06292800
C	-3.22294100	4.57568900	15.03462400
C	-2.77178100	4.10768400	13.79370000
C	-1.38580600	3.91603900	13.66479700
C	0.24680700	2.64669100	12.53380300
C	0.35485200	1.50882800	11.72740900
C	1.57354600	0.82819300	11.72366800
C	2.62749100	1.25335400	12.52381700
C	2.46550800	2.34885500	13.36906300
C	1.26827900	3.06605300	13.38901100
C	1.01643200	4.29689500	14.24668000
C	1.27270300	5.54702900	13.37714800
C	1.93172900	4.34915100	15.46458400
C	-0.87296200	0.91829600	11.06844900
C	-1.46394800	-0.11991600	12.01412200
C	-1.43967900	-1.47934100	11.72972200
C	-2.08475500	-2.41566500	12.54317900
C	-2.72455500	-1.93818900	13.71041100
C	-2.61392400	-0.59314400	14.12760100
C	-2.02594400	0.29630000	13.21876100
C	-3.03659800	-0.00949100	15.49182600
C	-4.24732400	0.91502500	15.30962600
C	-1.86908700	0.83936000	16.03734200
C	-3.34033000	-1.03799800	16.58900600
C	-2.03676800	-3.90816000	12.17955800
C	-1.33683200	-4.68656600	13.30345500
C	-1.24152400	-4.14382500	10.88970700
C	-3.43676900	-4.49777300	11.95133200

C	-4.85284800	-2.68711600	14.32303600
C	0.50312700	-0.07357500	7.37228900
C	-0.79295900	-0.75822700	6.97708100
C	1.11083200	0.65943800	6.18250600
C	1.50228800	-1.02668200	8.00557700
N	-0.57337200	0.27687300	9.78083000
O	-0.95030400	3.34713800	12.48621300
O	-0.86816800	2.19427800	7.93435500
O	-3.44928500	-2.84417100	14.46430900
S	0.14215200	1.29524000	8.60938000
H	-4.22742900	5.38490200	10.65452300
H	-1.56438600	5.95289400	12.03574300
H	-3.08690100	6.68922700	12.51019600
H	-3.33643500	7.68123200	10.23268400
H	-1.82385400	8.19184200	10.97882600
H	-1.48776900	7.51261300	8.58461300
H	-0.56394800	6.49831900	9.69065900
H	-1.67653300	5.06623800	8.00567400
H	-3.24641700	5.76195600	8.40819800
H	-2.93967000	3.55916000	9.52750600
H	-1.46873000	4.13109100	10.29535300
H	-6.02978700	4.27381500	11.74182500
H	-5.18398900	6.13354800	14.01507300
H	-5.25071600	6.51705000	12.30042700
H	-7.27746500	7.31870100	13.51031000
H	-7.67454500	6.16625800	12.23881200
H	-8.95689400	5.64793100	14.30566100
H	-7.45768700	5.50689900	15.22180500
H	-8.26987300	3.74798400	12.84911200
H	-8.28094000	3.24902900	14.54016500
H	-6.21387700	2.45116000	13.34224300
H	-5.84166600	3.51293100	14.69351800
H	-0.27239300	5.04206600	16.65051600
H	-2.70819500	5.21532700	17.02099000
H	-4.28691300	4.71484700	15.20677100
H	1.66891200	-0.06451700	11.10607800
H	3.57252300	0.71416100	12.51159700
H	3.28511800	2.64905000	14.01821000
H	0.66142700	5.53501200	12.46557800
H	2.32638100	5.58856500	13.07434700
H	1.03494700	6.46105300	13.93582500
H	1.74605000	5.25646900	16.04910800
H	2.98323000	4.38365600	15.16179200
H	1.79011200	3.48149400	16.12034600

H	-1.62987200	1.71496300	10.94467800
H	-0.94634000	-1.80528300	10.82150000
H	-1.98974000	1.35531400	13.47167900
H	-4.02942300	1.71367300	14.58661900
H	-4.51429600	1.39320800	16.26294500
H	-5.13255100	0.37453400	14.94661900
H	-0.96401600	0.23196500	16.16527100
H	-2.14193800	1.24930400	17.01842900
H	-1.61721800	1.69125000	15.39538400
H	-4.27617300	-1.58110400	16.44044800
H	-3.43041600	-0.50405600	17.54393800
H	-2.53449700	-1.77274800	16.69228200
H	-0.30712100	-4.33303300	13.44269800
H	-1.29160100	-5.75422700	13.05023200
H	-1.86856500	-4.58411200	14.25390100
H	-1.70632200	-3.65296500	10.02414300
H	-1.20620500	-5.22019700	10.68287800
H	-0.20559100	-3.79078000	10.96784300
H	-3.98424300	-4.63393000	12.88644600
H	-3.34728700	-5.48528600	11.48014200
H	-4.03807500	-3.86742700	11.28121200
H	-5.17692500	-1.66164500	14.54852700
H	-5.32027000	-3.37712700	15.03088000
H	-5.18056400	-2.93560400	13.30351800
H	-1.56863500	-0.01913700	6.73573300
H	-1.17459200	-1.41867800	7.76342700
H	-0.62549900	-1.37239000	6.08341100
H	1.37621800	-0.06539900	5.40365600
H	2.02376000	1.20120900	6.46115400
H	0.40098300	1.37581500	5.75488200
H	1.05923300	-1.57287200	8.84554600
H	2.39102800	-0.49515800	8.37051400
H	1.83755900	-1.75697200	7.25799600
H	-1.42800200	-0.17305900	9.42402300
C	-7.95140000	-2.27781600	7.25851900
C	-8.10874100	-1.55393400	8.43349900
C	-7.00279200	-1.25082700	9.23358400
C	-5.71228100	-1.64993500	8.80605200
C	-6.68471400	-2.71307200	6.85494400
H	-8.82593100	-2.51979600	6.65718700
H	-9.09676400	-1.23257600	8.76017300
H	-6.57022700	-3.28893700	5.93901600
C	-4.49442700	-1.18154700	9.51469900
C	-4.74792400	-0.42081100	10.75632800

H	-3.91325200	-0.61773700	11.45433000
C	-6.08886600	-0.76135300	11.36670200
H	-6.10875000	-1.83085300	11.63929600
N	-7.19855900	-0.53358200	10.44481600
O	-3.35777400	-1.38490700	9.06960500
C	-5.57805300	-2.39090900	7.62445300
H	-4.57372600	-2.68182200	7.31986100
C	-7.42329300	0.91710600	10.22976600
H	-8.41124200	1.05509400	9.77272400
H	-7.45620200	1.37558800	11.22841800
C	-6.35663000	1.50631800	9.34916000
C	-6.70329800	2.06558600	8.11518200
C	-4.99184200	1.32508800	9.65822100
C	-5.73833200	2.42996300	7.17828100
H	-7.76284000	2.19242200	7.88399900
C	-4.03439000	1.58247600	8.66338500
C	-4.39675900	2.16772900	7.45404700
H	-6.03480700	2.86961300	6.22770800
H	-2.98259000	1.36289700	8.84057800
H	-3.61631100	2.41188400	6.73277900
P	-3.84173300	3.76890500	12.33449600
Pd	-4.13567300	1.56467000	11.53860500
H	-6.27970000	-0.19526200	12.28794700

TS-(*R*,*R*)

C	7.33378000	-2.66726800	13.44743900
C	6.35311600	-1.61754500	12.91918100
C	7.04636300	-0.27273100	12.72515500
C	7.69341100	0.20464600	14.01999400
C	8.65812700	-0.84059600	14.56887400
C	7.97488400	-2.19099100	14.75415800
C	7.06733500	-5.09992100	11.88569700
C	6.77616500	-4.26425600	10.63380300
C	7.39426100	-4.91984400	9.40010900
C	6.93017100	-6.36137300	9.23430100
C	7.24460400	-7.18043400	10.48049600
C	6.62128900	-6.55688000	11.72410000
C	3.01704500	-3.02948900	14.90353700
C	2.15816600	-3.28493900	13.83422400
C	2.60681400	-3.98103000	12.71468400
C	3.94169000	-4.36185700	12.61593000
C	4.84282800	-4.12912800	13.66227500
C	4.31673600	-3.53803100	14.82396600
C	4.56253600	-3.36724500	17.16217900
C	5.21564800	-4.00572300	18.22275900

C	4.65820000	-3.88678400	19.49718700
C	3.46816800	-3.19584100	19.69194200
C	2.79676900	-2.64301800	18.60362800
C	3.33367000	-2.72035300	17.31810400
C	2.69226200	-2.11673200	16.07992800
C	3.36436000	-0.75308700	15.80749300
C	1.19296200	-1.90144300	16.24862900
C	6.34751700	-4.97333900	17.95482300
C	5.76746200	-6.37496400	17.84675600
C	6.02578000	-7.36756000	18.78711300
C	5.44040200	-8.63306900	18.70328700
C	4.52307500	-8.85816400	17.65083500
C	4.32494600	-7.92265900	16.61444700
C	4.95074800	-6.67953200	16.76372300
C	3.51861400	-8.13471700	15.31648200
C	4.43002700	-7.79491300	14.12329000
C	2.32197300	-7.17230700	15.30735900
C	3.01851900	-9.55846000	15.04375200
C	5.87854900	-9.75117500	19.66284700
C	4.72216800	-10.34394800	20.48036800
C	6.93071800	-9.25490400	20.66043000
C	6.52757300	-10.86718600	18.82872200
C	2.48797100	-9.85333100	18.15629600
C	9.26431200	-3.90517400	20.57627500
C	10.13067300	-5.10123200	20.22486300
C	10.12220400	-2.66695400	20.80754200
C	8.34266800	-4.17028000	21.75473600
O	5.16154300	-3.42336200	15.90980700
O	9.15000700	-3.25645100	17.93946900
O	3.80554100	-10.04352000	17.65827200
H	8.14782500	-2.77770000	12.70716900
H	5.53236900	-1.49692200	13.64408700
H	5.88171400	-1.95186800	11.98490400
H	7.81872000	-0.37305900	11.94499900
H	6.32640900	0.46952100	12.35518300
H	8.20960100	1.16110400	13.86264400
H	6.90507400	0.39583700	14.76626500
H	9.09038500	-0.50765900	15.52242900
H	9.50090900	-0.95631600	13.86796700
H	8.69293500	-2.94124100	15.11676000
H	7.20101700	-2.11168900	15.53328400
H	8.16901900	-5.13046600	11.98325500
H	5.69383700	-4.15256300	10.47792100
H	7.17667700	-3.24801200	10.75081000

H	7.15500000	-4.32889700	8.50619300
H	8.49167500	-4.90383100	9.49908000
H	7.39457700	-6.81352200	8.34790400
H	5.84232100	-6.37505200	9.05692100
H	8.33828200	-7.22887900	10.61575900
H	6.89929500	-8.21689800	10.36580900
H	6.89645300	-7.12685900	12.62471700
H	5.52403200	-6.61312400	11.64733100
H	1.13161500	-2.92636300	13.86652300
H	1.92381900	-4.18833600	11.89378300
H	4.28581300	-4.85098300	11.70872700
H	5.15845800	-4.37977300	20.33018400
H	3.04362000	-3.11077900	20.69006000
H	1.84347200	-2.14334500	18.76094900
H	4.45271500	-0.85784700	15.71395700
H	3.16080900	-0.05713500	16.63077700
H	2.98169900	-0.31372400	14.87751400
H	0.76334400	-1.44514400	15.35035700
H	0.98984800	-1.21237900	17.07497200
H	0.66295000	-2.84119900	16.44562600
H	6.78830000	-4.72382900	16.97059600
H	6.70287900	-7.14979200	19.60585300
H	4.80102500	-5.91716400	15.99911200
H	4.83950700	-6.78042600	14.17154300
H	3.85974200	-7.87223900	13.18699600
H	5.27885300	-8.48861100	14.06411900
H	1.62975900	-7.38190000	16.13386200
H	1.76181000	-7.26782100	14.36730200
H	2.64447800	-6.12544600	15.39784100
H	3.82693100	-10.29399400	15.11181500
H	2.62294200	-9.58825600	14.01978900
H	2.21076700	-9.88055900	15.70550300
H	4.14407800	-9.56104100	20.98839000
H	5.12578400	-11.00988400	21.25453100
H	4.04301900	-10.93338300	19.86056400
H	7.82232600	-8.85874000	20.15906800
H	7.24756600	-10.09433600	21.29131700
H	6.53532800	-8.47605300	21.32612700
H	5.82547200	-11.27564900	18.09408800
H	6.85595900	-11.68769000	19.48134500
H	7.41099200	-10.49033600	18.29499700
H	2.49930400	-9.55354700	19.21362900
H	1.96978200	-10.81096300	18.05409600
H	1.94712000	-9.08417400	17.58645100

H	10.63281500	-4.94933800	19.26015300
H	9.56247900	-6.03795400	20.17971000
H	10.90757600	-5.22718500	20.98910900
H	10.77892600	-2.83343900	21.66960800
H	9.50906400	-1.78201300	21.01983100
H	10.75089400	-2.45105800	19.93675700
H	7.75203900	-5.08051500	21.60657200
H	7.65035600	-3.33481300	21.92318900
H	8.94190800	-4.29205900	22.66605800
C	13.08793800	-10.00484400	15.98746500
C	12.78707400	-8.87419100	15.23872300
C	11.79992500	-7.98208700	15.66661900
C	11.07875100	-8.26610600	16.85192400
C	12.40587300	-10.26988900	17.17975900
H	13.87015300	-10.68109500	15.64670300
H	13.33053700	-8.65532100	14.32061200
H	12.65185600	-11.15268000	17.76591400
C	9.91678700	-7.43675100	17.25334200
C	10.87469300	-5.78928000	15.67491300
H	10.66389700	-4.93660700	15.01904200
O	9.23138900	-7.69296100	18.25174900
C	11.40842400	-9.40421200	17.60043200
H	10.83626300	-9.59966000	18.50673500
P	6.65511900	-4.41571300	13.56811100
Pd	7.91580600	-5.64425800	15.14591500
H	11.60300000	-5.44326300	16.42861000
C	10.74348200	-7.14721400	13.66179700
H	10.48711900	-6.18423100	13.19905200
H	11.38881800	-7.68801400	12.95823300
C	9.53670500	-7.97940700	13.99689700
C	8.64025900	-7.56767400	15.00228900
C	9.39683200	-9.26030300	13.45644300
C	7.72965500	-8.49040300	15.53641500
C	8.41224100	-10.13833100	13.90611600
H	10.09694400	-9.57593000	12.68028500
C	7.59136900	-9.75432000	14.96526100
H	7.11558800	-8.21136200	16.39151200
H	8.31886400	-11.12915800	13.46565300
H	6.84126600	-10.44074700	15.36053000
N	11.52656900	-6.82990200	14.88113800
C	9.62776300	-6.28790600	16.37023500
H	9.14881800	-5.48811000	16.96724100
N	7.37715600	-4.94311500	19.00211300
H	8.04482900	-5.70686900	18.82749700

S	8.19732300	-3.44340900	19.10102300

13. ^{1}H, ^{13}C, ^{19}F, ^{31}P NMR Spectra

^{1}H NMR (400 MHz, $CDCl_3$) of compound **1a:**

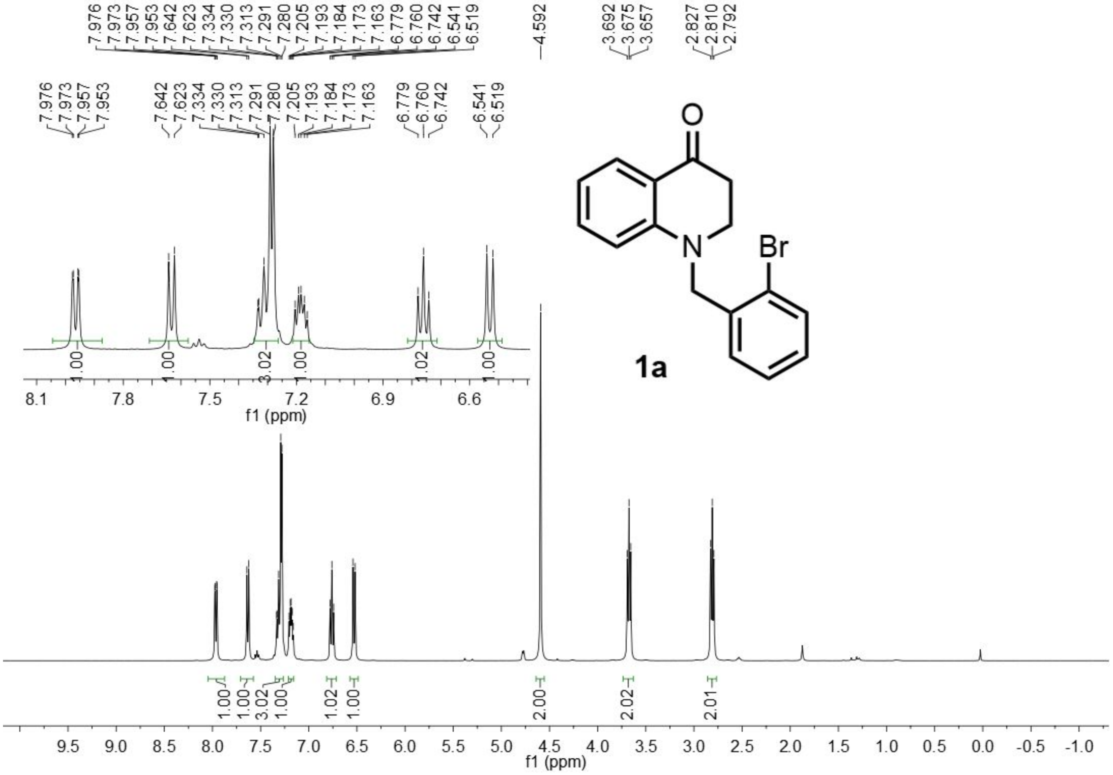

^{13}C NMR (100 MHz, $CDCl_3$) of compound **1a:**

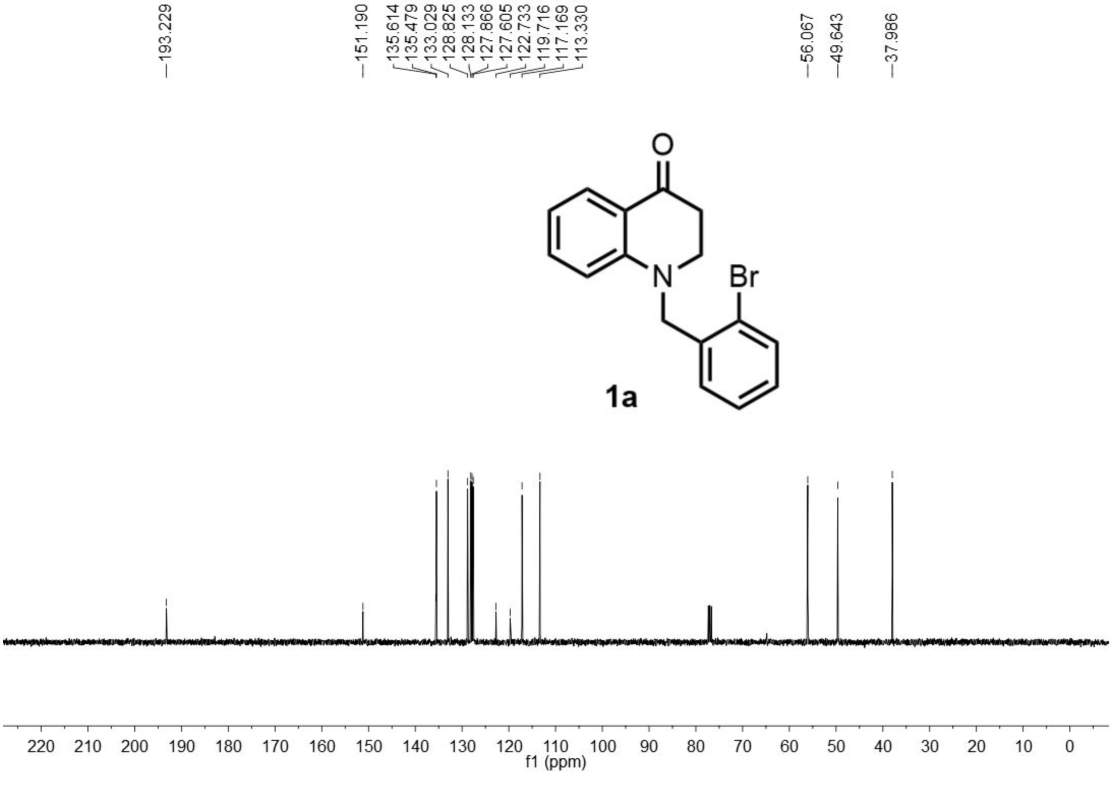

^{1}H NMR (400 MHz, $CDCl_3$) of compound **1b:**

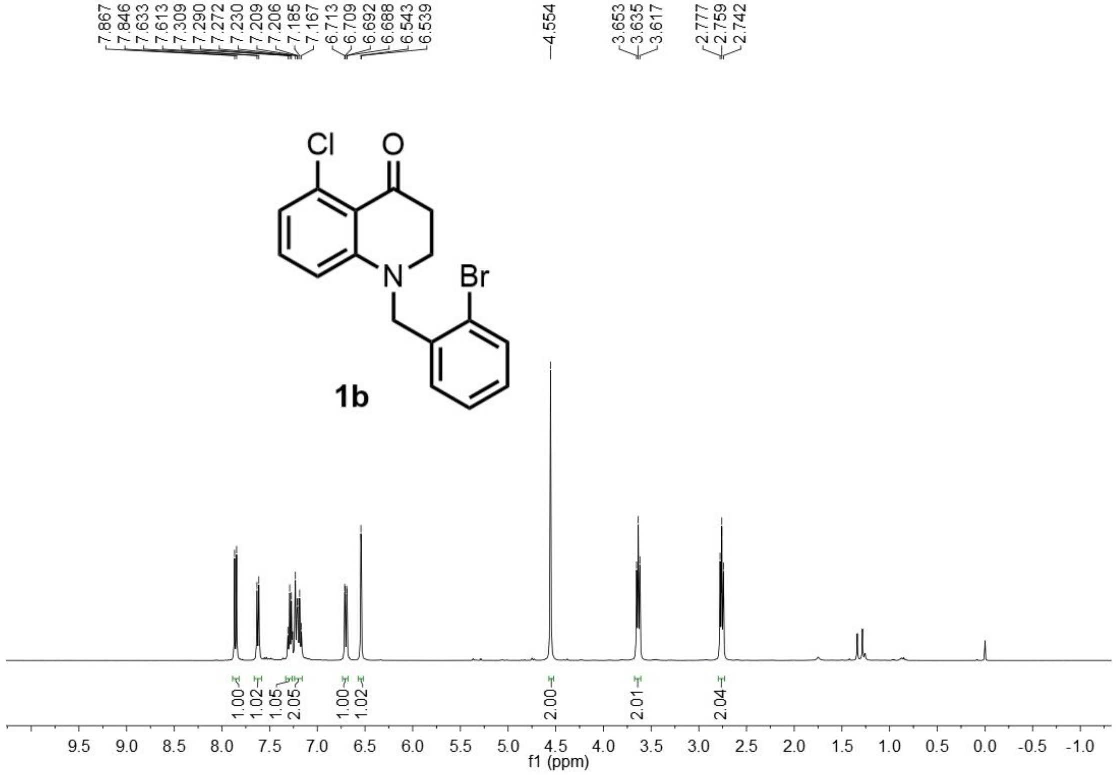

^{13}C NMR (100 MHz, $CDCl_3$) of compound **1b:**

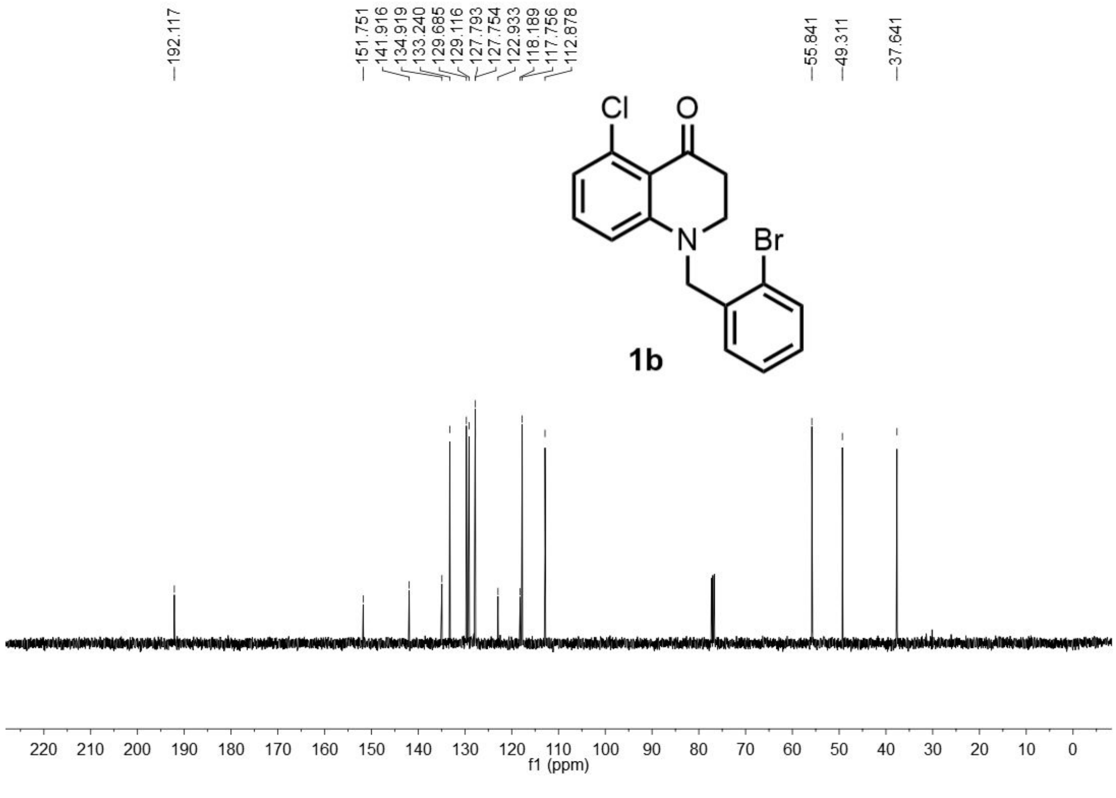

^{1}H NMR (400 MHz, $CDCl_3$) of compound **1c:**

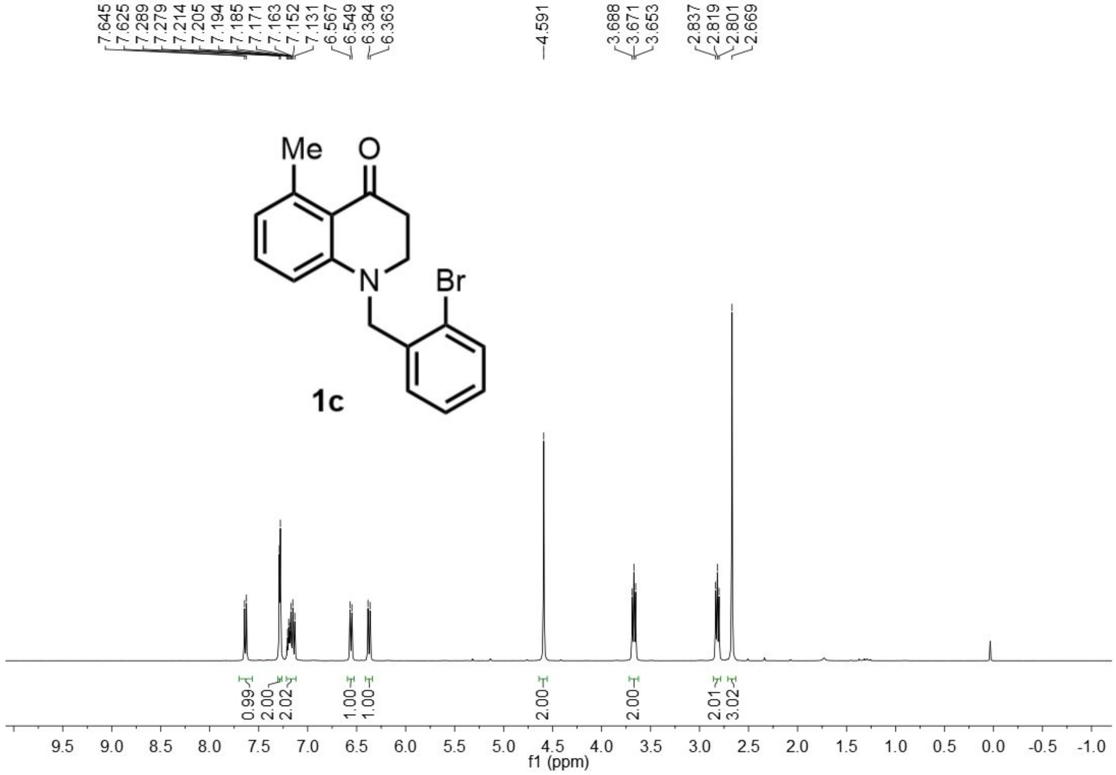

^{13}C NMR (100 MHz, $CDCl_3$) of compound **1c:**

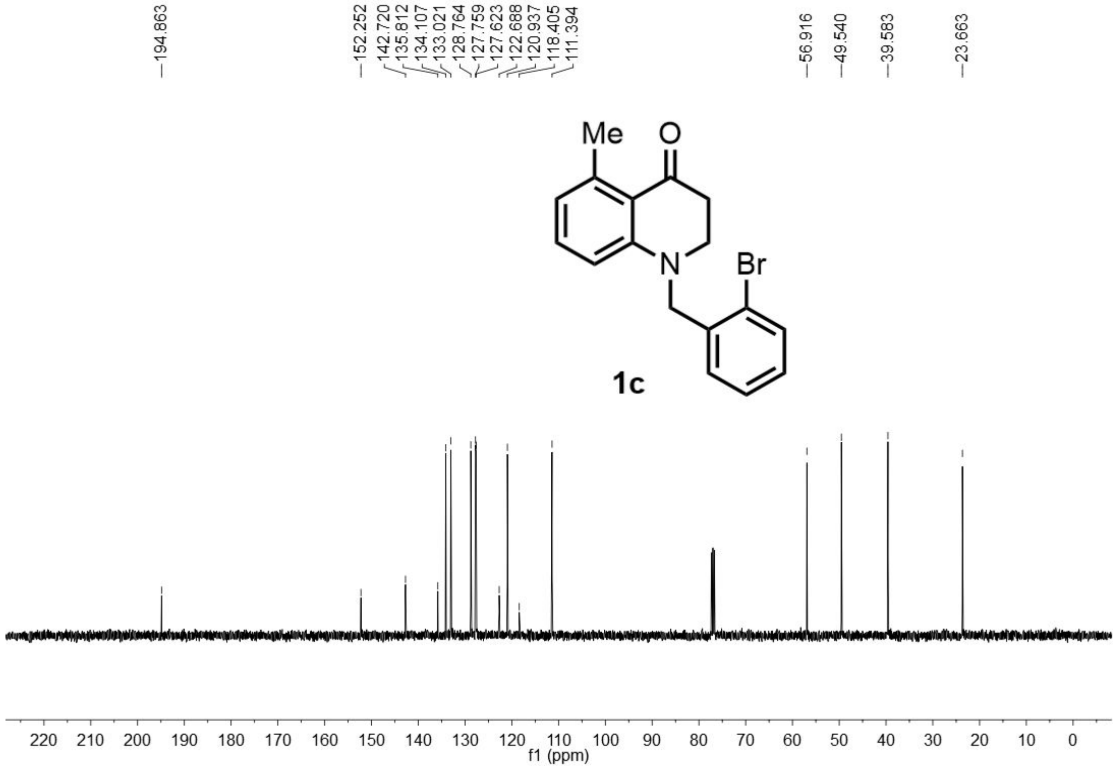

^{1}H NMR (400 MHz, $CDCl_3$) of compound **1d:**

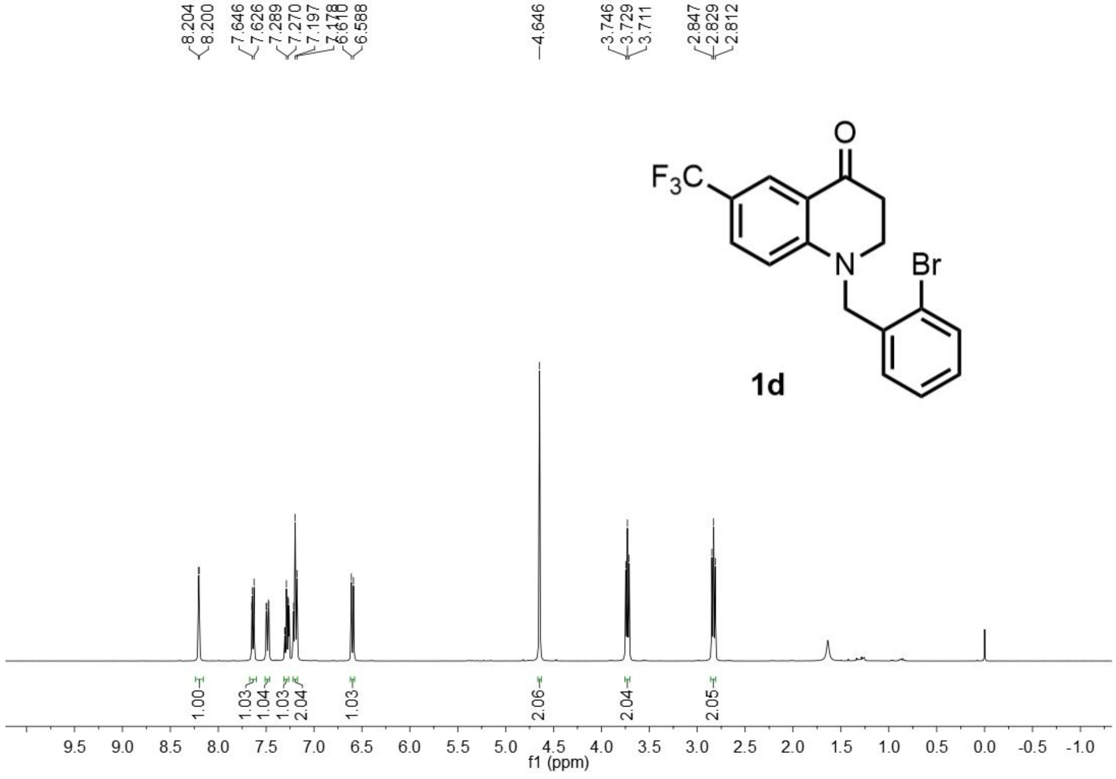

^{13}C NMR (100 MHz, $CDCl_3$) of compound **1d:**

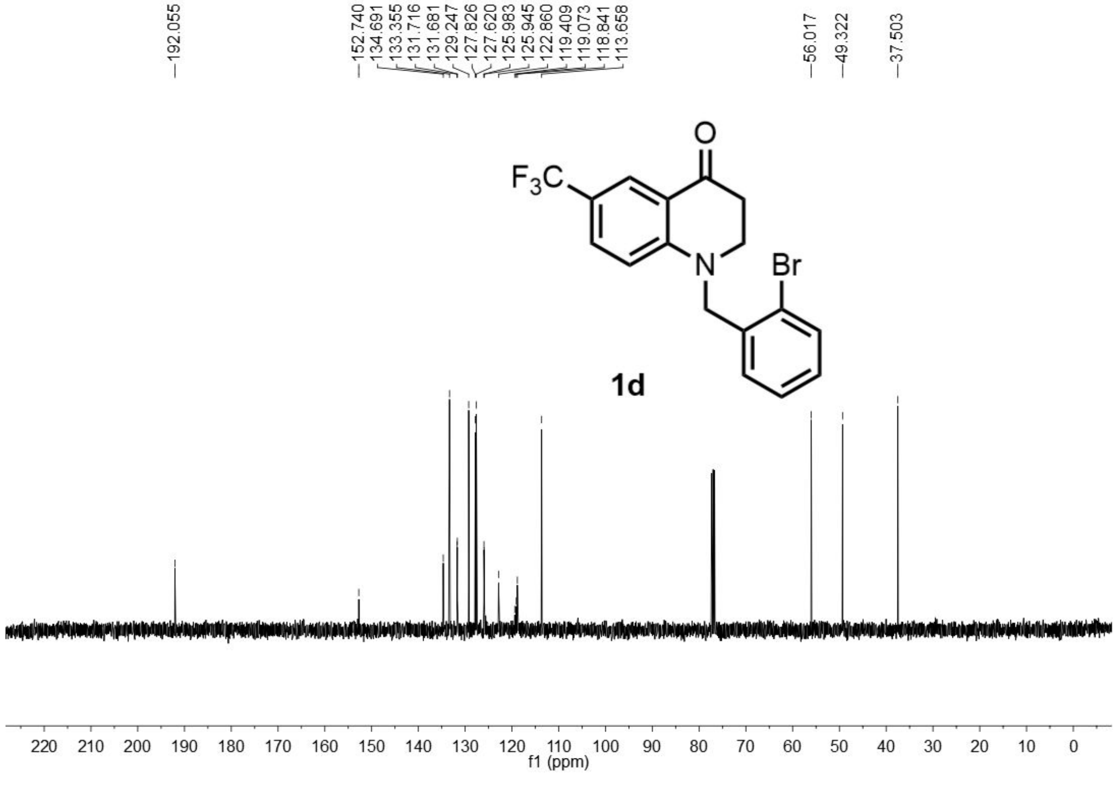

^{19}F NMR (377 MHz, $CDCl_3$) of compound **1d:**

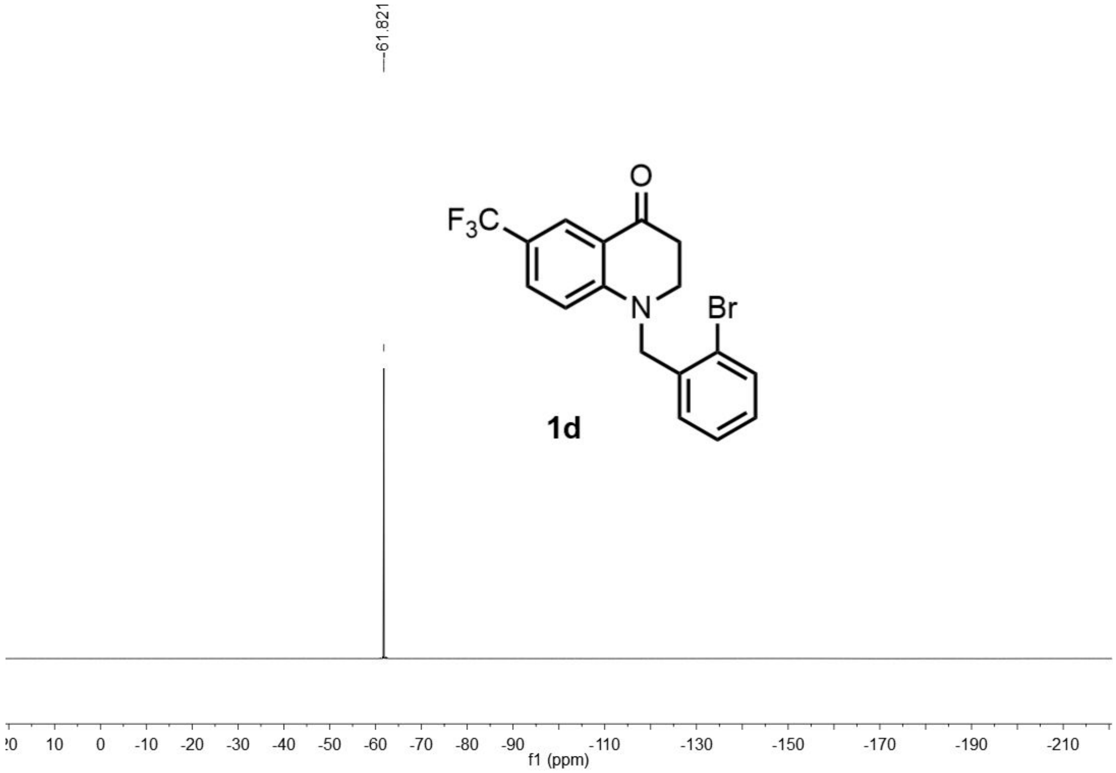

^{1}H NMR (400 MHz, $CDCl_3$) of compound **1e:**

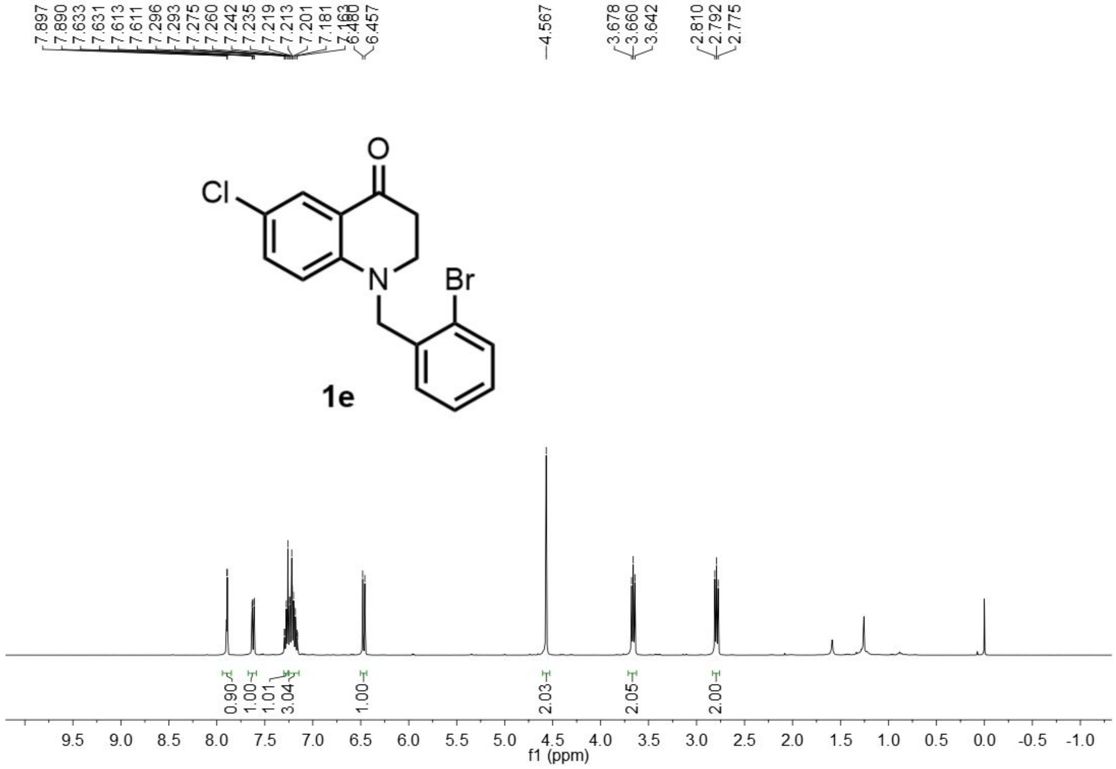

^{13}C NMR (100 MHz, $CDCl_3$) of compound **1e:**

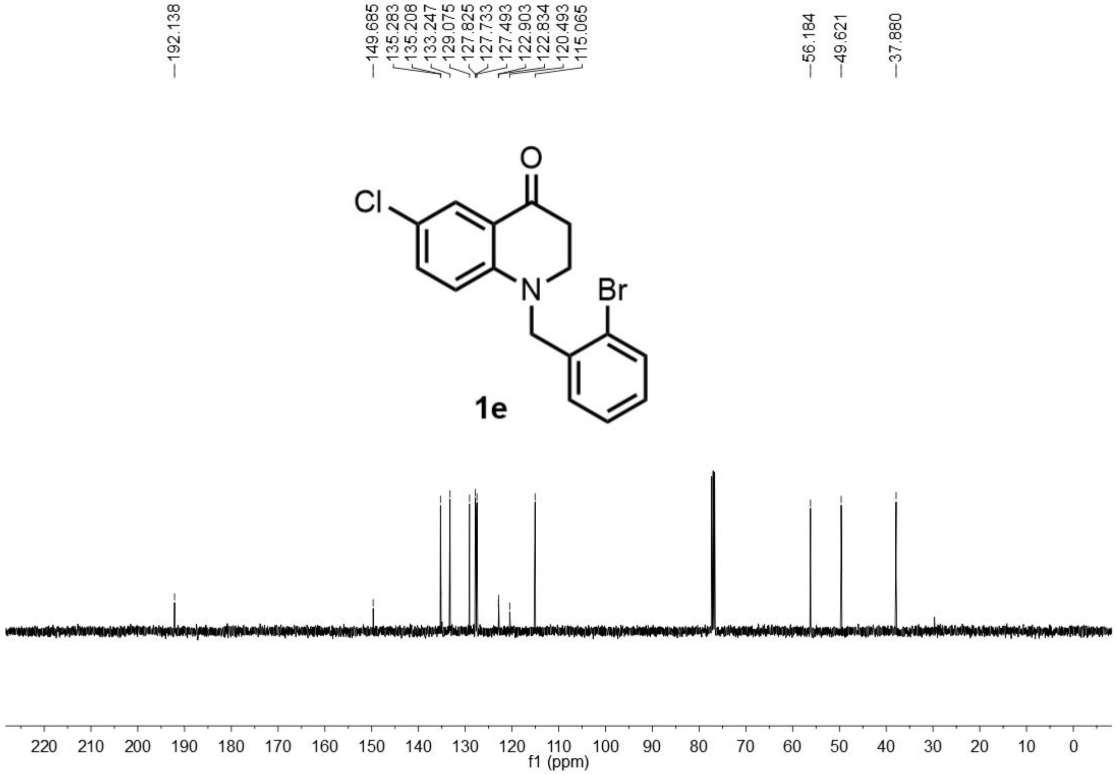

^{1}H NMR (400 MHz, $CDCl_3$) of compound **1f:**

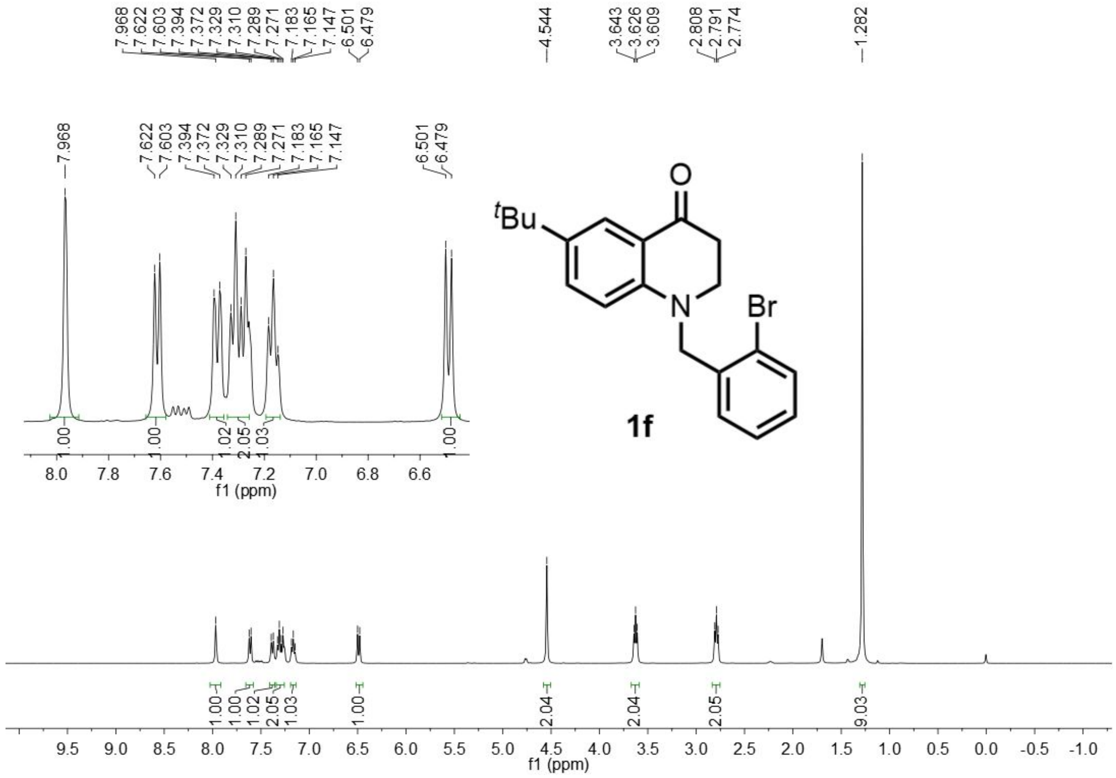

^{13}C NMR (100 MHz, $CDCl_3$) of compound **1f:**

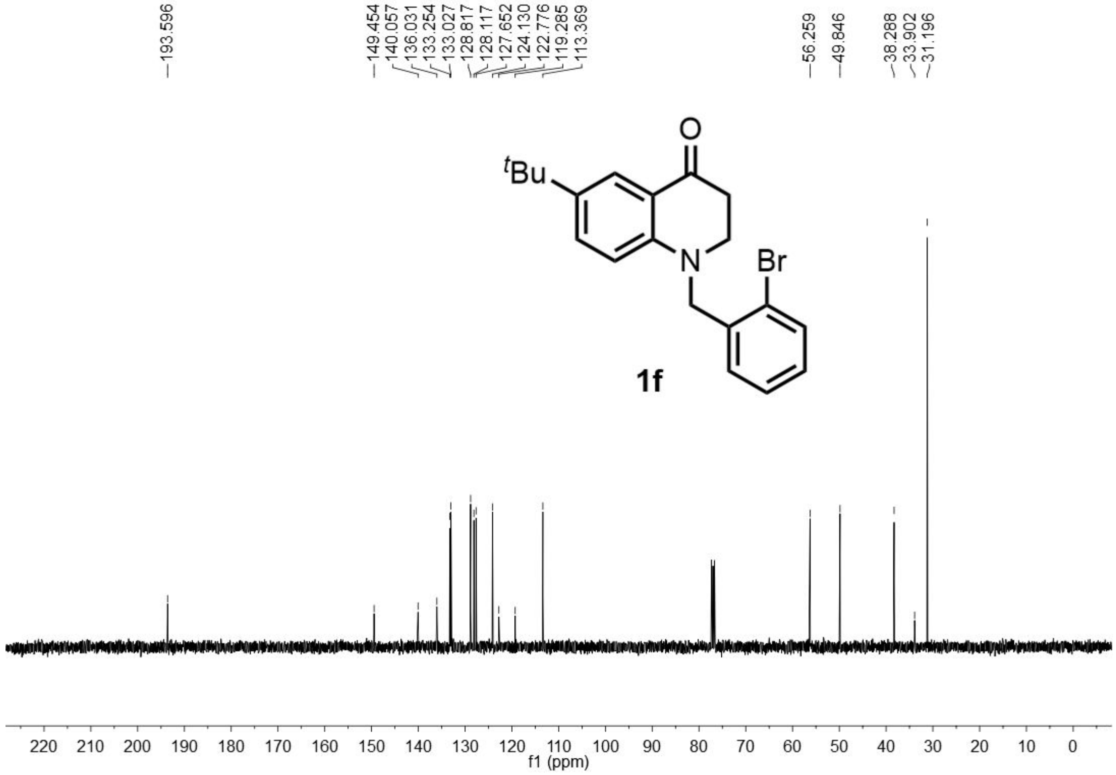

^{1}H NMR (400 MHz, $CDCl_3$) of compound **1g:**

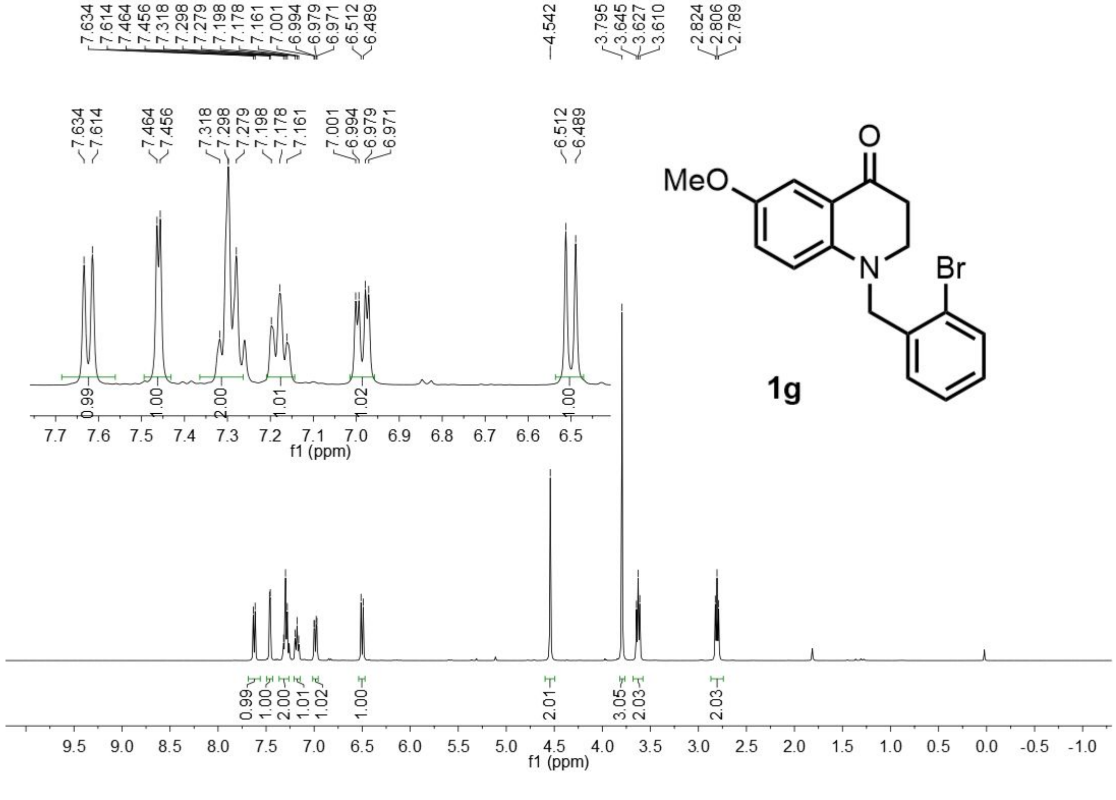

^{13}C NMR (100 MHz, $CDCl_3$) of compound **1g:**

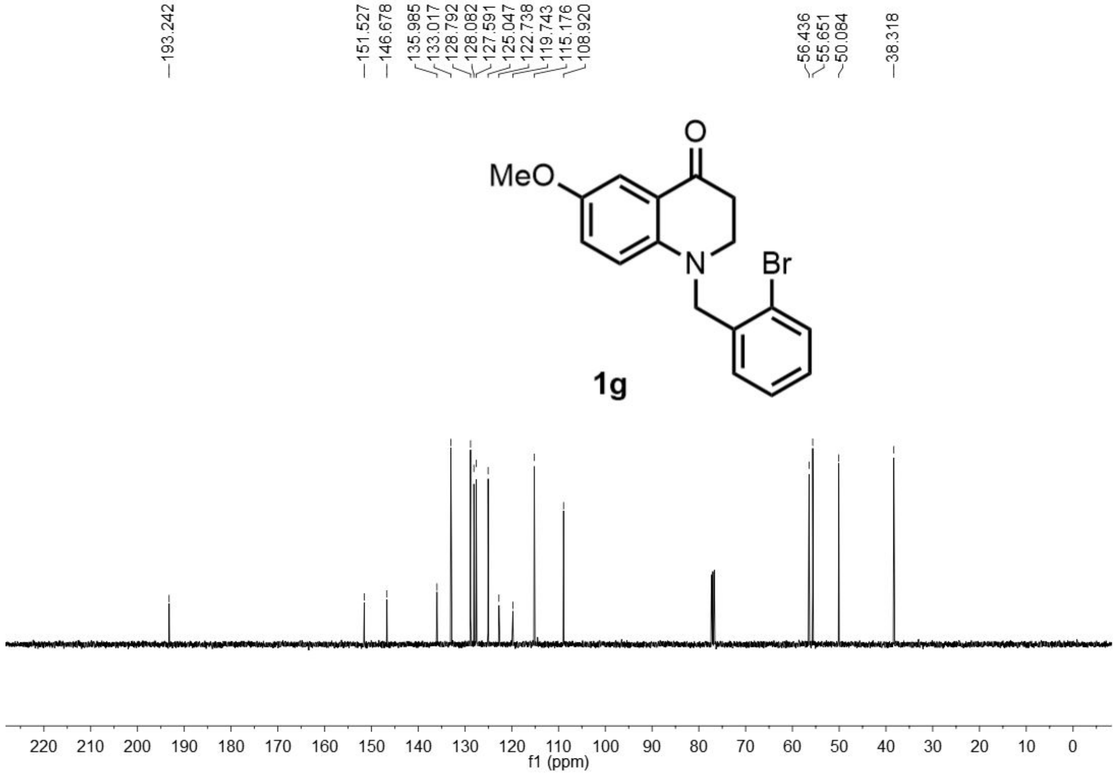

^{1}H NMR (400 MHz, $CDCl_3$) of compound **1h:**

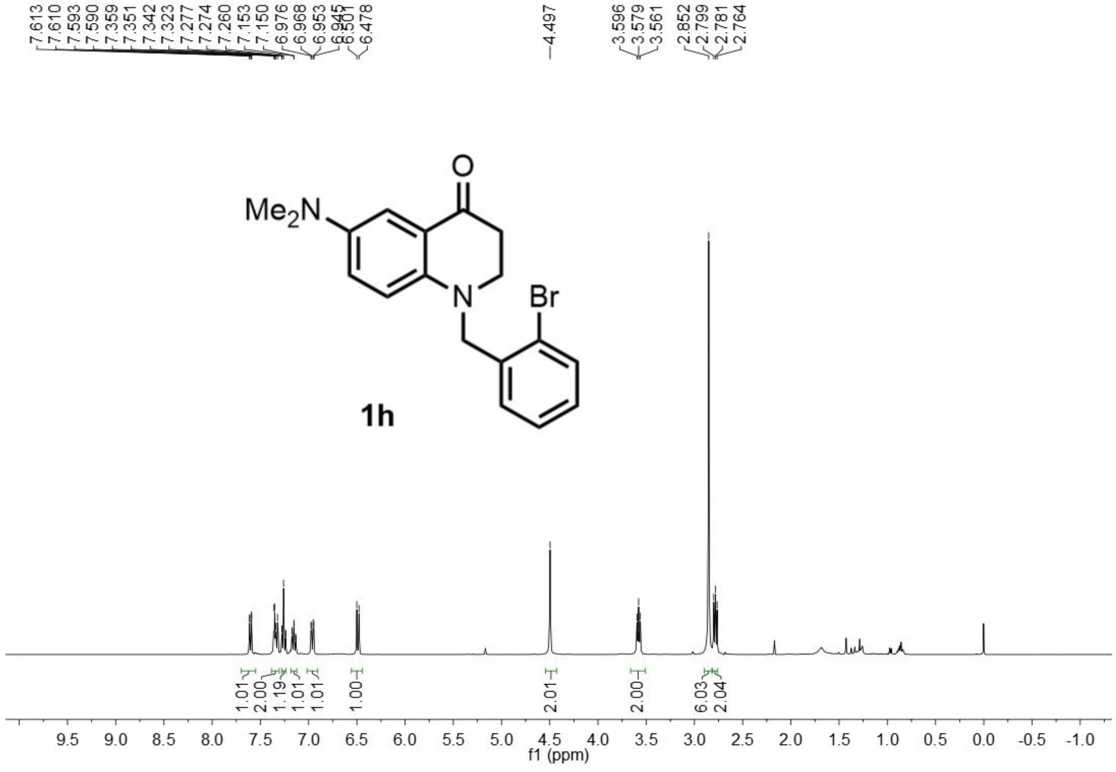

^{13}C NMR (100 MHz, $CDCl_3$) of compound **1h:**

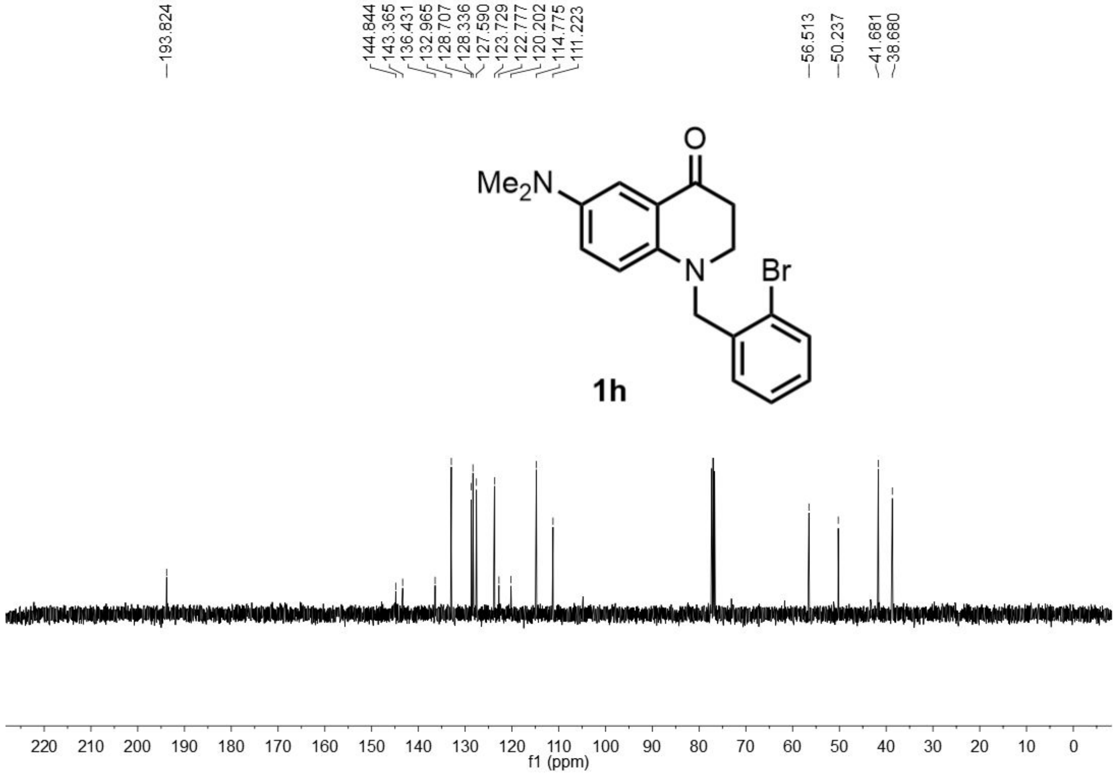

^{1}H NMR (400 MHz, $CDCl_3$) of compound **1i:**

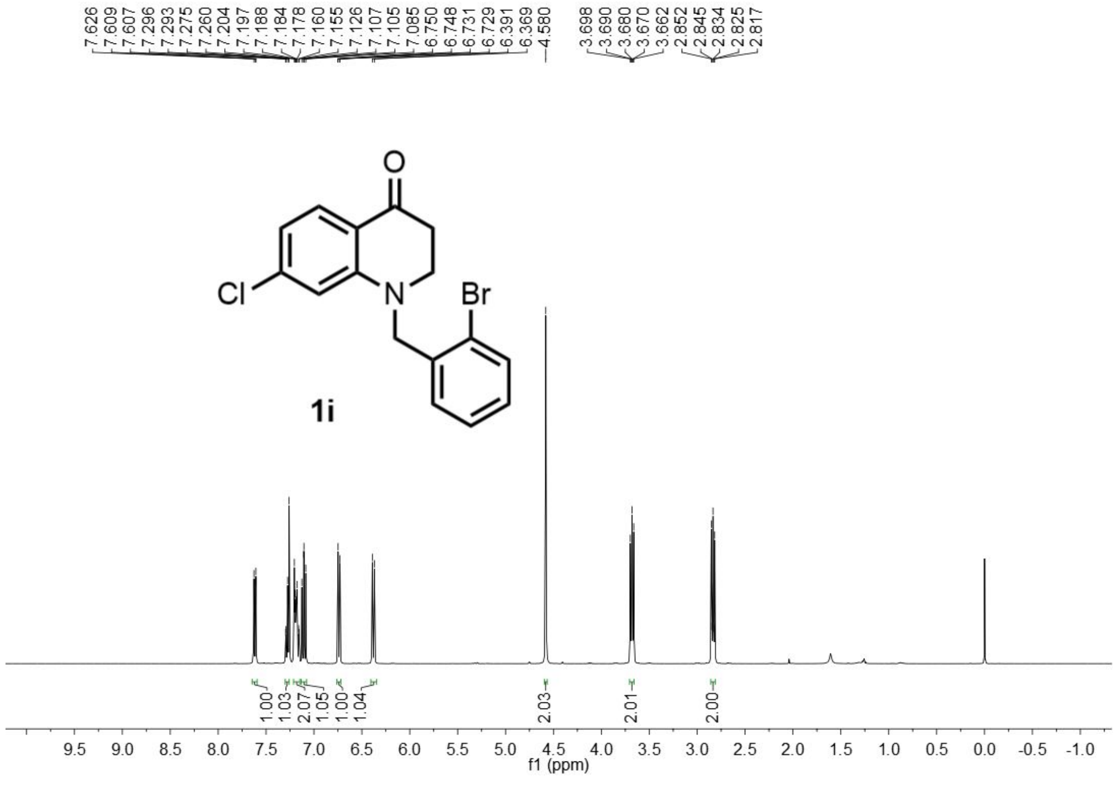

^{13}C NMR (100 MHz, $CDCl_3$) of compound **1i:**

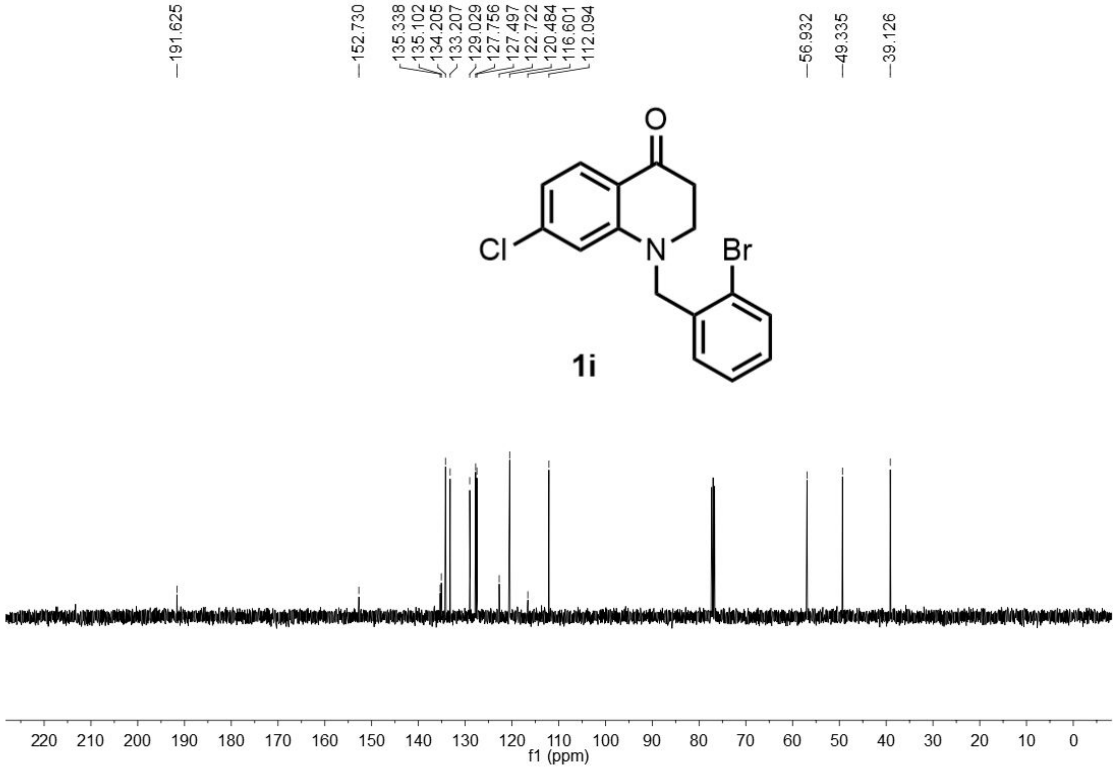

^{1}H NMR (400 MHz, $CDCl_3$) of compound **1j:**

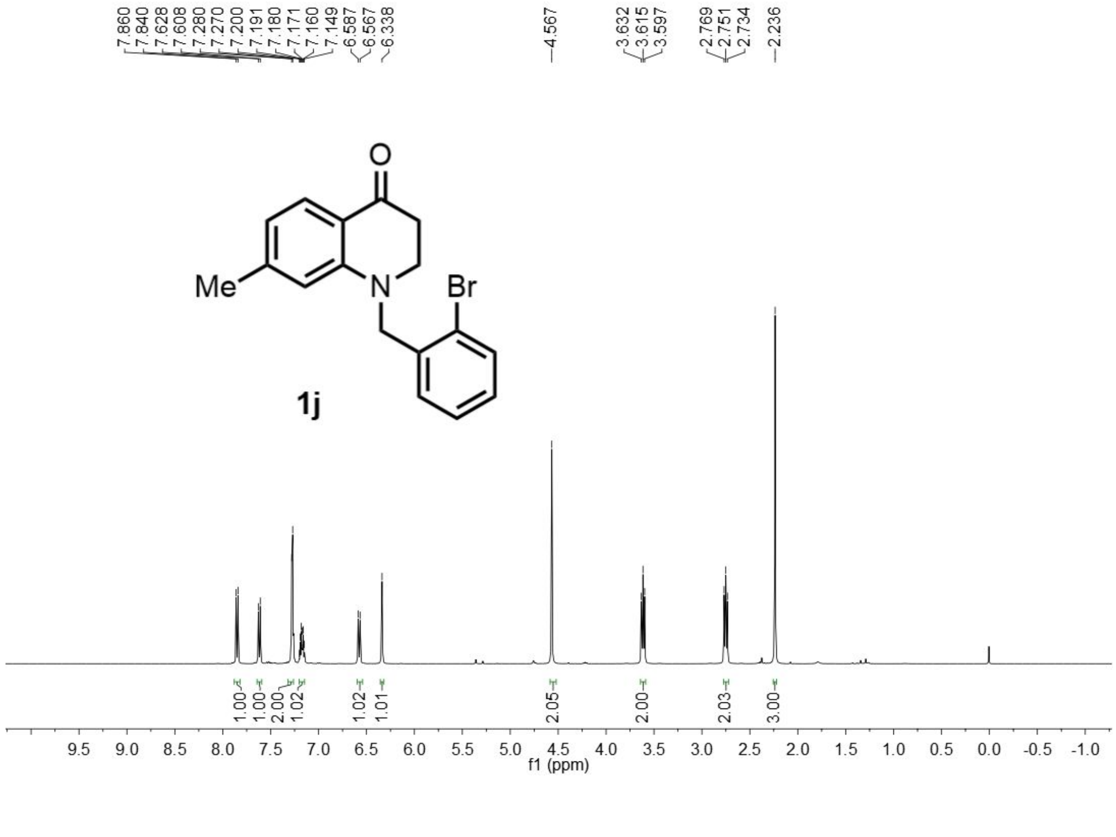

^{13}C NMR (100 MHz, $CDCl_3$) of compound **1j:**

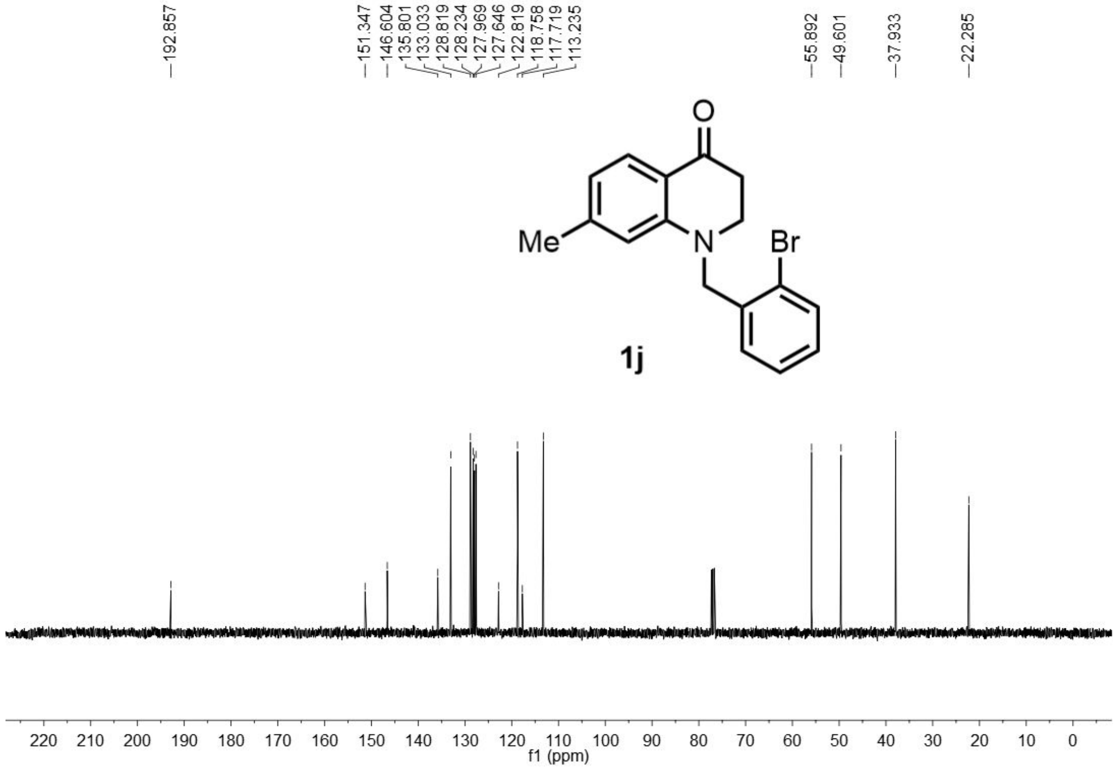

^{1}H NMR (400 MHz, $CDCl_3$) of compound **1k:**

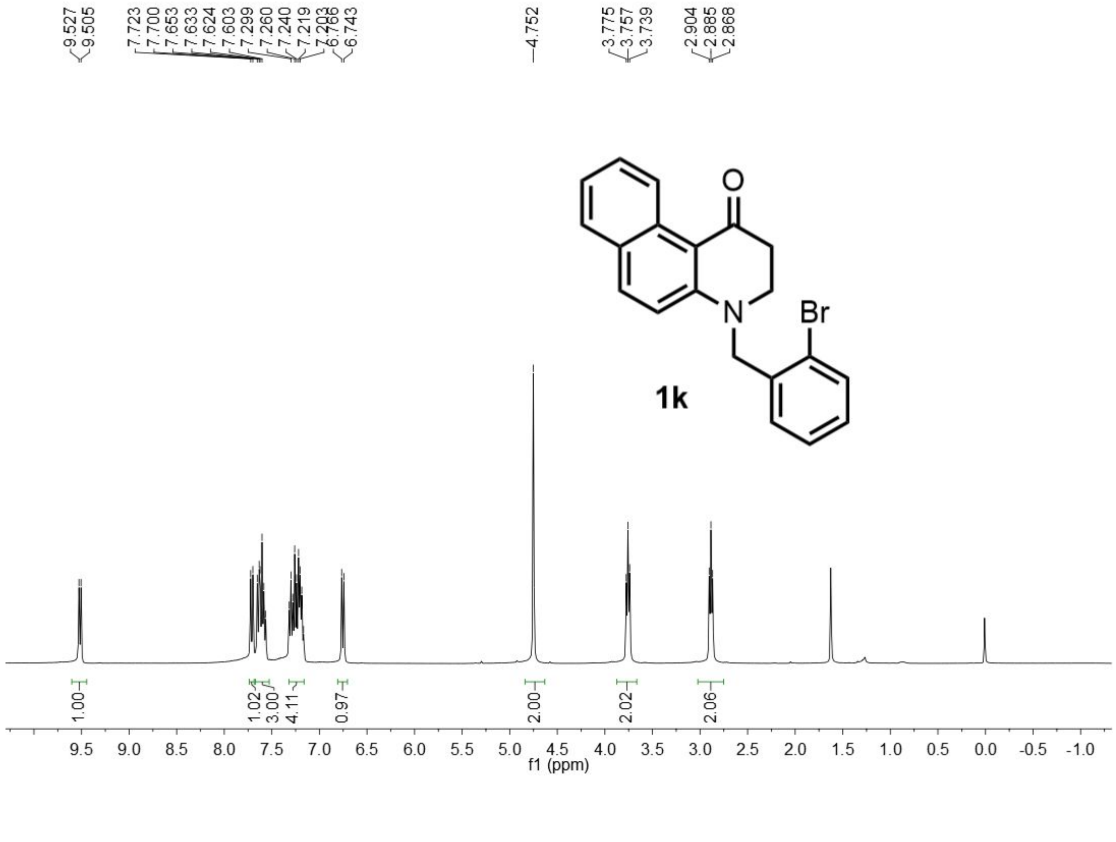

^{13}C NMR (100 MHz, $CDCl_3$) of compound **1k:**

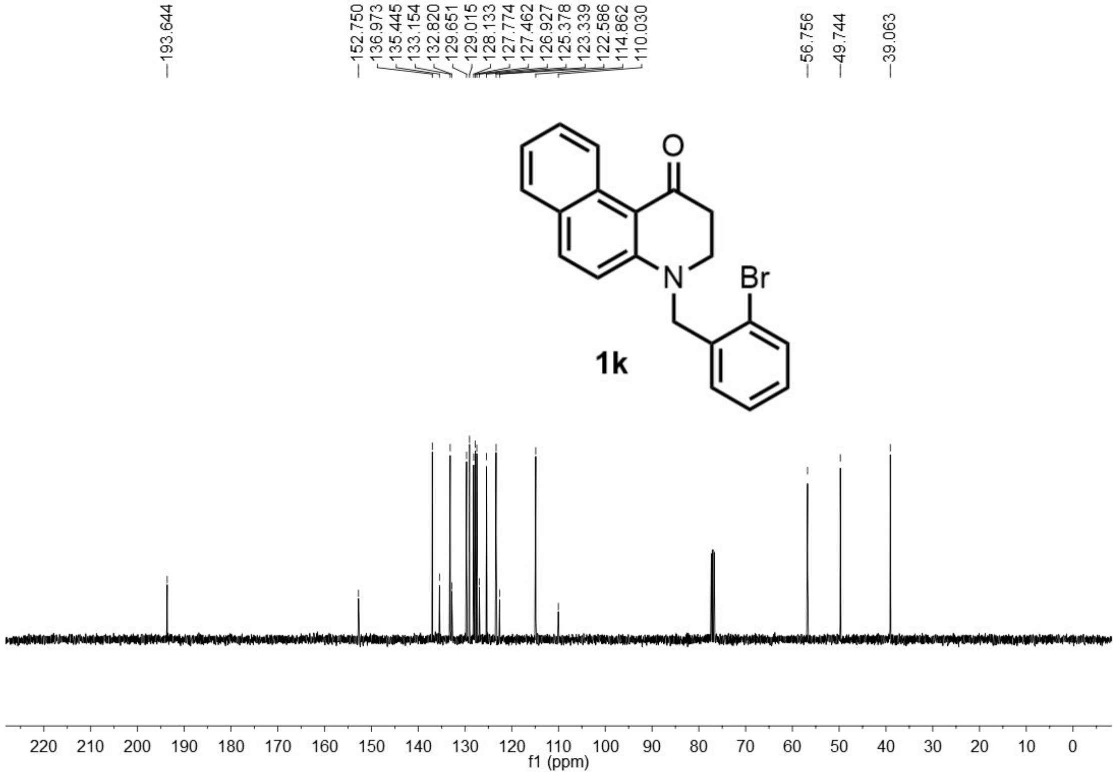

^{1}H NMR (400 MHz, $CDCl_3$) of compound **1l:**

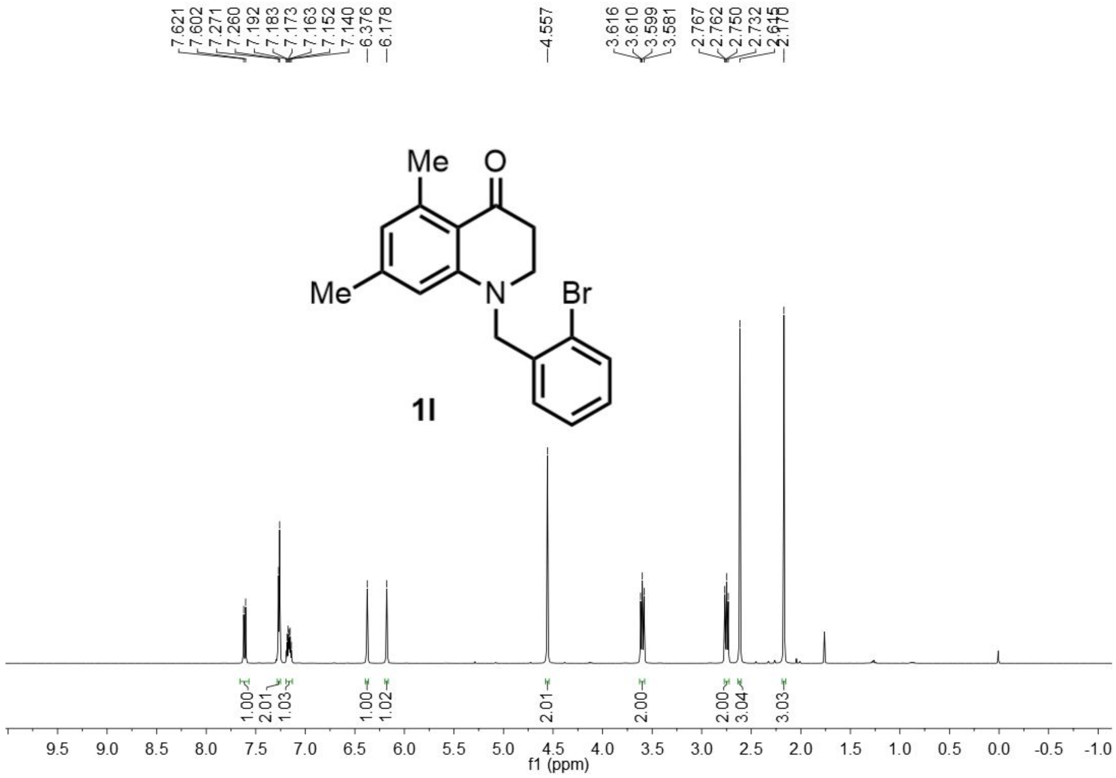

^{13}C NMR (100 MHz, $CDCl_3$) of compound **1l:**

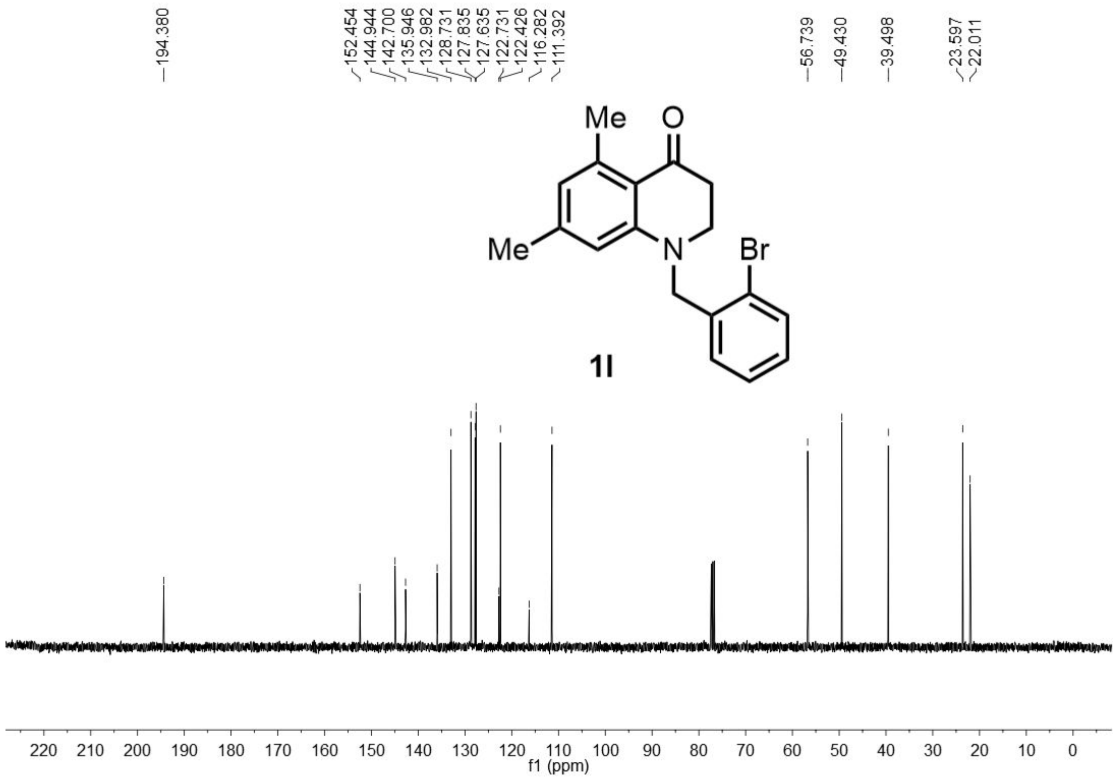

^{1}H NMR (400 MHz, $CDCl_3$) of compound **1m:**

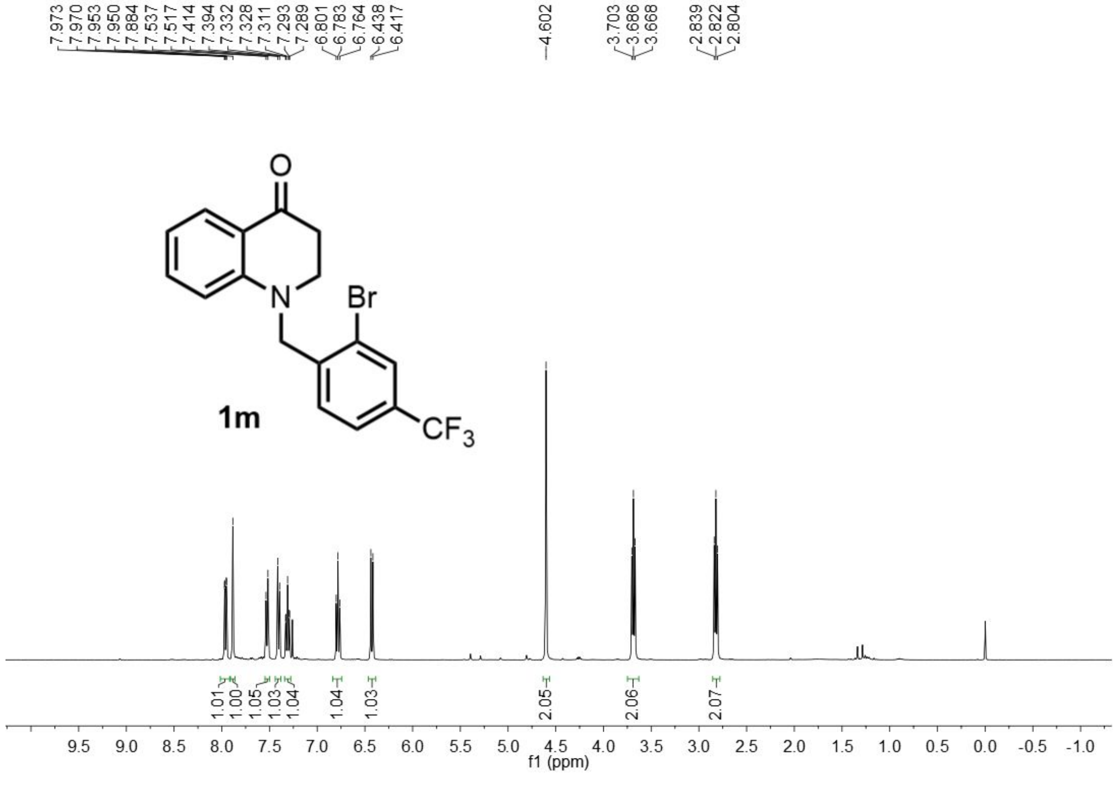

^{13}C NMR (100 MHz, $CDCl_3$) of compound **1m:**

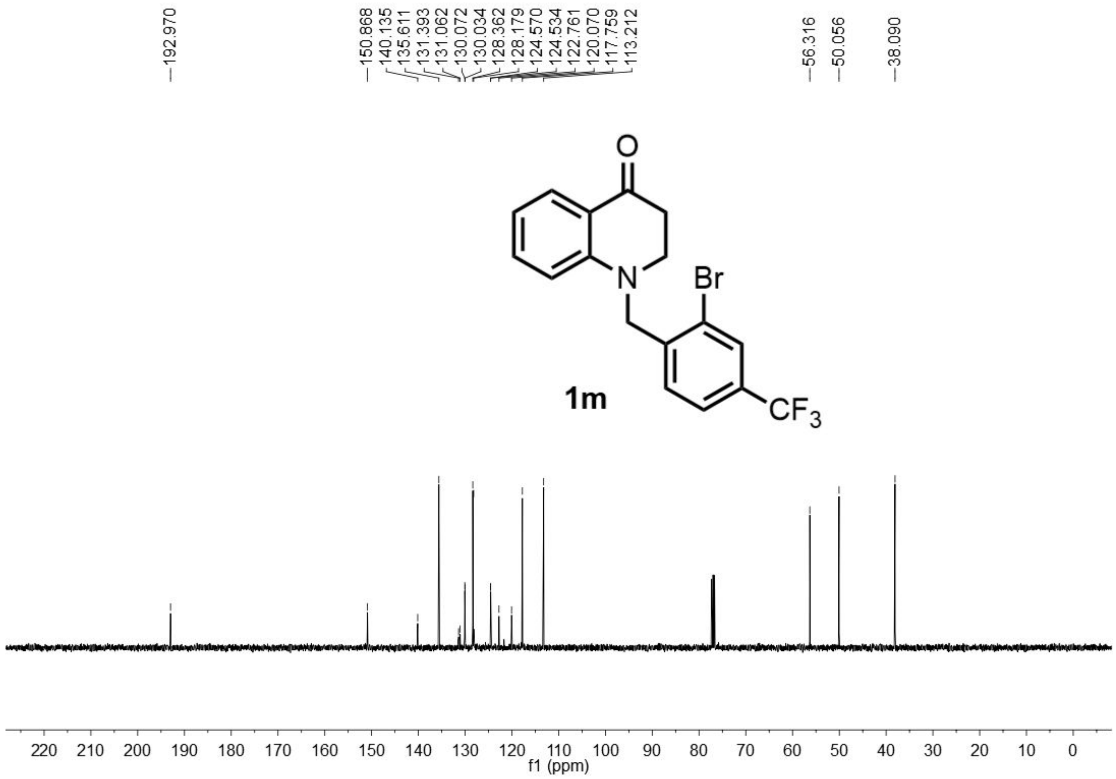

^{19}F NMR (377 MHz, $CDCl_3$) of compound **1m:**

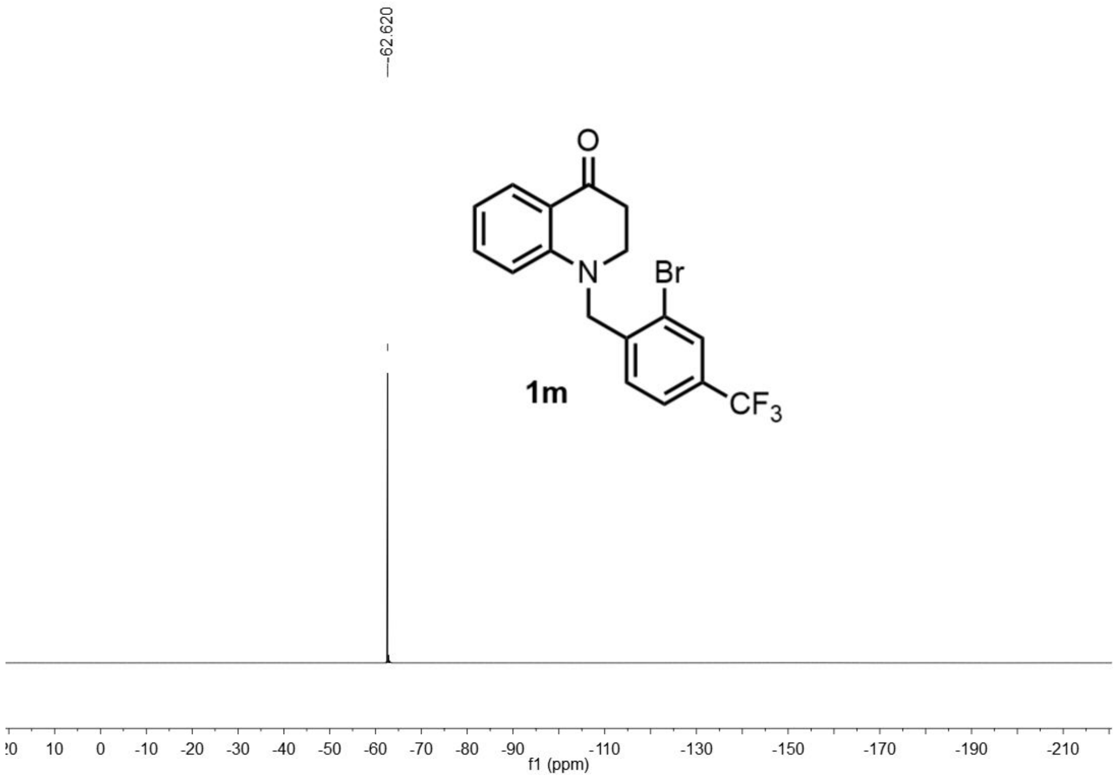

^{1}H NMR (400 MHz, $CDCl_3$) of compound **1n:**

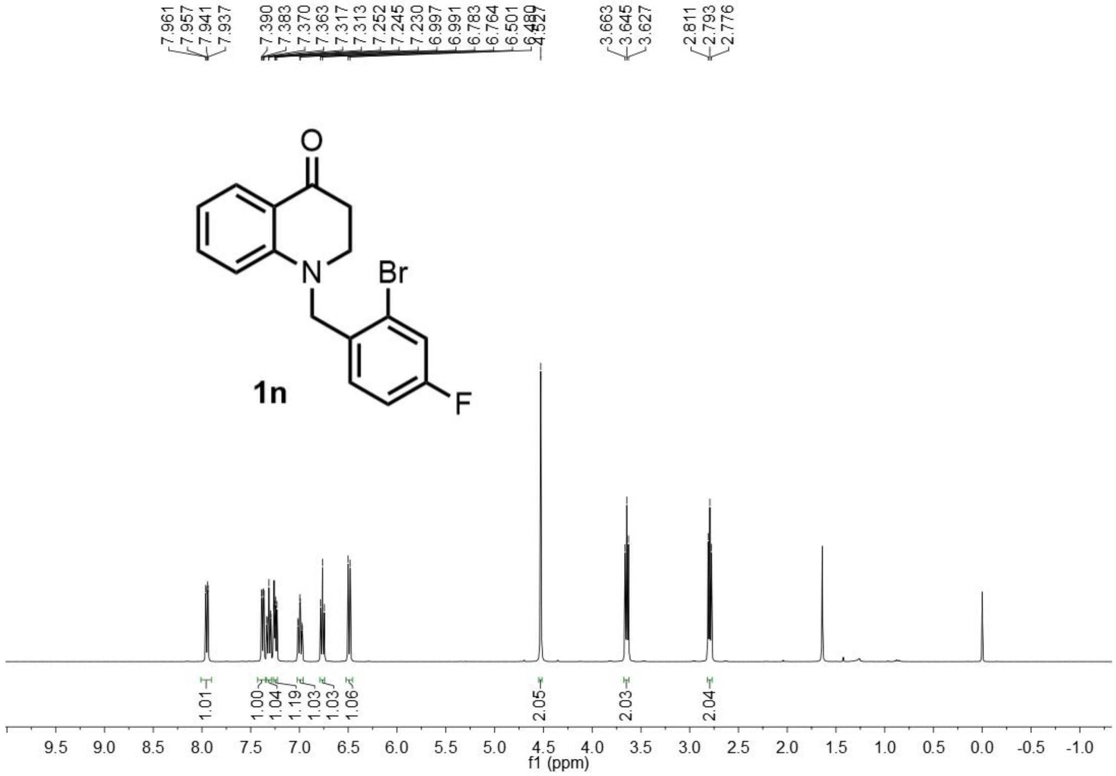

^{13}C NMR (100 MHz, $CDCl_3$) of compound **1n:**

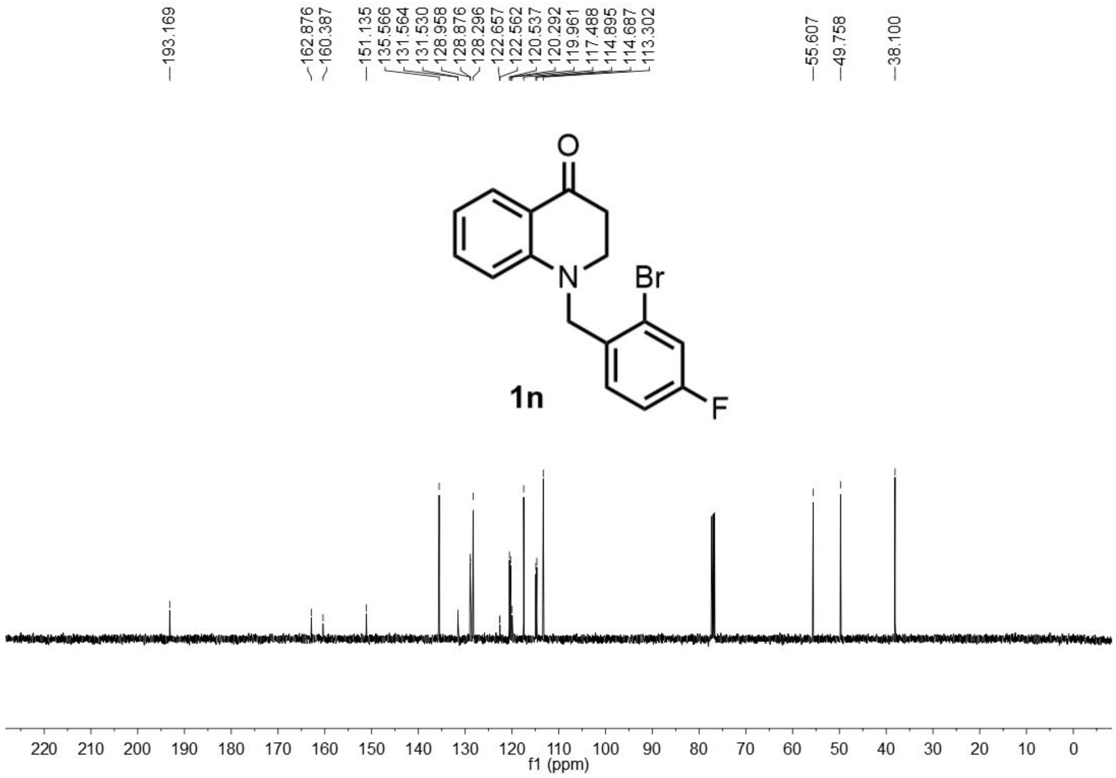

^{19}F NMR (377 MHz, $CDCl_3$) of compound **1n:**

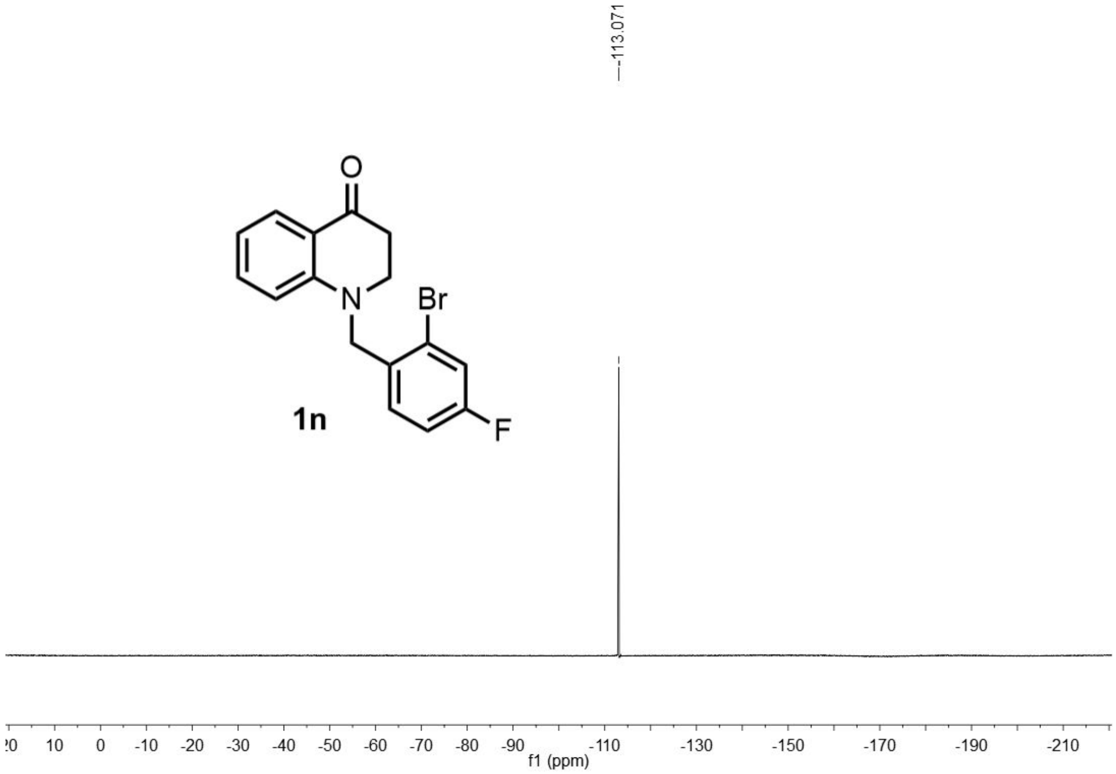

^{1}H NMR (400 MHz, $CDCl_3$) of compound **1o:**

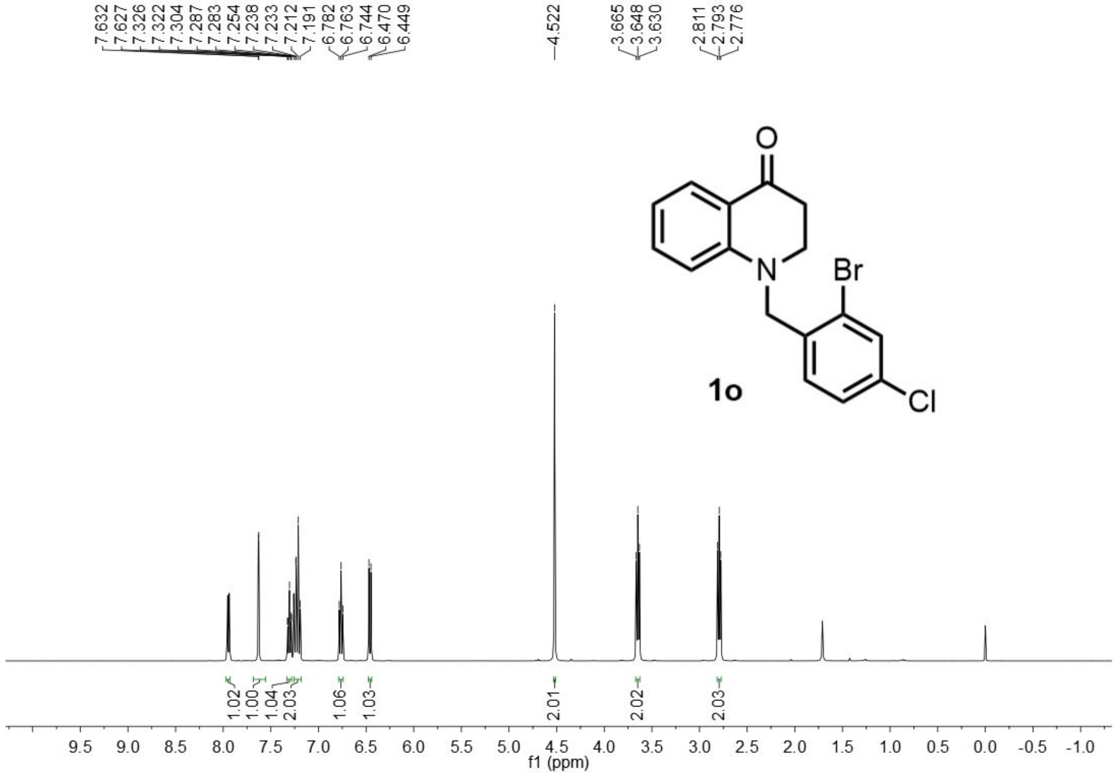

^{13}C NMR (100 MHz, $CDCl_3$) of compound **1o:**

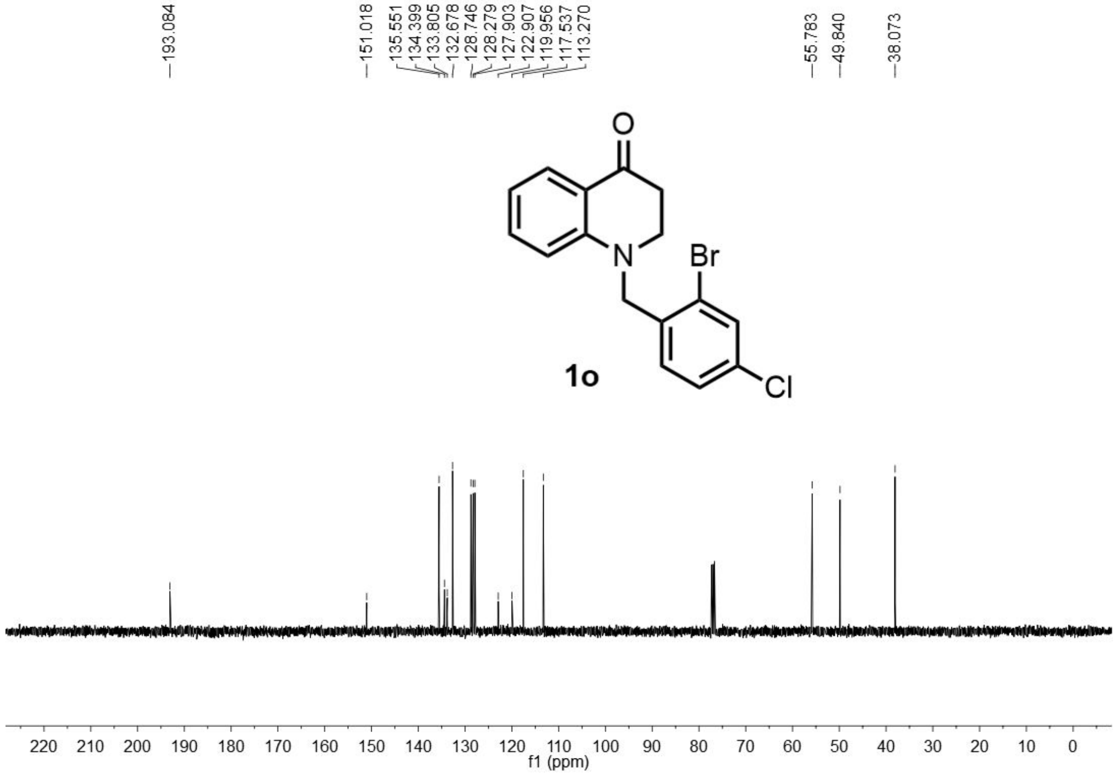

^{1}H NMR (400 MHz, $CDCl_3$) of compound **1p:**

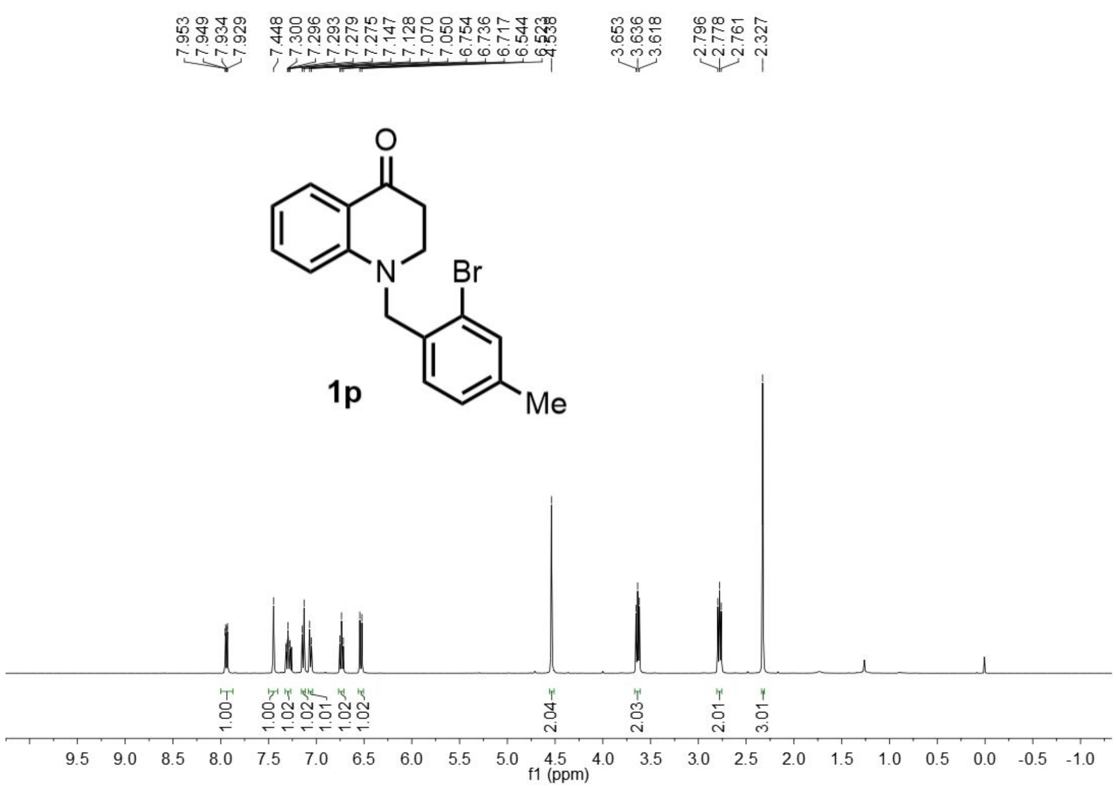

^{13}C NMR (100 MHz, $CDCl_3$) of compound **1p:**

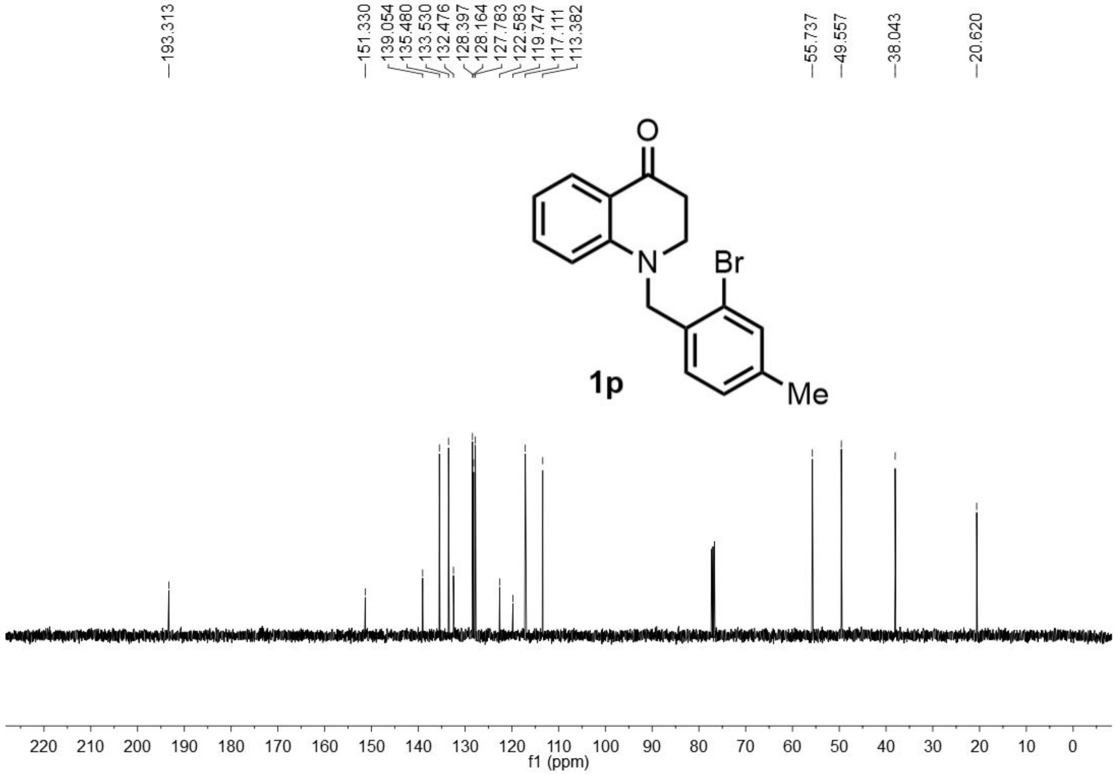

^{1}H NMR (400 MHz, $CDCl_3$) of compound **1q:**

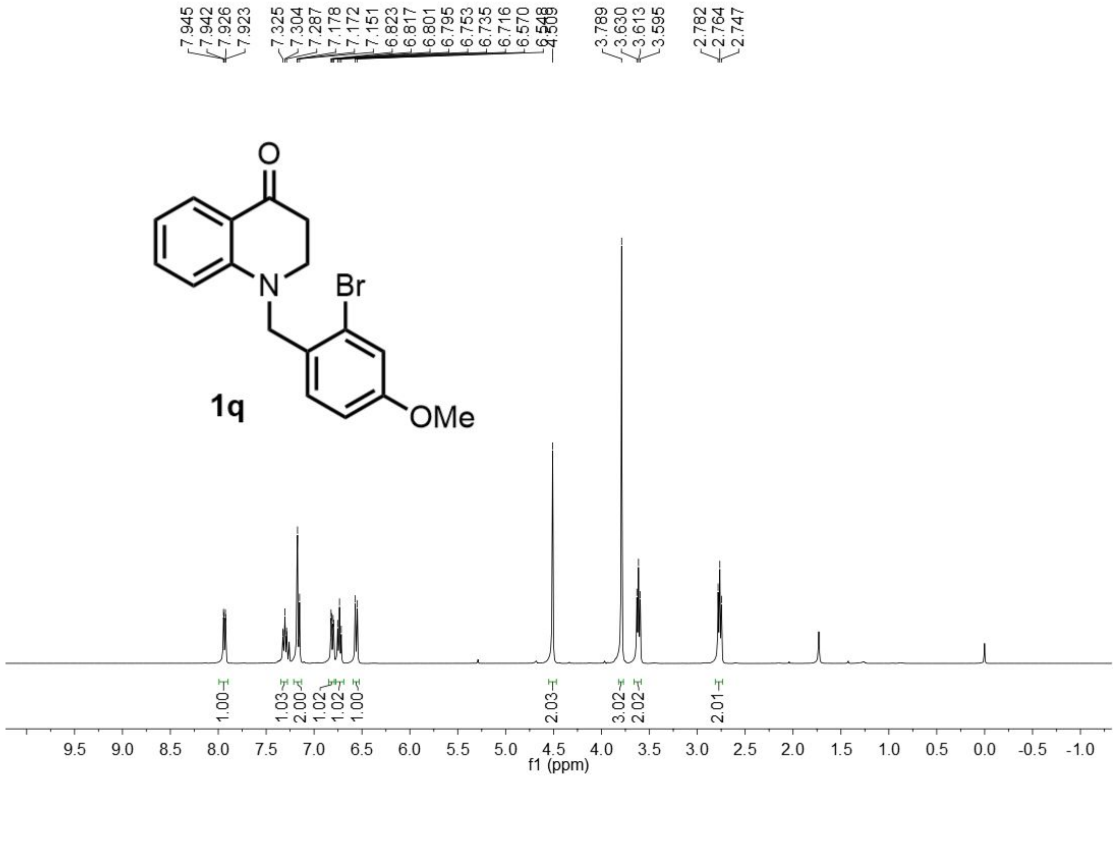

^{13}C NMR (100 MHz, $CDCl_3$) of compound **1q:**

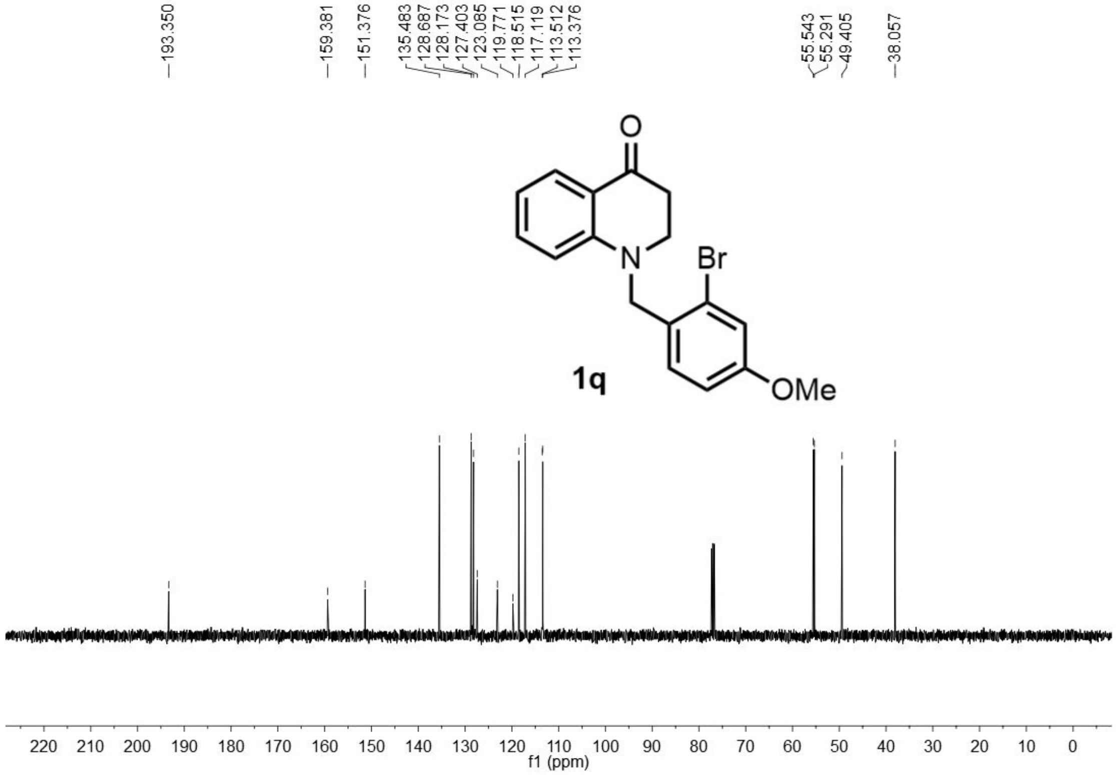

^{1}H NMR (400 MHz, $CDCl_3$) of compound **1r:**

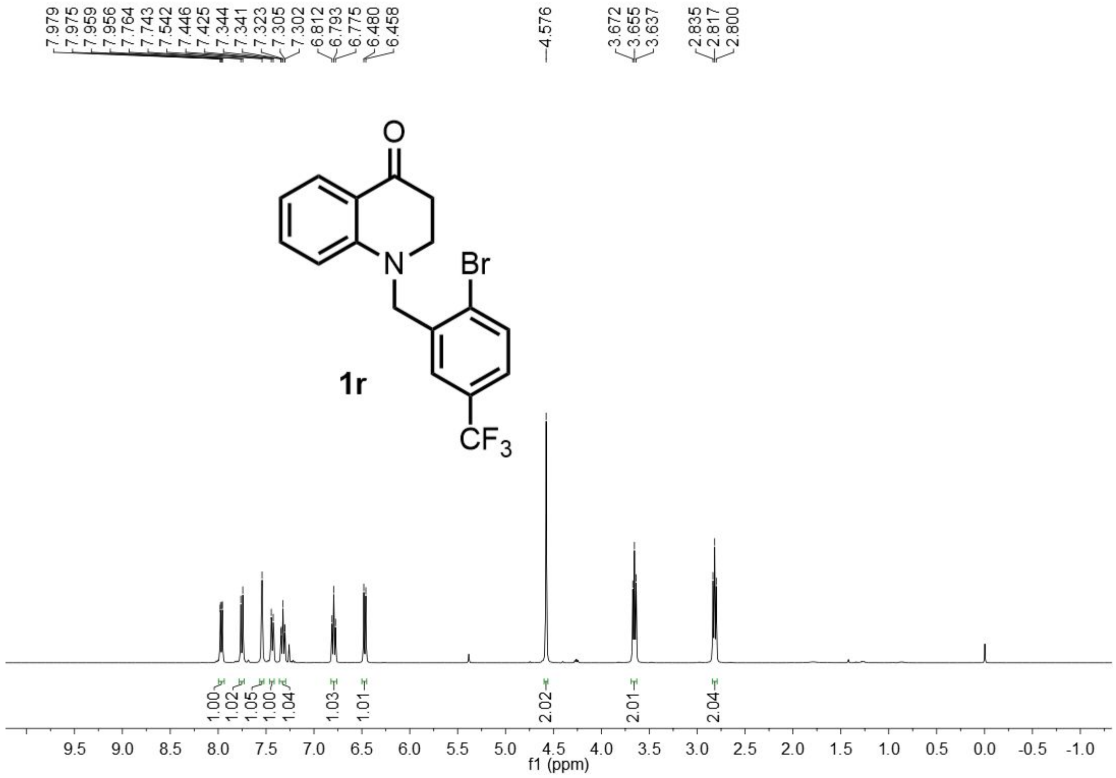

^{13}C NMR (100 MHz, $CDCl_3$) of compound **1r:**

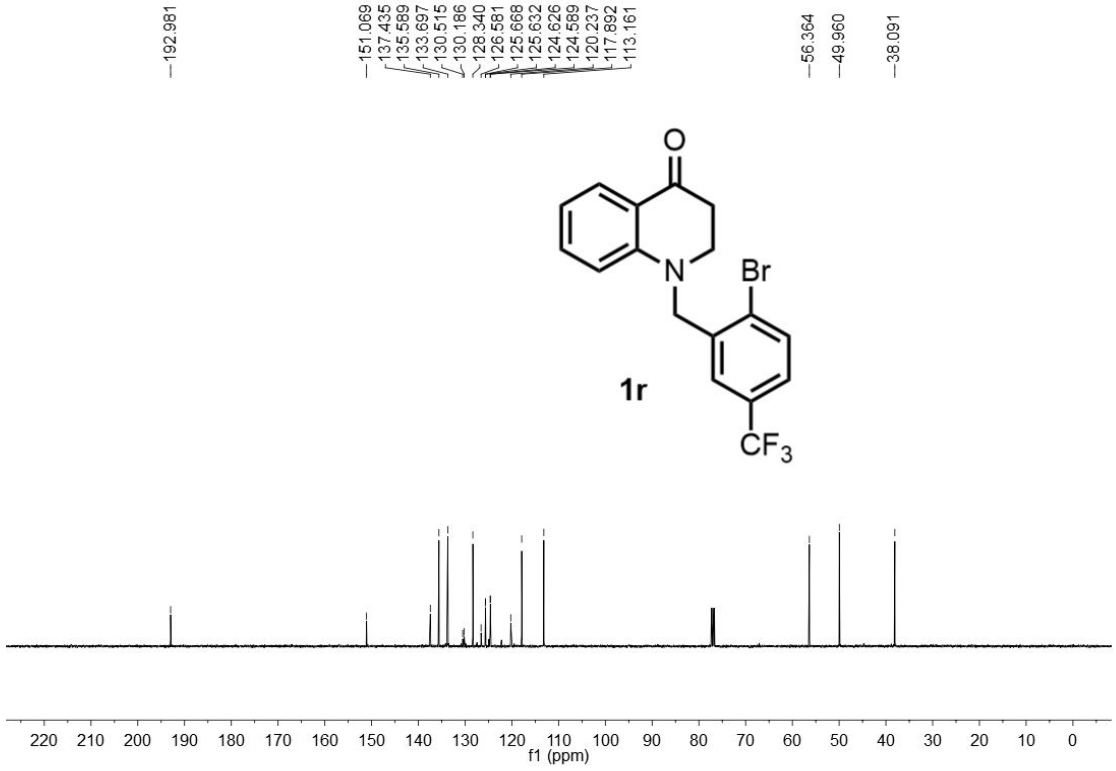

^{19}F NMR (377 MHz, $CDCl_3$) of compound **1r:**

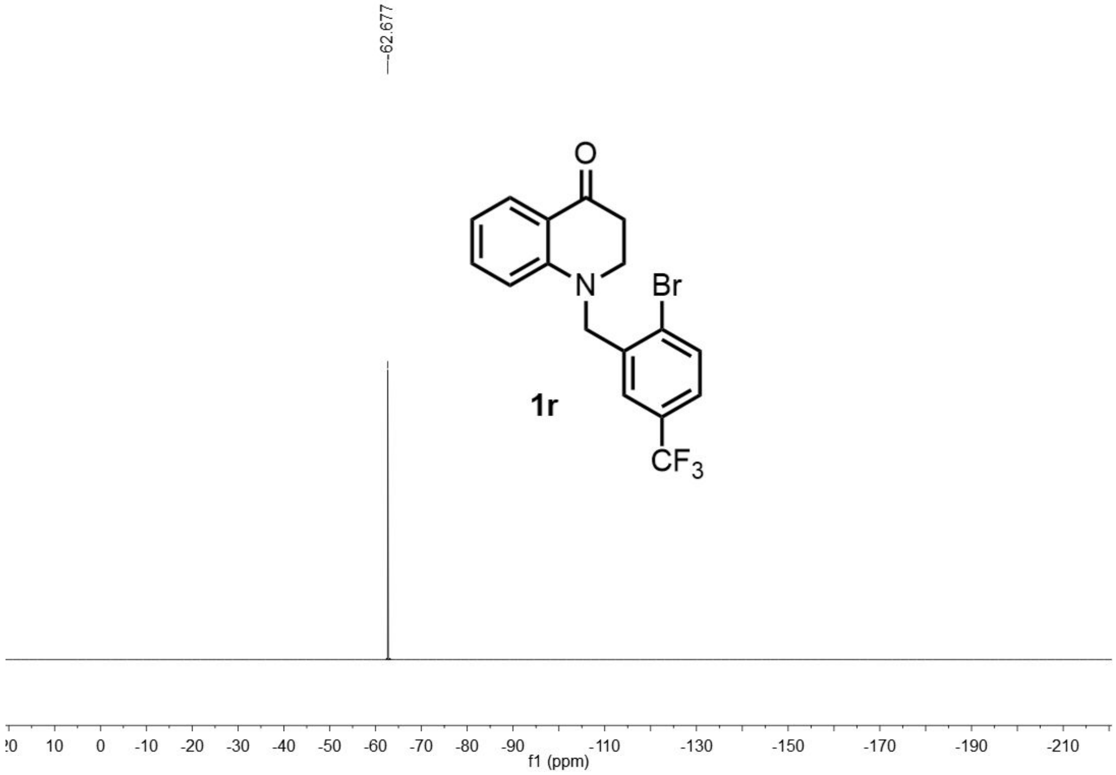

^{1}H NMR (400 MHz, $CDCl_3$) of compound **1s:**

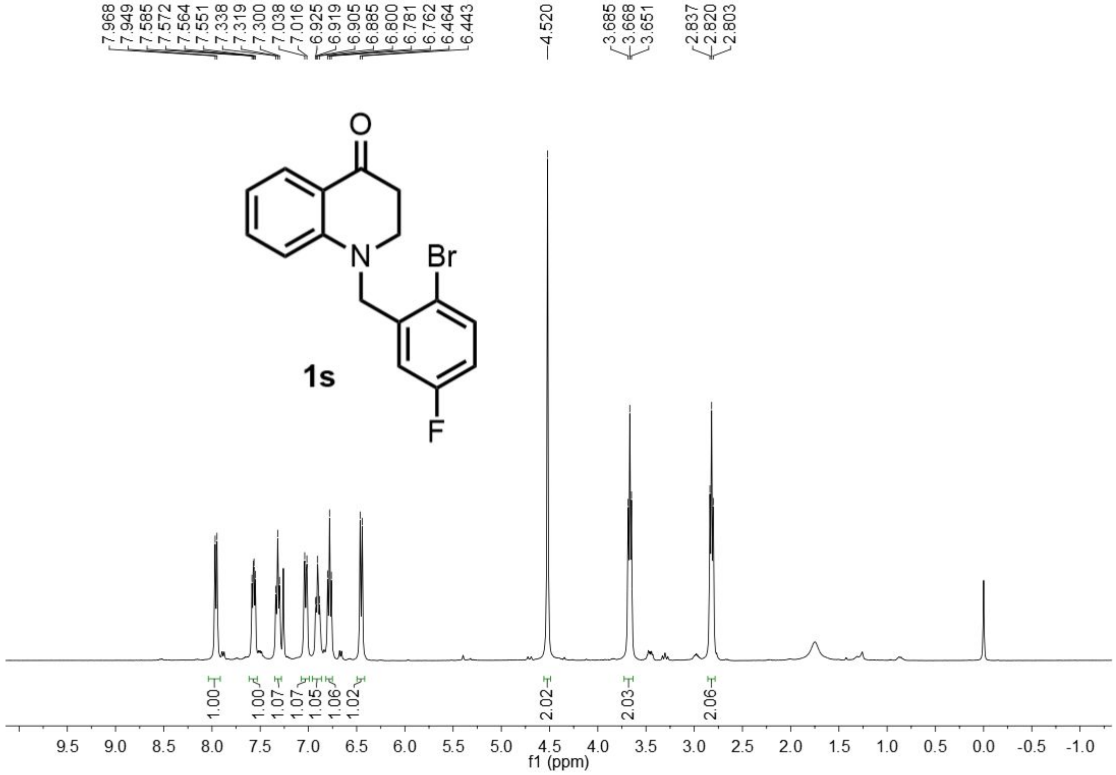

^{13}C NMR (100 MHz, $CDCl_3$) of compound **1s:**

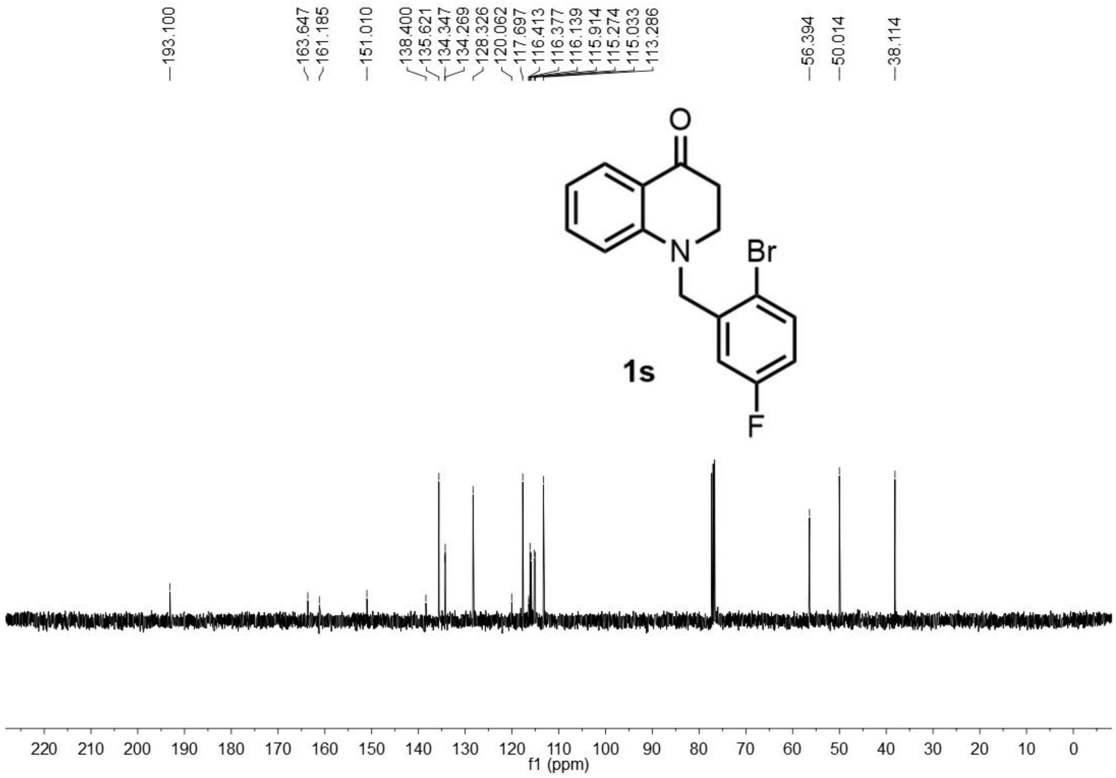

^{19}F NMR (377 MHz, $CDCl_3$) of compound **1s:**

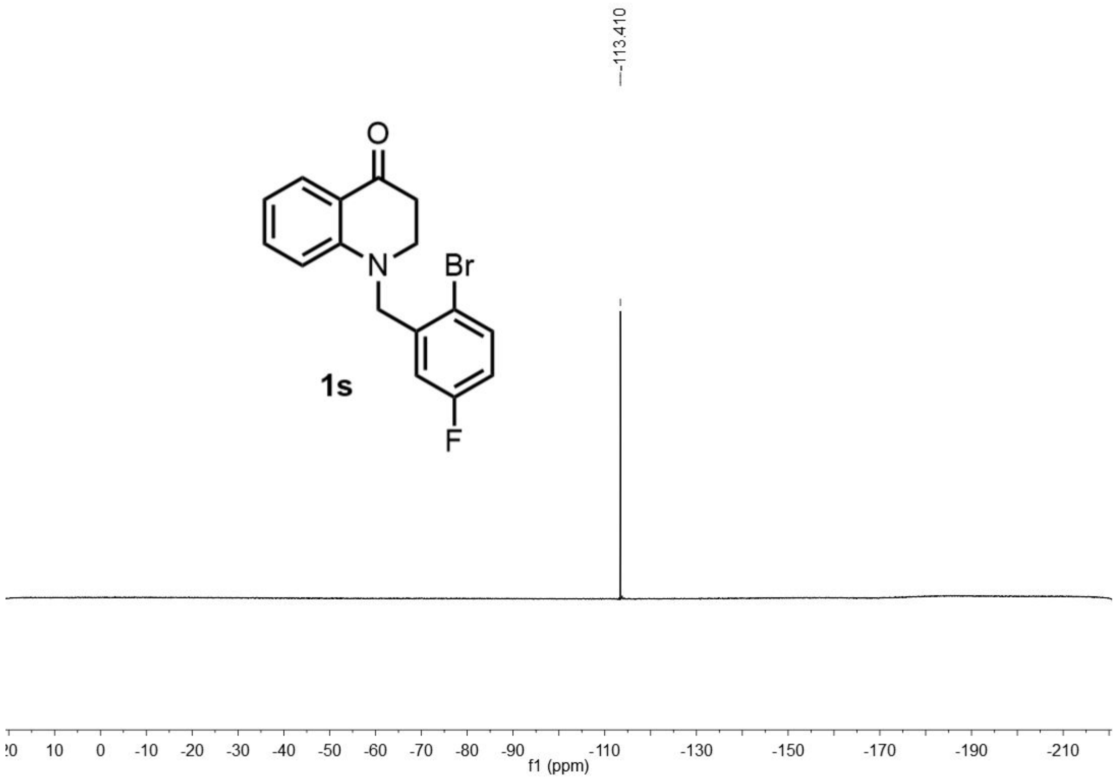

^{1}H NMR (400 MHz, $CDCl_3$) of compound **1t:**

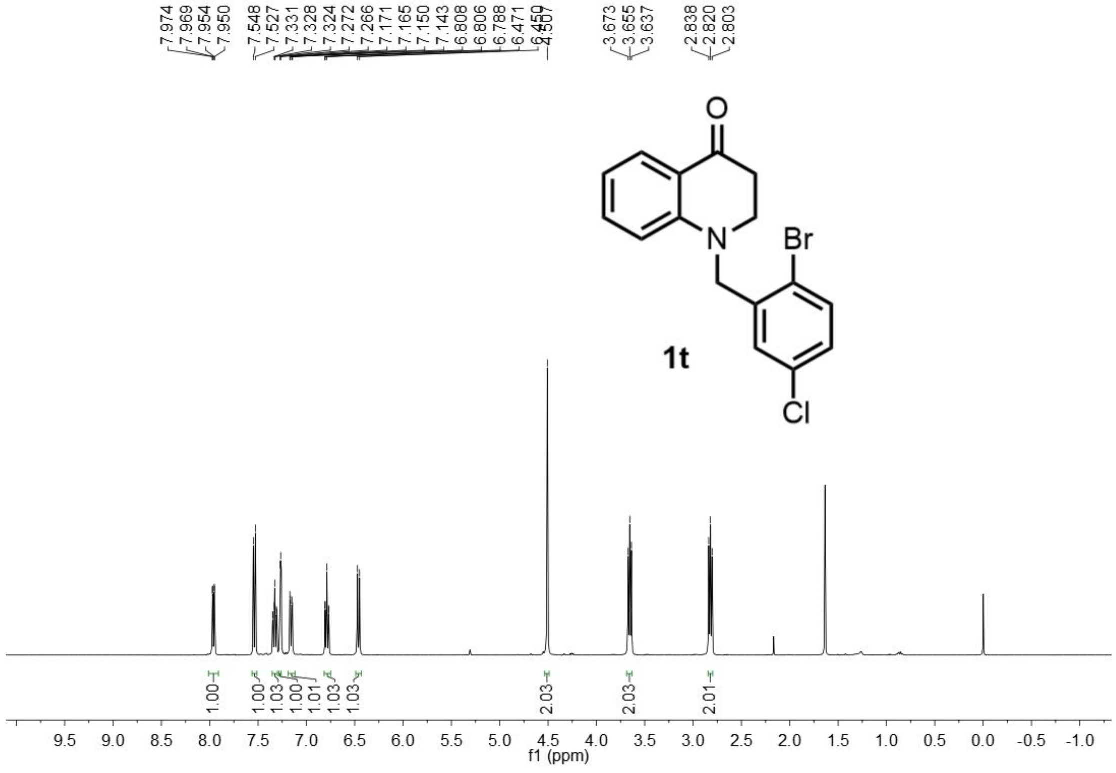

^{13}C NMR (100 MHz, $CDCl_3$) of compound **1t:**

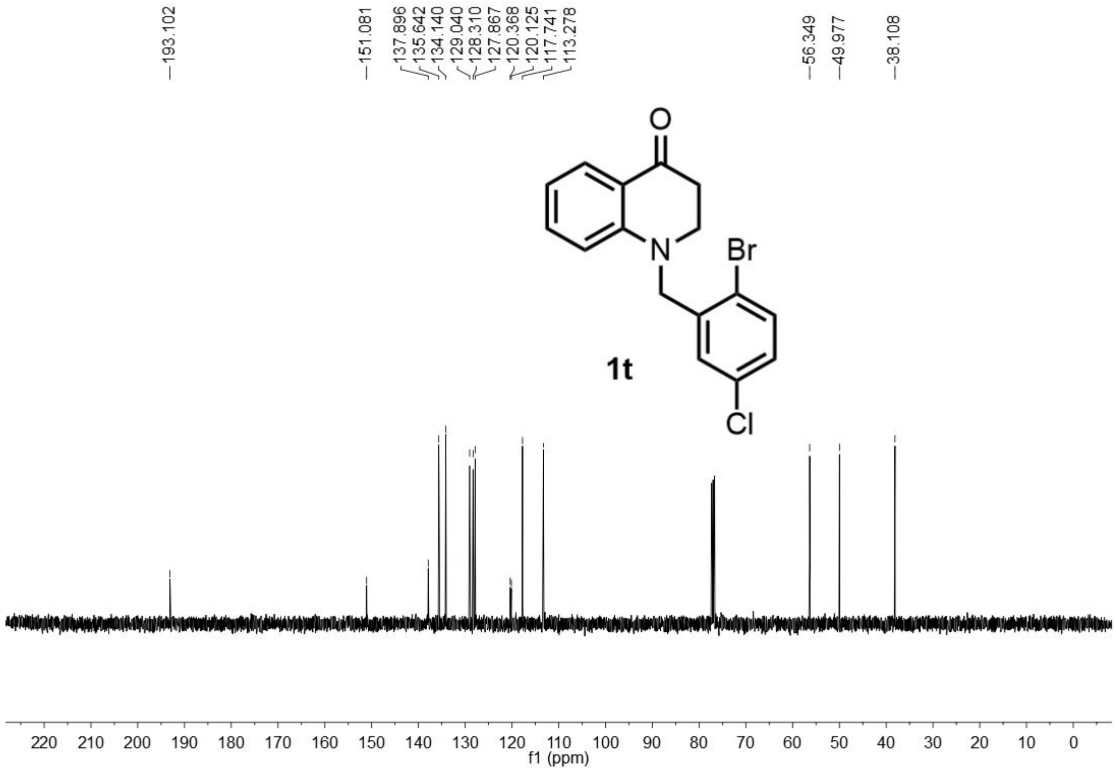

^{1}H NMR (400 MHz, $CDCl_3$) of compound **1u:**

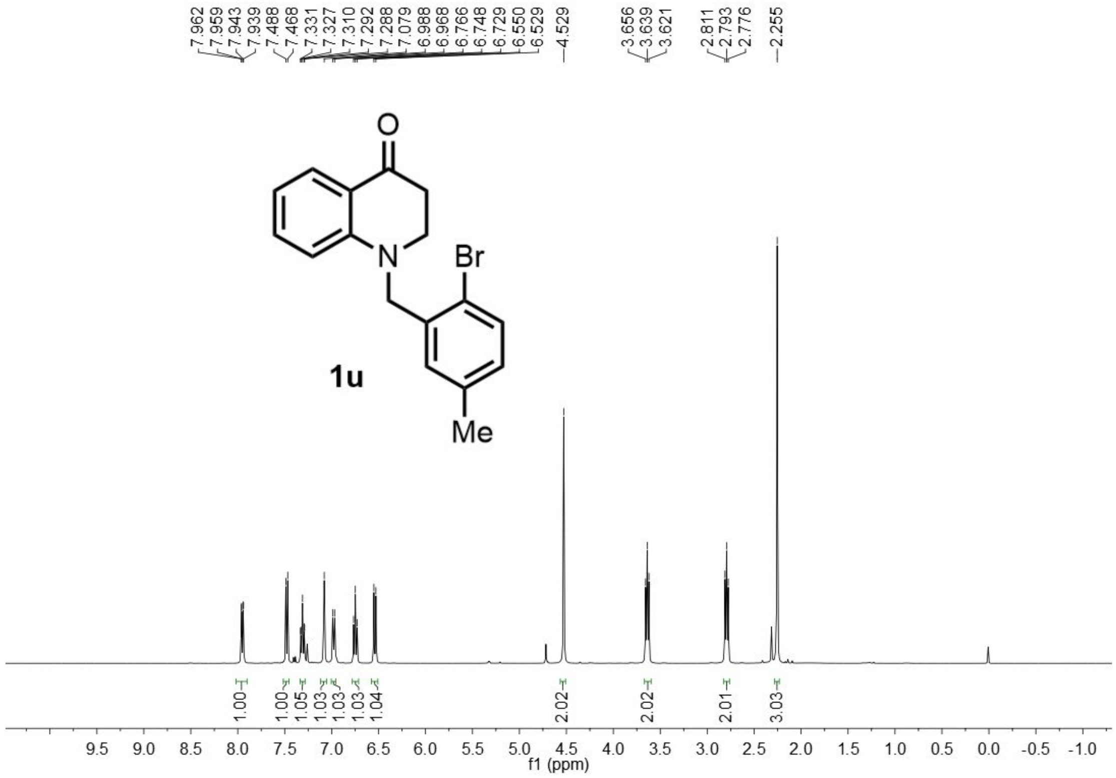

^{13}C NMR (100 MHz, $CDCl_3$) of compound **1u:**

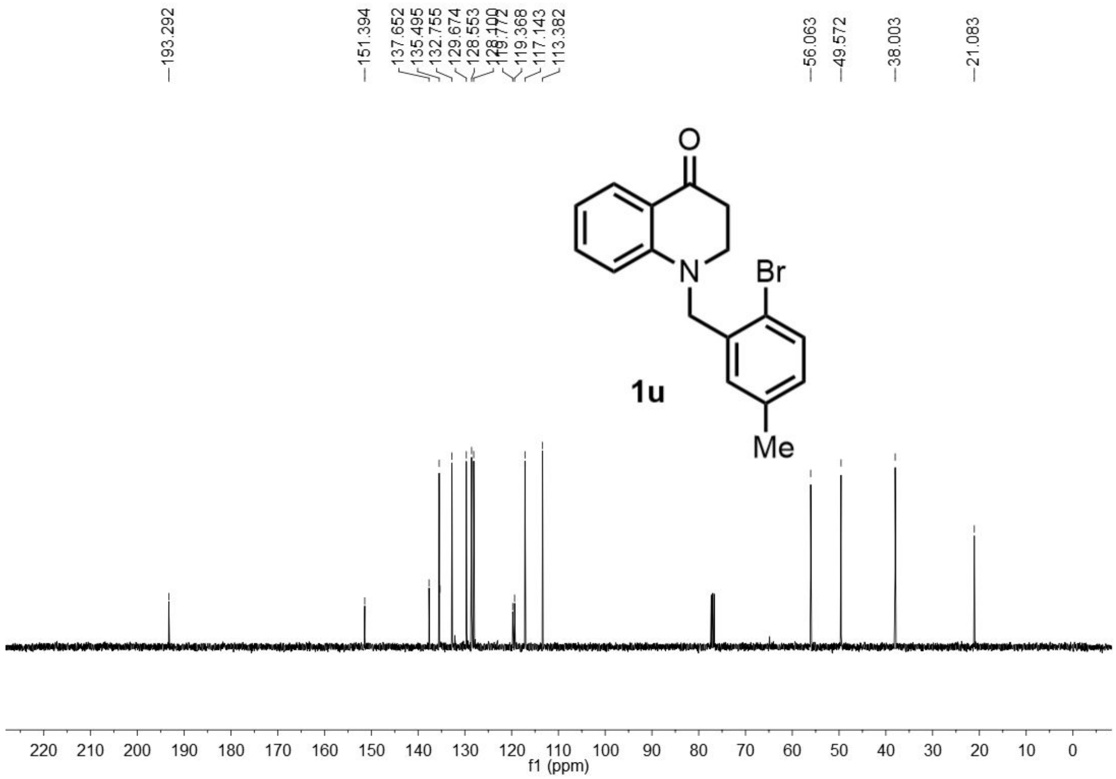

^{1}H NMR (400 MHz, $CDCl_3$) of compound **1v:**

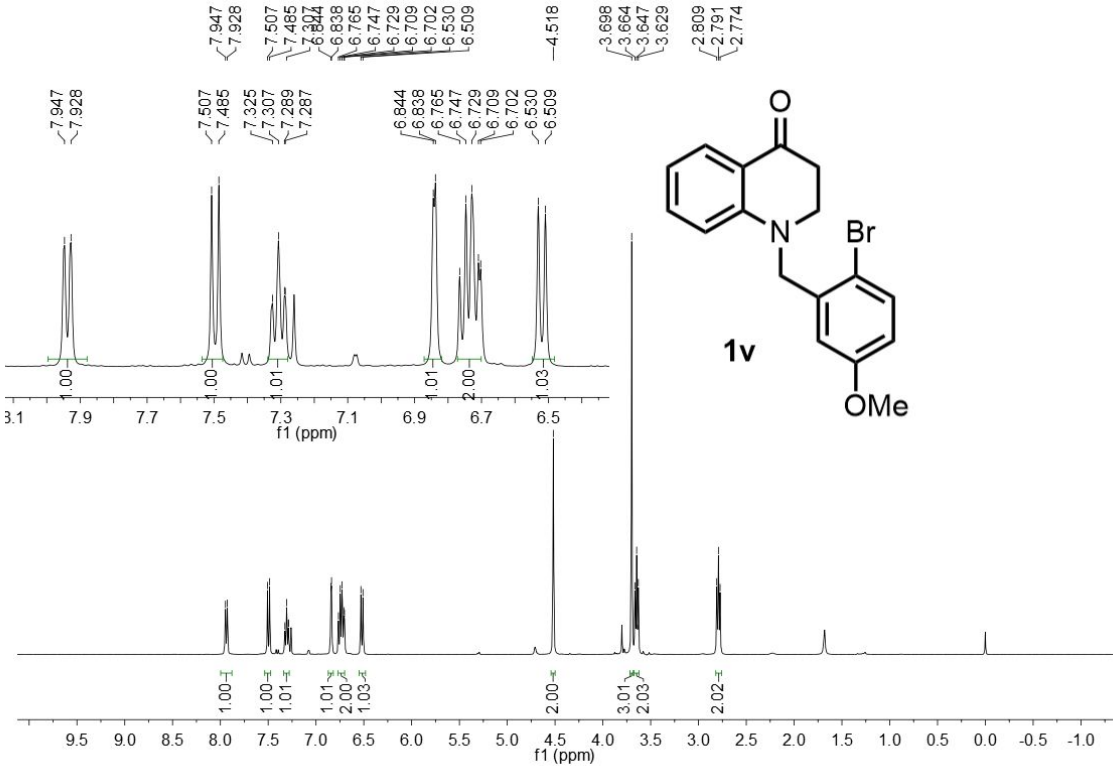

^{13}C NMR (100 MHz, $CDCl_3$) of compound **1v:**

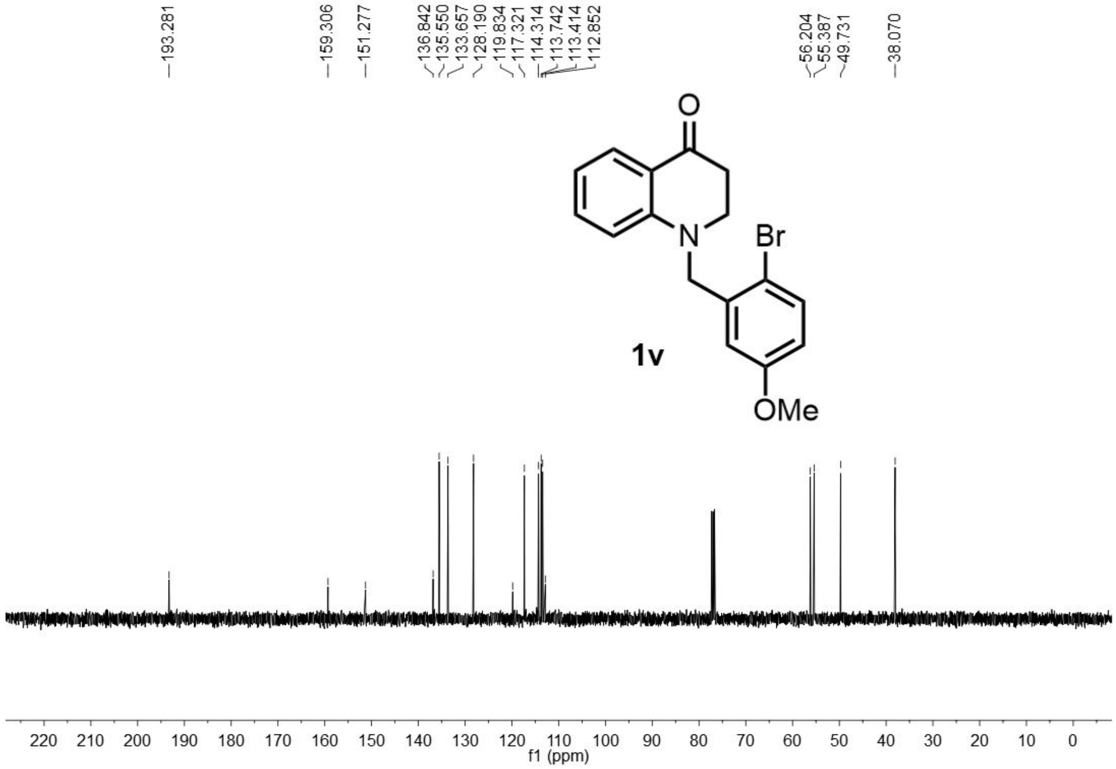

^{1}H NMR (400 MHz, $CDCl_3$) of compound **1w:**

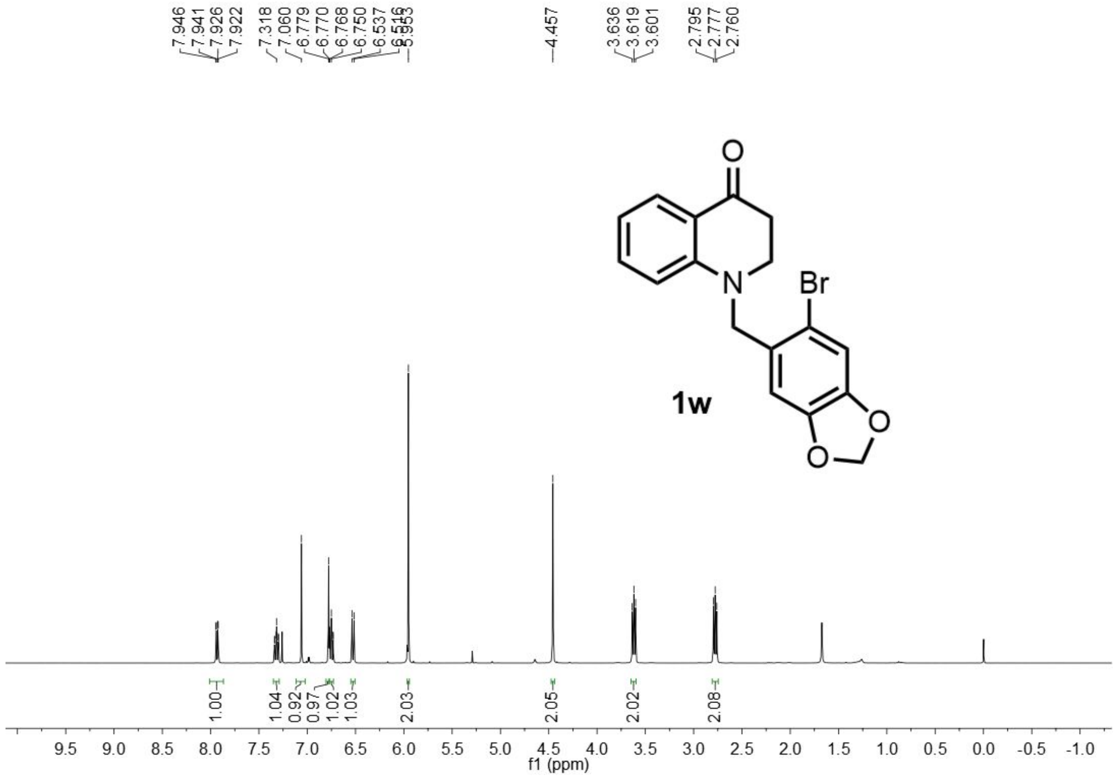

^{13}C NMR (100 MHz, $CDCl_3$) of compound **1w:**

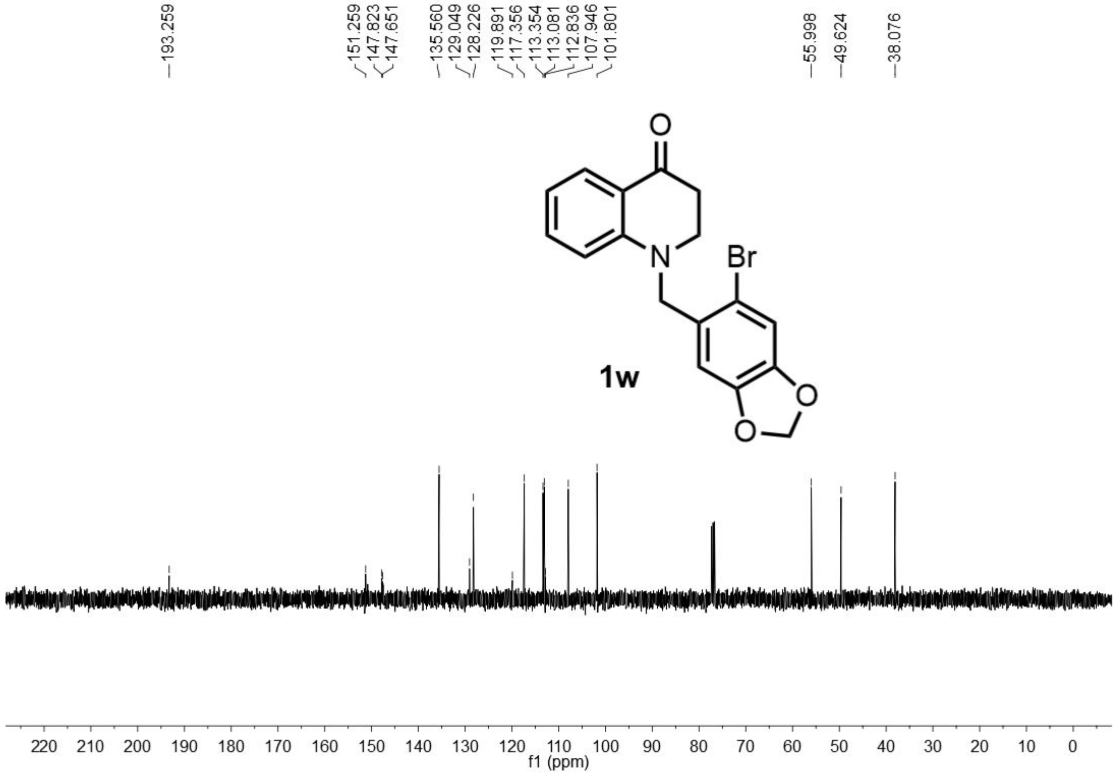

^{1}H NMR (400 MHz, $CDCl_3$) of compound **1x:**

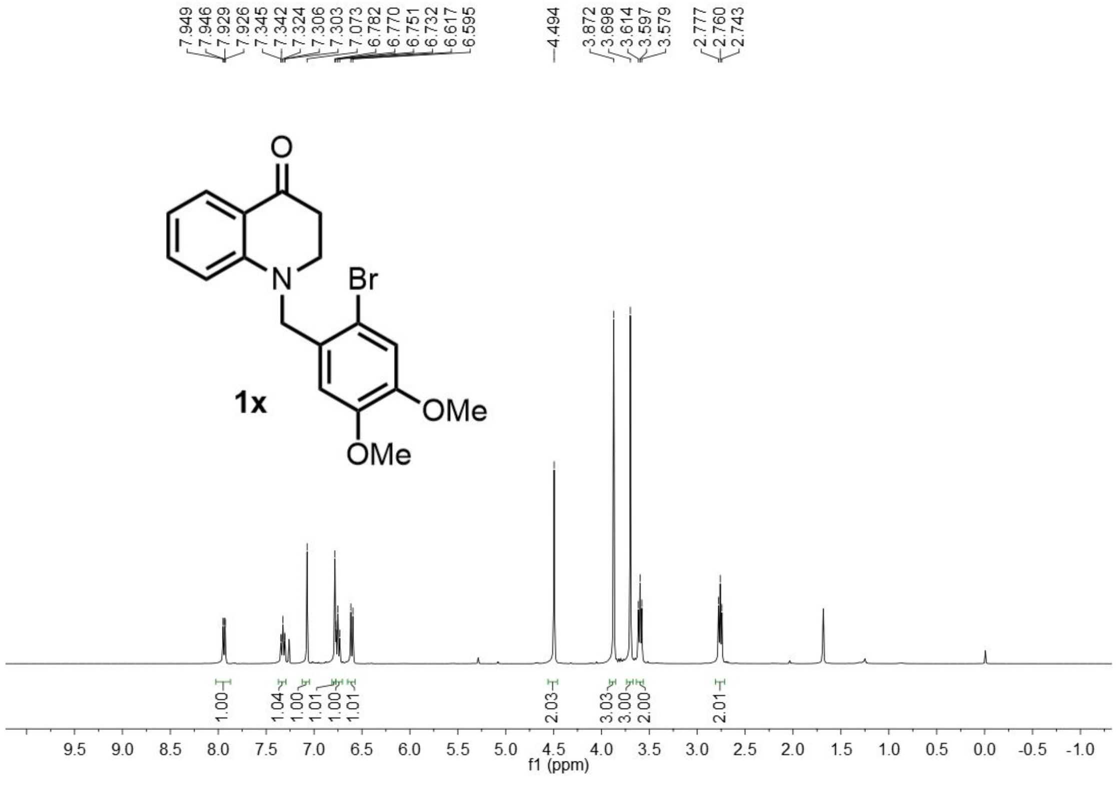

^{13}C NMR (100 MHz, $CDCl_3$) of compound **1x:**

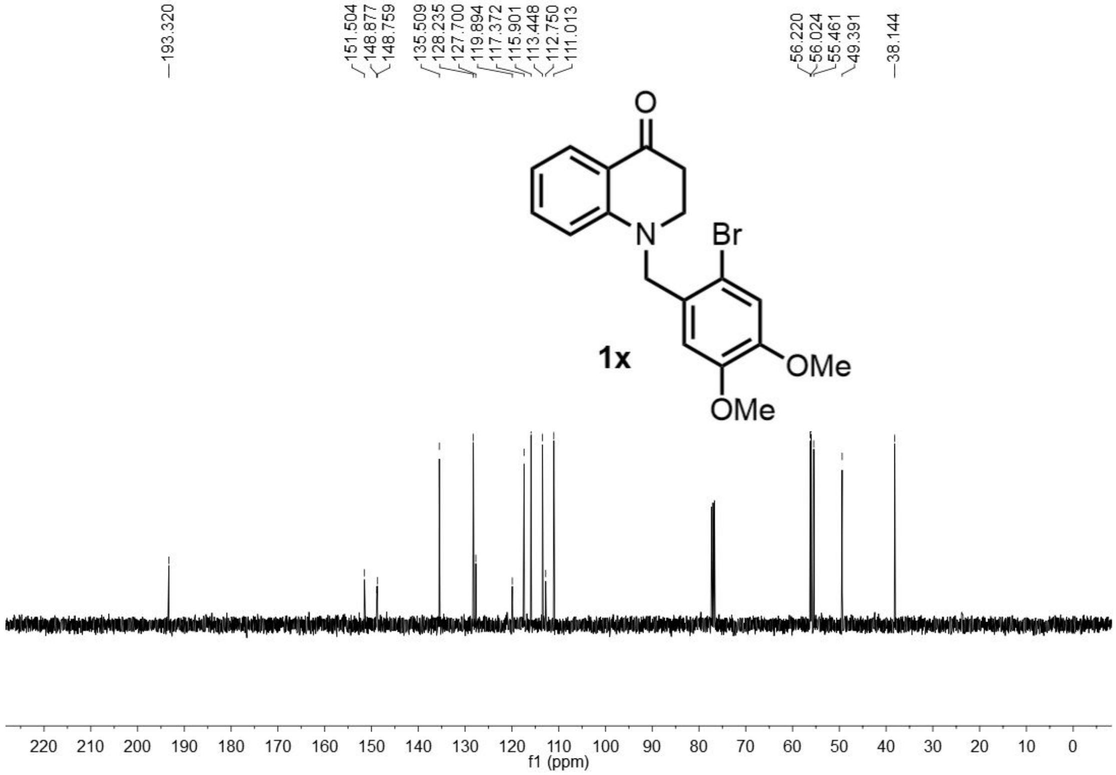

^{1}H NMR (400 MHz, $CDCl_3$) of compound **1y:**

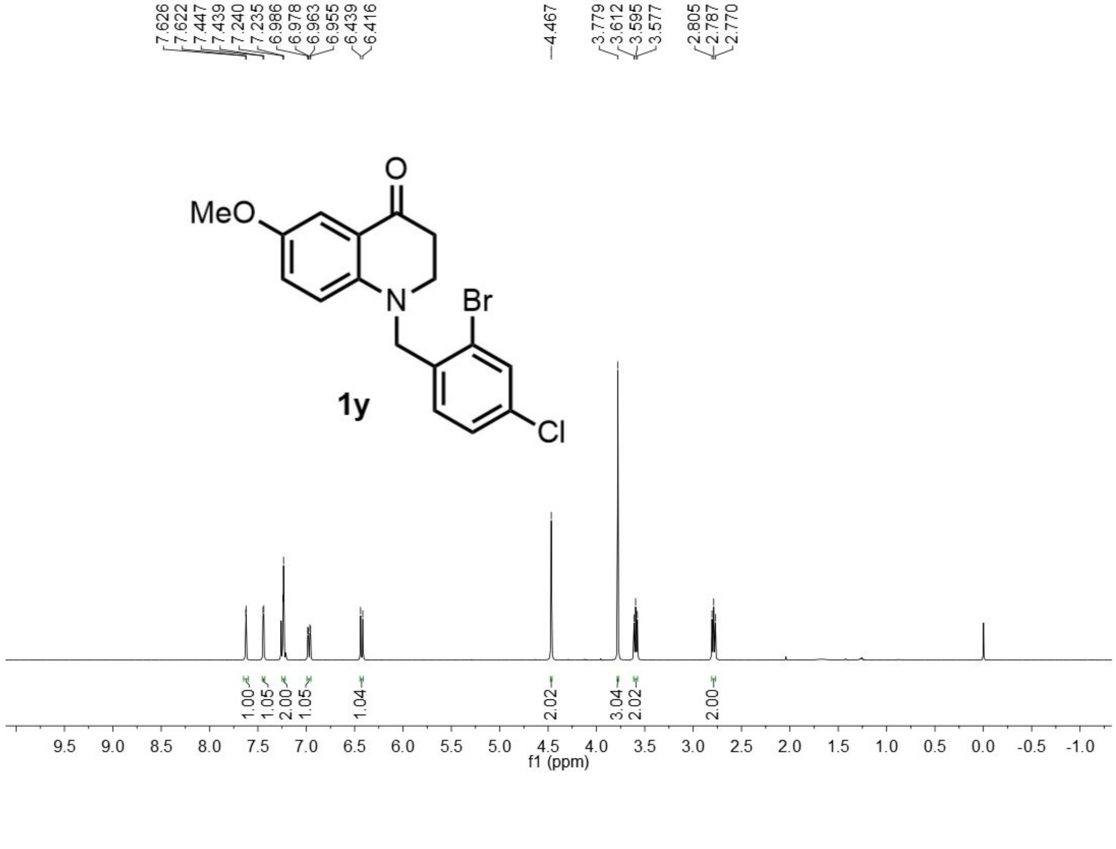

^{13}C NMR (100 MHz, $CDCl_3$) of compound **1y:**

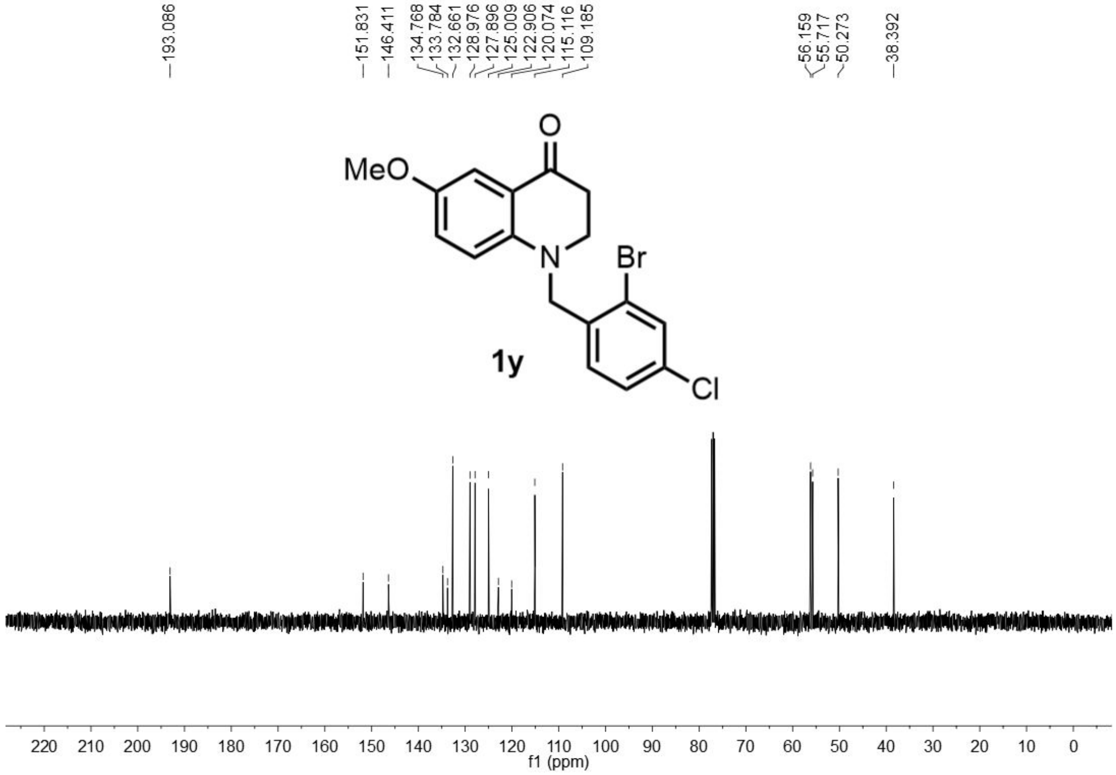

^{1}H NMR (400 MHz, $CDCl_3$) of compound **1z:**

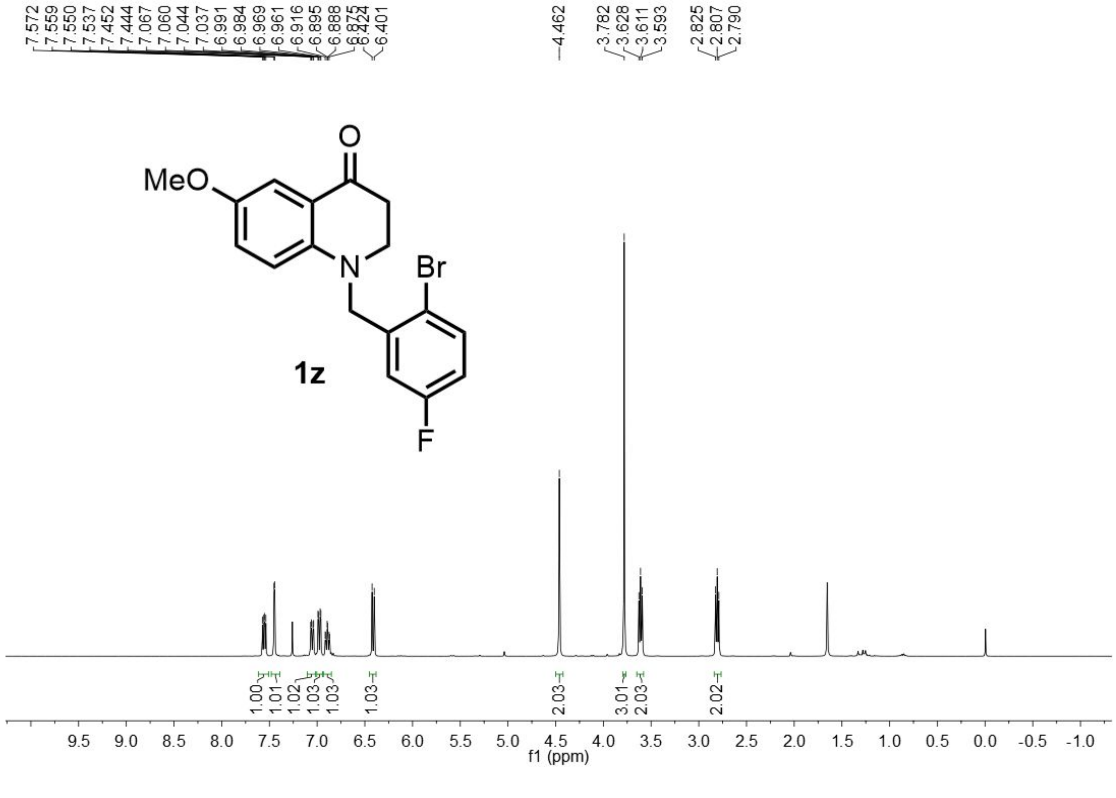

^{13}C NMR (100 MHz, $CDCl_3$) of compound **1z:**

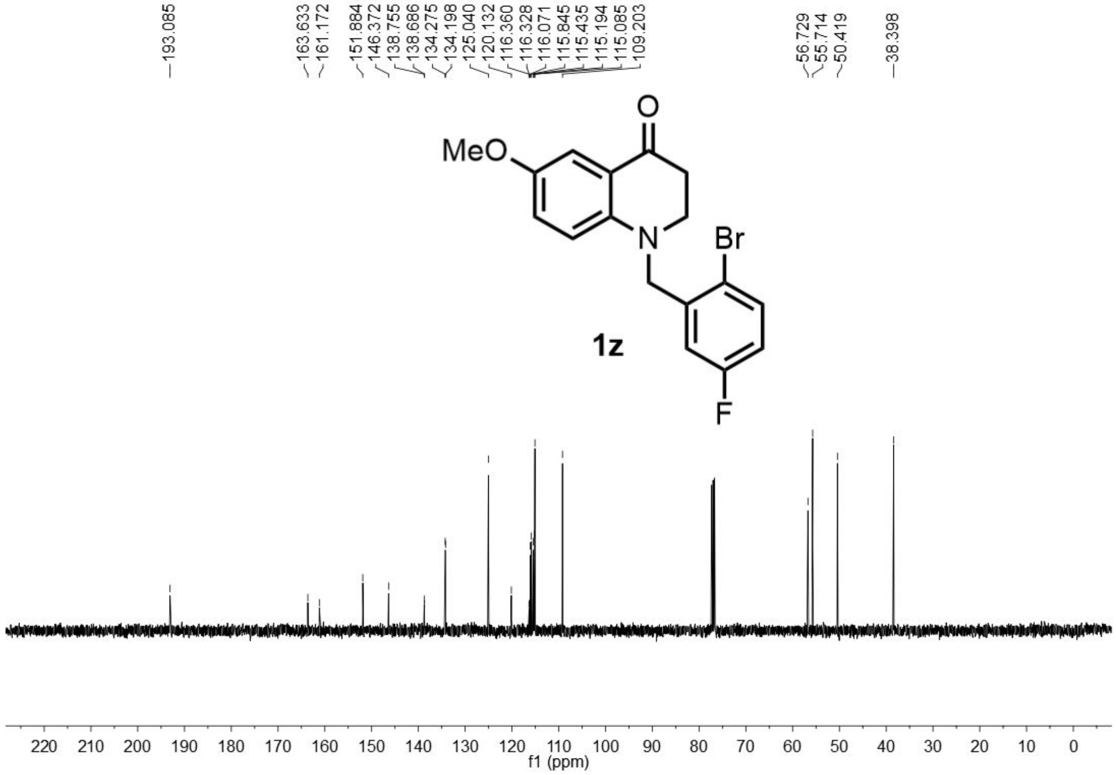

^{19}F NMR (377 MHz, $CDCl_3$) of compound **1z:**

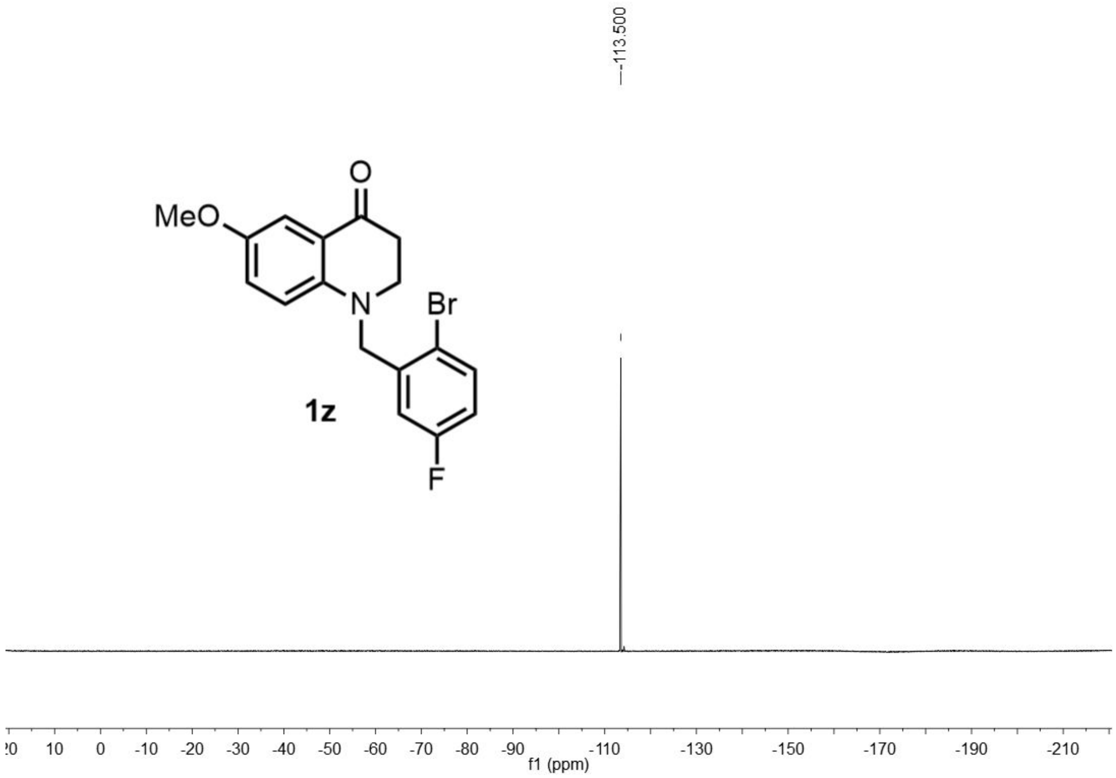

^1H NMR (400 MHz, $CDCl_3$) of compound **1aa:**

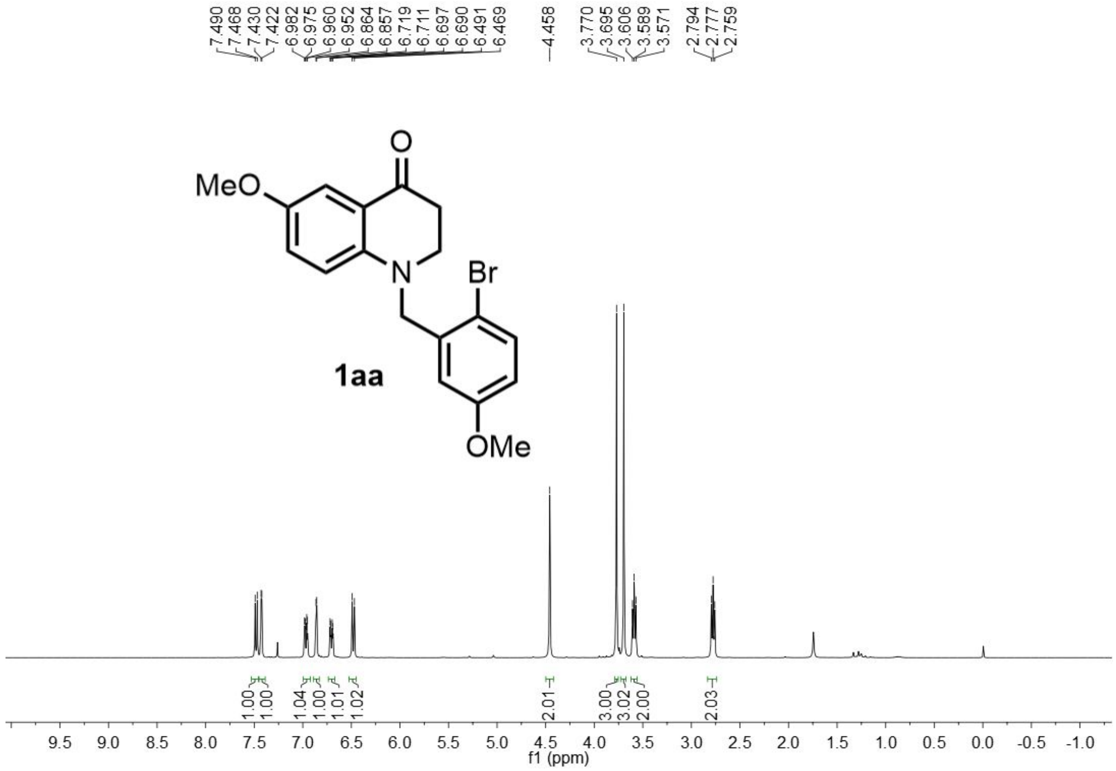

^{13}C NMR (100 MHz, $CDCl_3$) of compound **1aa:**

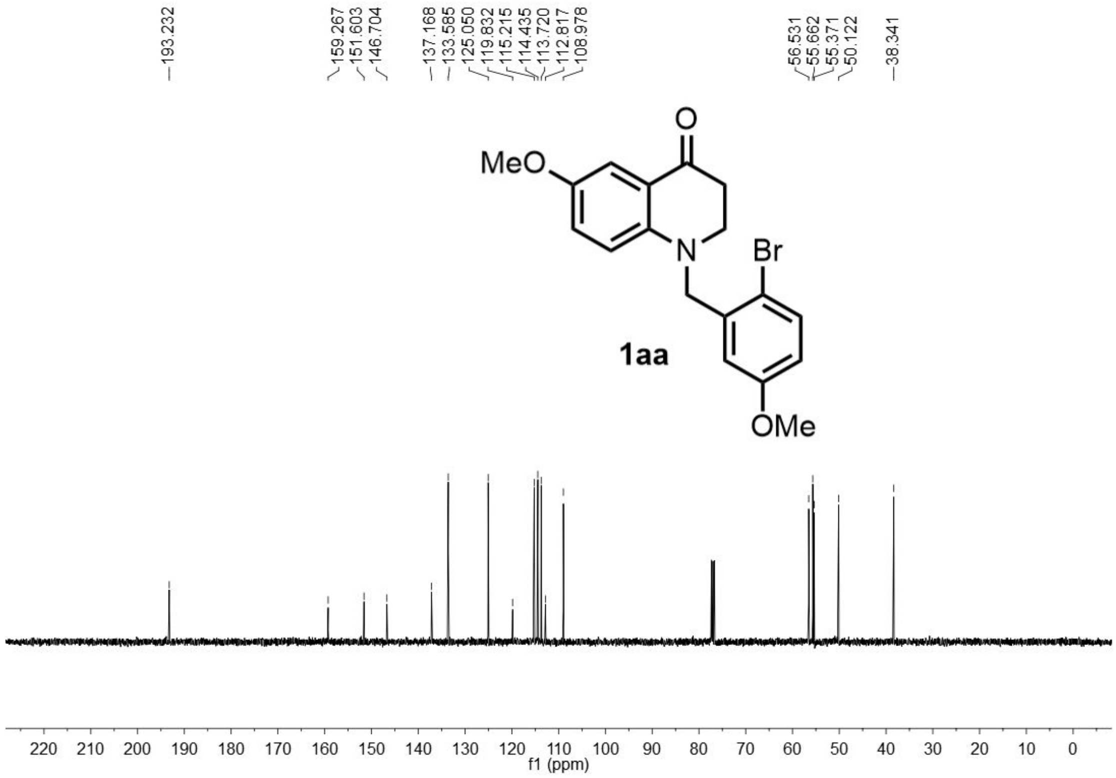

^{1}H NMR (400 MHz, $CDCl_3$) of compound **1ab:**

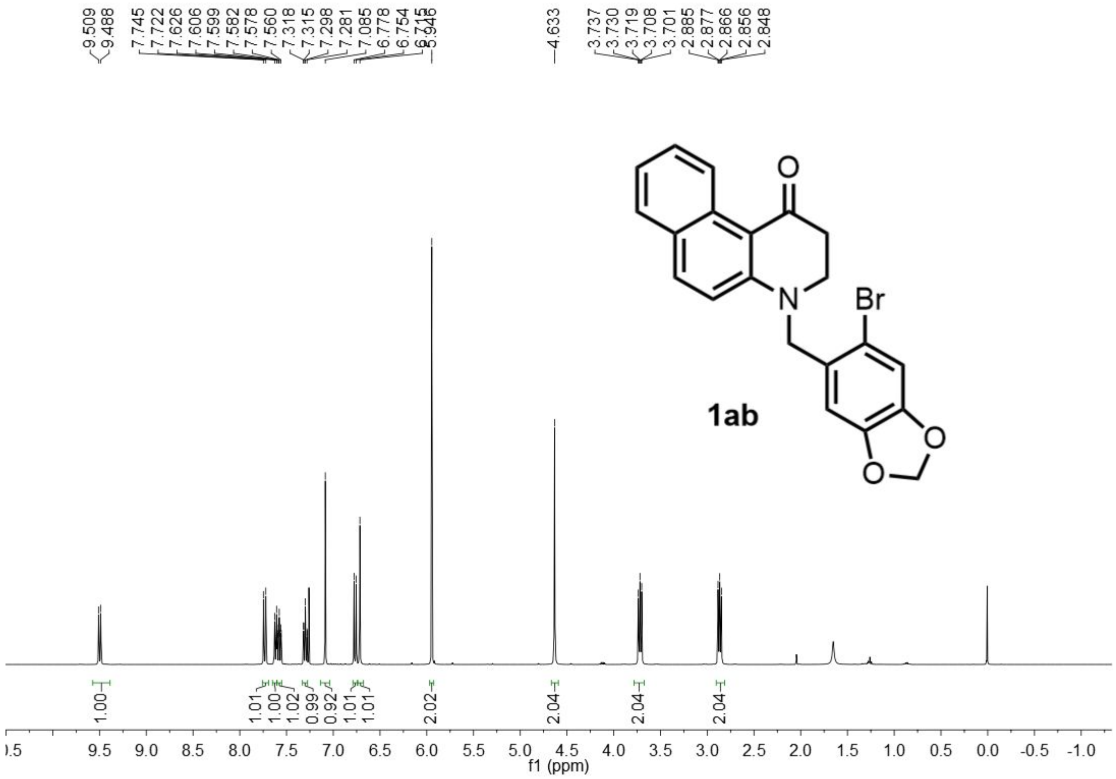

^{13}C NMR (100 MHz, $CDCl_3$) of compound **1ab:**

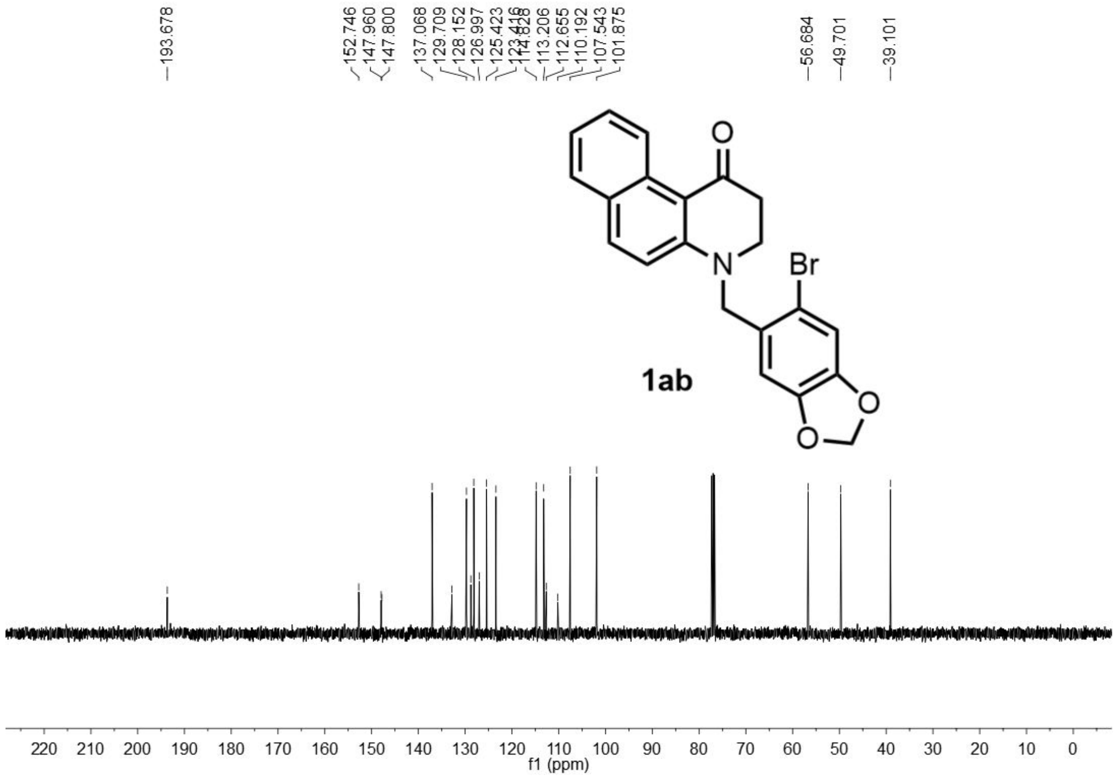

^1H NMR (400 MHz, $CDCl_3$) of compound **1ac:**

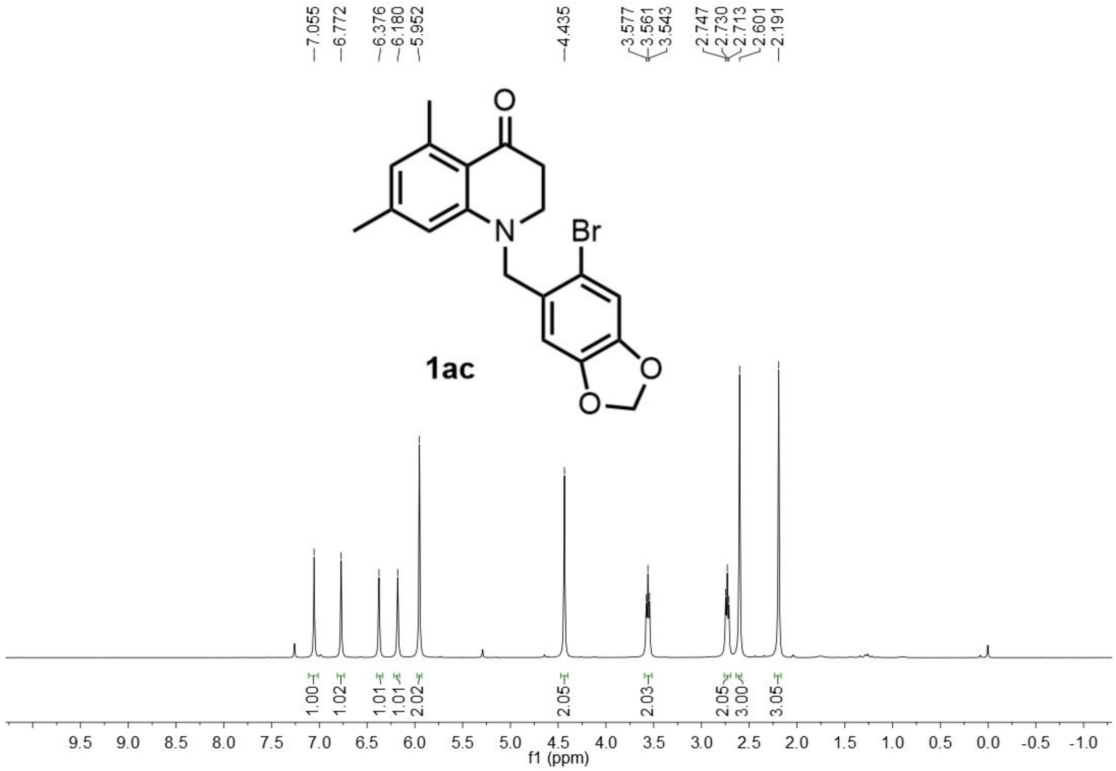

^{13}C NMR (100 MHz, $CDCl_3$) of compound **1ac:**

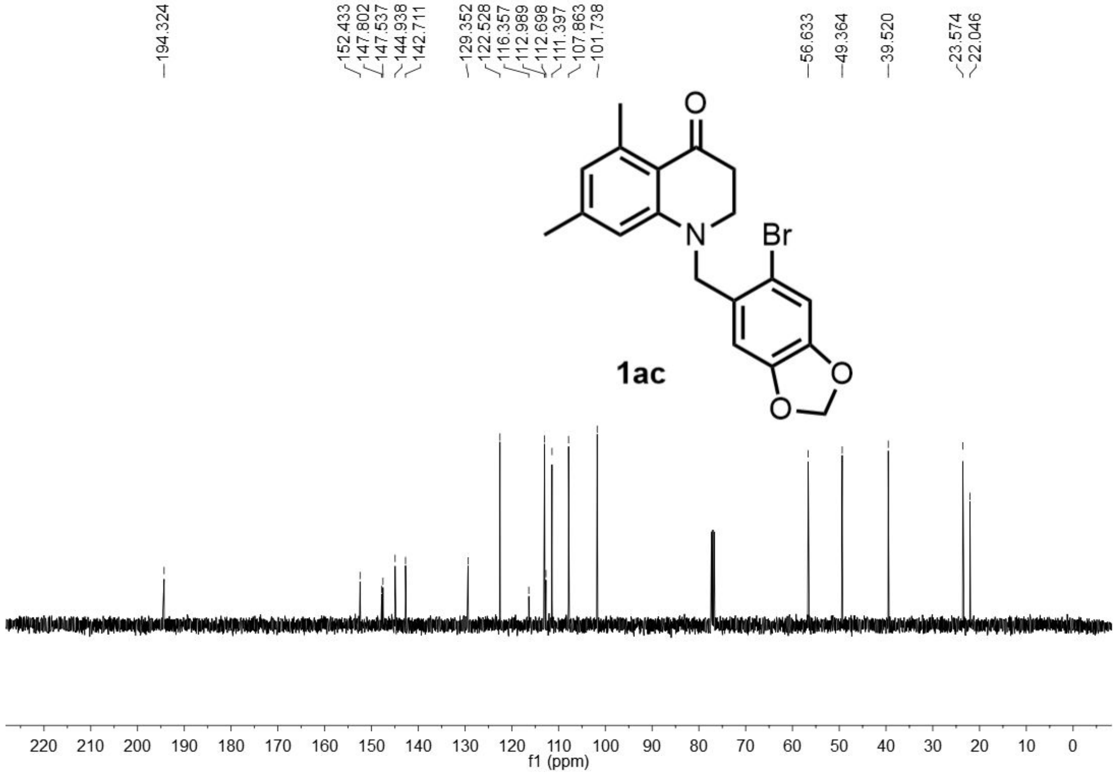

^{1}H NMR (400 MHz, $CDCl_3$) of compound **1ad:**

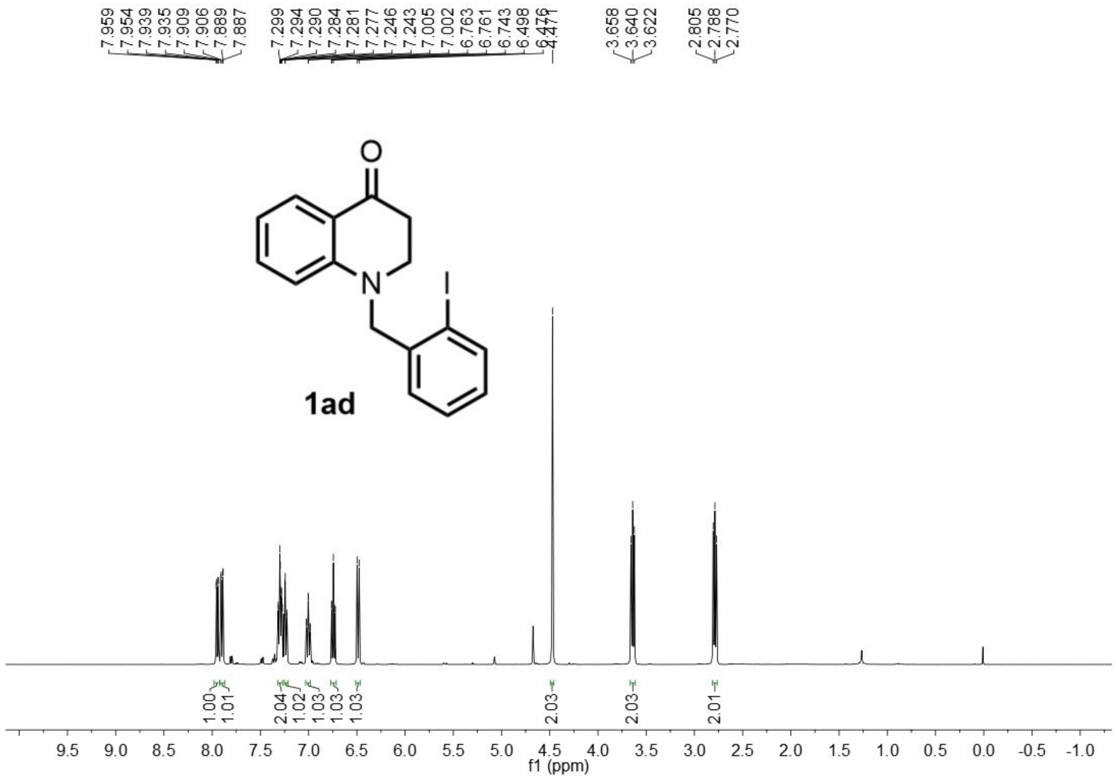

^{13}C NMR (100 MHz, $CDCl_3$) of compound **1ad:**

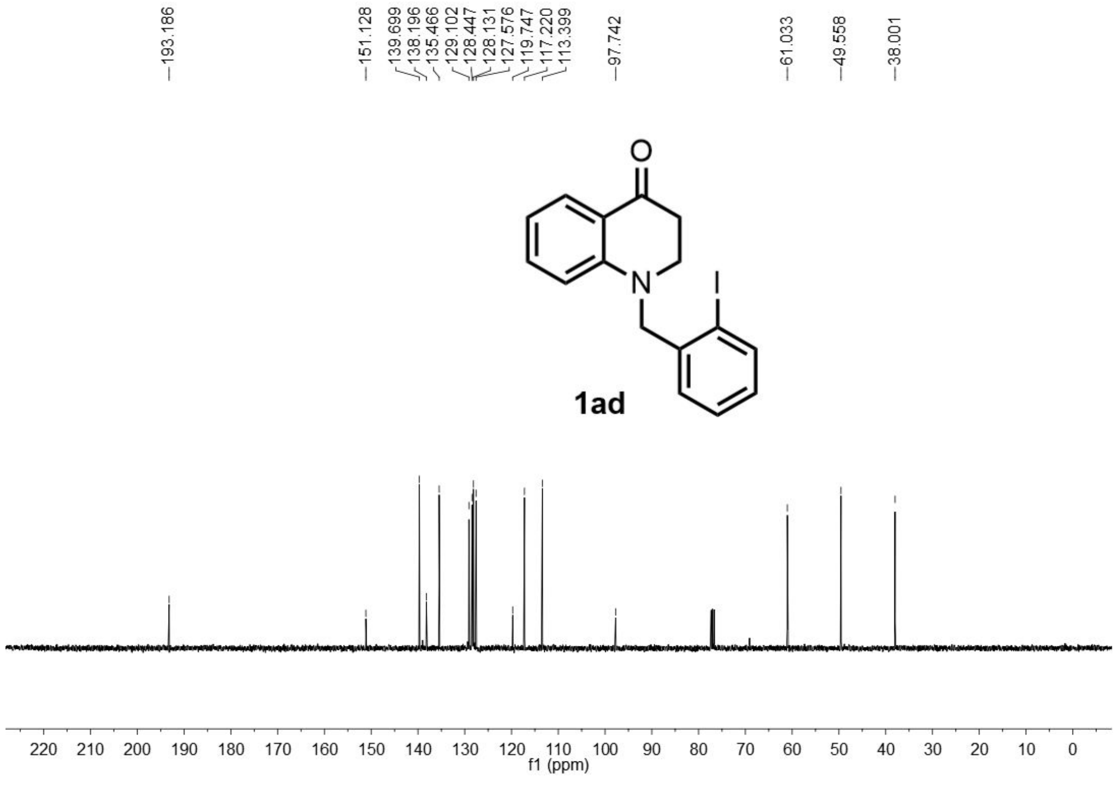

^{1}H NMR (400 MHz, $CDCl_3$) of compound **1ae:**

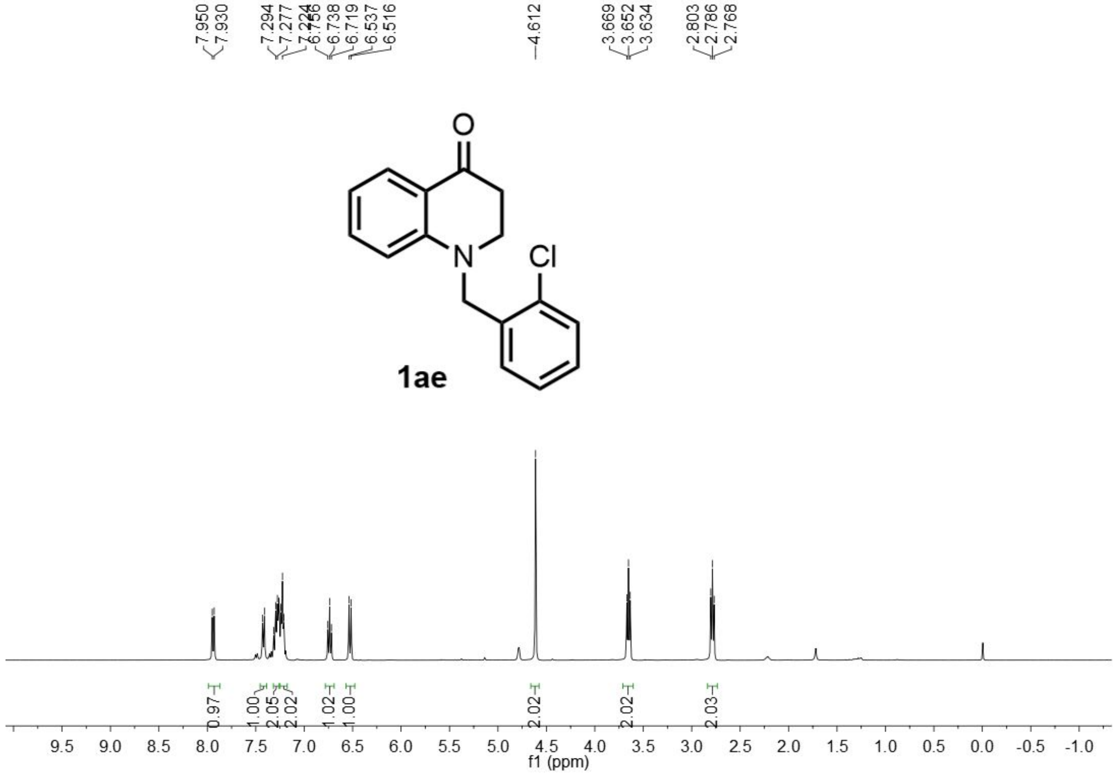

^{13}C NMR (100 MHz, $CDCl_3$) of compound **1ae:**

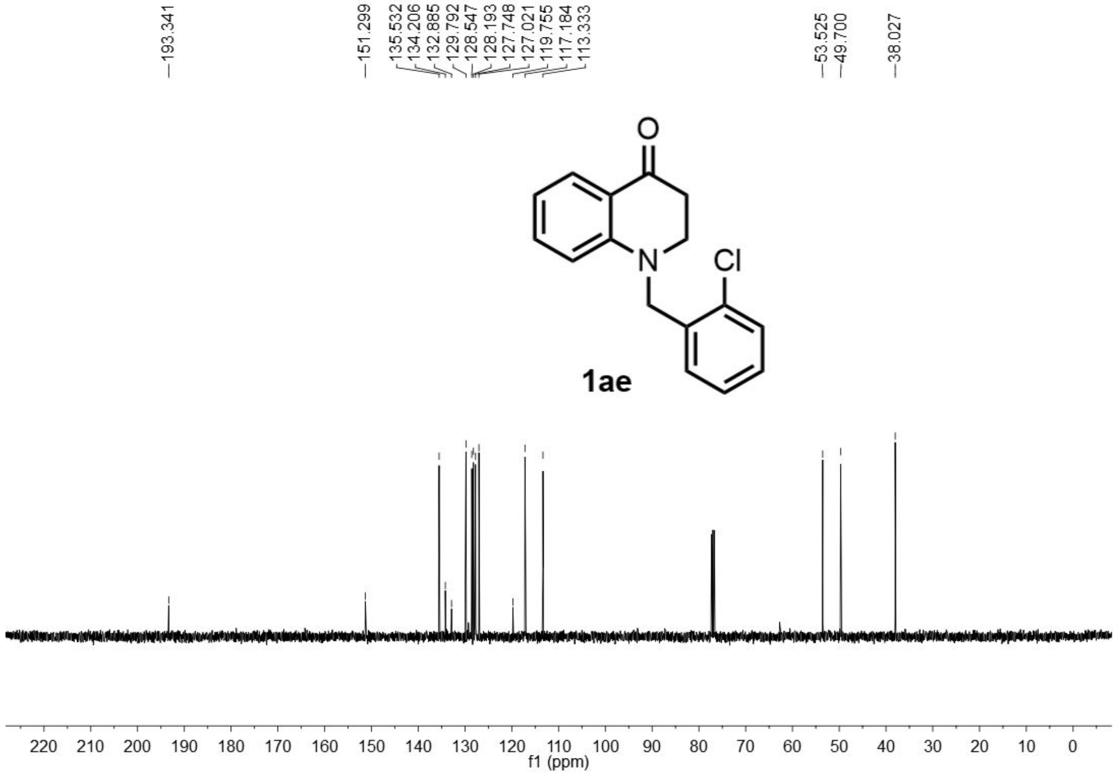

^{1}H NMR (400 MHz, $CDCl_3$) of compound **1af:**

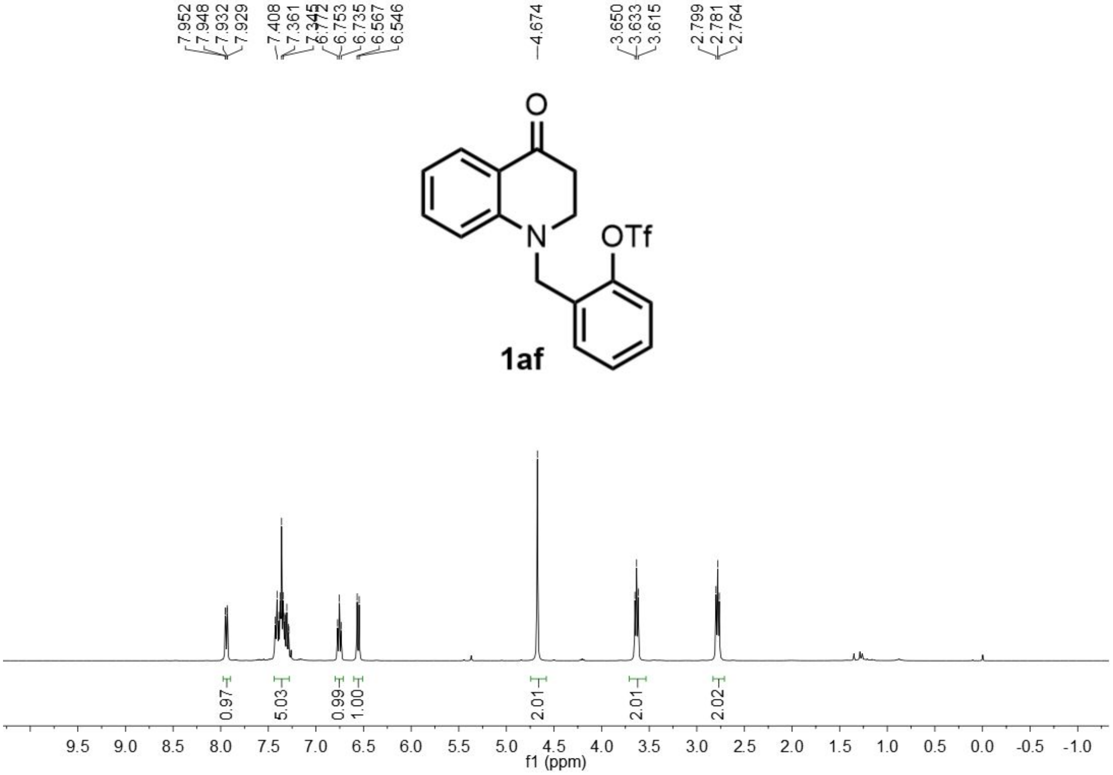

^{13}C NMR (100 MHz, $CDCl_3$) of compound **1af:**

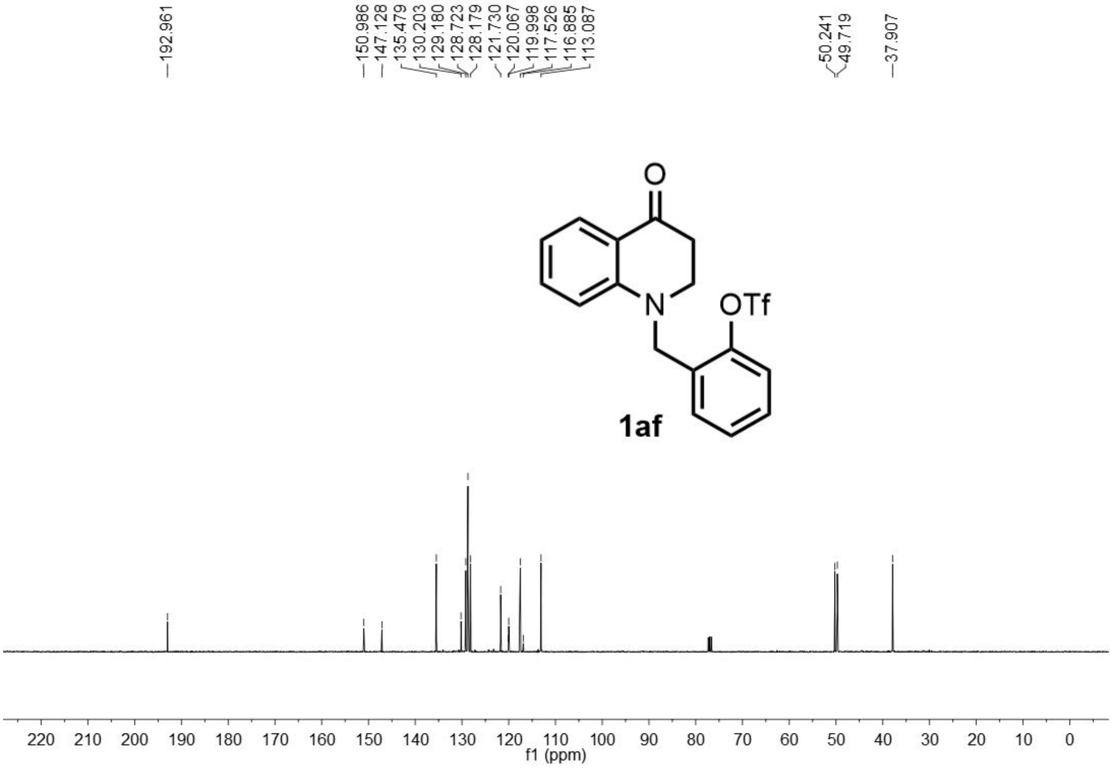

^{19}F NMR (377 MHz, $CDCl_3$) of compound **1af:**

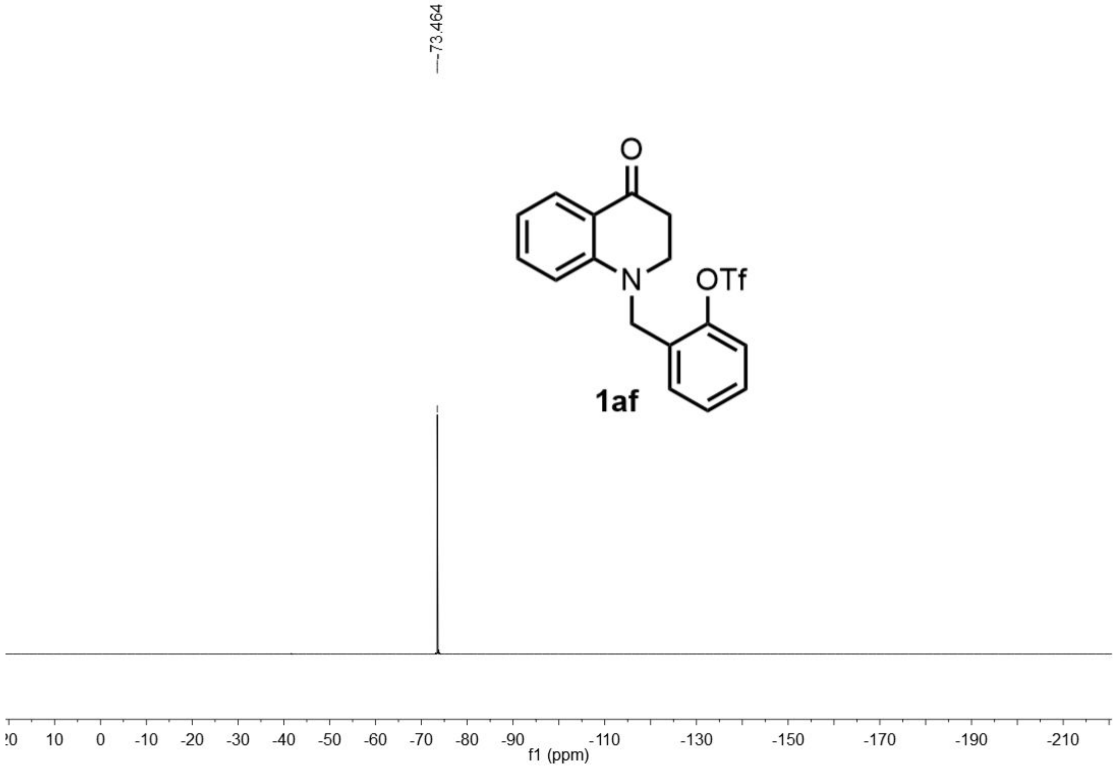

^{1}H NMR (400 MHz, $CDCl_3$) of compound **2a:**

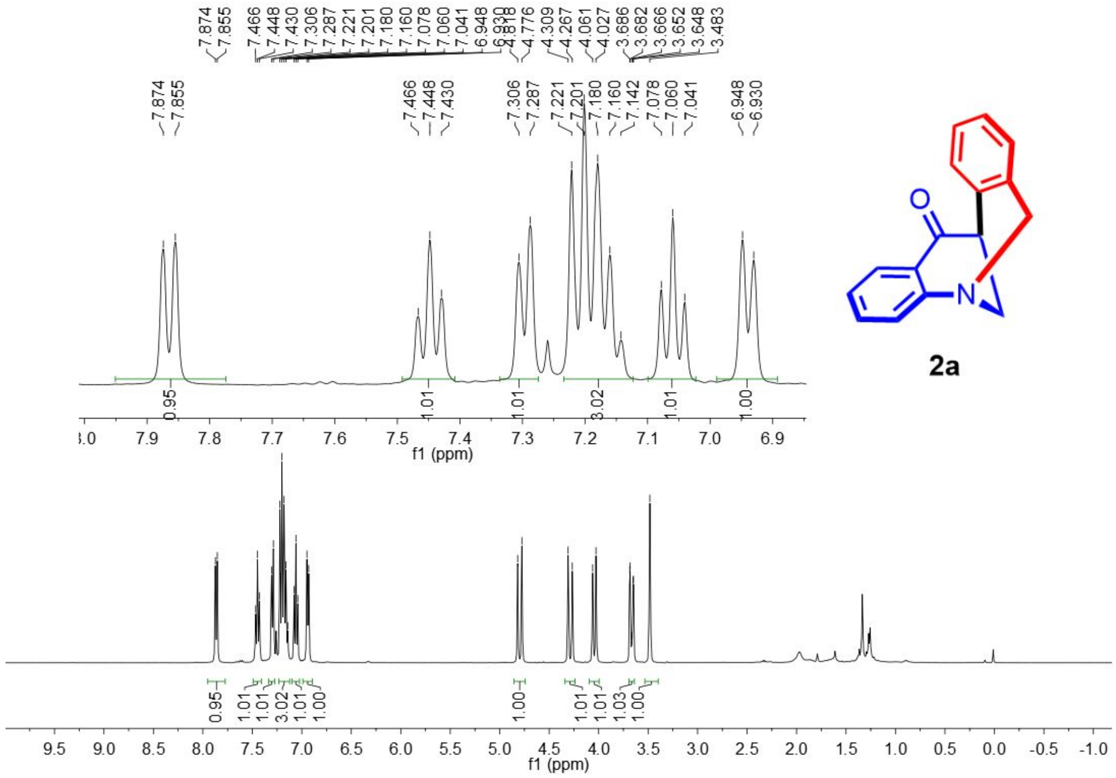

^{13}C NMR (100 MHz, $CDCl_3$) of compound **2a:**

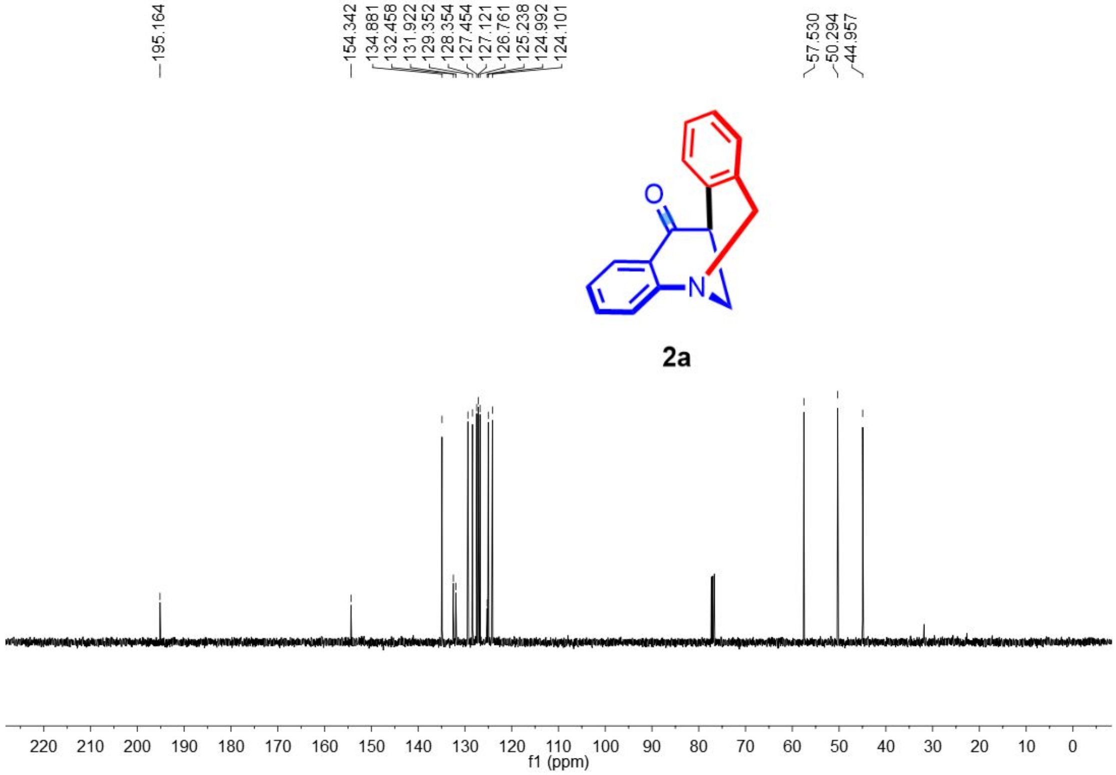

^{1}H NMR (400 MHz, $CDCl_3$) of compound **2b:**

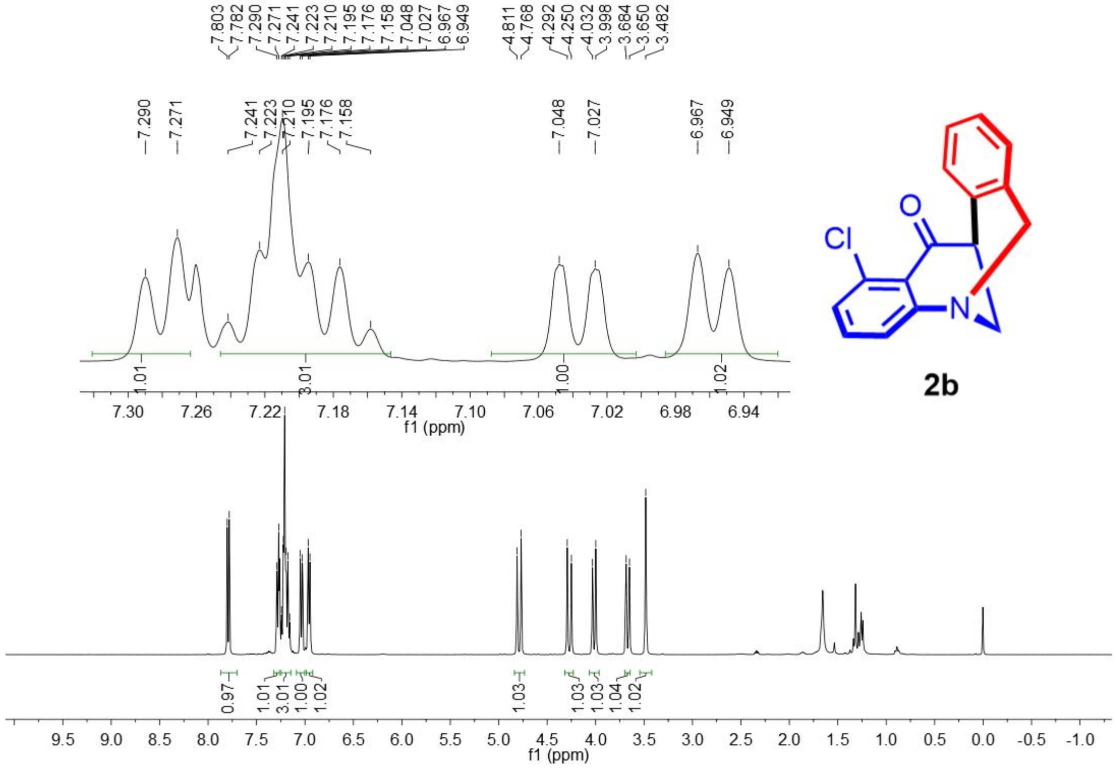

^{13}C NMR (100 MHz, $CDCl_3$) of compound **2b:**

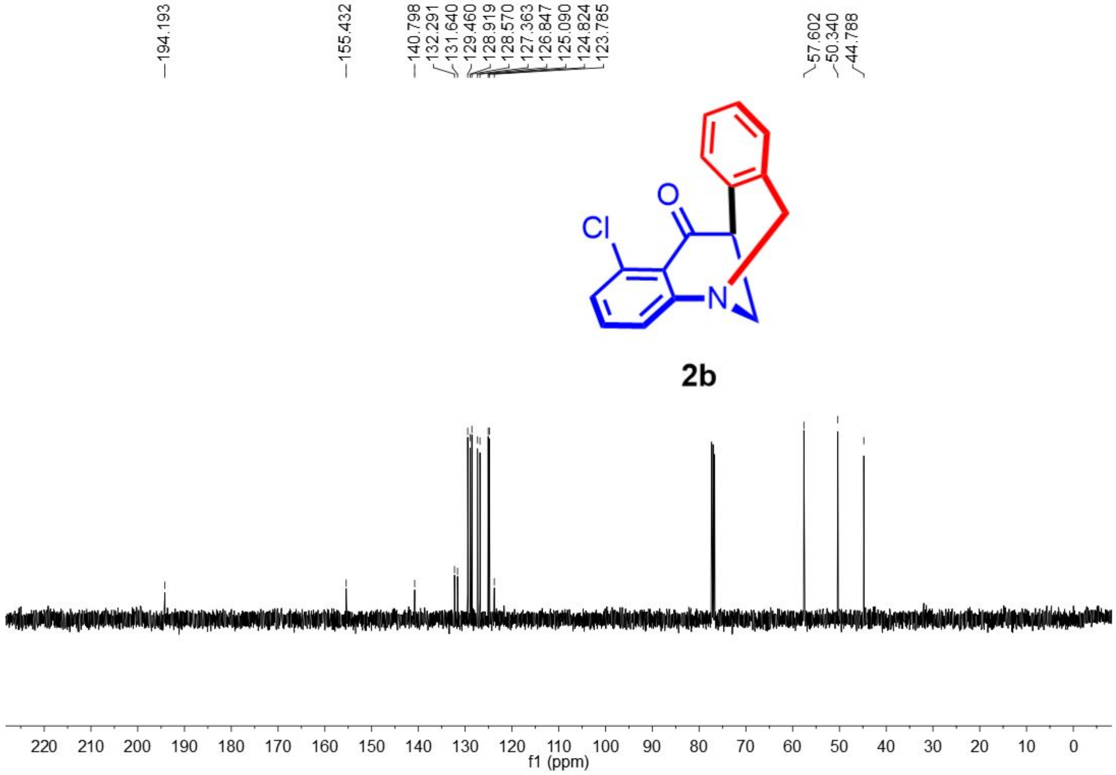

^{1}H NMR (400 MHz, $CDCl_3$) of compound **2c:**

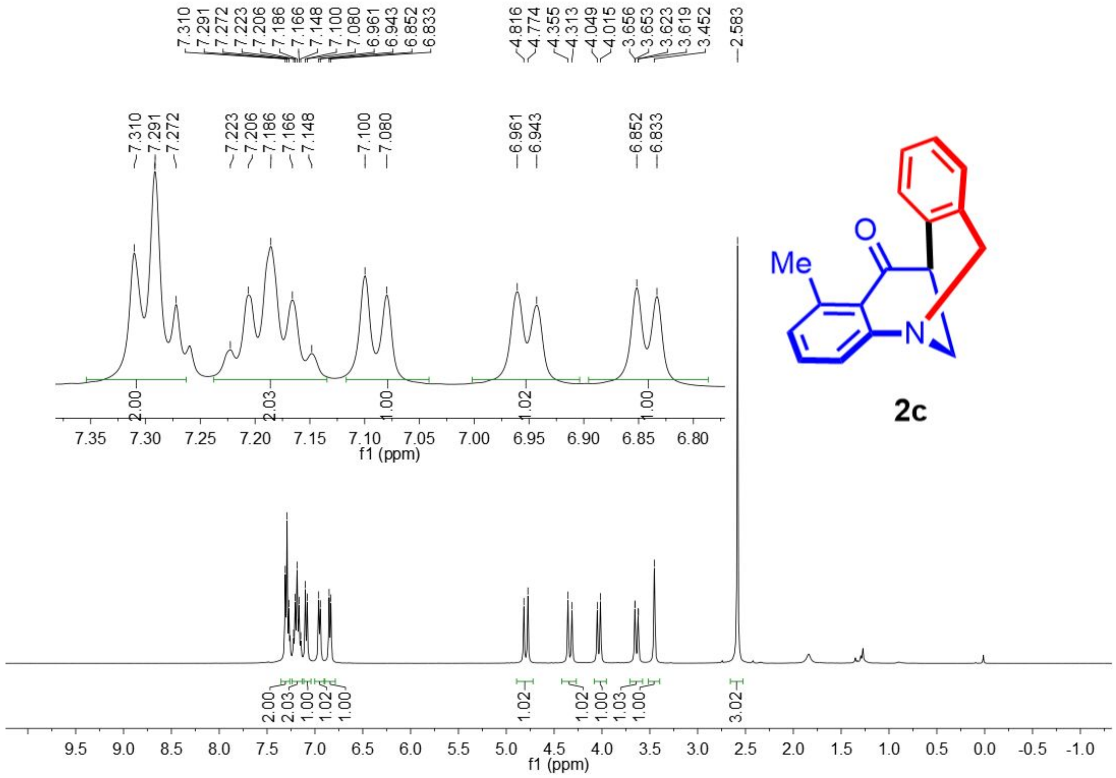

^{13}C NMR (100 MHz, $CDCl_3$) of compound **2c:**

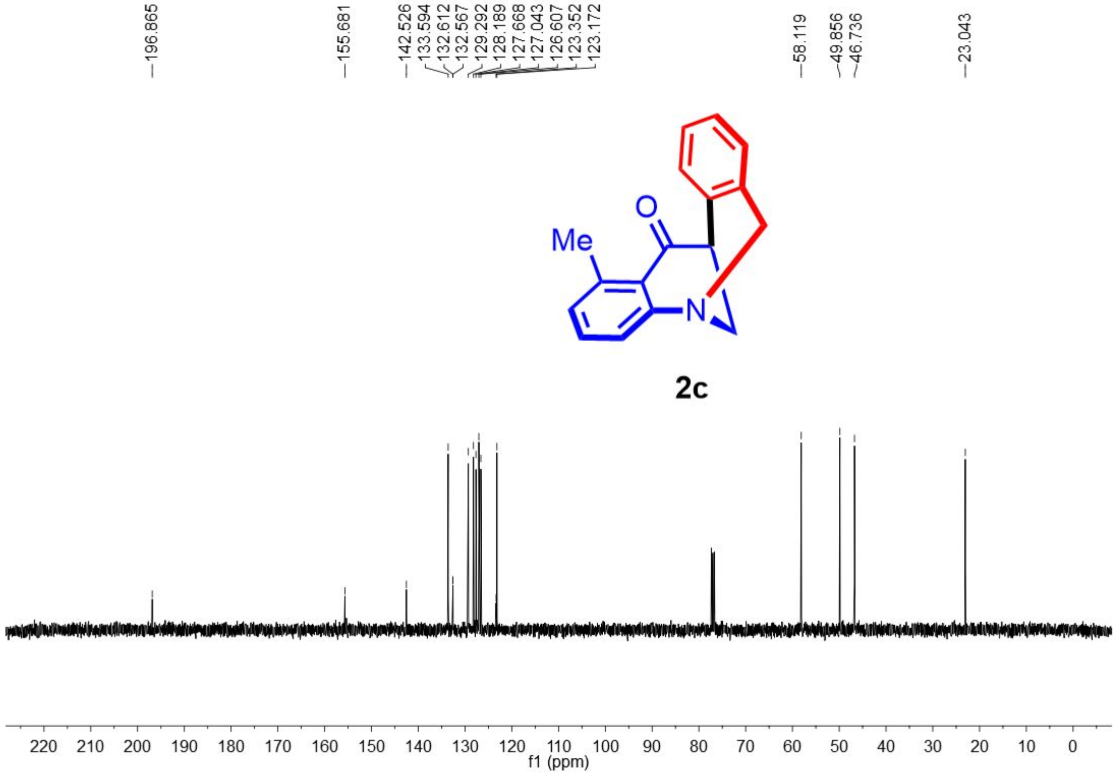

^{1}H NMR (400 MHz, $CDCl_3$) of compound **2d:**

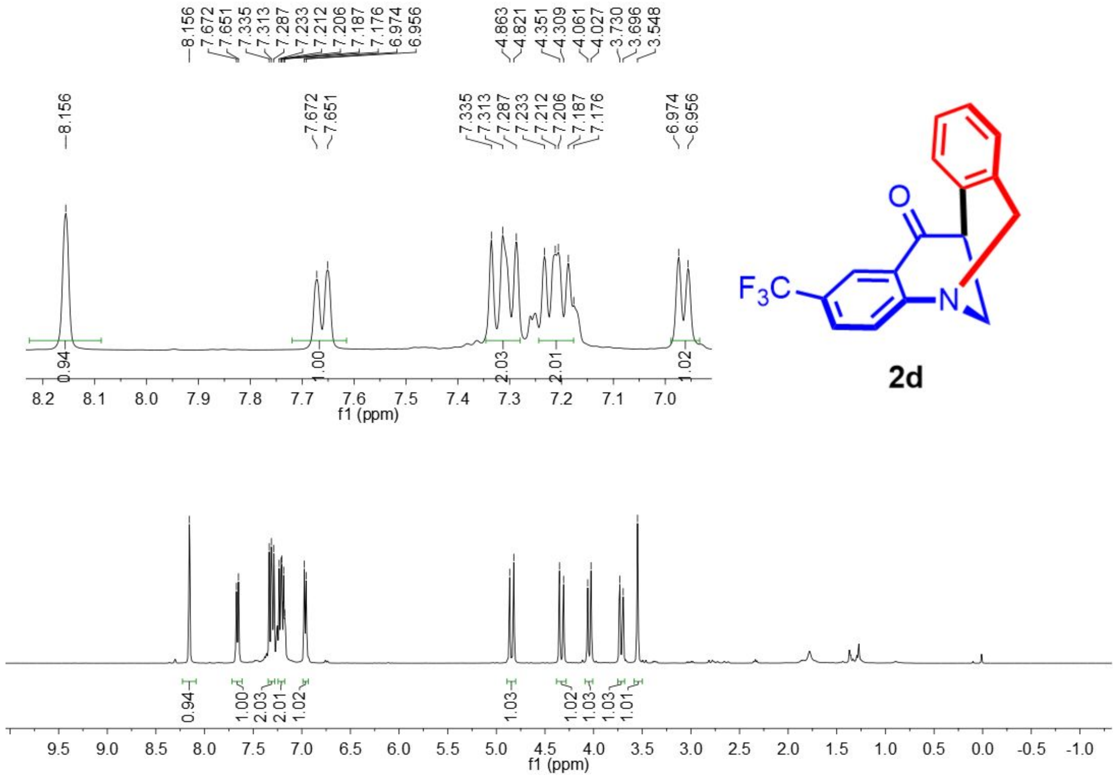

^{13}C NMR (100 MHz, $CDCl_3$) of compound **2d:**

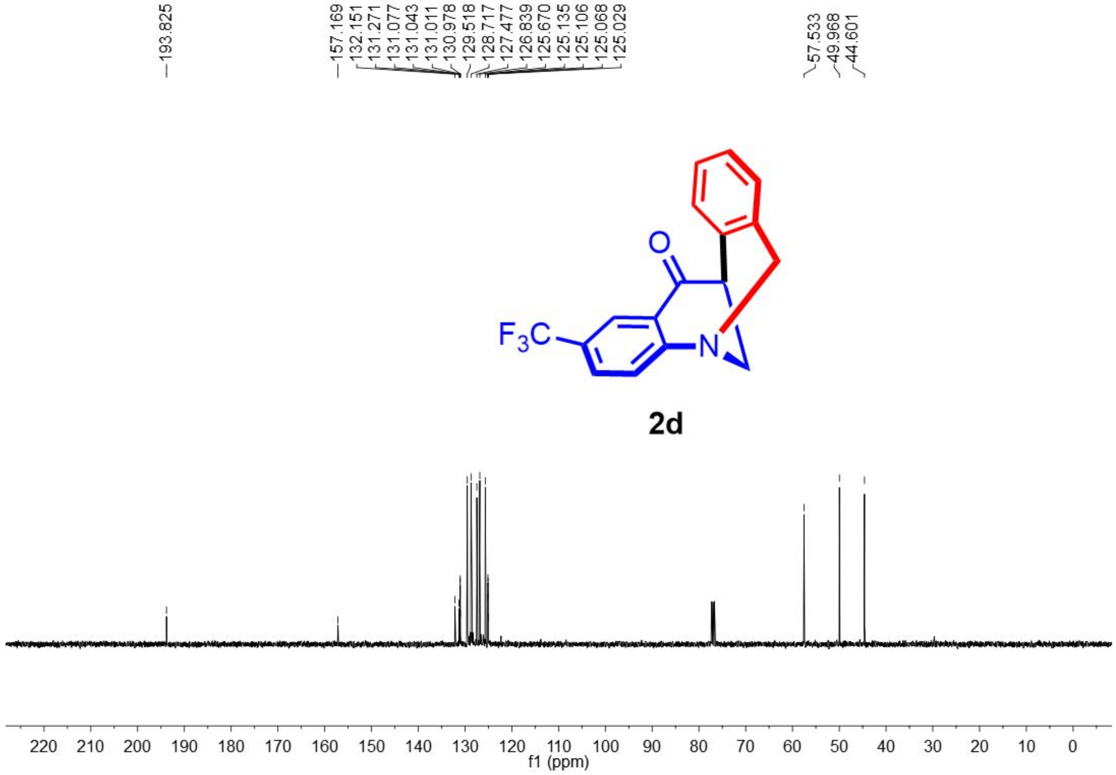

^{19}F NMR (377 MHz, $CDCl_3$) of compound **2d:**

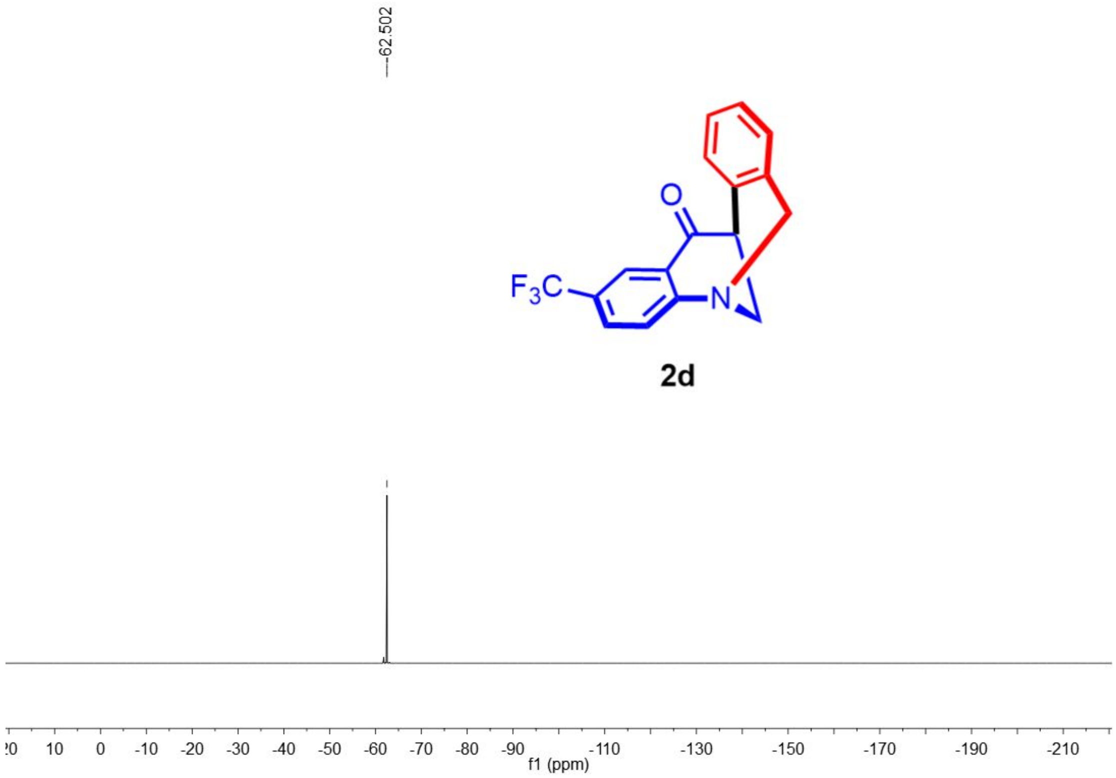

^{1}H NMR (400 MHz, $CDCl_3$) of compound **2e:**

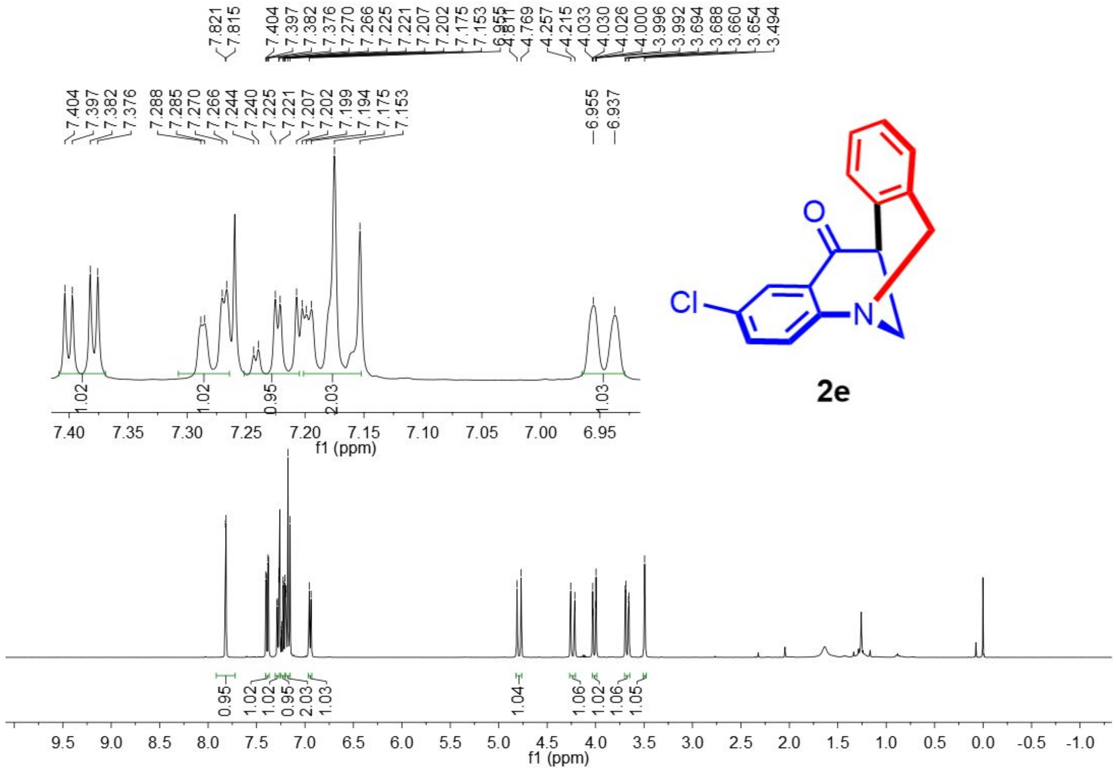

^{13}C NMR (100 MHz, $CDCl_3$) of compound **2e:**

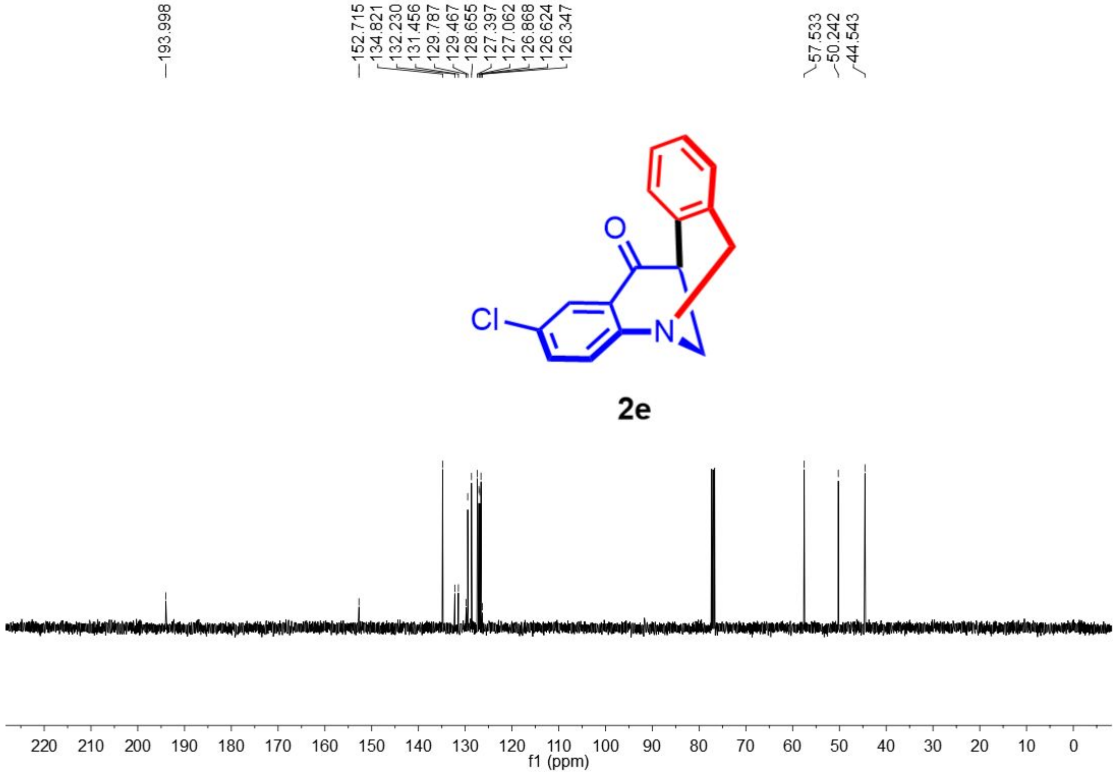

^1H NMR (400 MHz, $CDCl_3$) of compound **2f:**

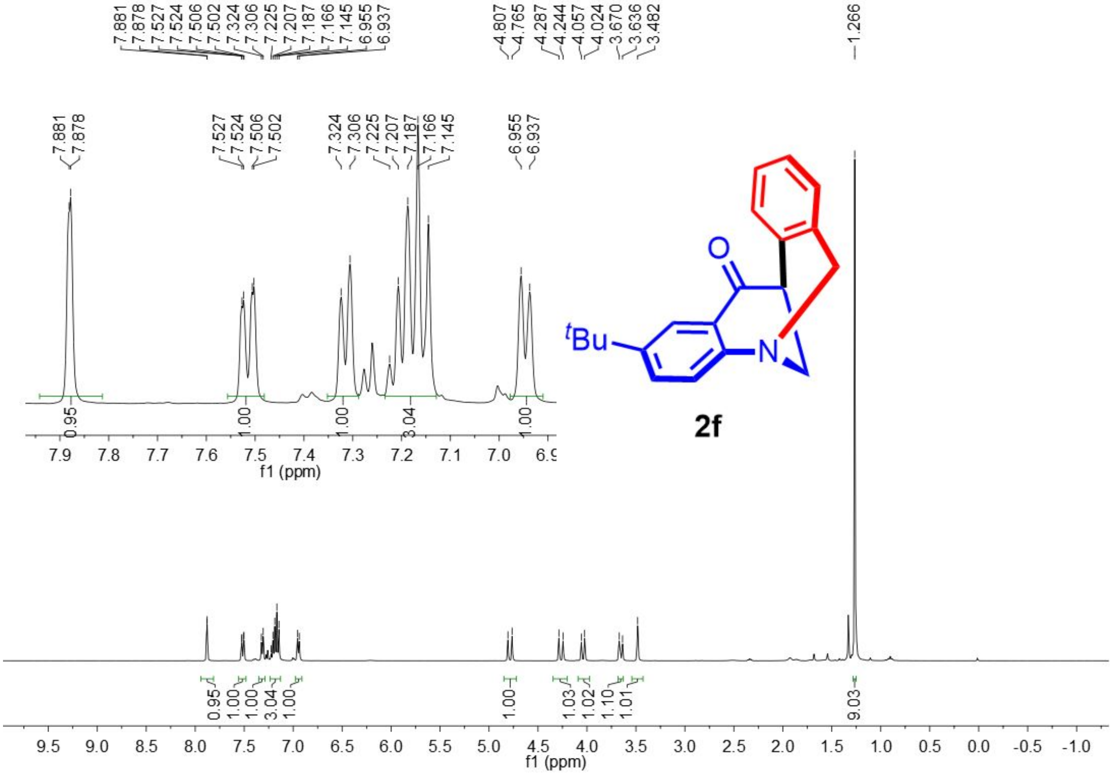

^{13}C NMR (100 MHz, $CDCl_3$) of compound **2f:**

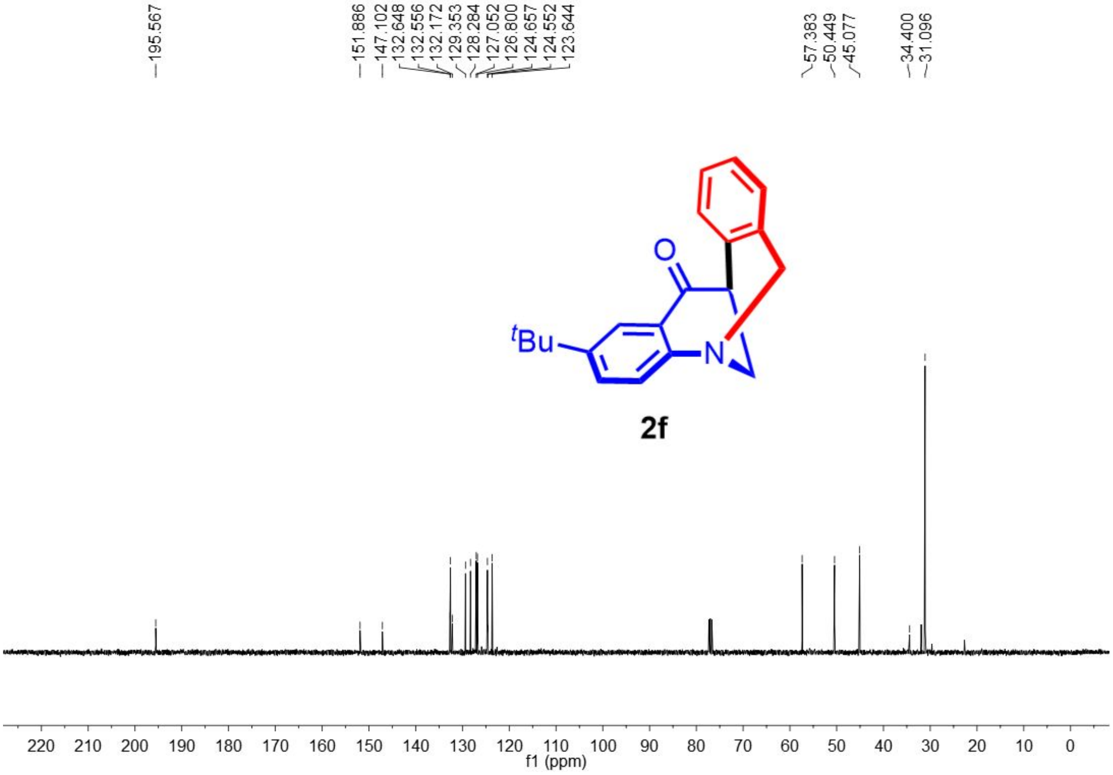

^{1}H NMR (400 MHz, $CDCl_3$) of compound **2g:**

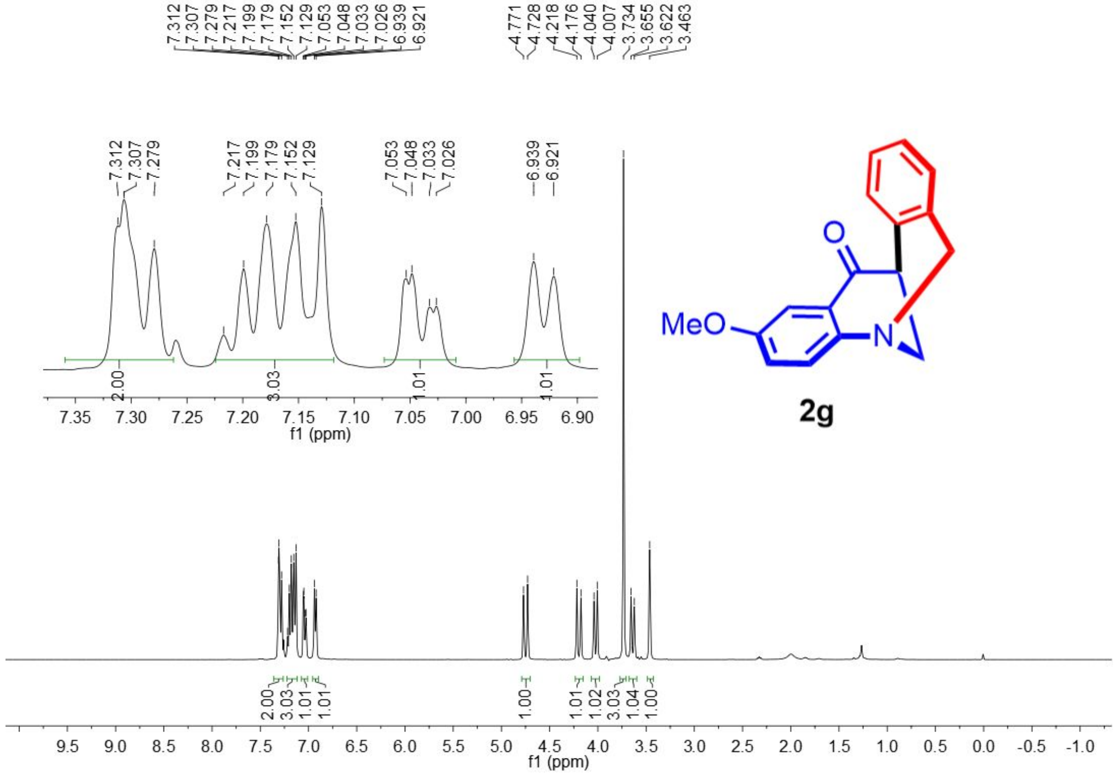

^{13}C NMR (100 MHz, $CDCl_3$) of compound **2g:**

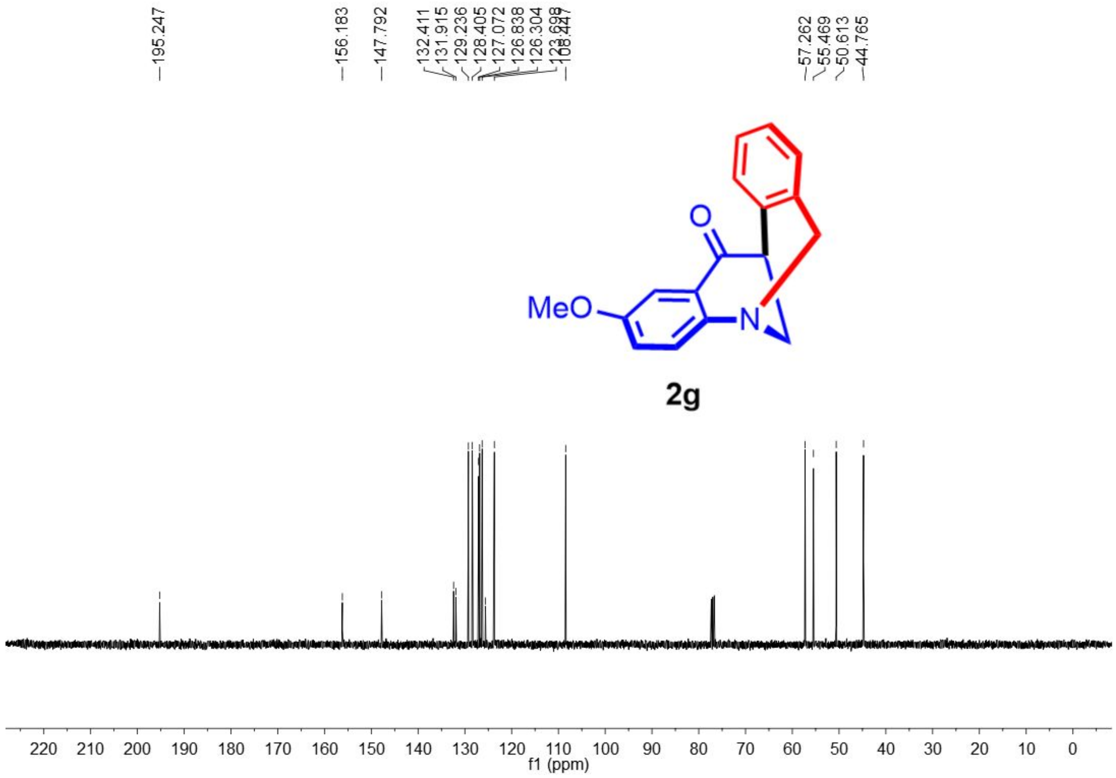

^{1}H NMR (400 MHz, $CDCl_3$) of compound **2h:**

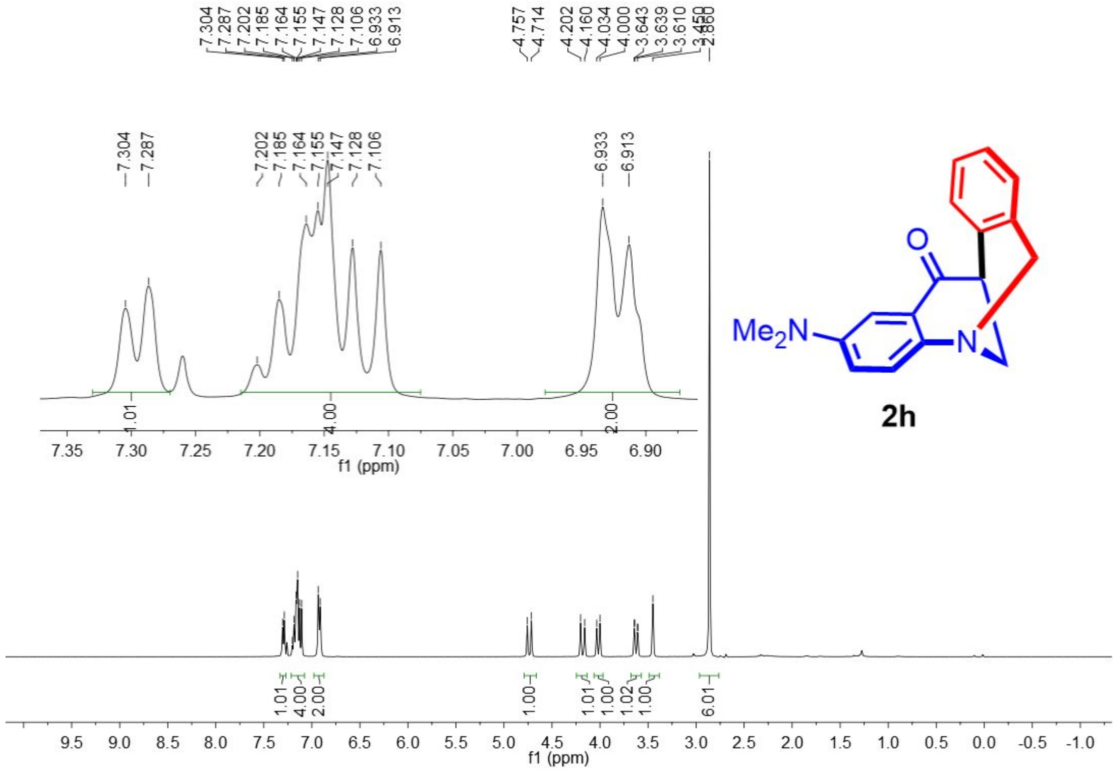

^{13}C NMR (100 MHz, $CDCl_3$) of compound **2h:**

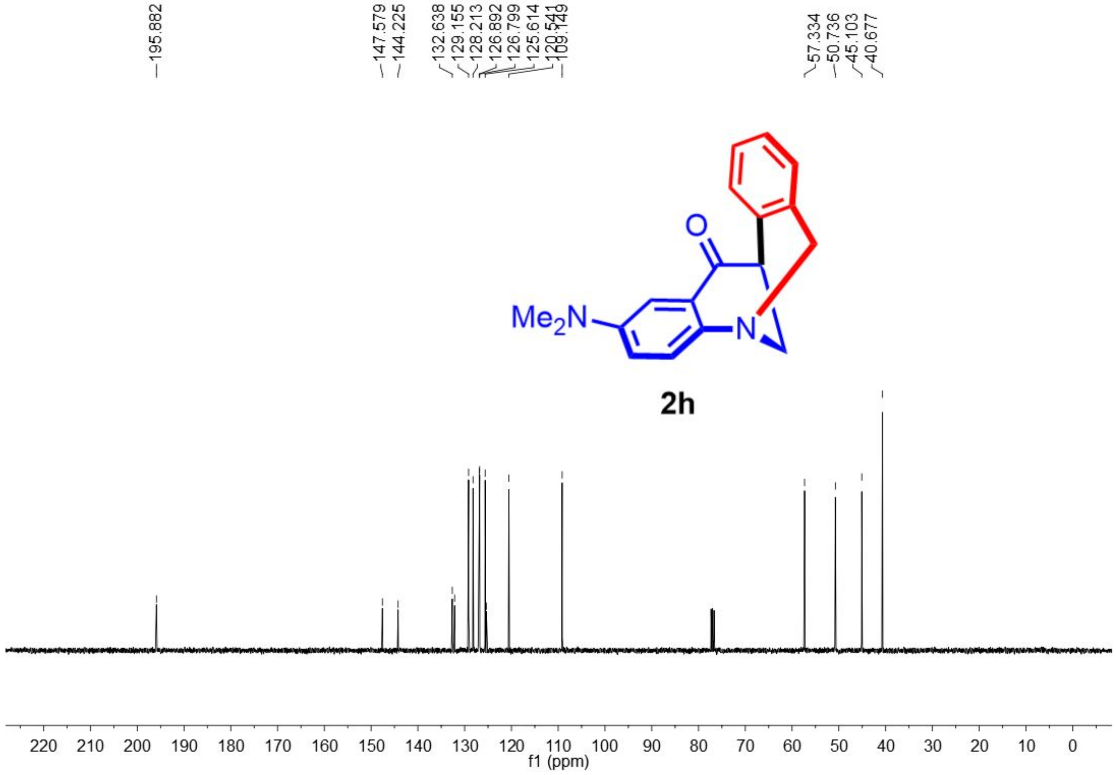

^{1}H NMR (400 MHz, $CDCl_3$) of compound **2i:**

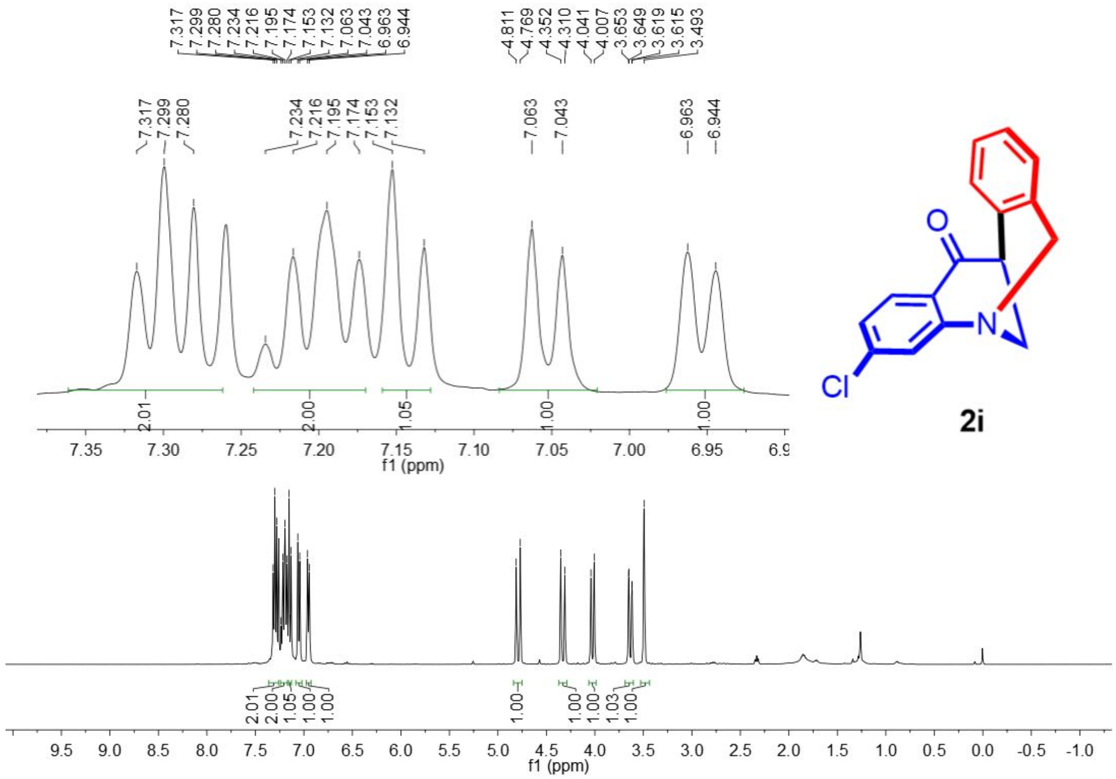

^{13}C NMR (100 MHz, $CDCl_3$) of compound **2i:**

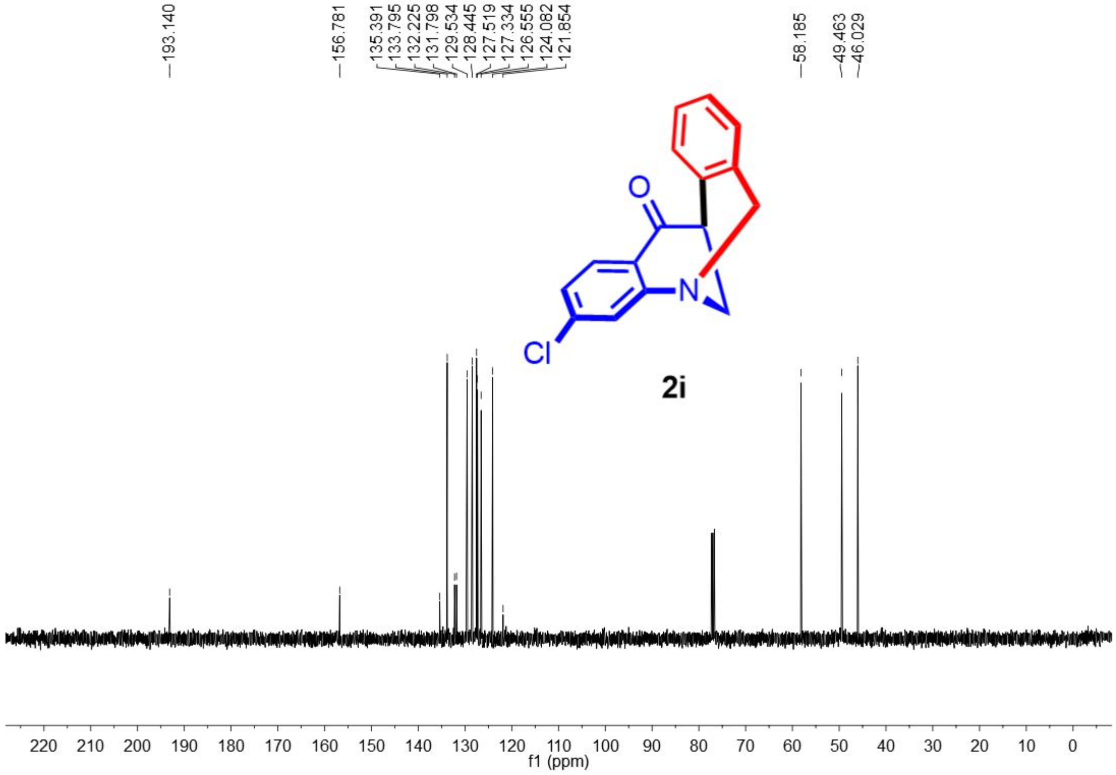

^{1}H NMR (400 MHz, $CDCl_3$) of compound **2j:**

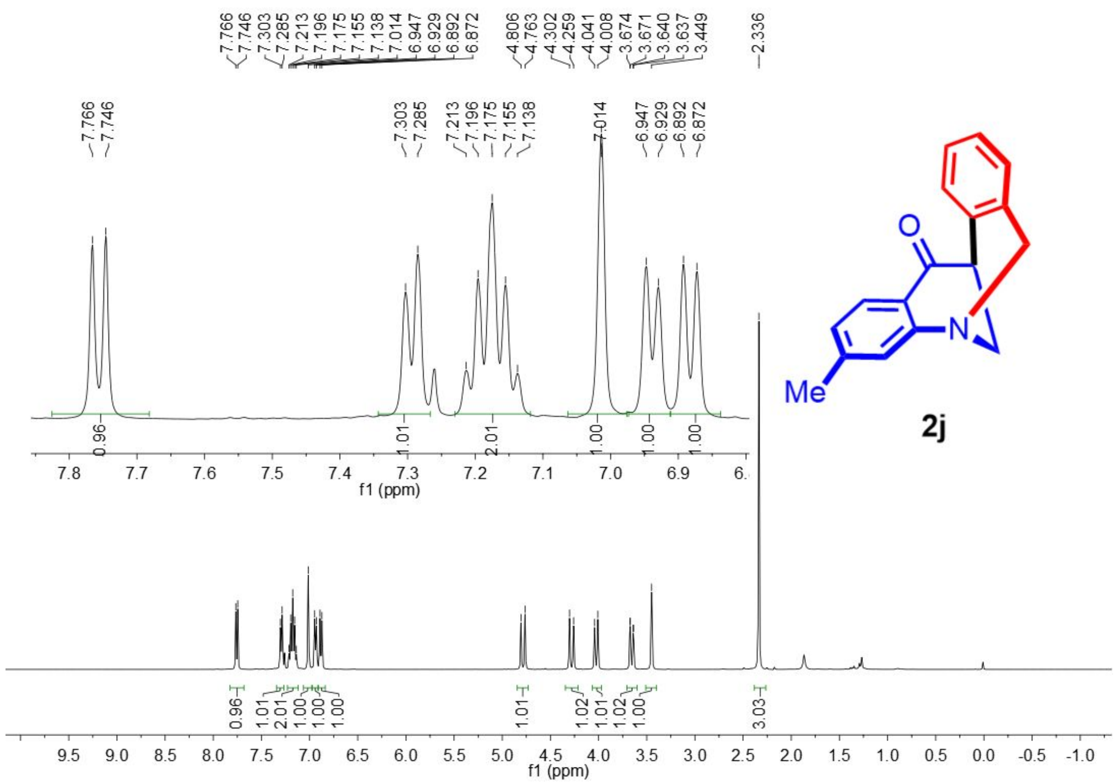

^{13}C NMR (100 MHz, $CDCl_3$) of compound **2j:**

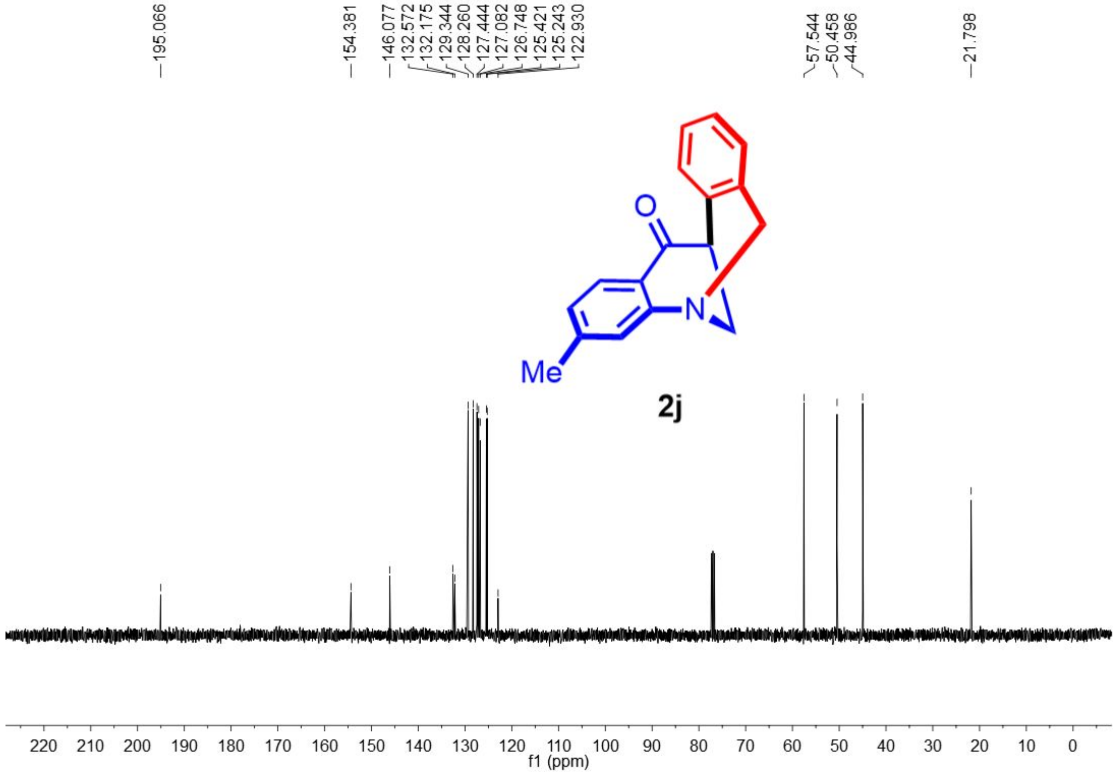

^{1}H NMR (400 MHz, $CDCl_3$) of compound **2k:**

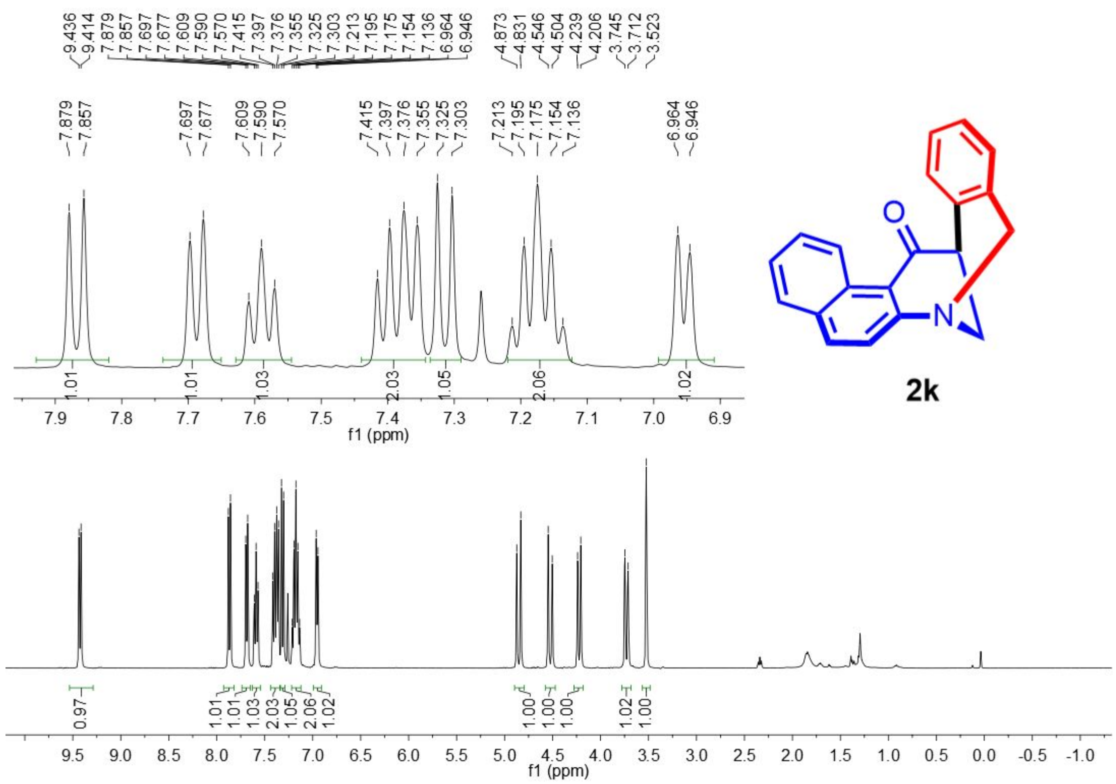

^{13}C NMR (100 MHz, $CDCl_3$) of compound **2k:**

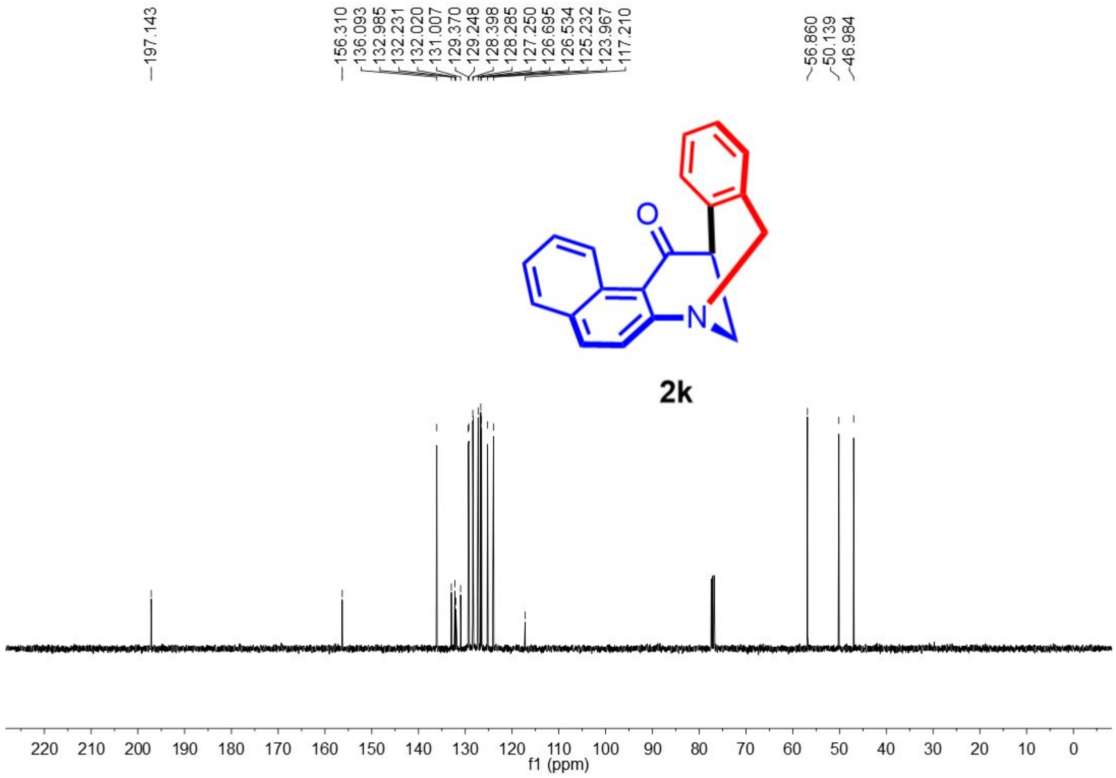

^{1}H NMR (400 MHz, $CDCl_3$) of compound **2l:**

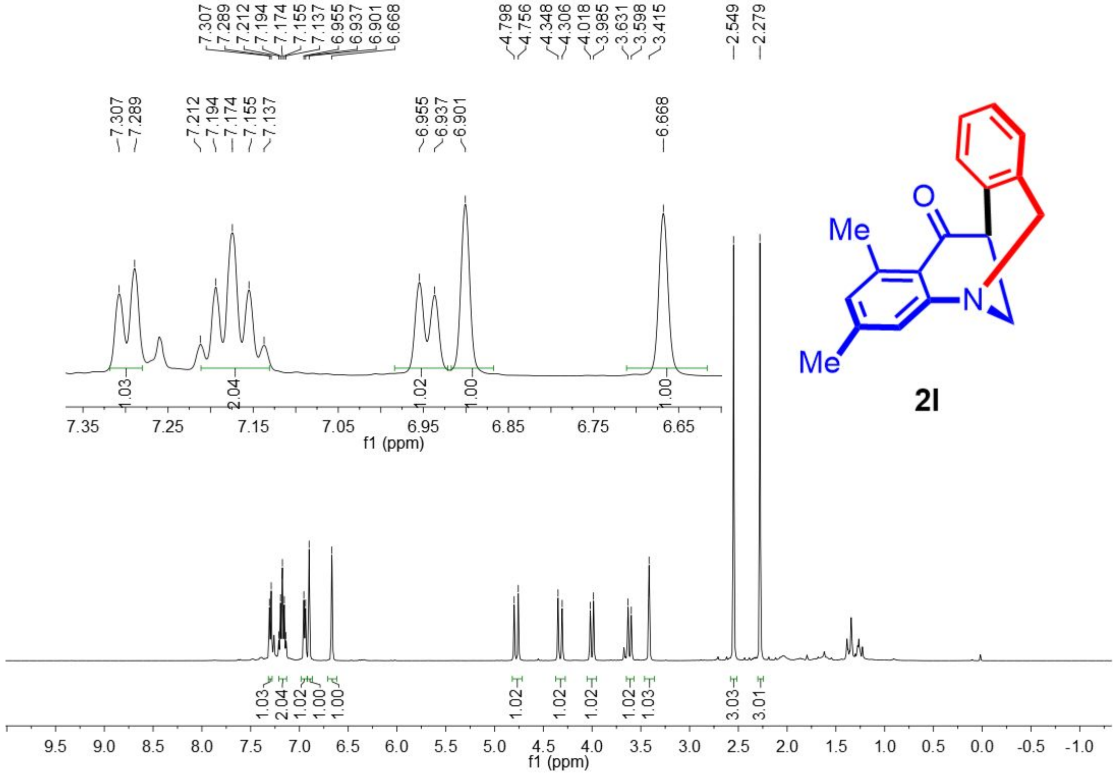

^{13}C NMR (100 MHz, $CDCl_3$) of compound **2l:**

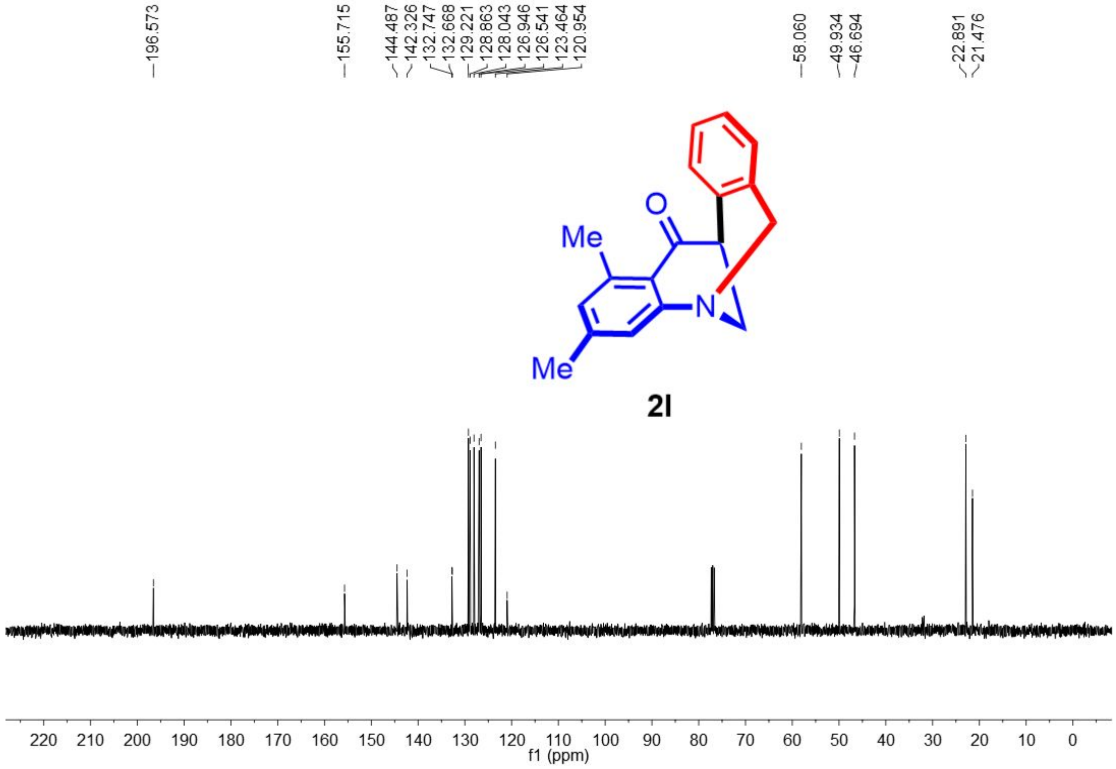

^{1}H NMR (400 MHz, $CDCl_3$) of compound **2m:**

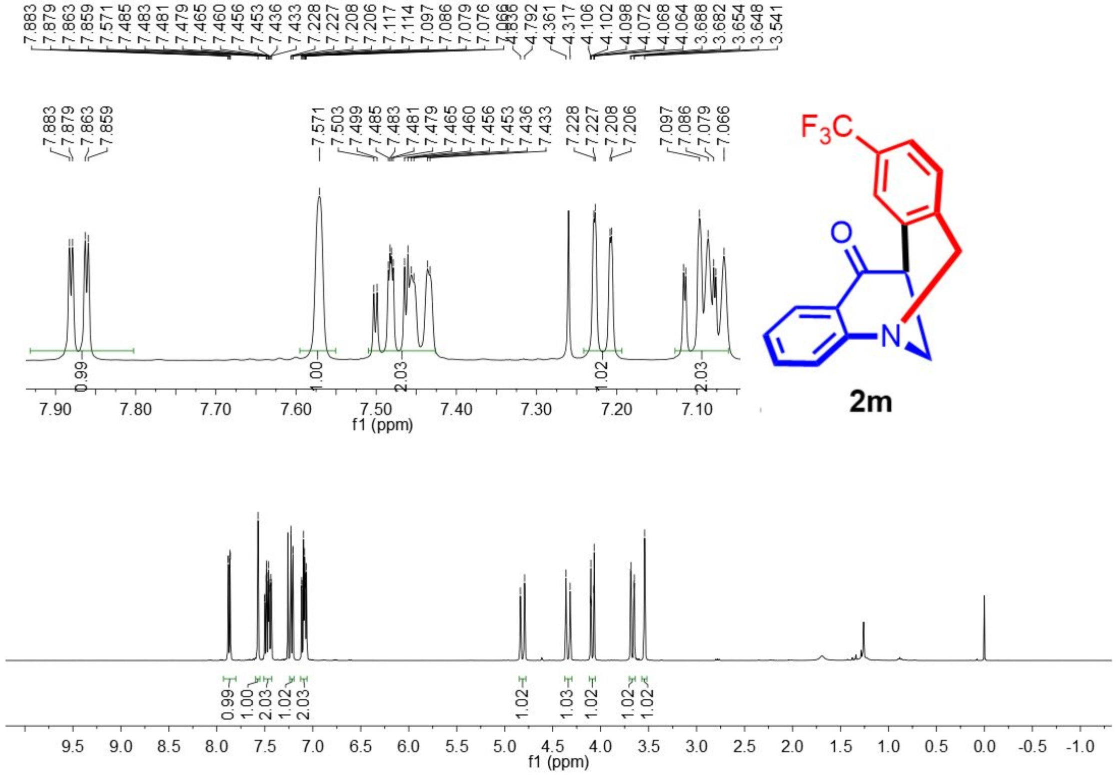

^{13}C NMR (100 MHz, $CDCl_3$) of compound **2m:**

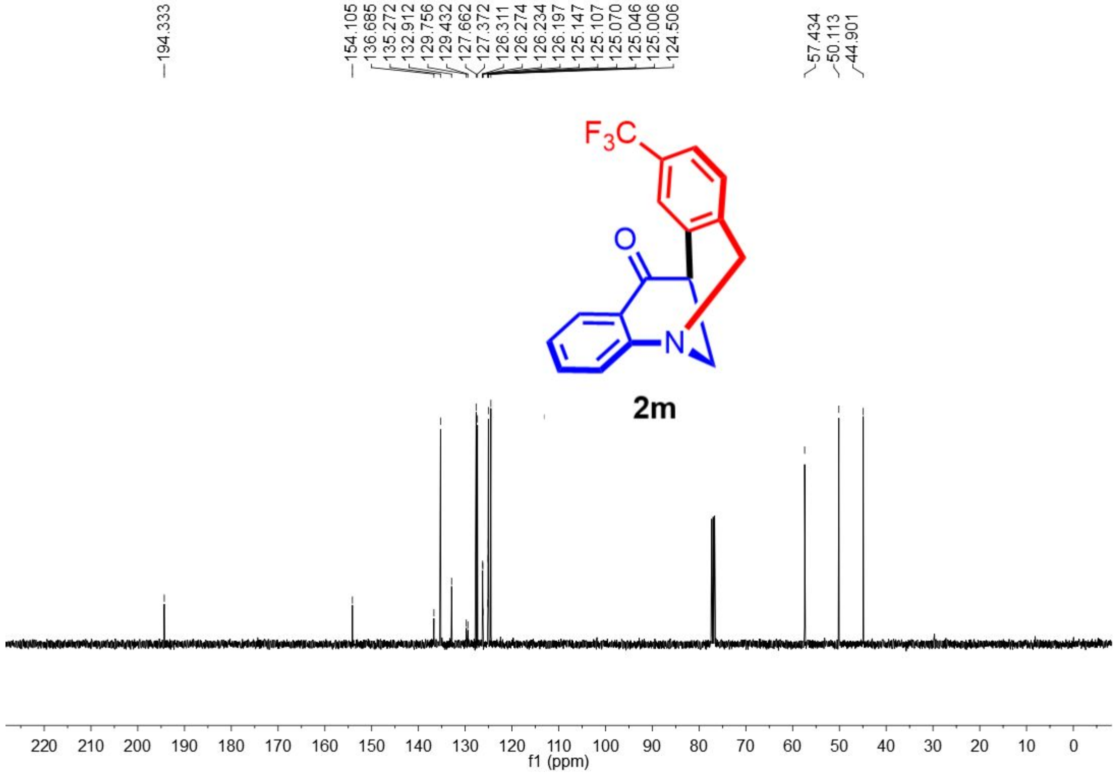

^{19}F NMR (377 MHz, $CDCl_3$) of compound **2m:**

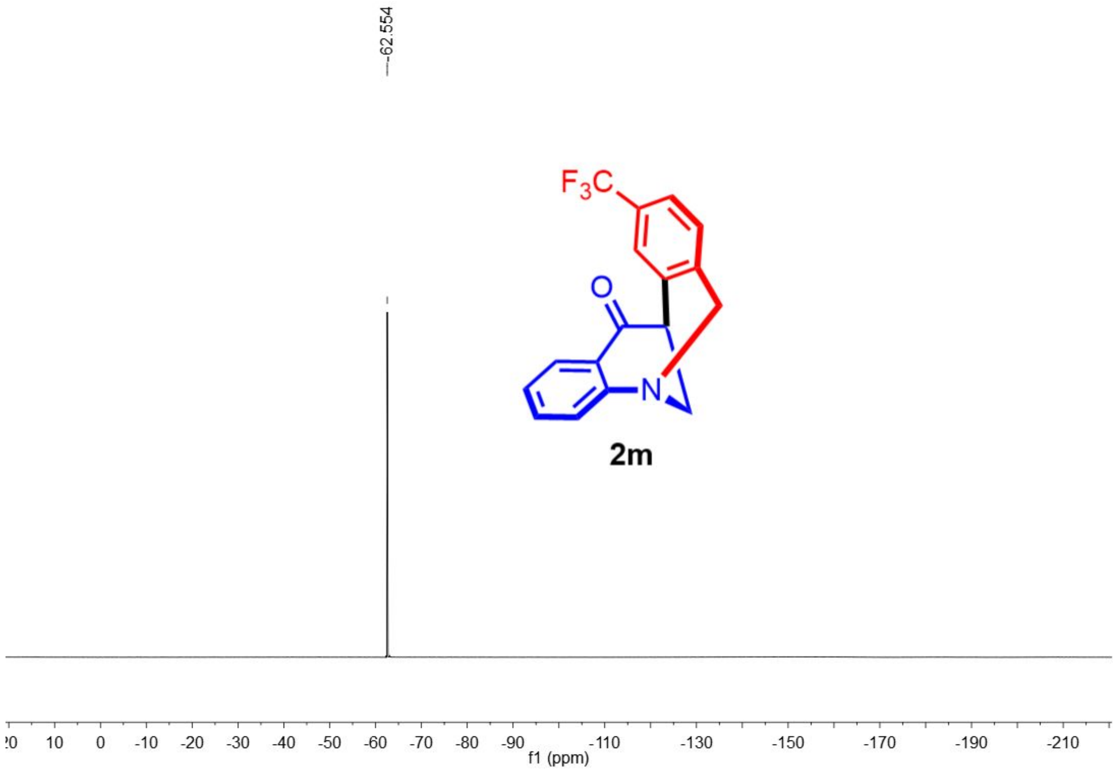

^{1}H NMR (400 MHz, $CDCl_3$) of compound **2n:**

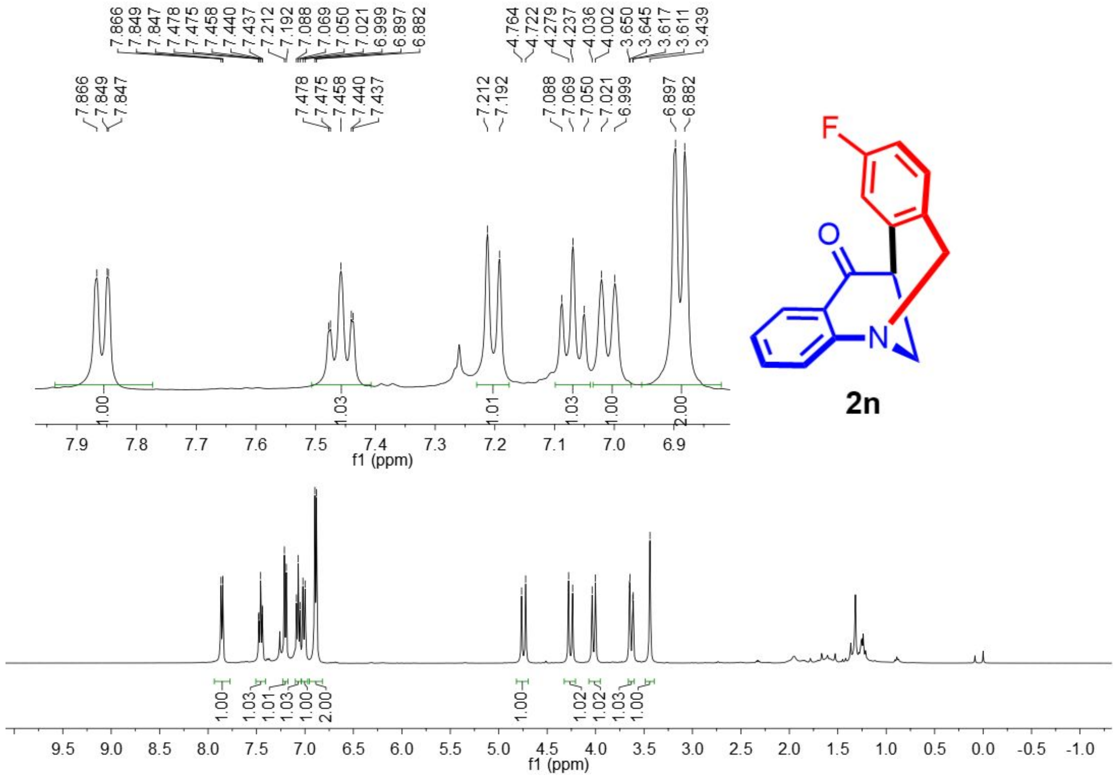

^{13}C NMR (100 MHz, $CDCl_3$) of compound **2n:**

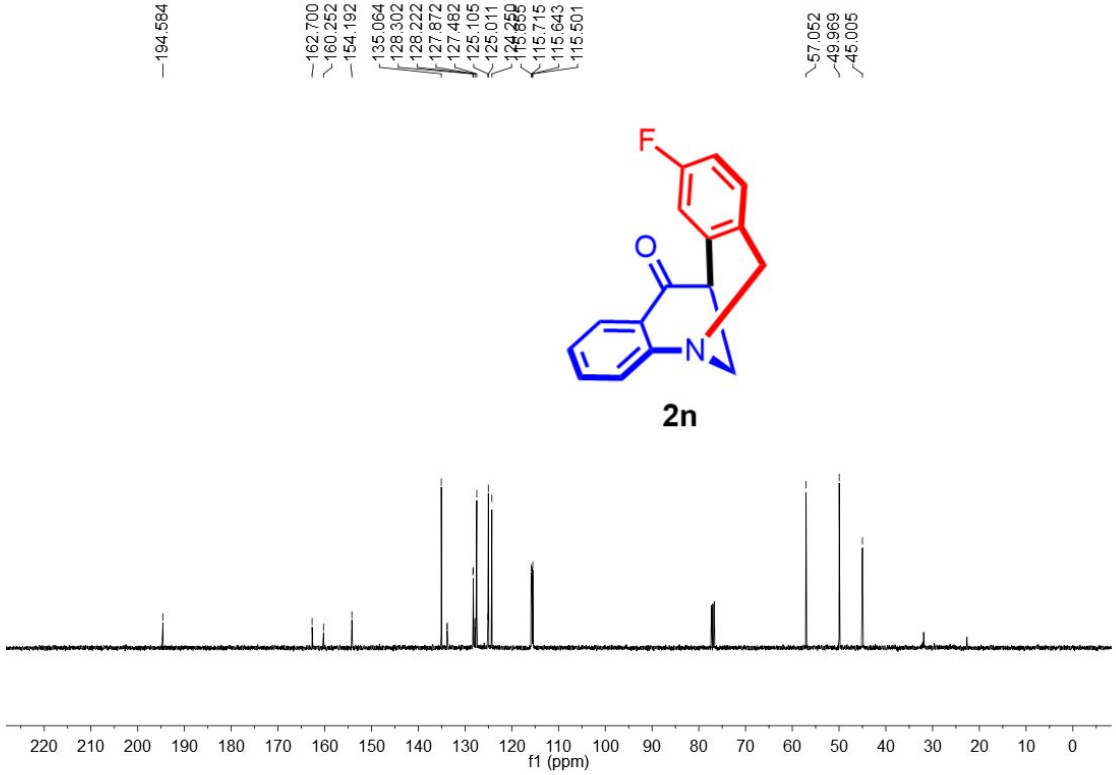

^{19}F NMR (377 MHz, $CDCl_3$) of compound **2n:**

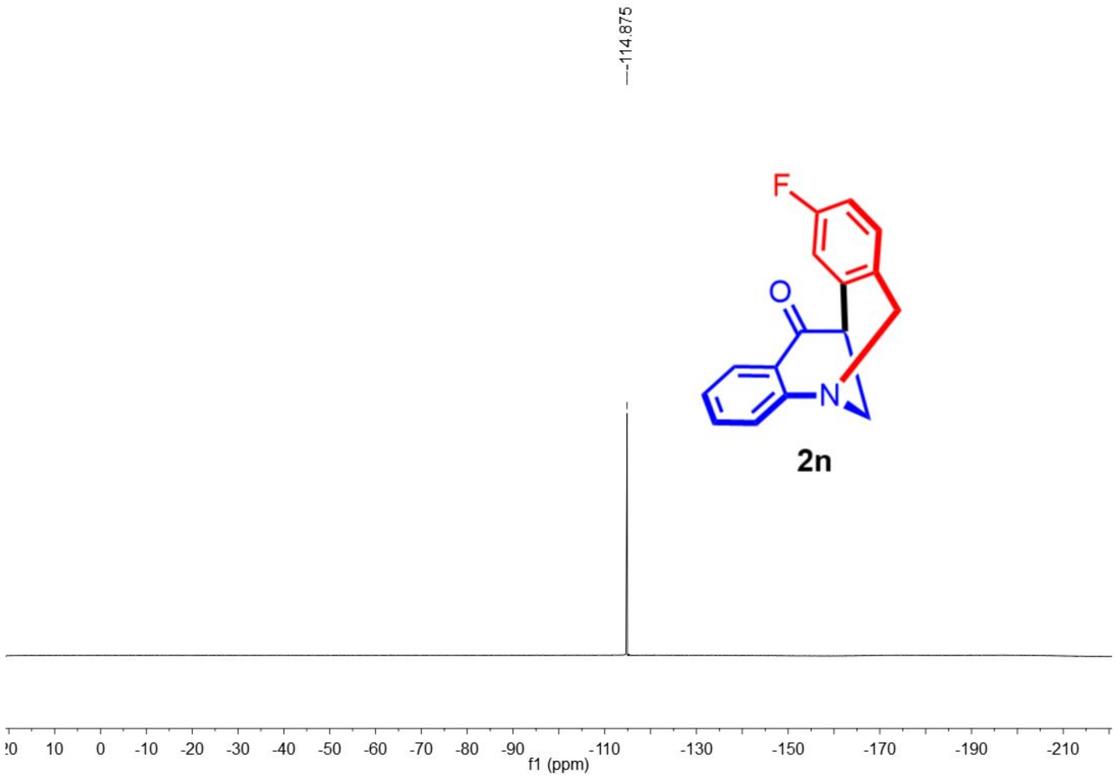

^{1}H NMR (400 MHz, $CDCl_3$) of compound **2o:**

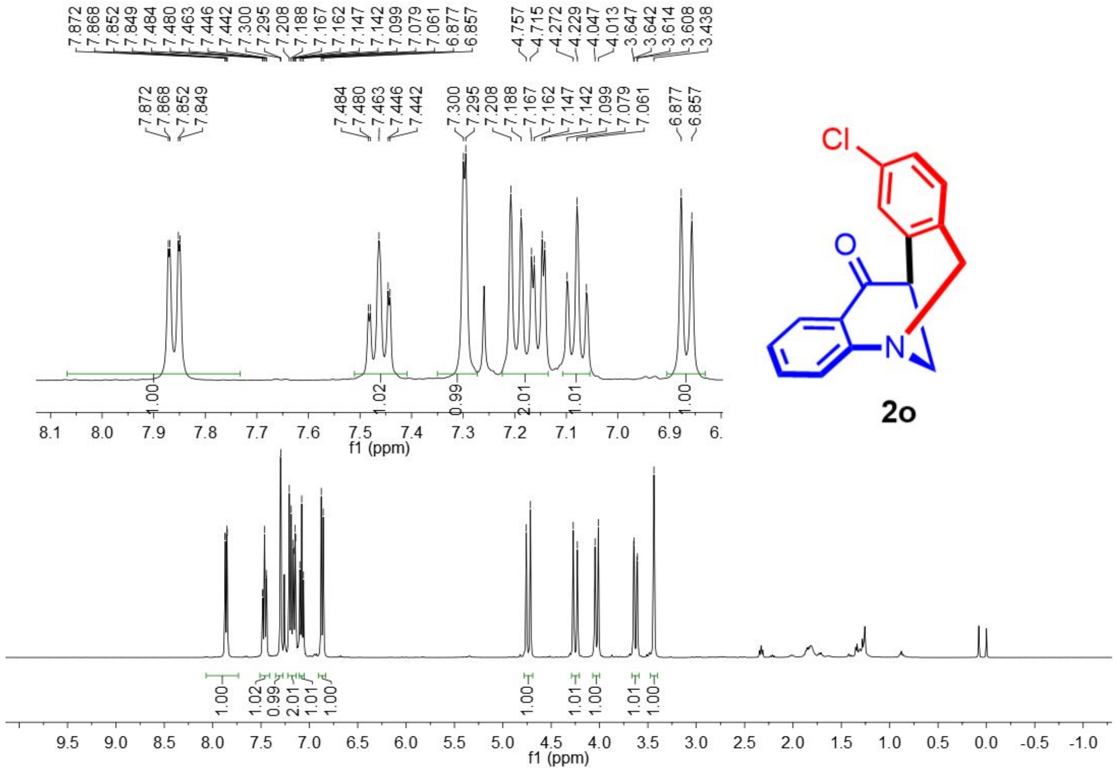

^{13}C NMR (100 MHz, $CDCl_3$) of compound **2o:**

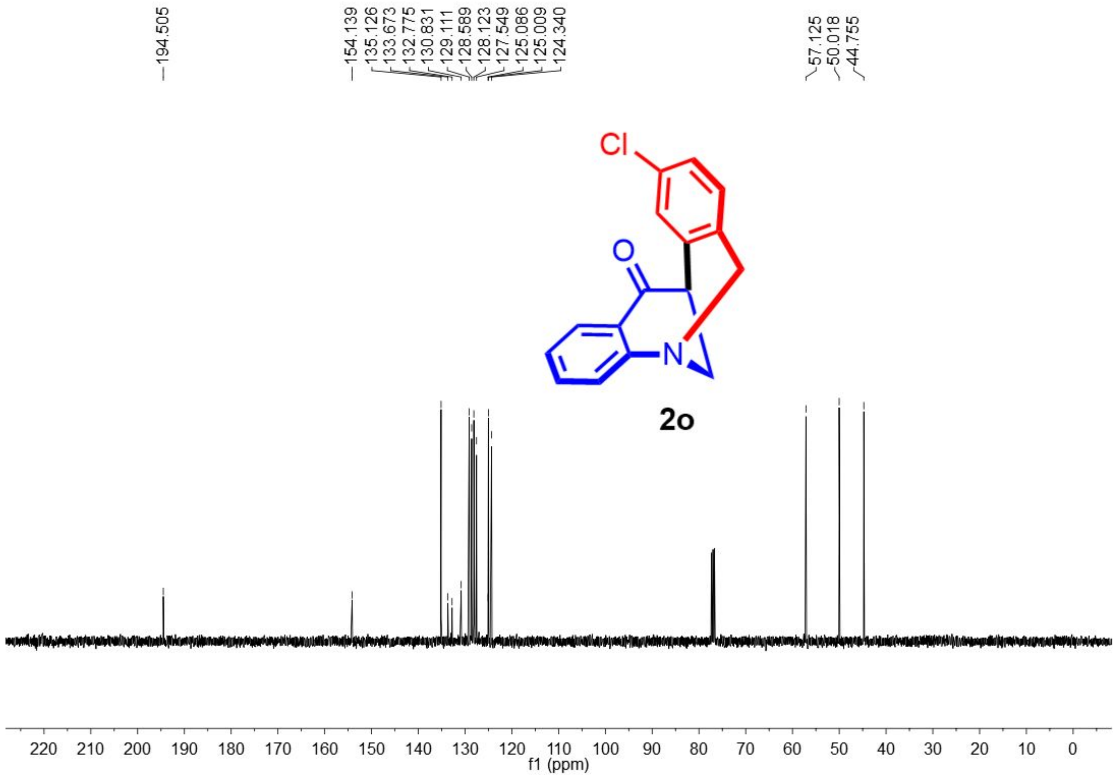

^{1}H NMR (400 MHz, $CDCl_3$) of compound **2p:**

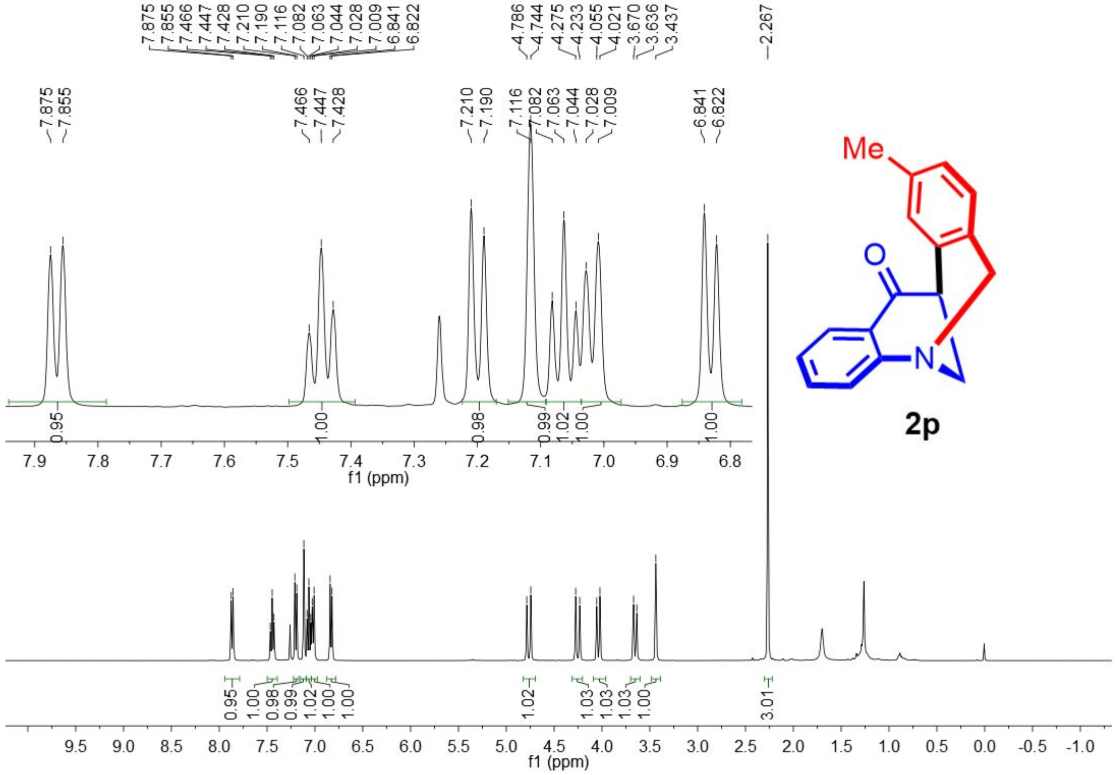

^{13}C NMR (100 MHz, $CDCl_3$) of compound **2p:**

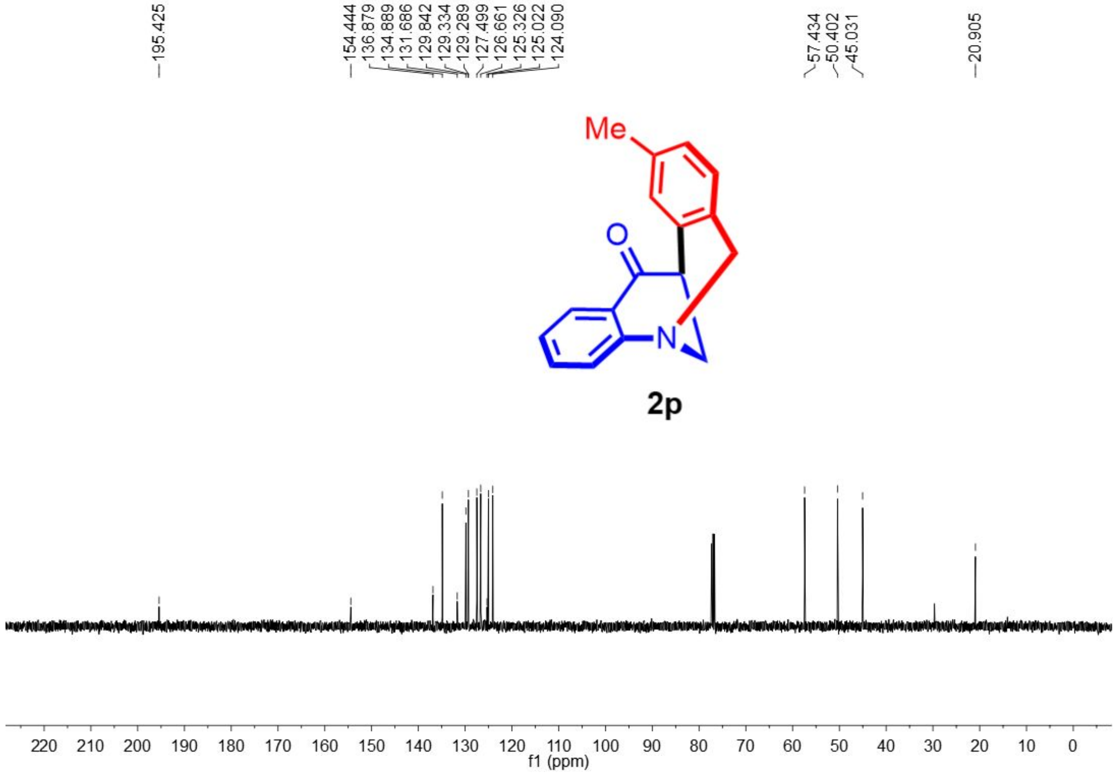

^{1}H NMR (400 MHz, $CDCl_3$) of compound **2q:**

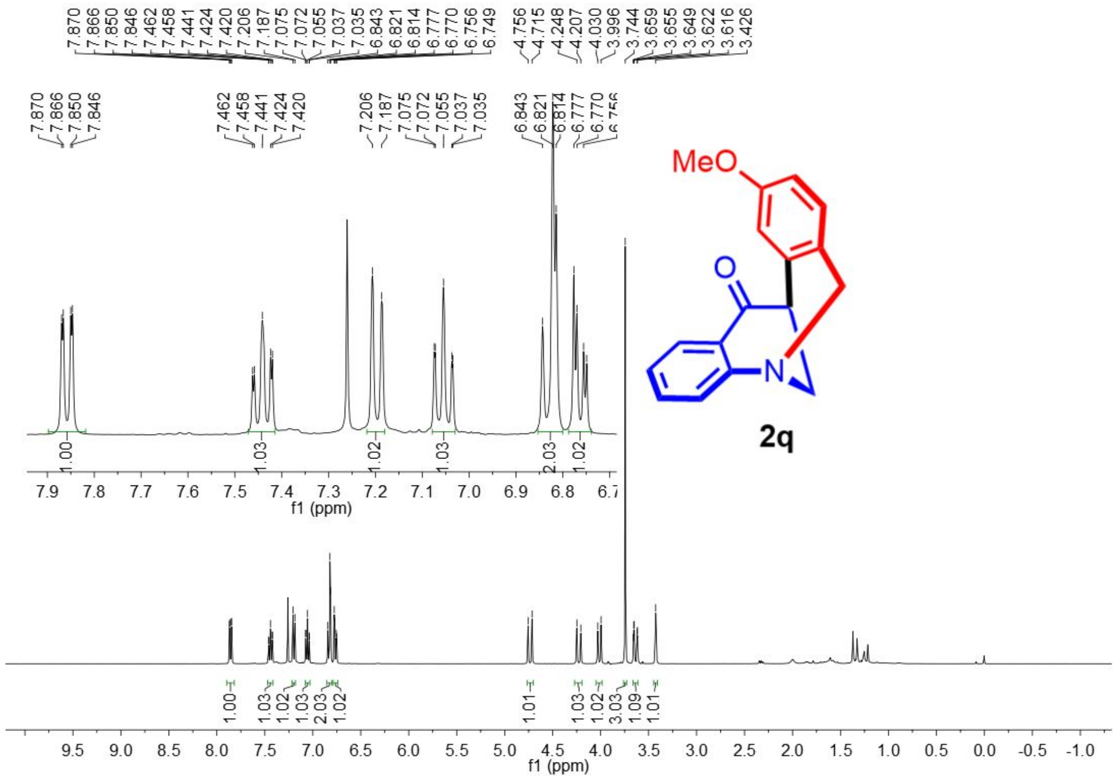

^{13}C NMR (100 MHz, $CDCl_3$) of compound **2q:**

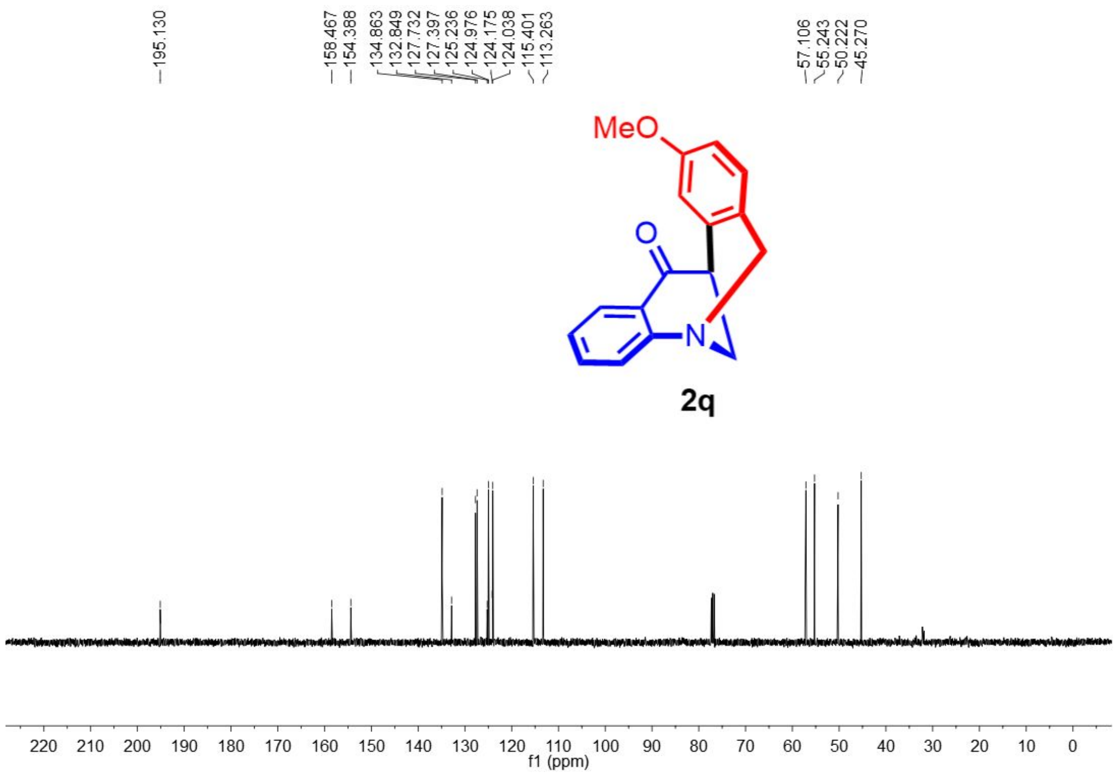

^{1}H NMR (400 MHz, $CDCl_3$) of compound **2r:**

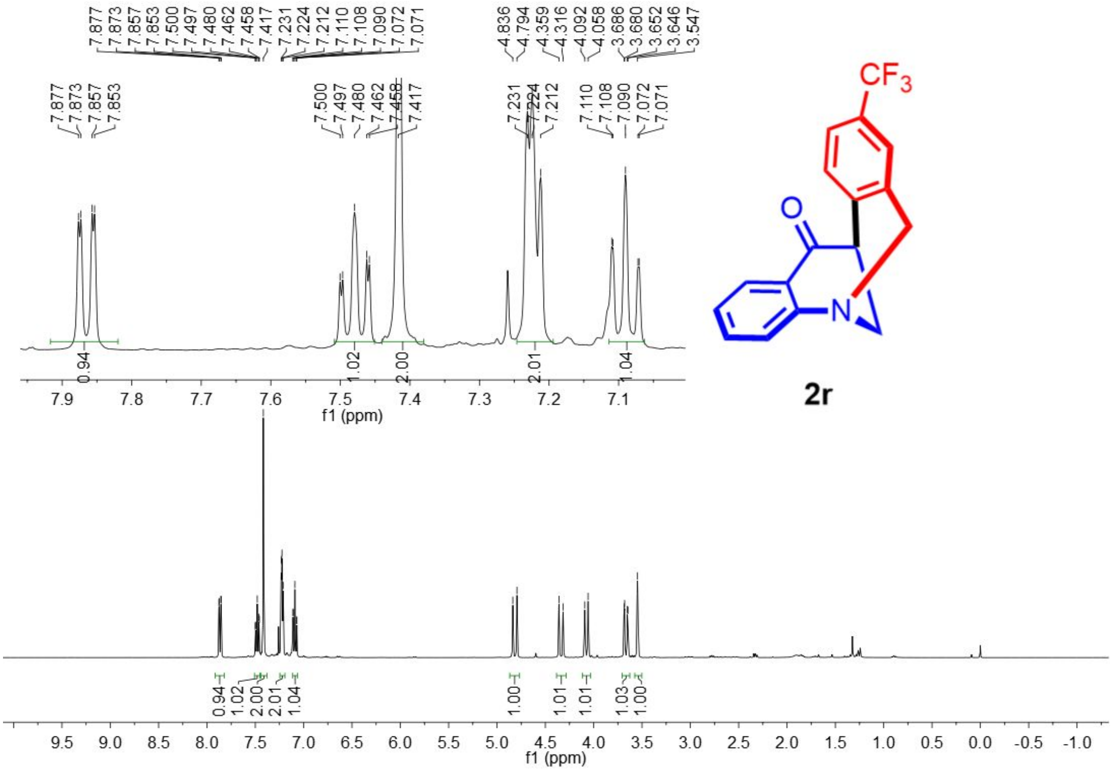

^{13}C NMR (100 MHz, $CDCl_3$) of compound **2r:**

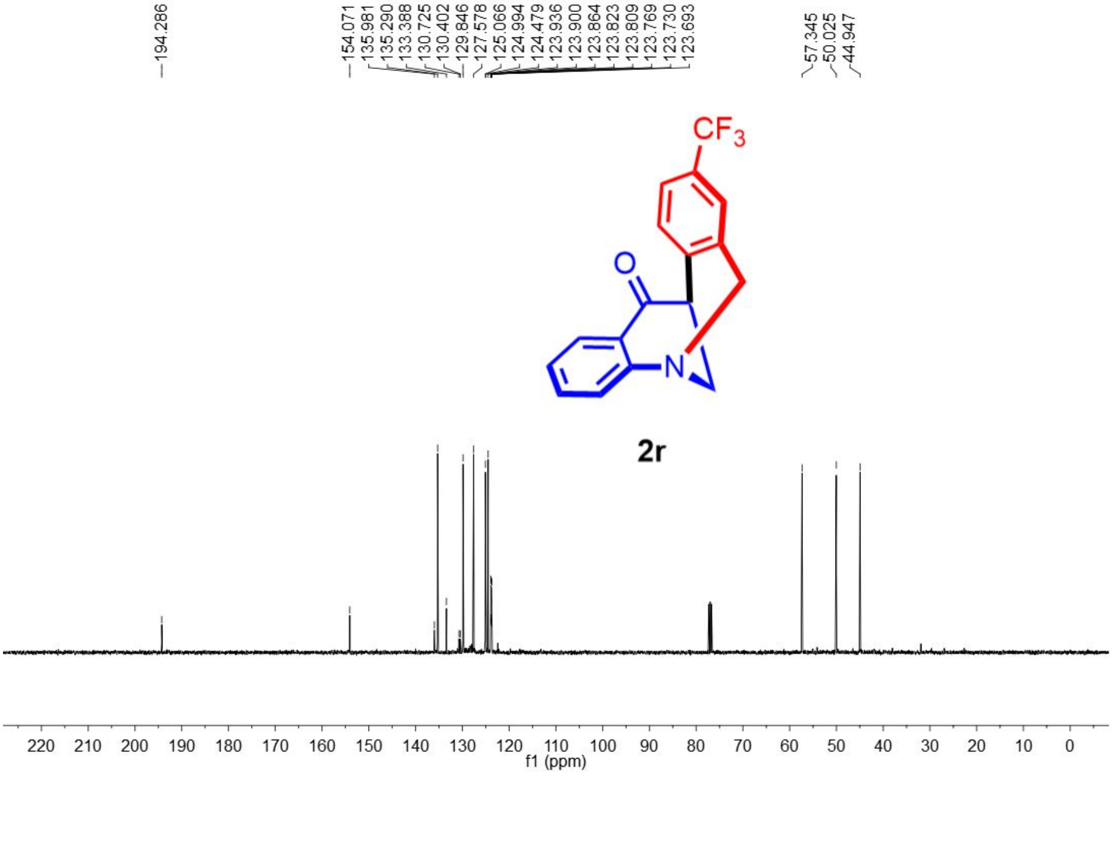

^{19}F NMR (377 MHz, $CDCl_3$) of compound **2r:**

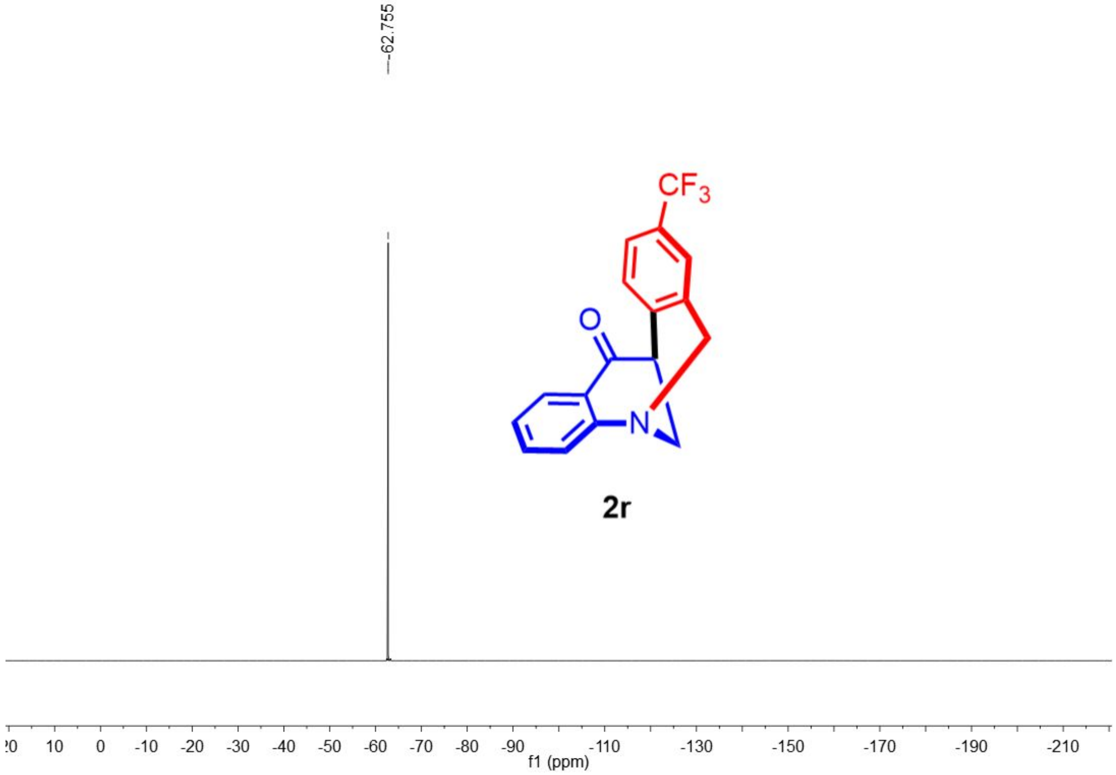

^{1}H NMR (400 MHz, $CDCl_3$) of compound **2s:**

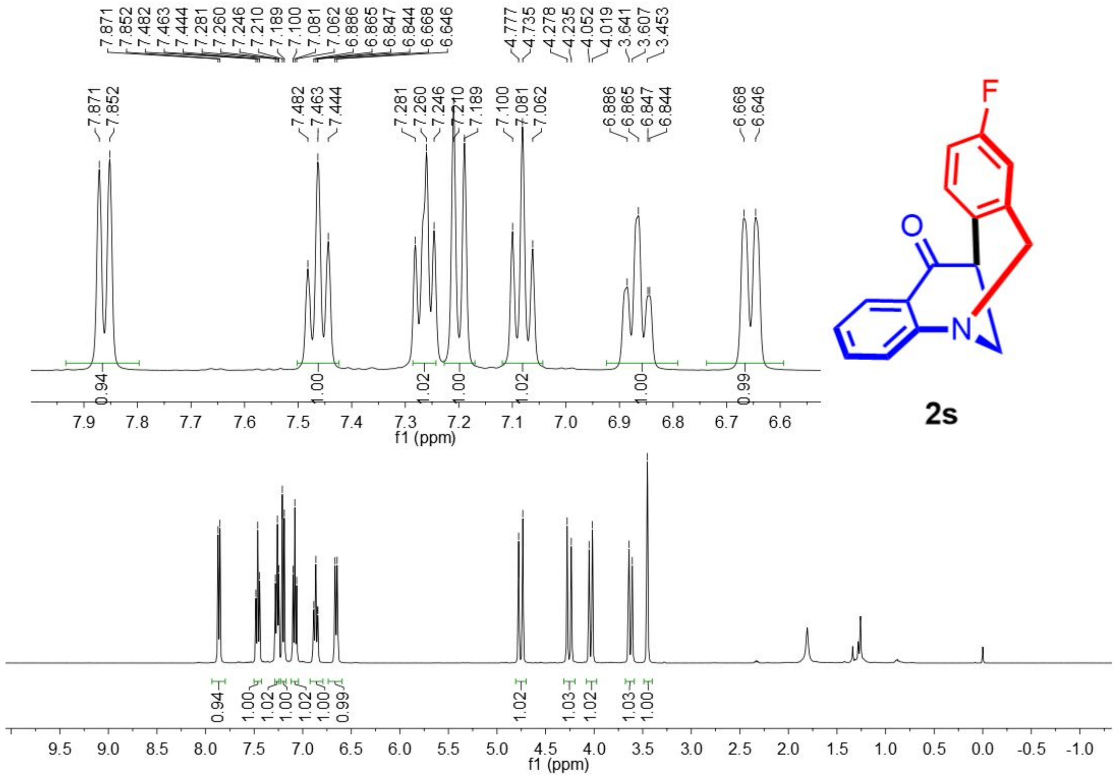

^{13}C NMR (100 MHz, $CDCl_3$) of compound **2s:**

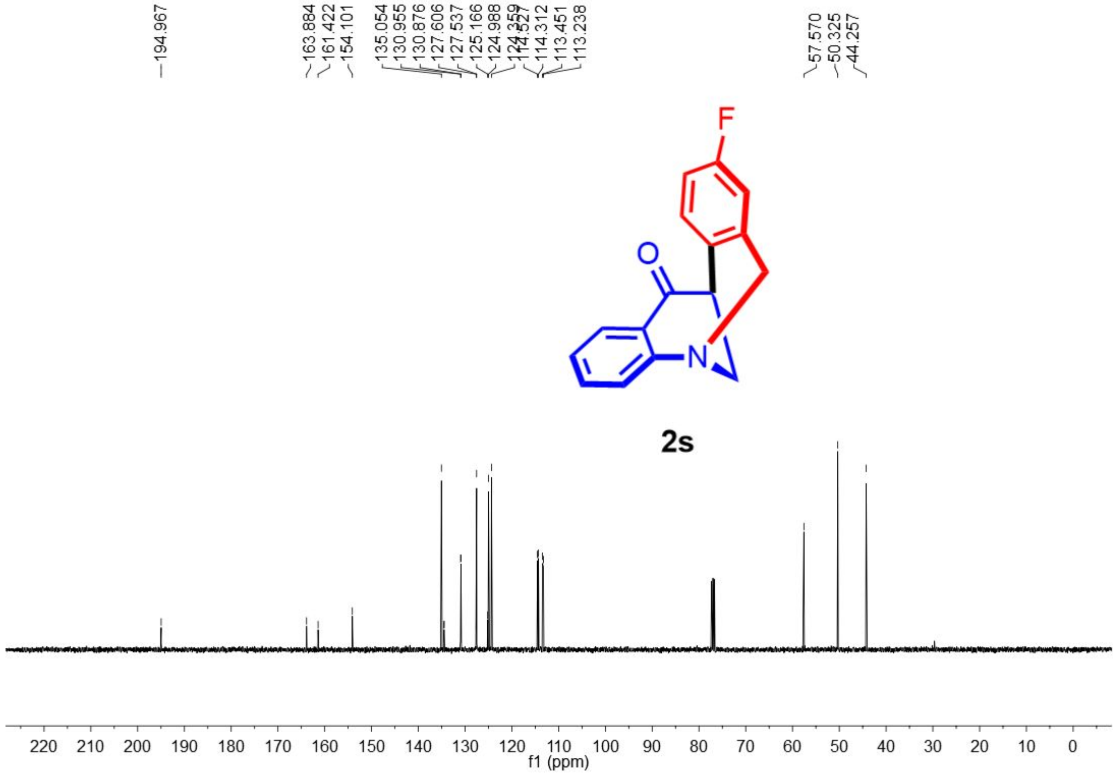

^{19}F NMR (377 MHz, $CDCl_3$) of compound **2s:**

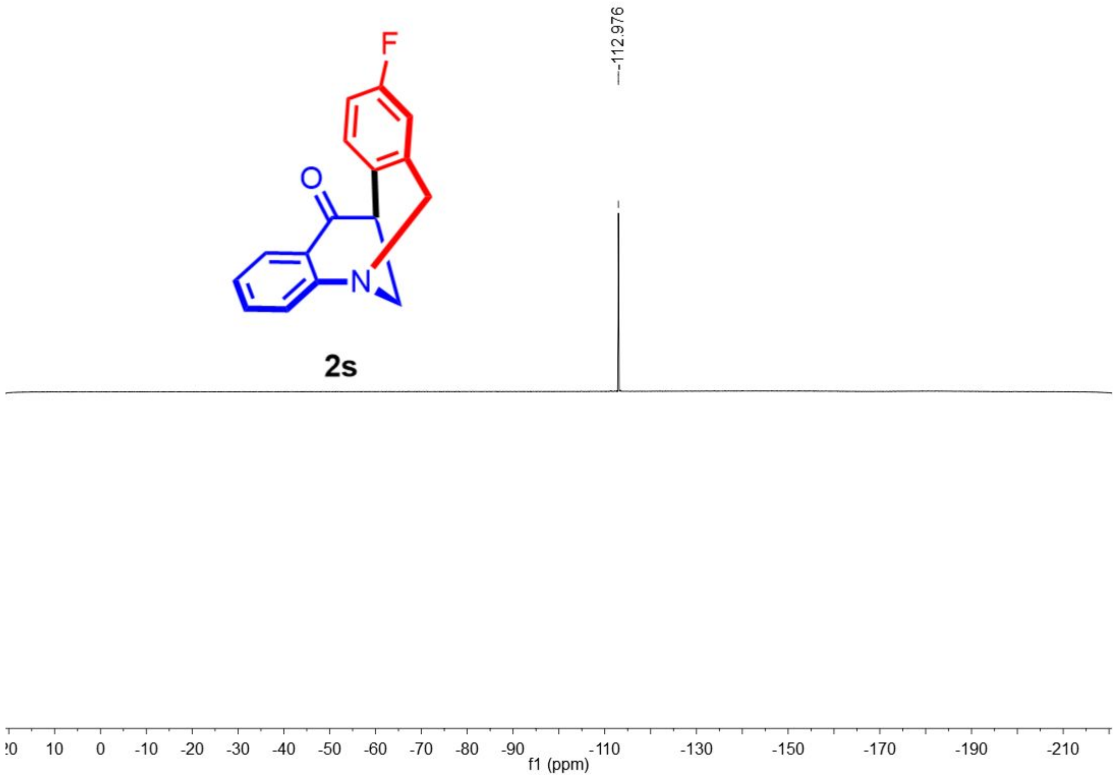

^{1}H NMR (400 MHz, $CDCl_3$) of compound **2t:**

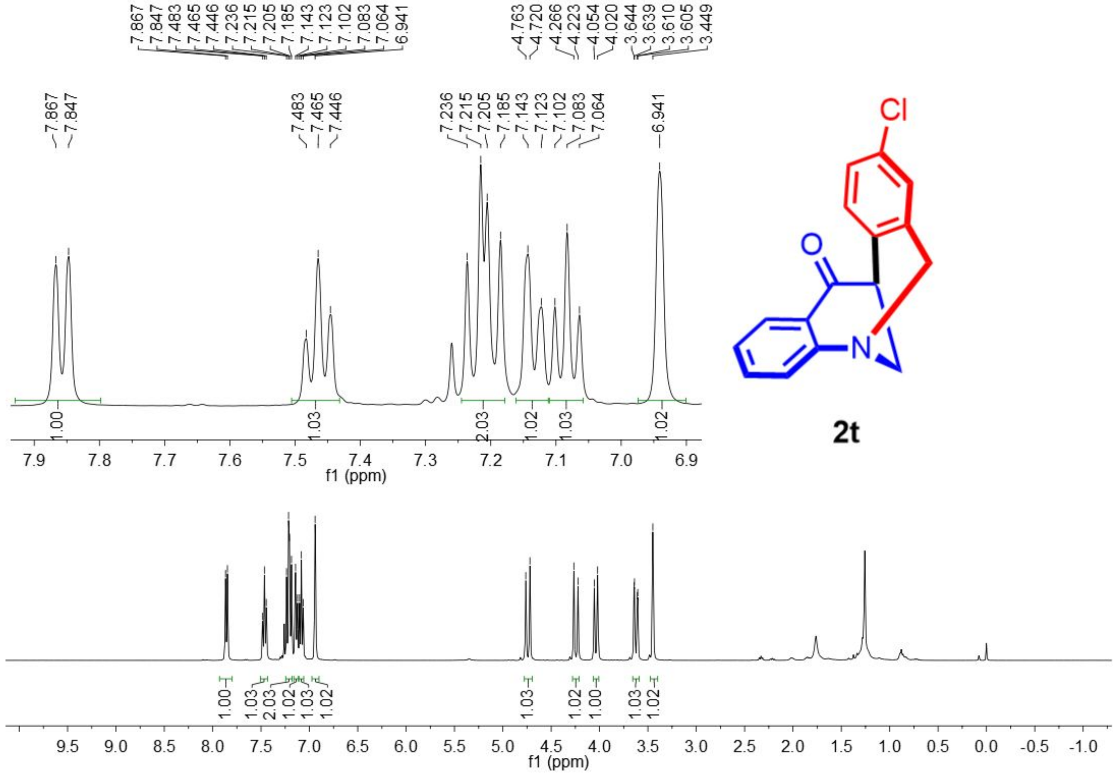

^{13}C NMR (100 MHz, $CDCl_3$) of compound **2t:**

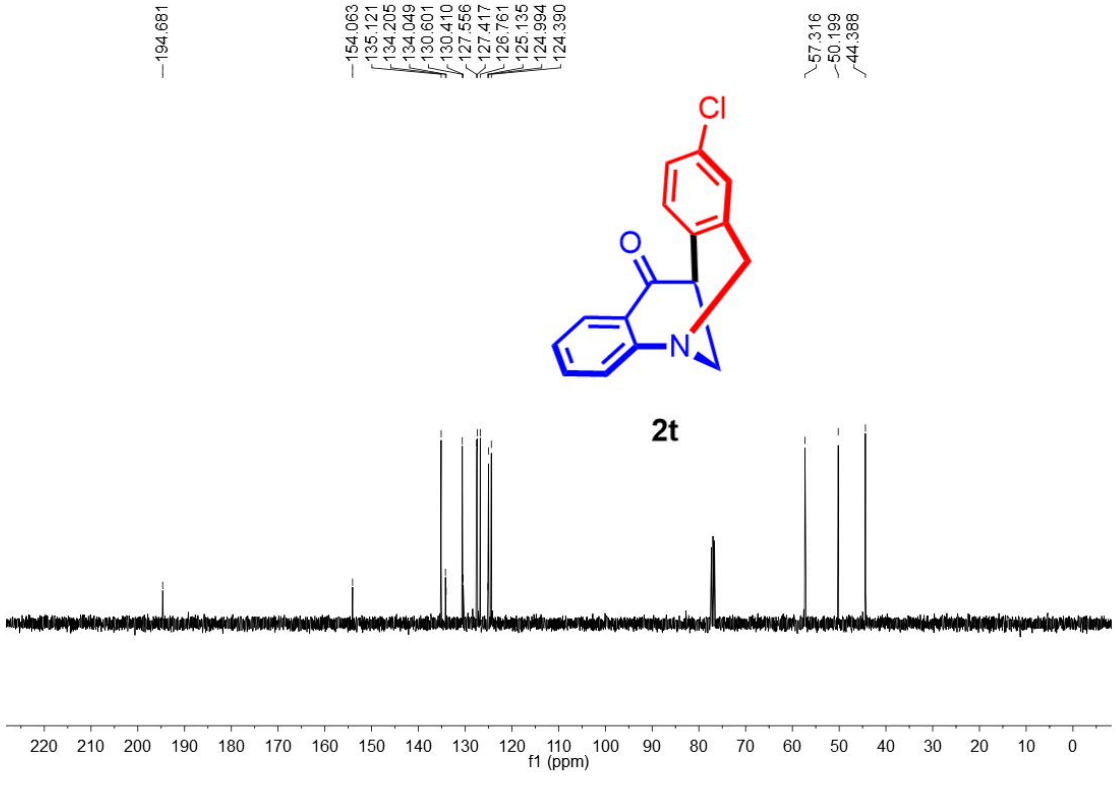

^{1}H NMR (400 MHz, $CDCl_3$) of compound **2u:**

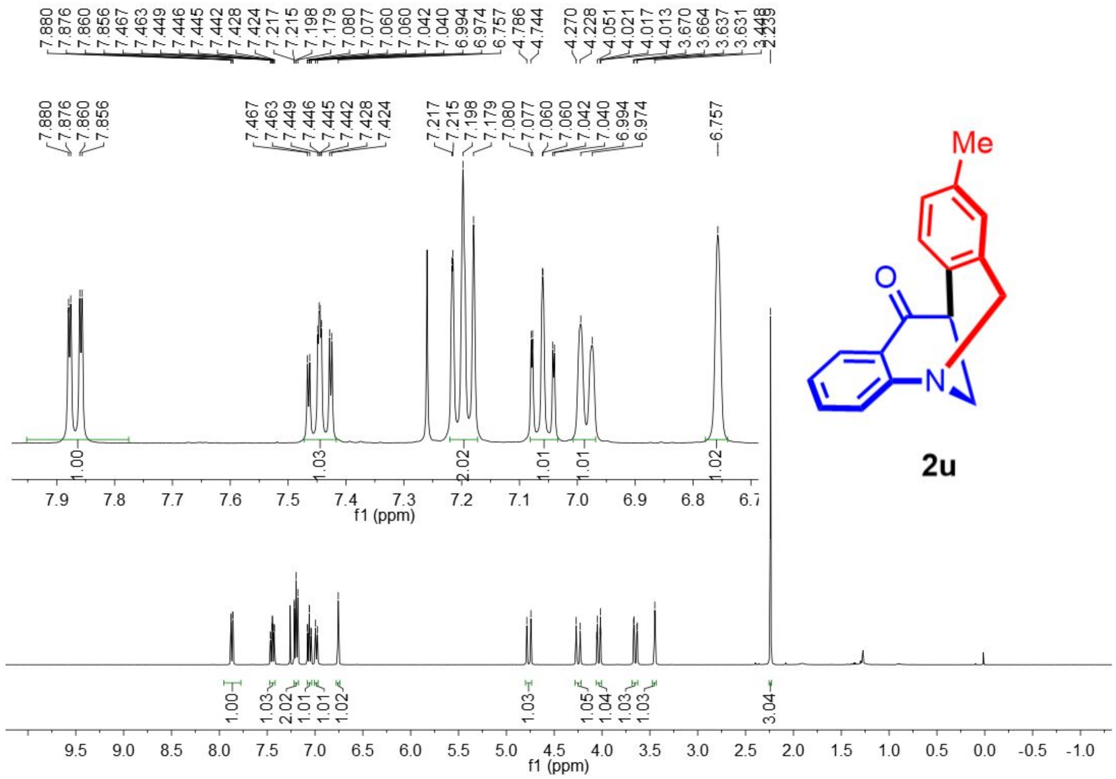

^{13}C NMR (100 MHz, $CDCl_3$) of compound **2u:**

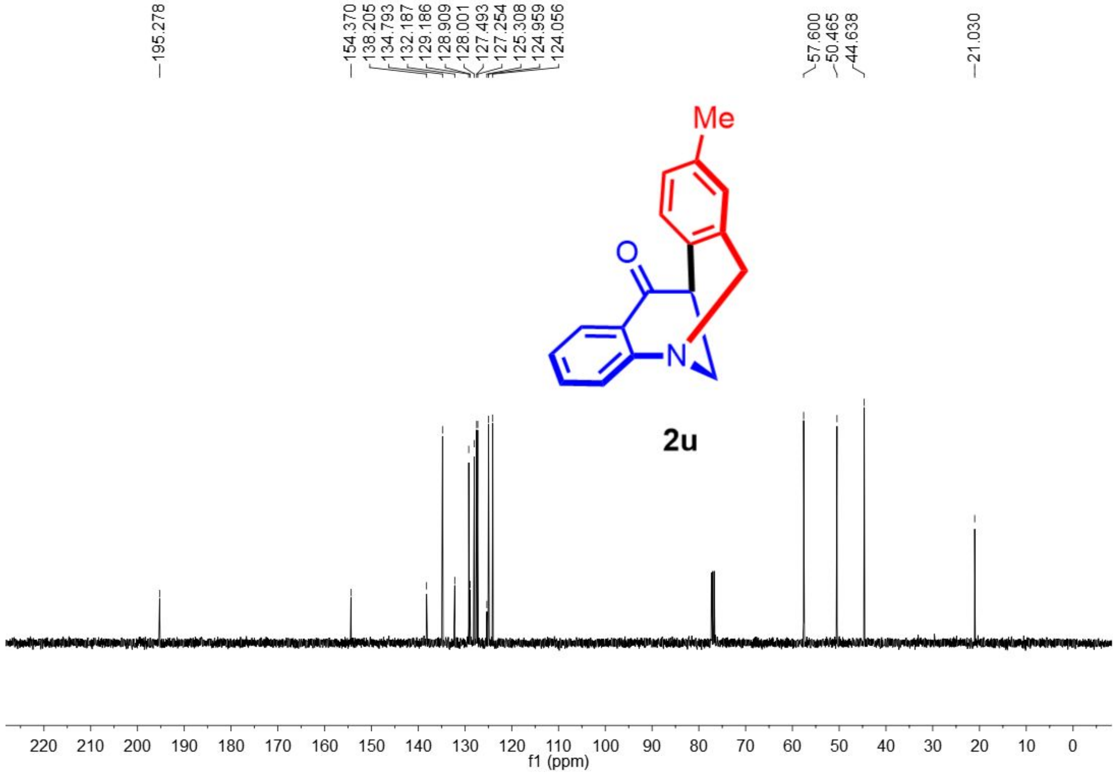

^{1}H NMR (400 MHz, CDCl$_3$) of compound **2v:**

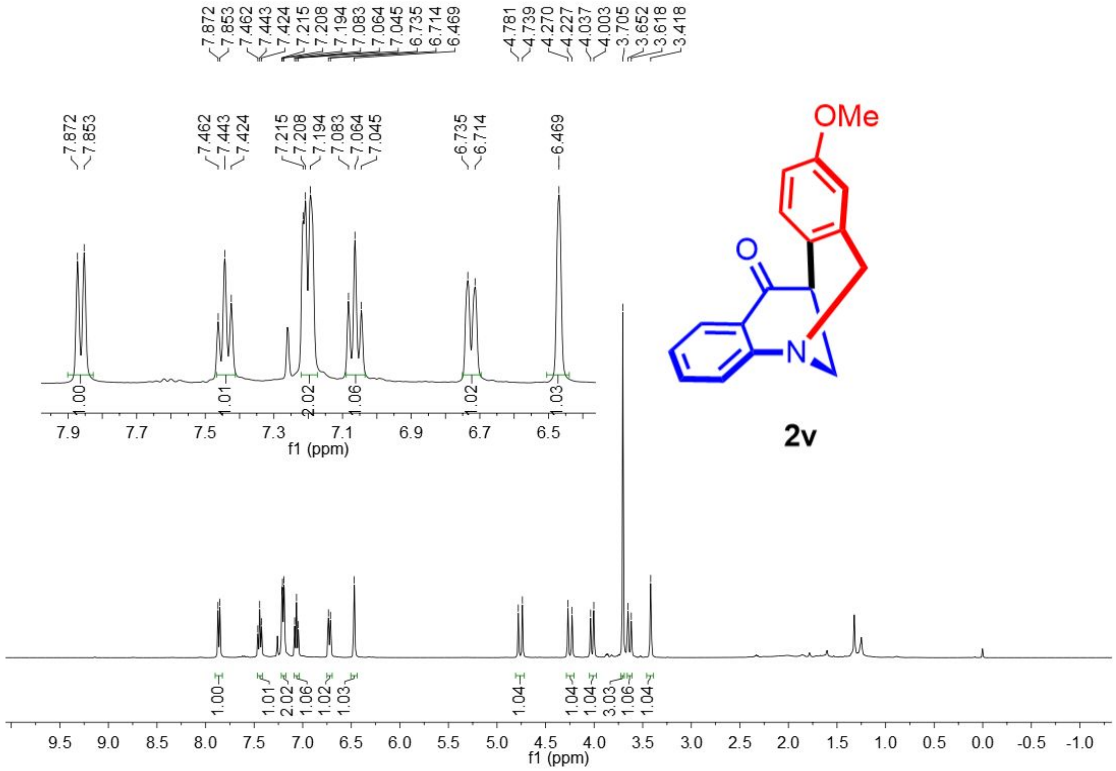

^{13}C NMR (100 MHz, CDCl$_3$) of compound **2v:**

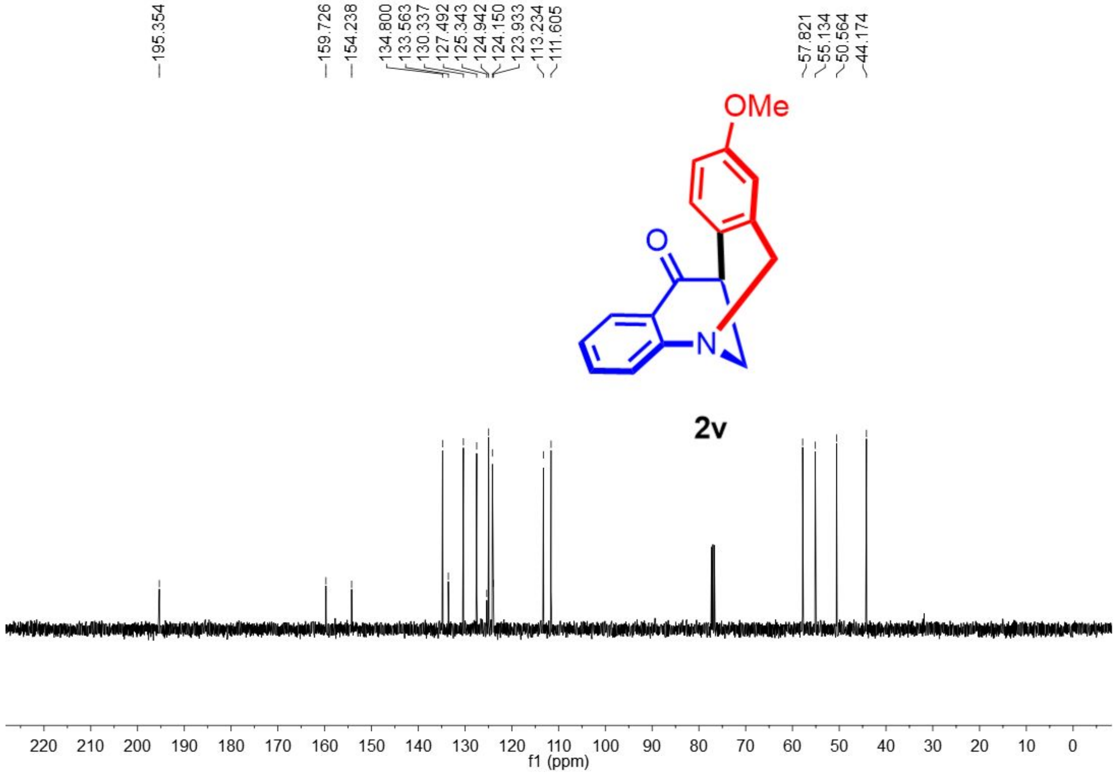

^{1}H NMR (400 MHz, $CDCl_3$) of compound **2w:**

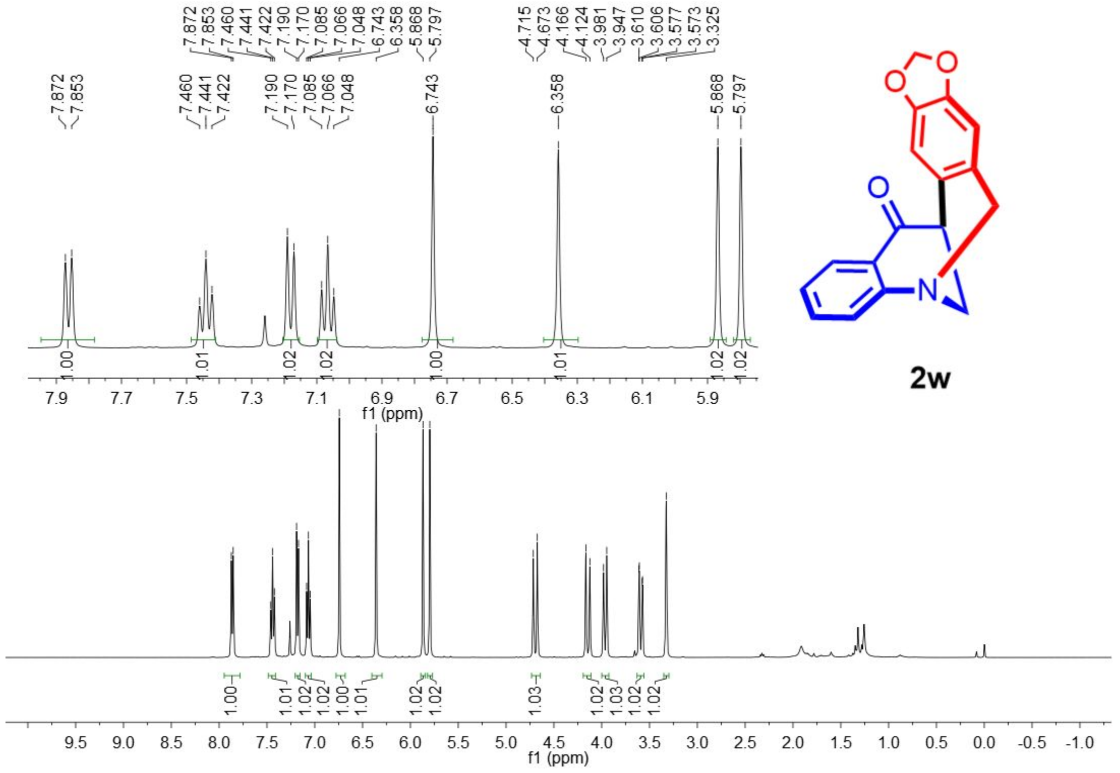

^{13}C NMR (100 MHz, $CDCl_3$) of compound **2w:**

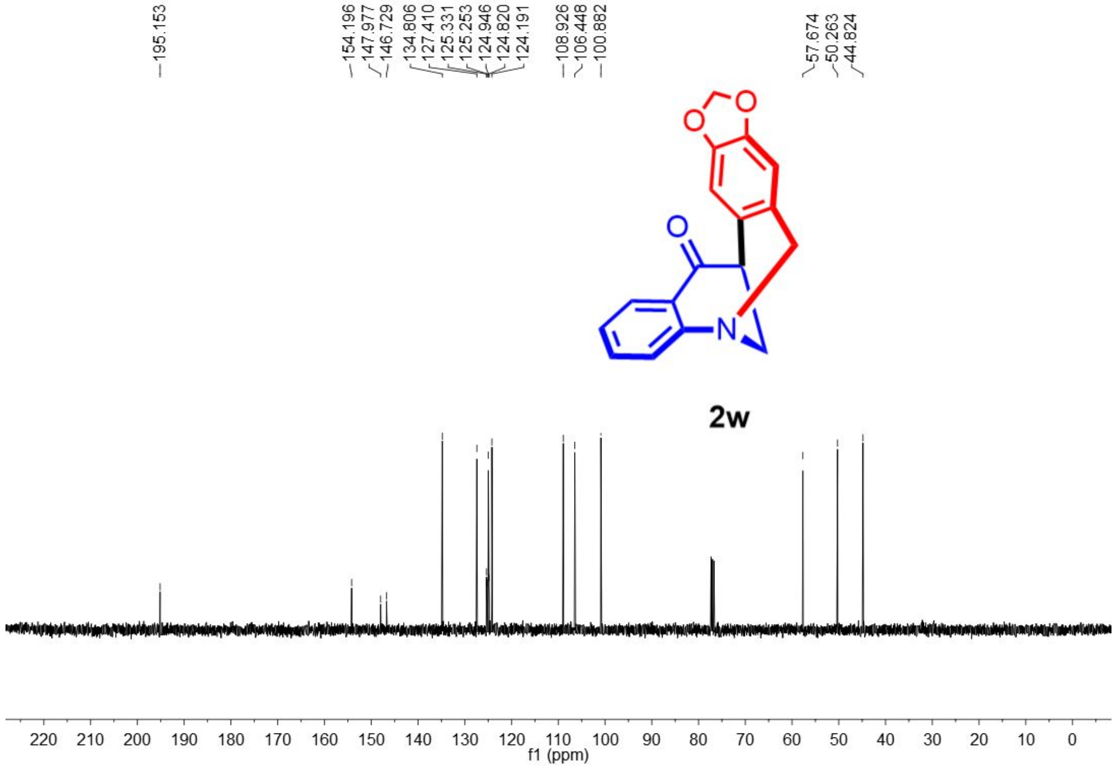

^{1}H NMR (400 MHz, $CDCl_3$) of compound **2x:**

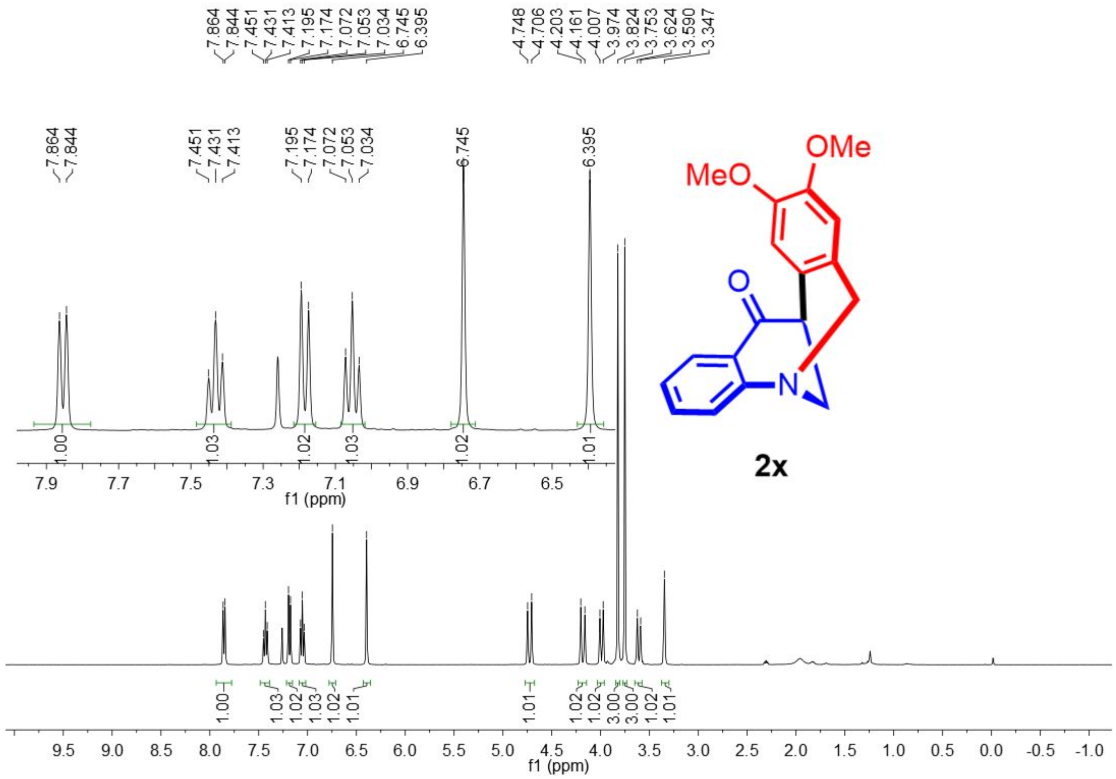

^{13}C NMR (100 MHz, $CDCl_3$) of compound **2x:**

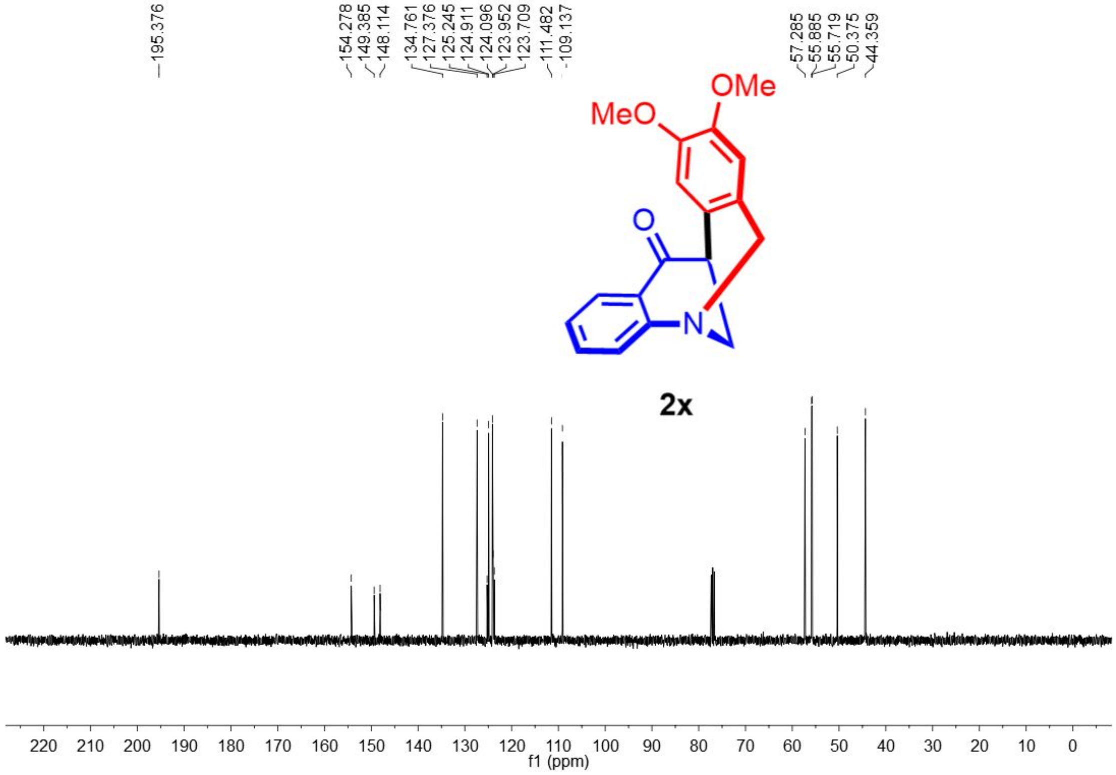

^{1}H NMR (400 MHz, $CDCl_3$) of compound **2y:**

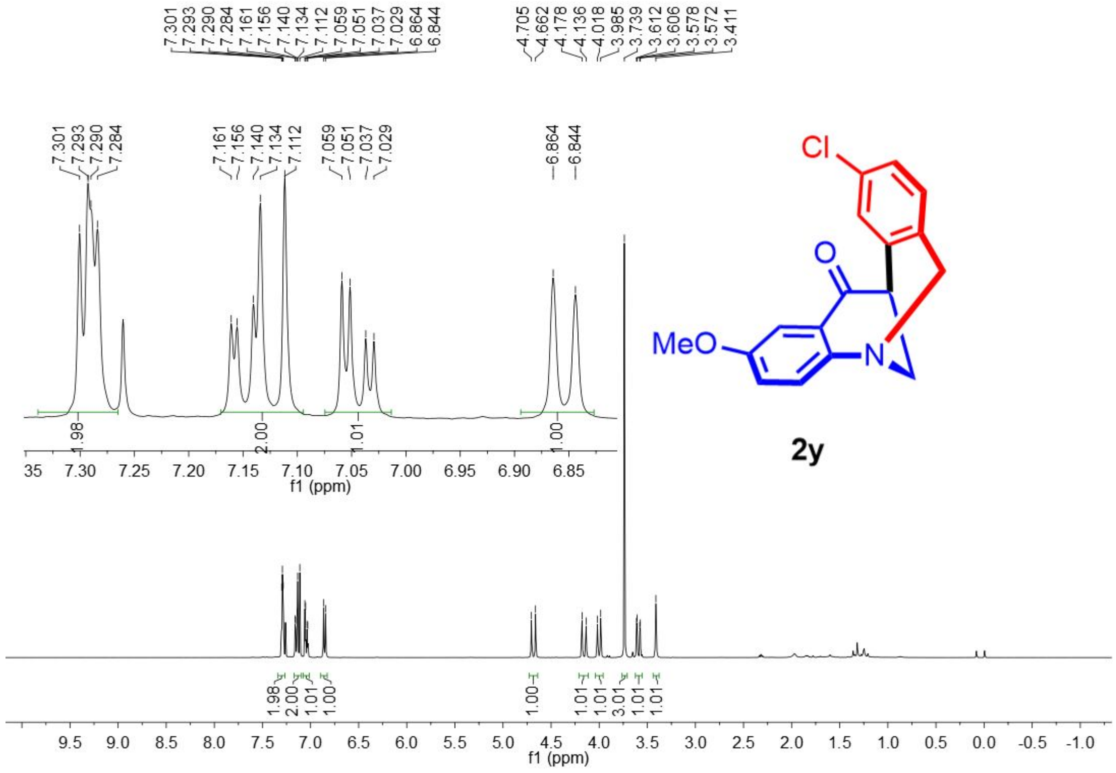

^{13}C NMR (100 MHz, $CDCl_3$) of compound **2y:**

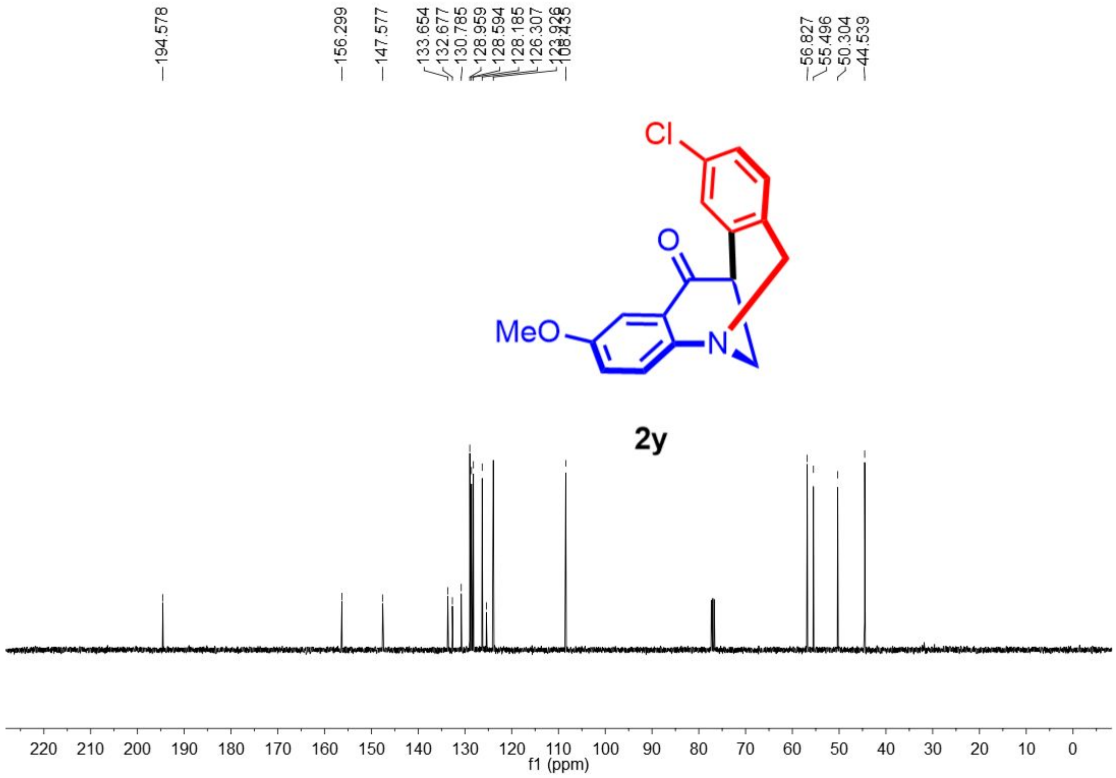

^{1}H NMR (400 MHz, $CDCl_3$) of compound **2z:**

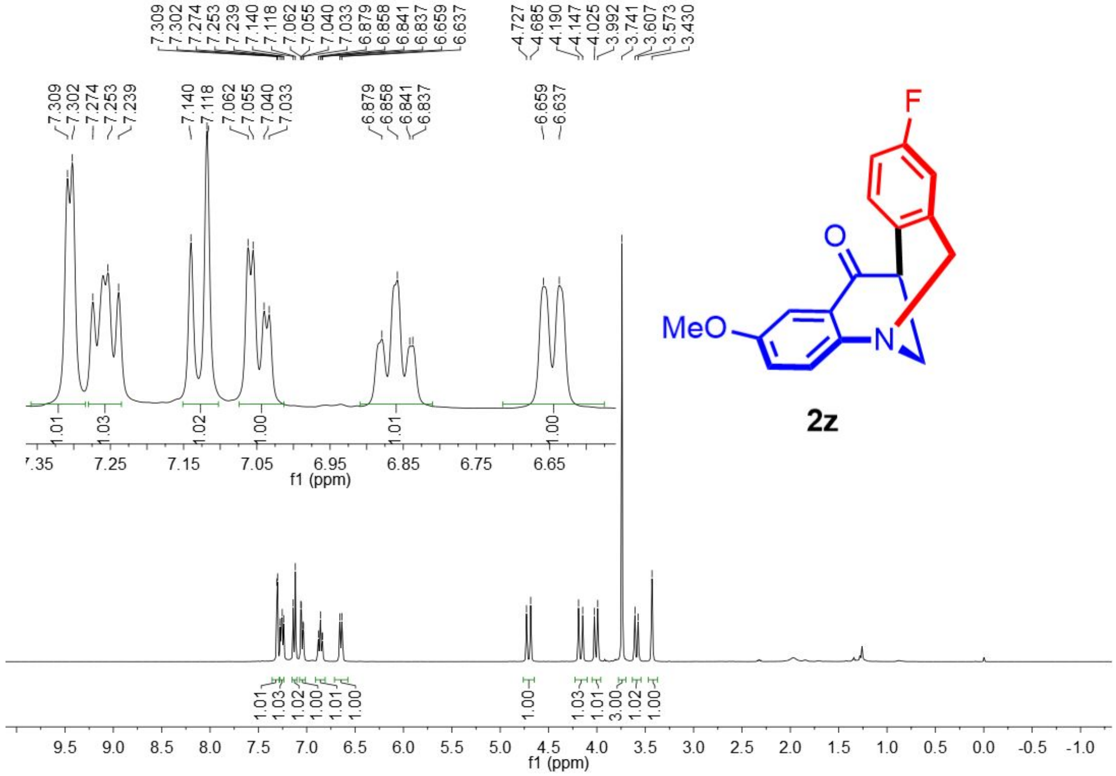

^{13}C NMR (100 MHz, $CDCl_3$) of compound **2z:**

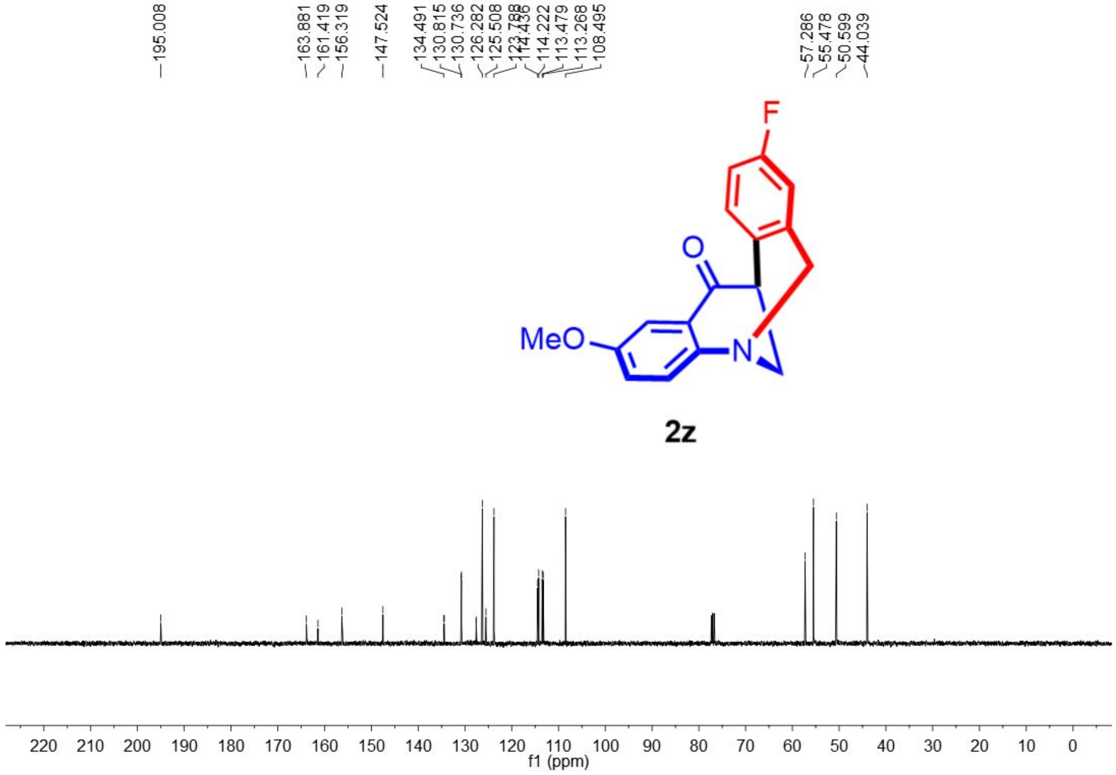

^{19}F NMR (377 MHz, $CDCl_3$) of compound **2z:**

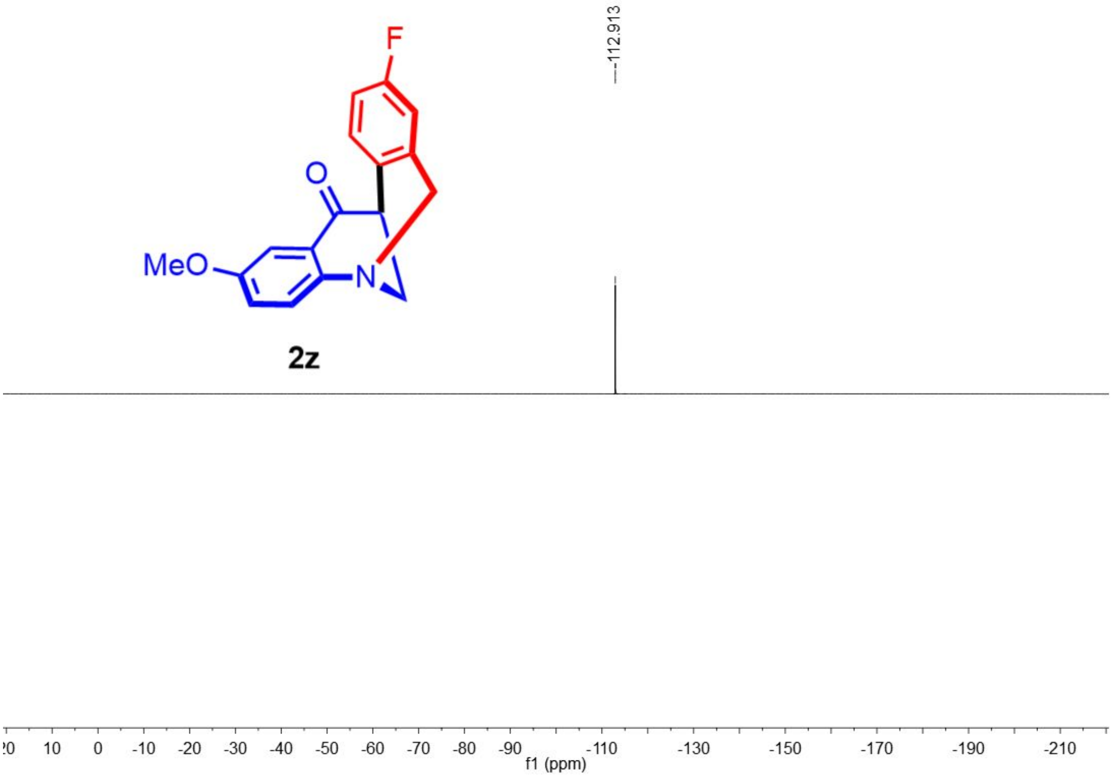

^{1}H NMR (400 MHz, $CDCl_3$) of compound **2aa:**

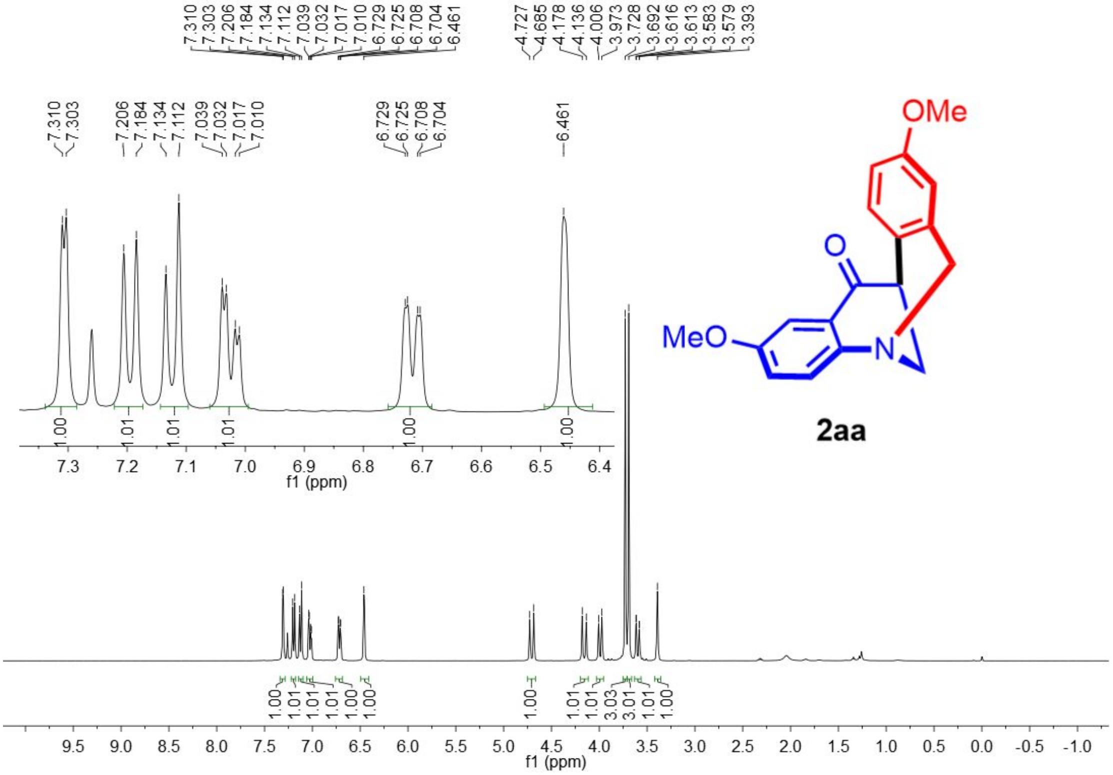

^{13}C NMR (100 MHz, $CDCl_3$) of compound **2aa:**

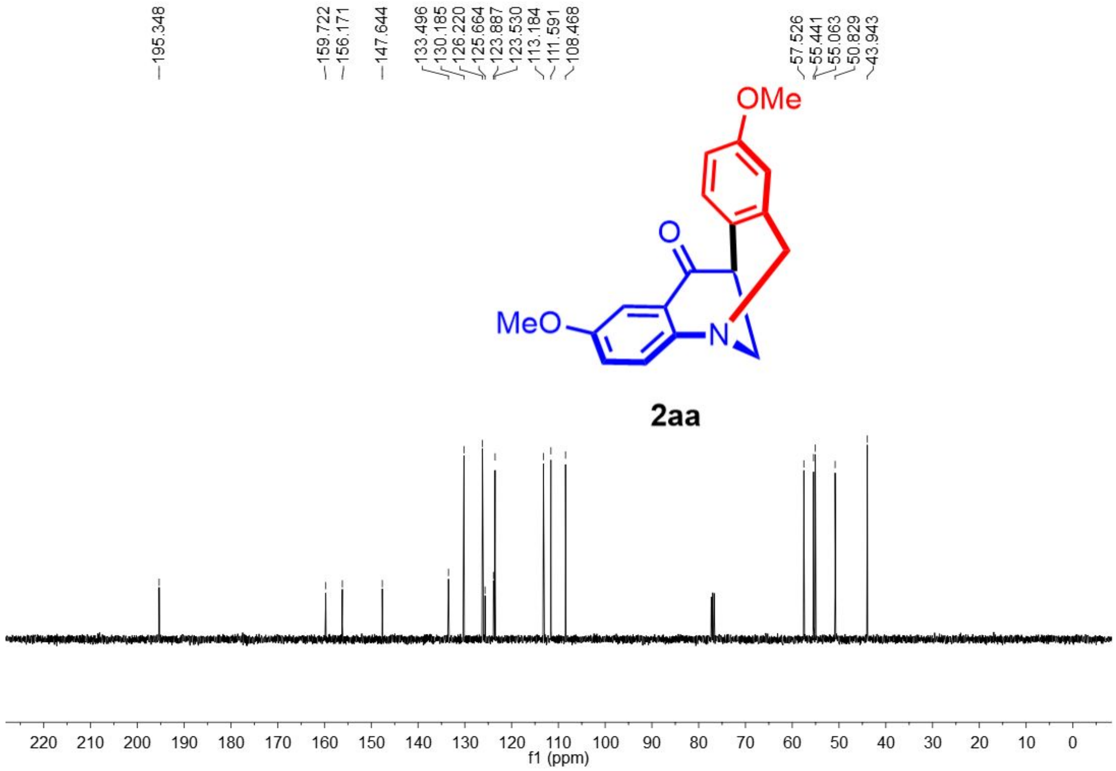

^{1}H NMR (400 MHz, $CDCl_3$) of compound **2ab:**

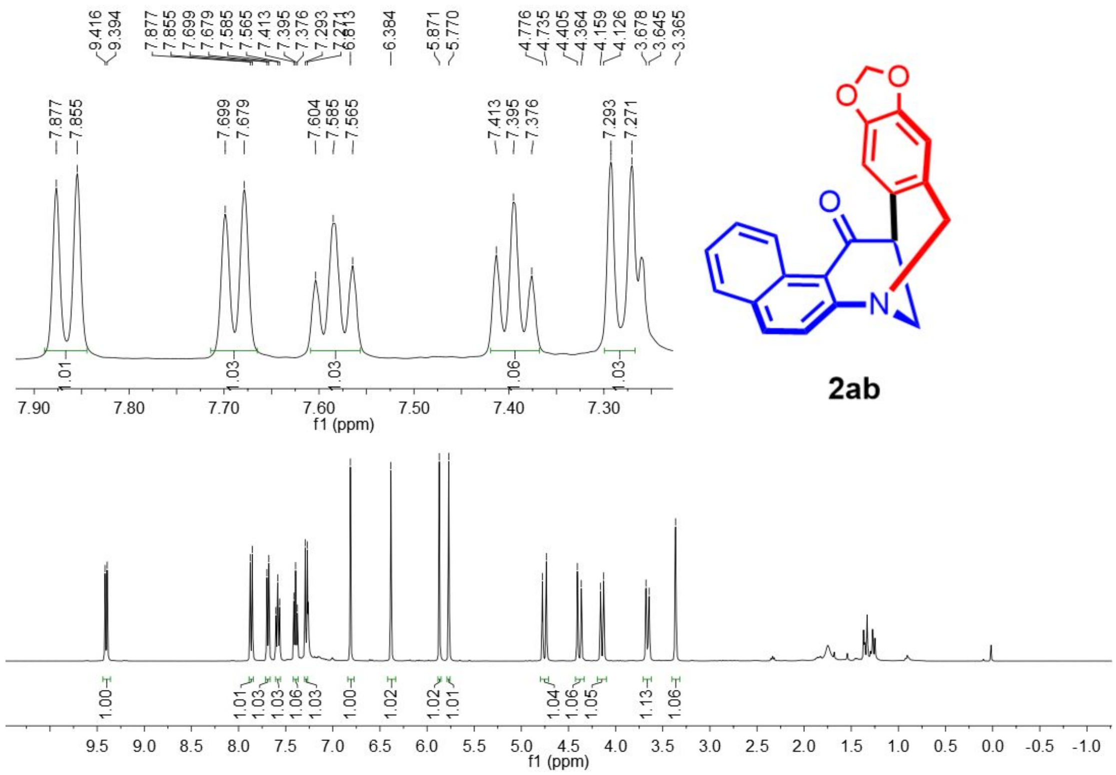

^{13}C NMR (100 MHz, $CDCl_3$) of compound **2ab:**

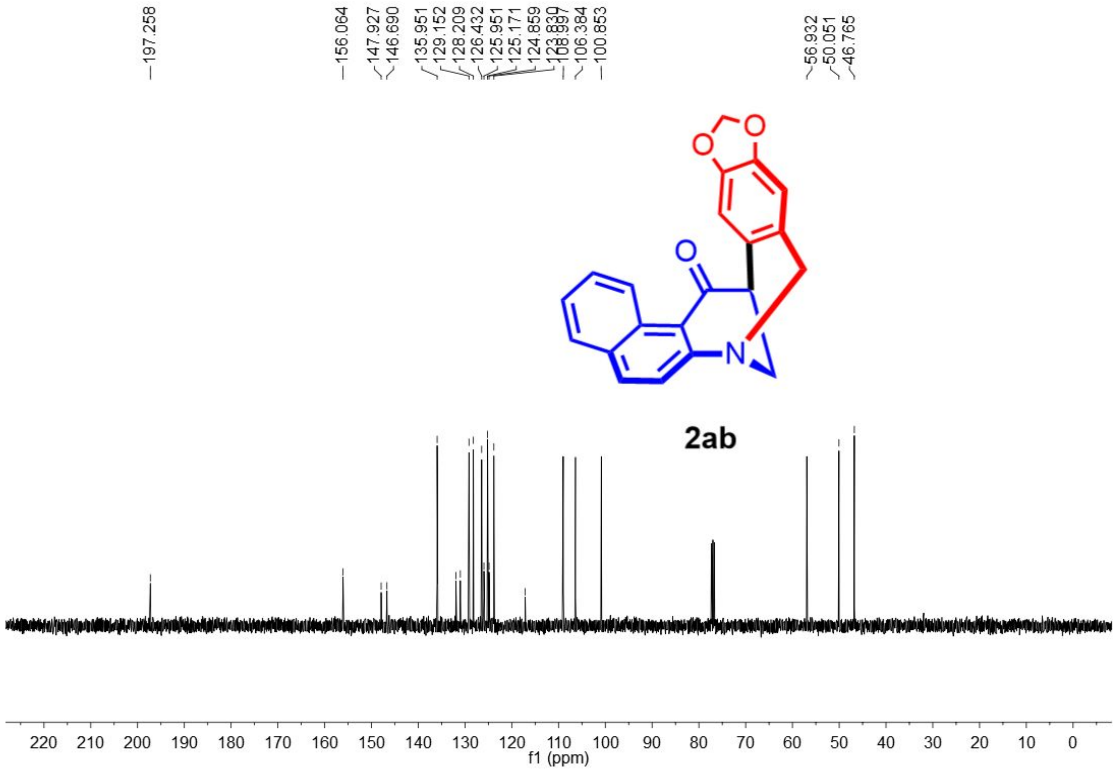

^{1}H NMR (400 MHz, $CDCl_3$) of compound **2ac:**

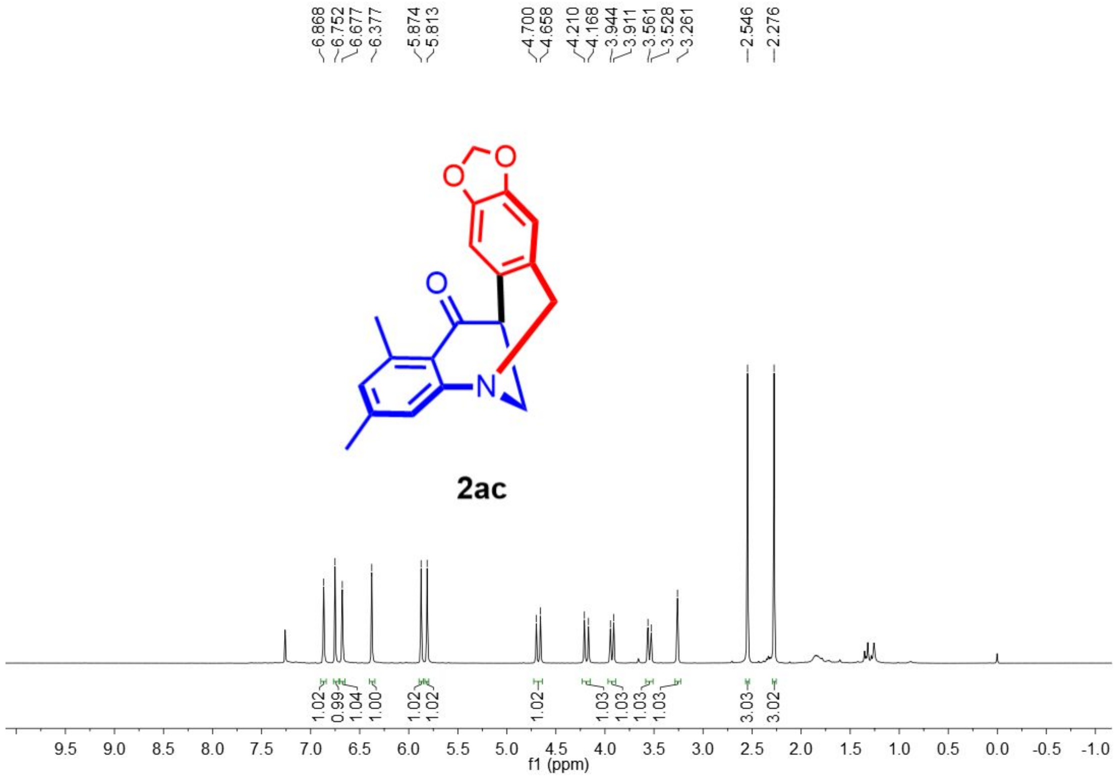

^{13}C NMR (100 MHz, $CDCl_3$) of compound **2ac:**

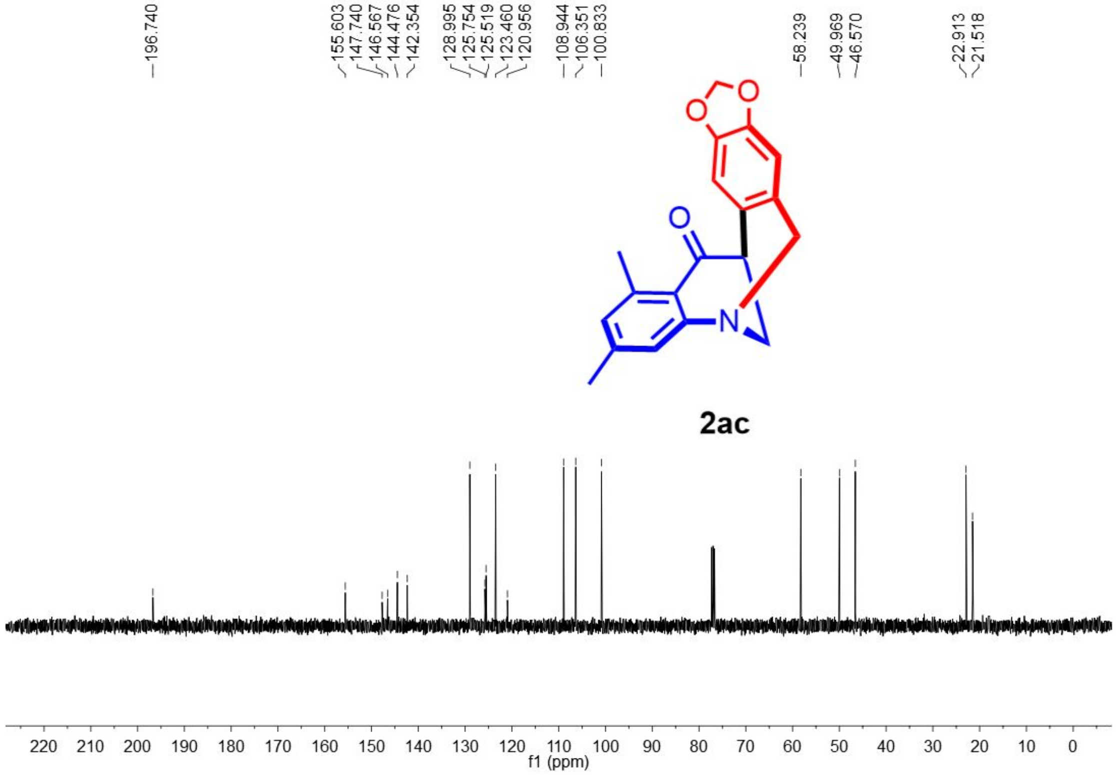

^{1}H NMR (400 MHz, $CDCl_3$) of compound **3:**

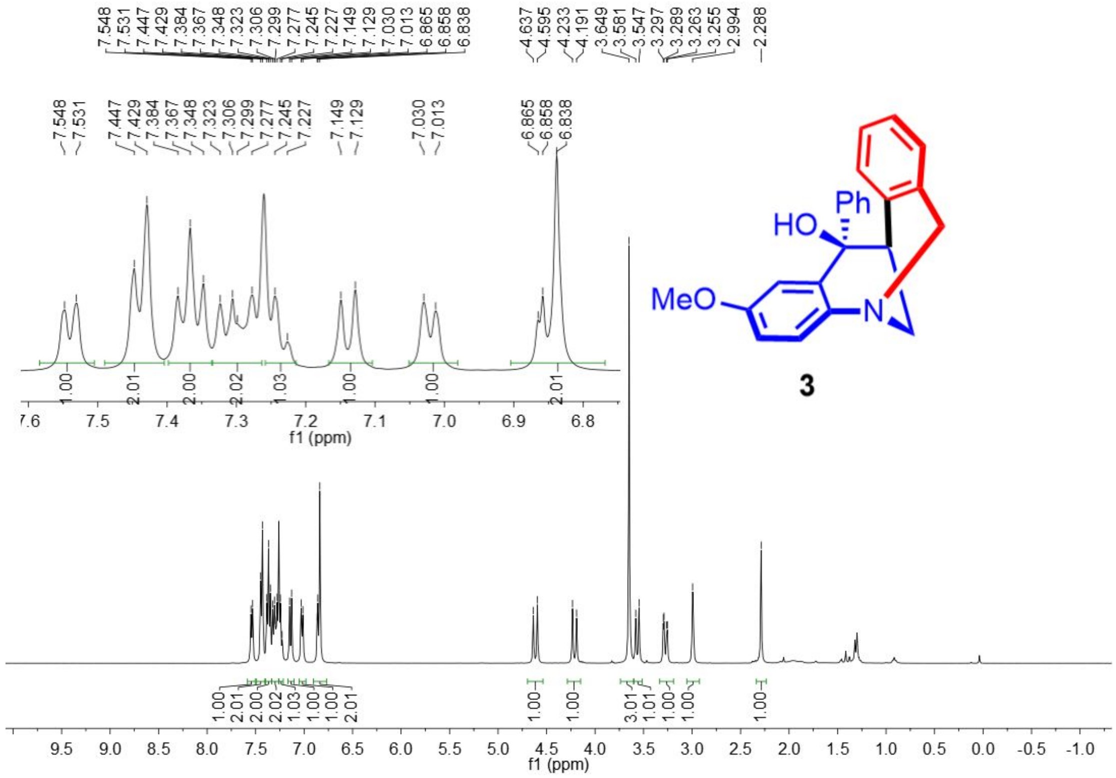

^{13}C NMR (100 MHz, $CDCl_3$) of compound **3:**

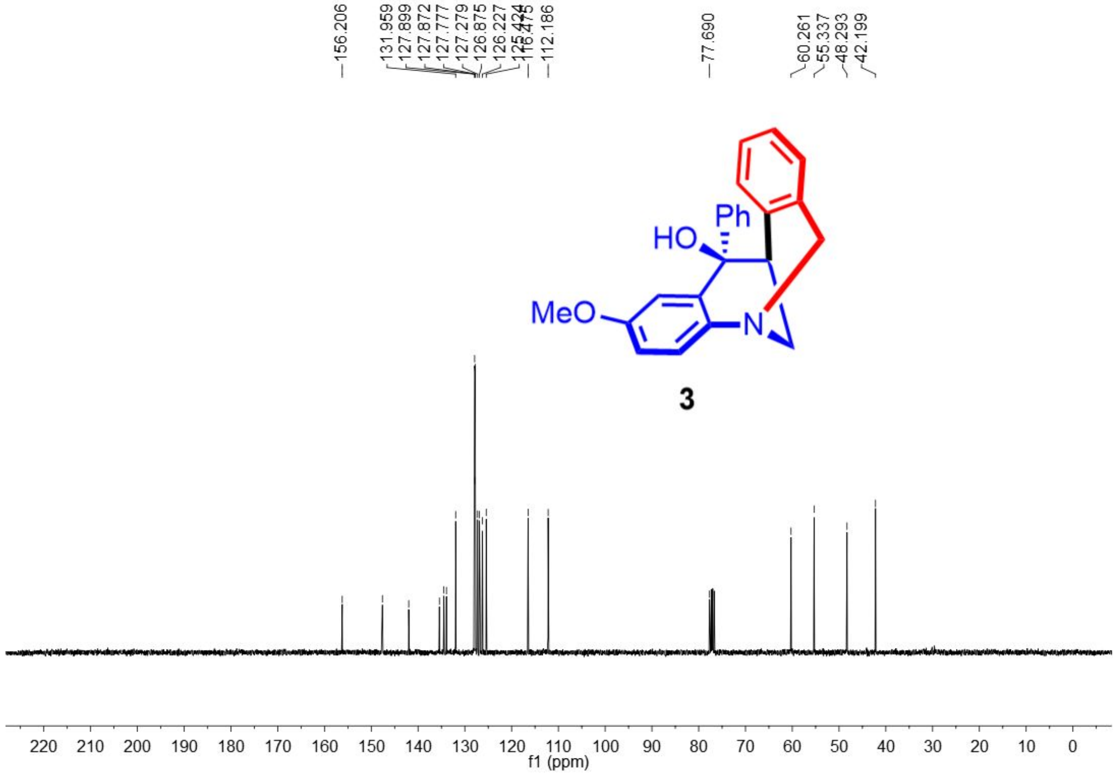

^{1}H NMR (400 MHz, $CDCl_3$) of compound **4:**

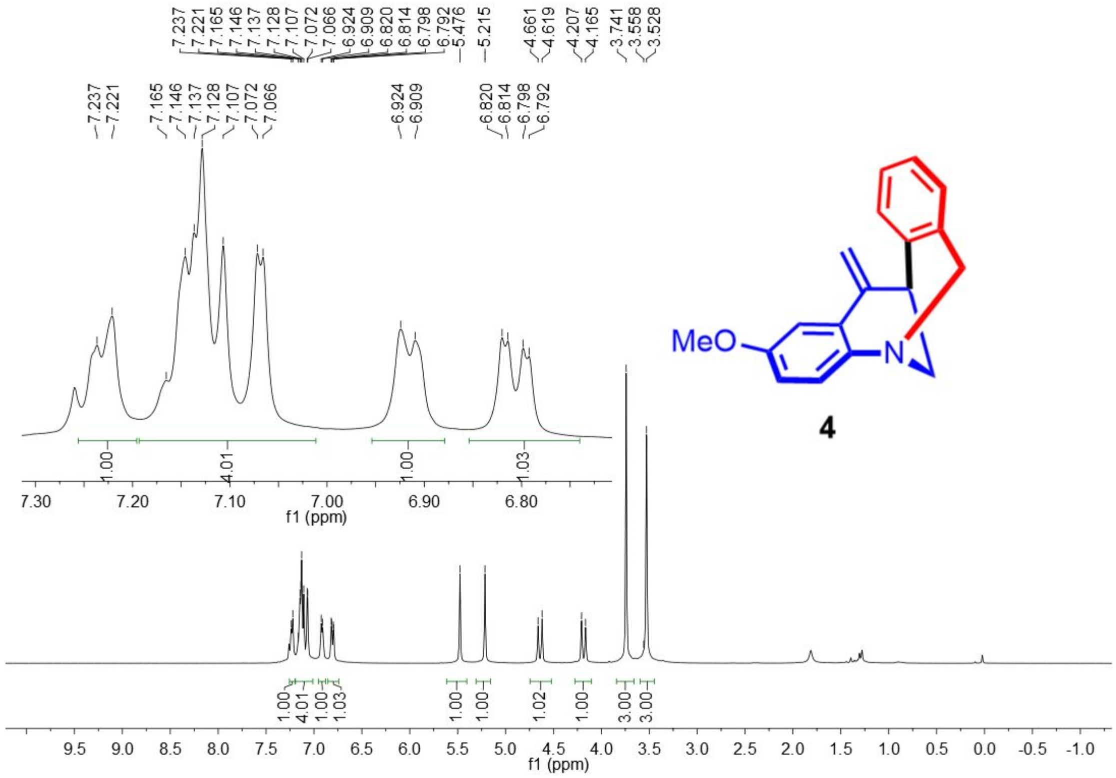

^{13}C NMR (100 MHz, $CDCl_3$) of compound **4:**

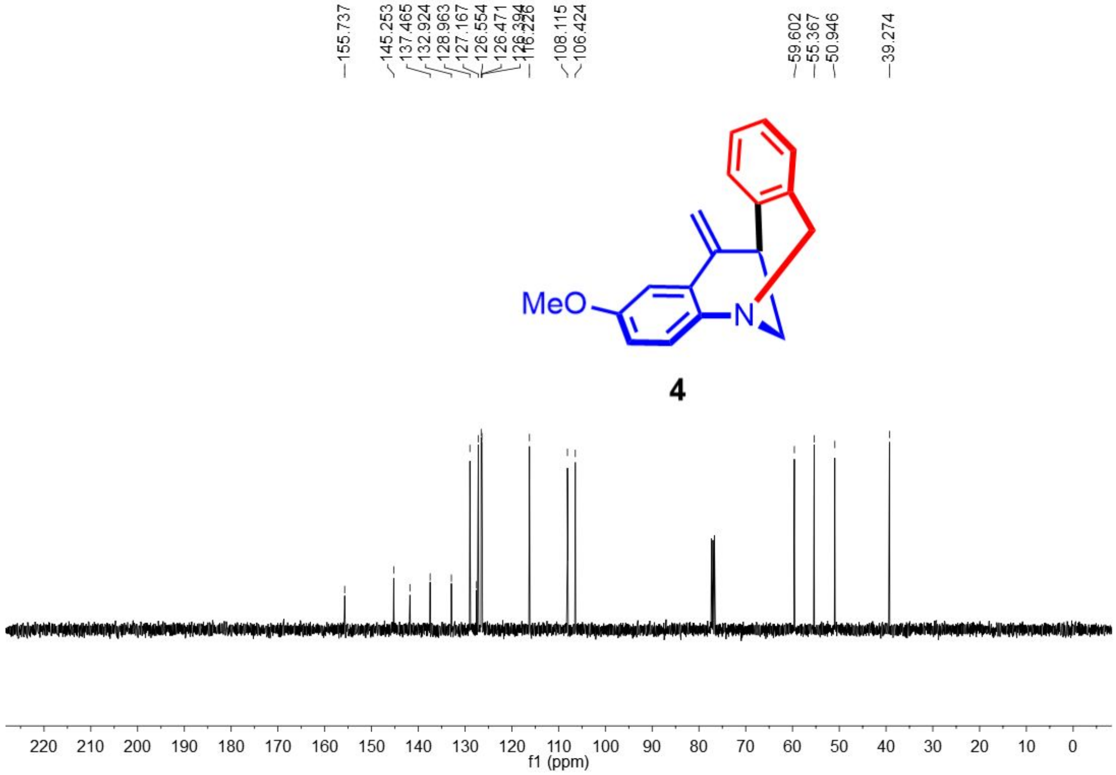

^{1}H NMR (400 MHz, $CDCl_3$) of compound **5:**

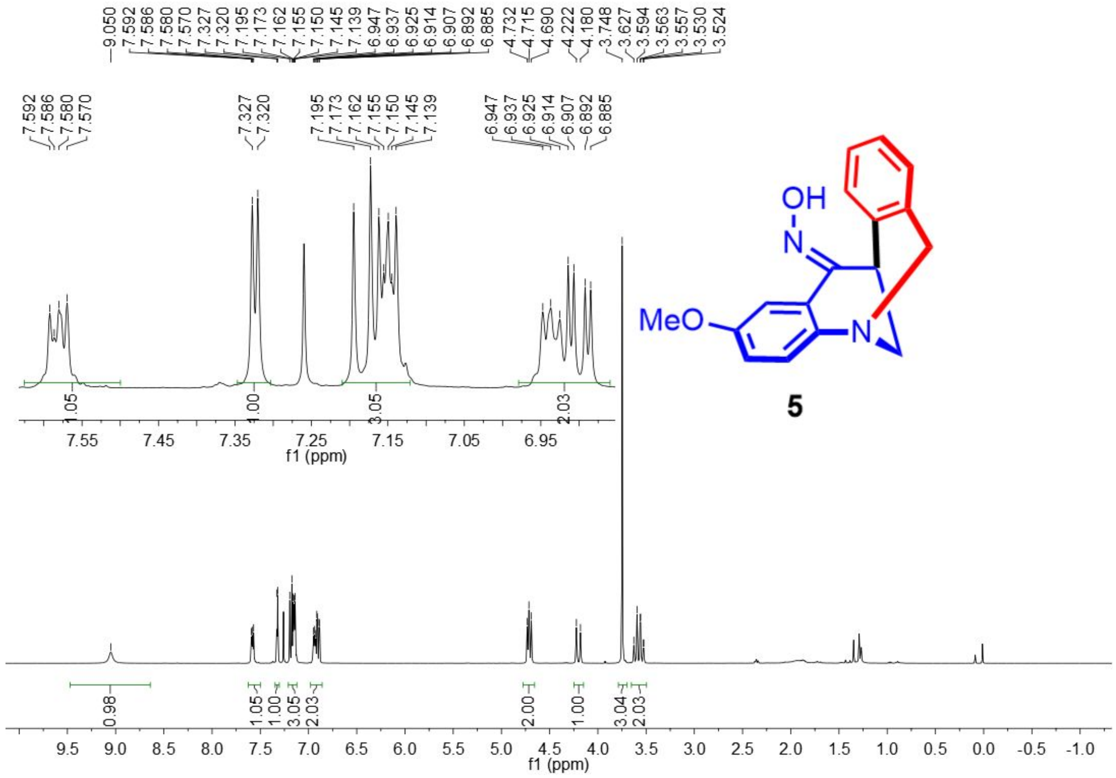

^{13}C NMR (100 MHz, $CDCl_3$) of compound **5:**

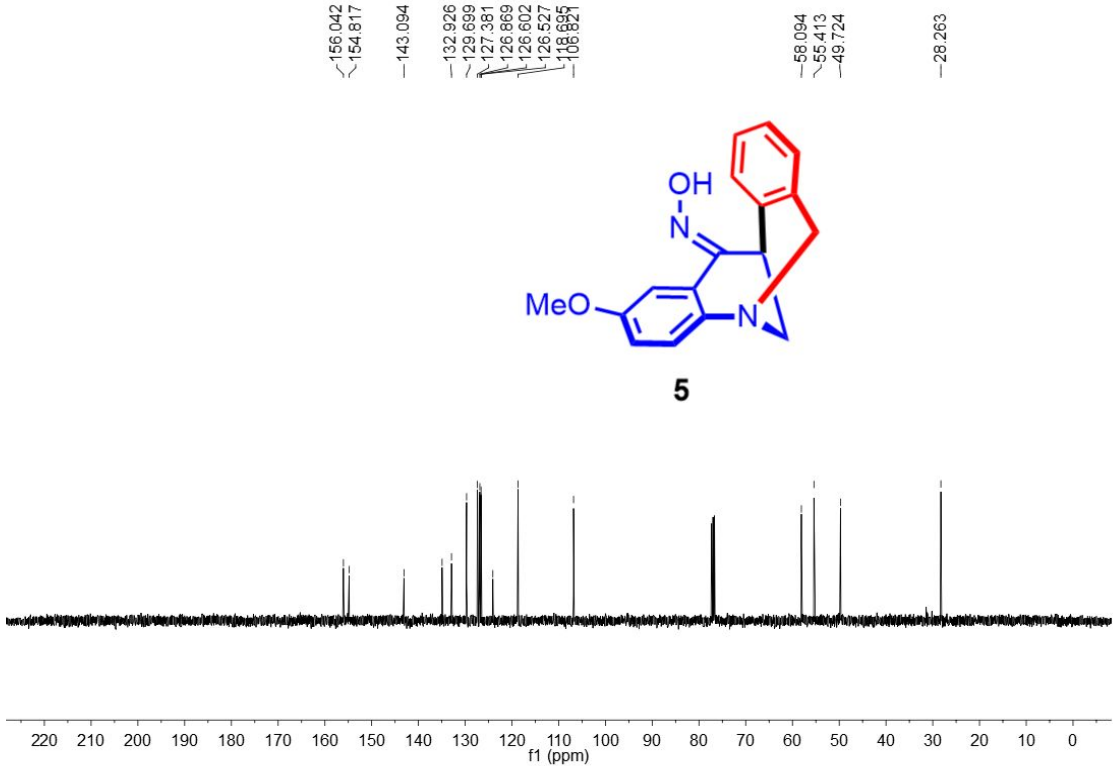

^{1}H NMR (400 MHz, $CDCl_3$) of compound **6:**

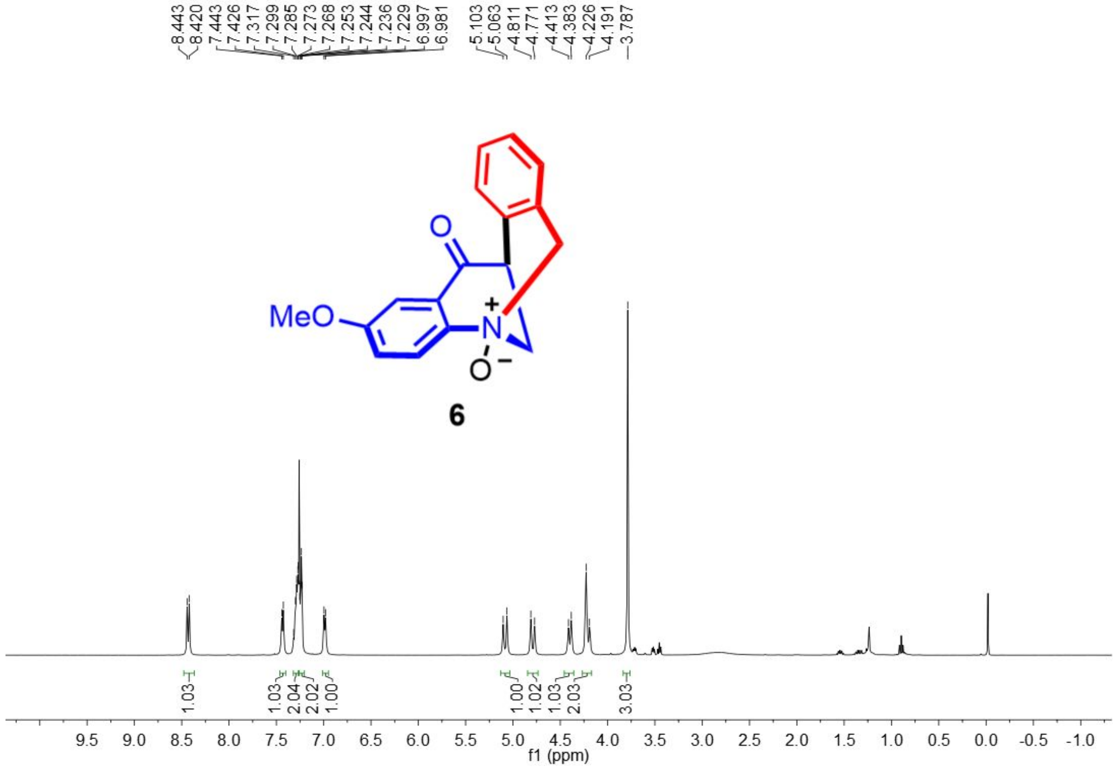

^{13}C NMR (100 MHz, $CDCl_3$) of compound **6:**

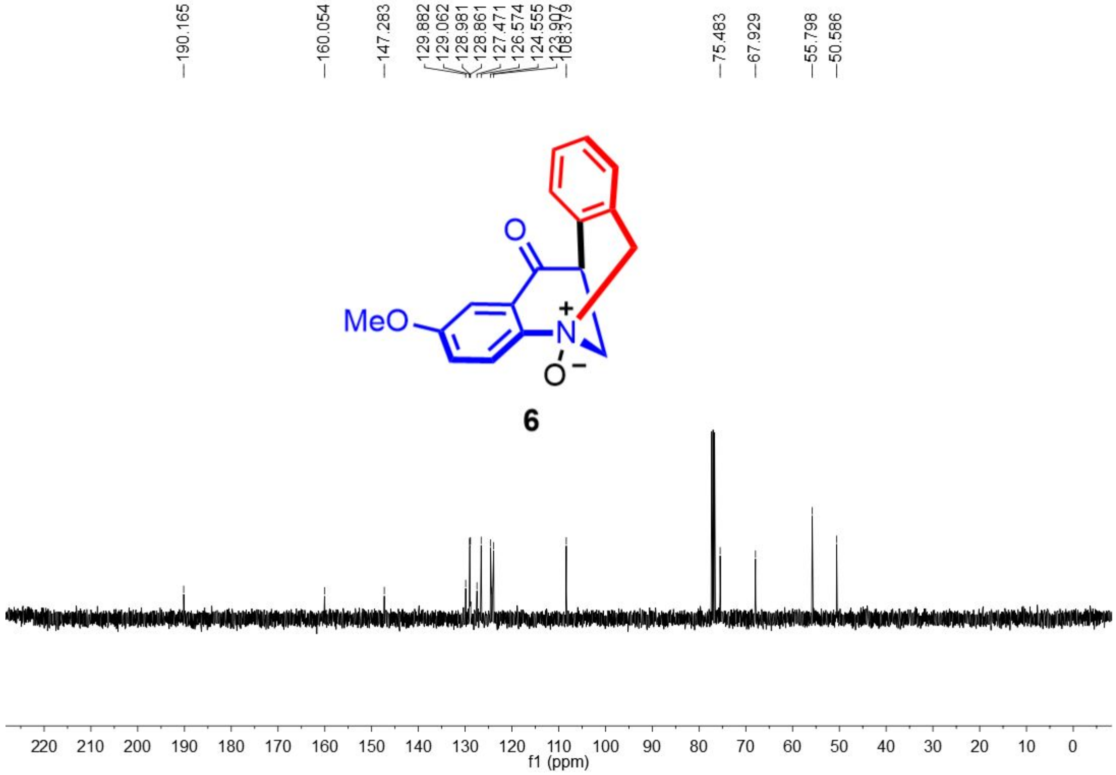

^{1}H NMR (400 MHz, $CDCl_3$) of compound **7:**

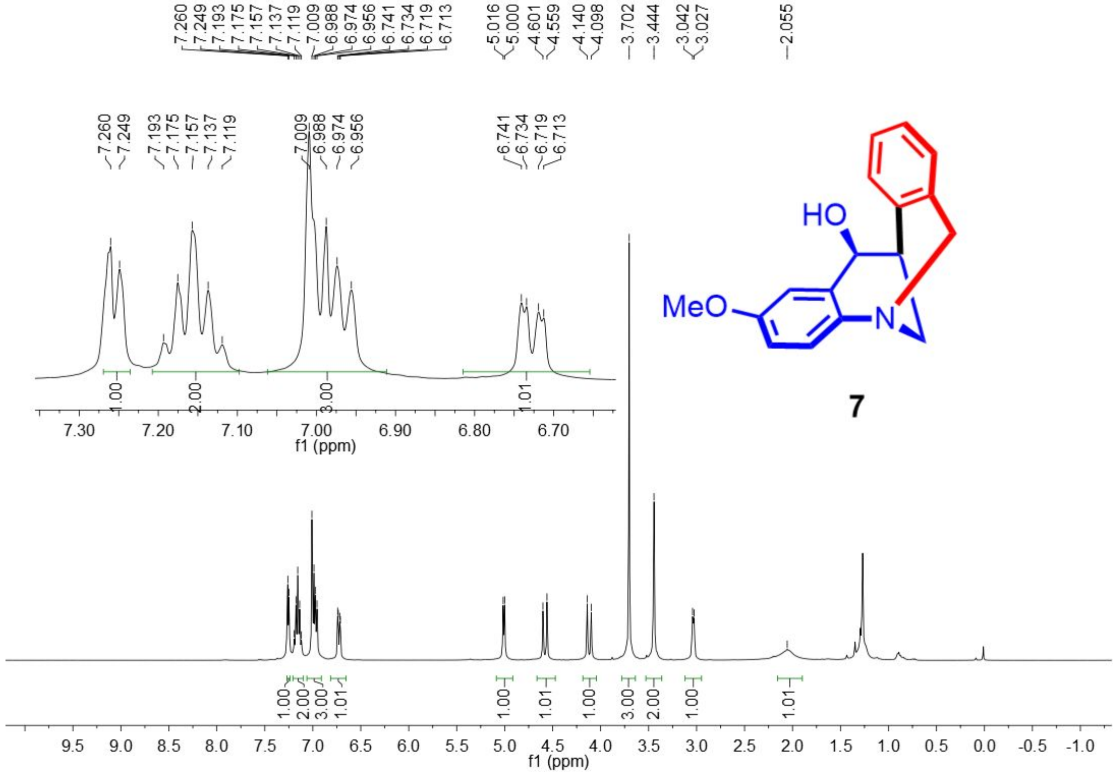

^{13}C NMR (100 MHz, $CDCl_3$) of compound **7:**

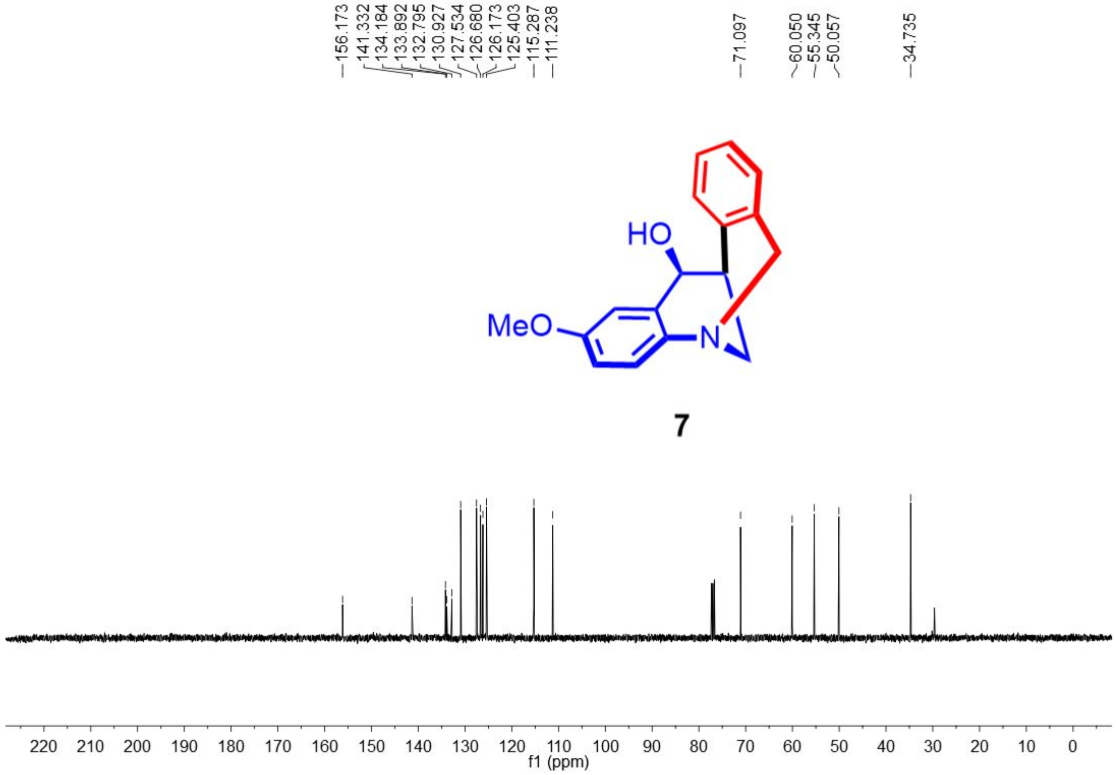

^{1}H NMR (400 MHz, DMSO- d_6) of compound **10:**

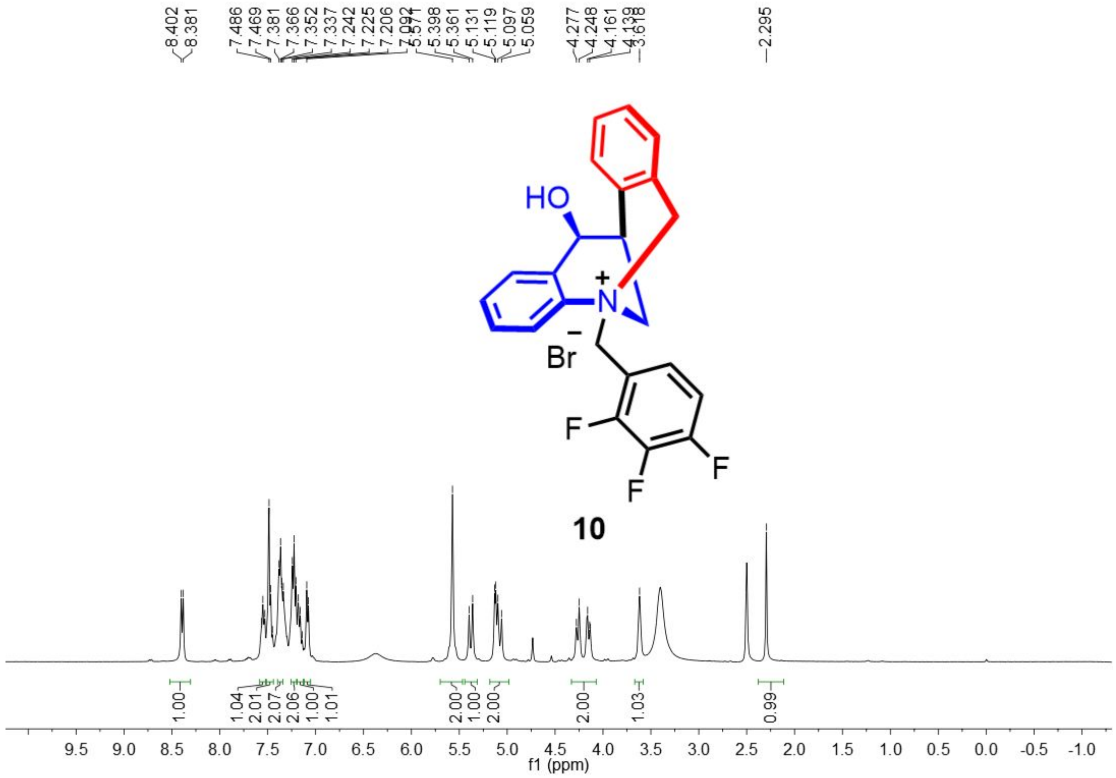

^{13}C NMR (100 MHz, DMSO-d_6) of compound **10:**

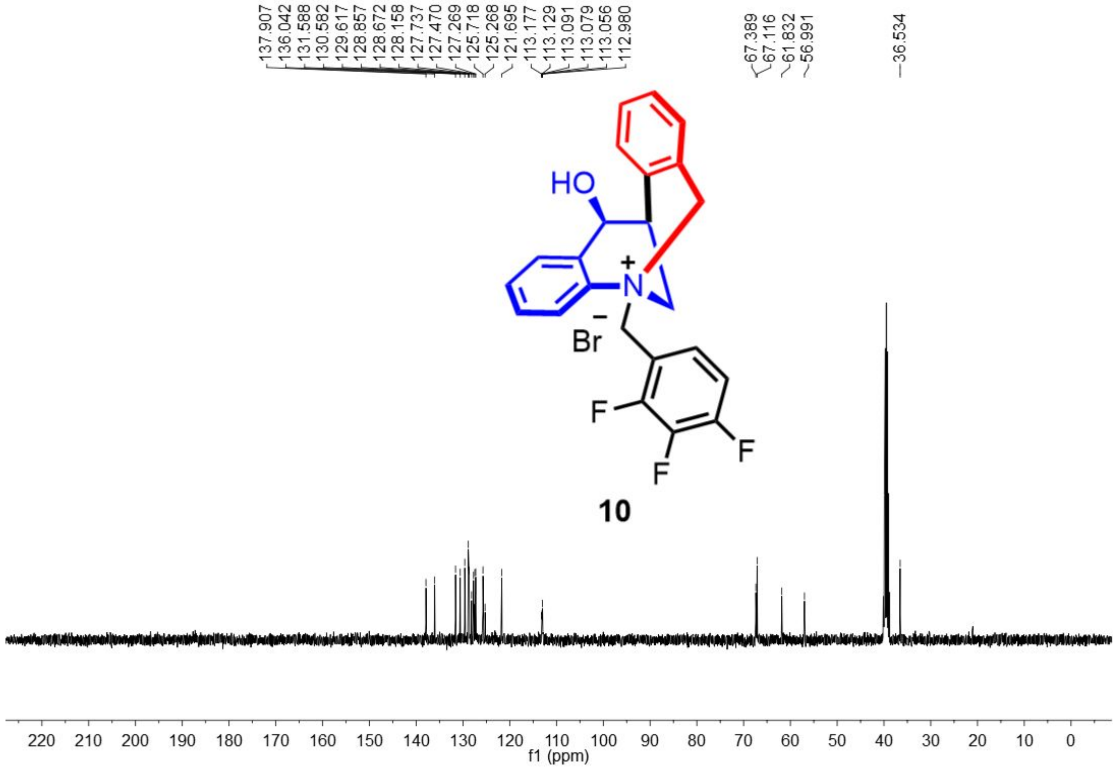

^{1}H NMR (400 MHz, $CDCl_3$) of compound **11:**

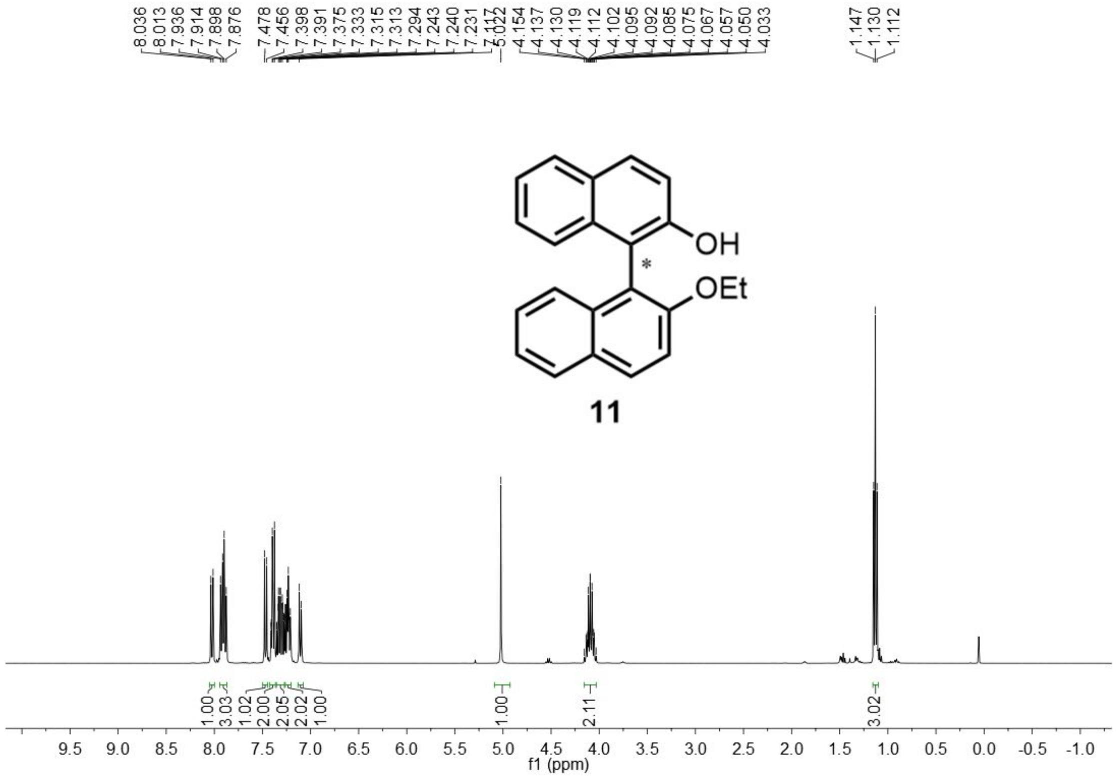

^{13}C NMR (100 MHz, $CDCl_3$) of compound **11:**

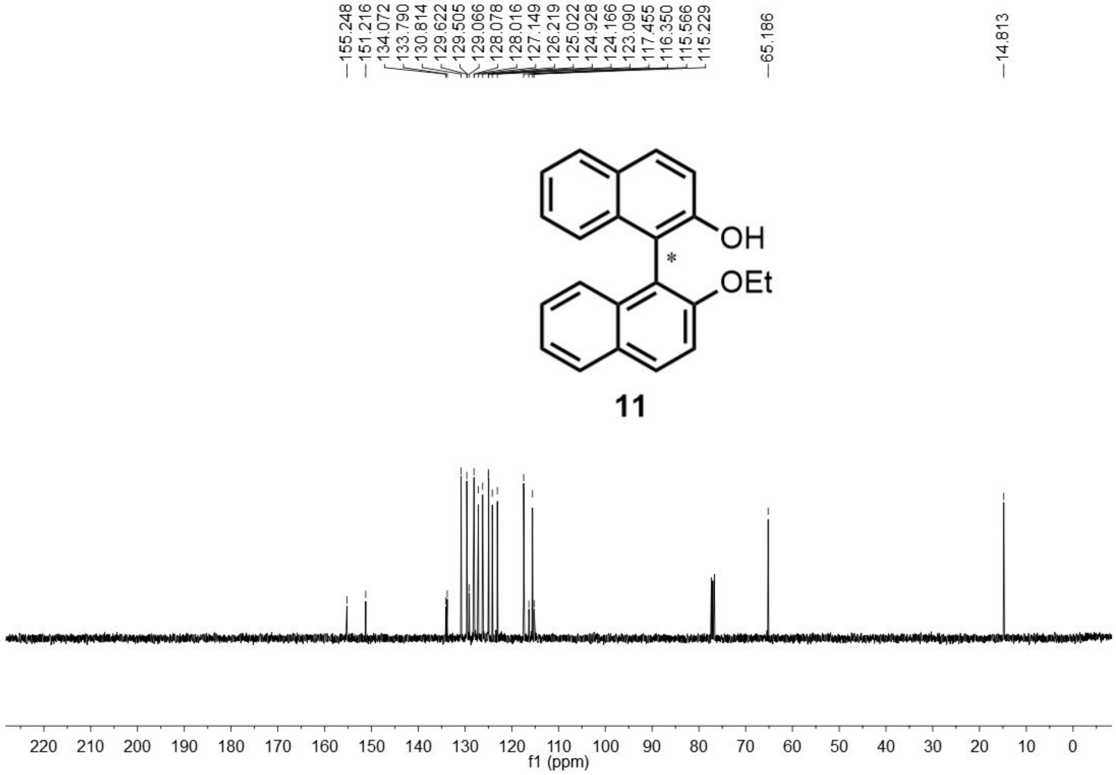

^{1}H NMR (400 MHz, $CDCl_3$) of compound **13:**

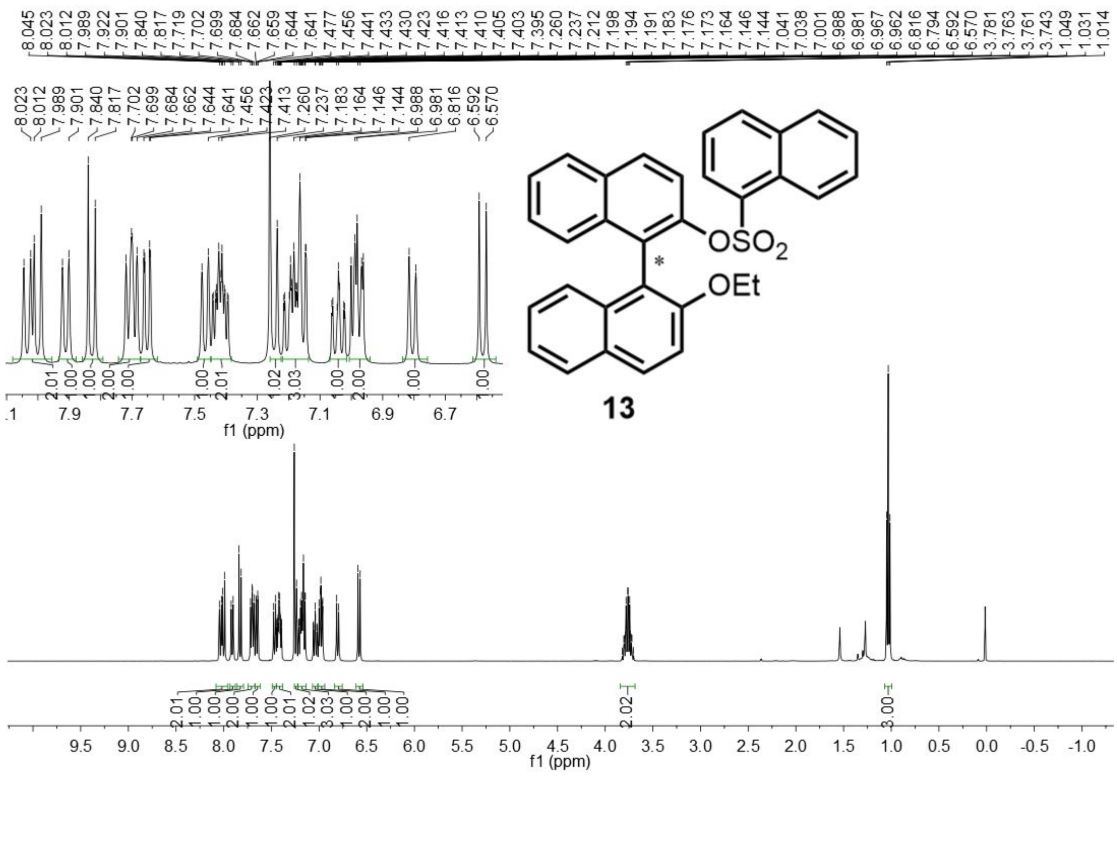

^{13}C NMR (100 MHz, $CDCl_3$) of compound **13:**

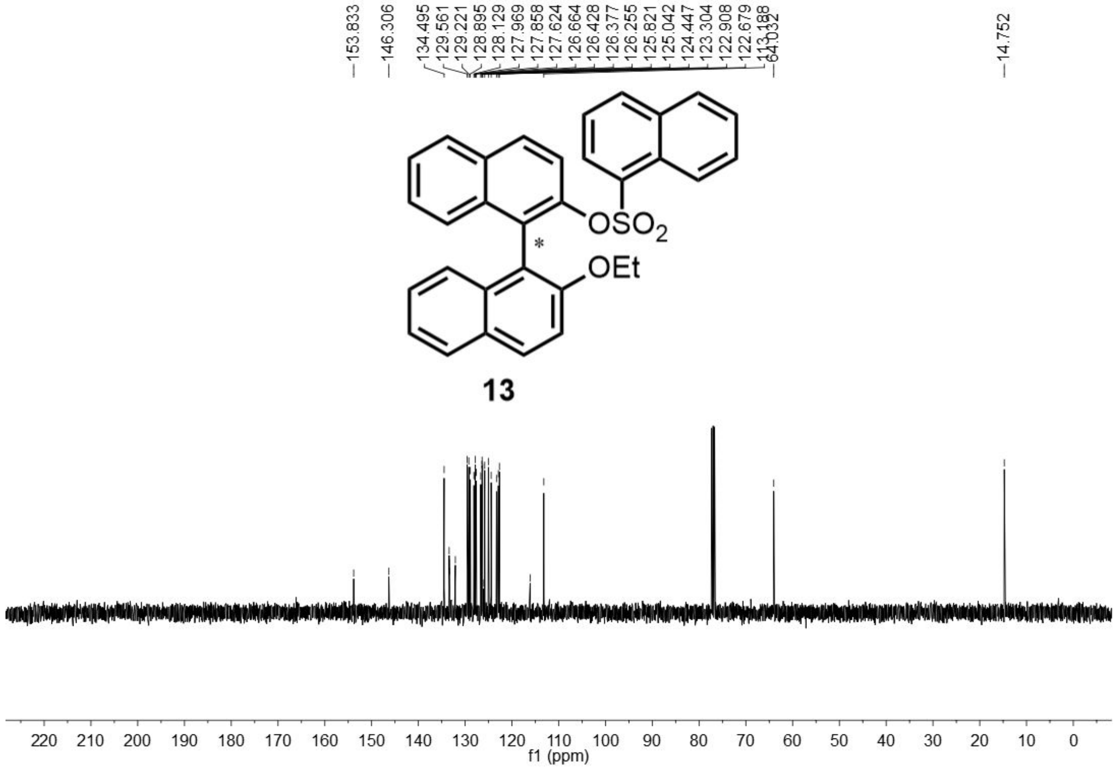

^{1}H NMR (400 MHz, $CDCl_3$) of compound **16:**

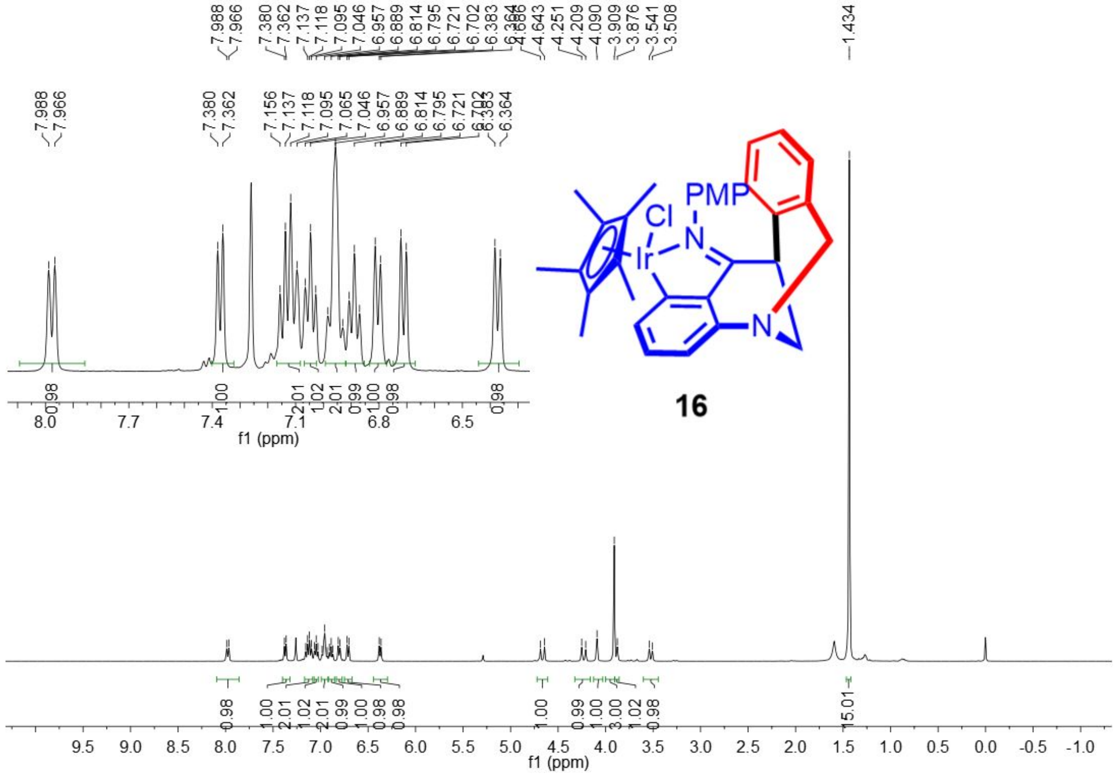

^{13}C NMR (100 MHz, $CDCl_3$) of compound **16:**

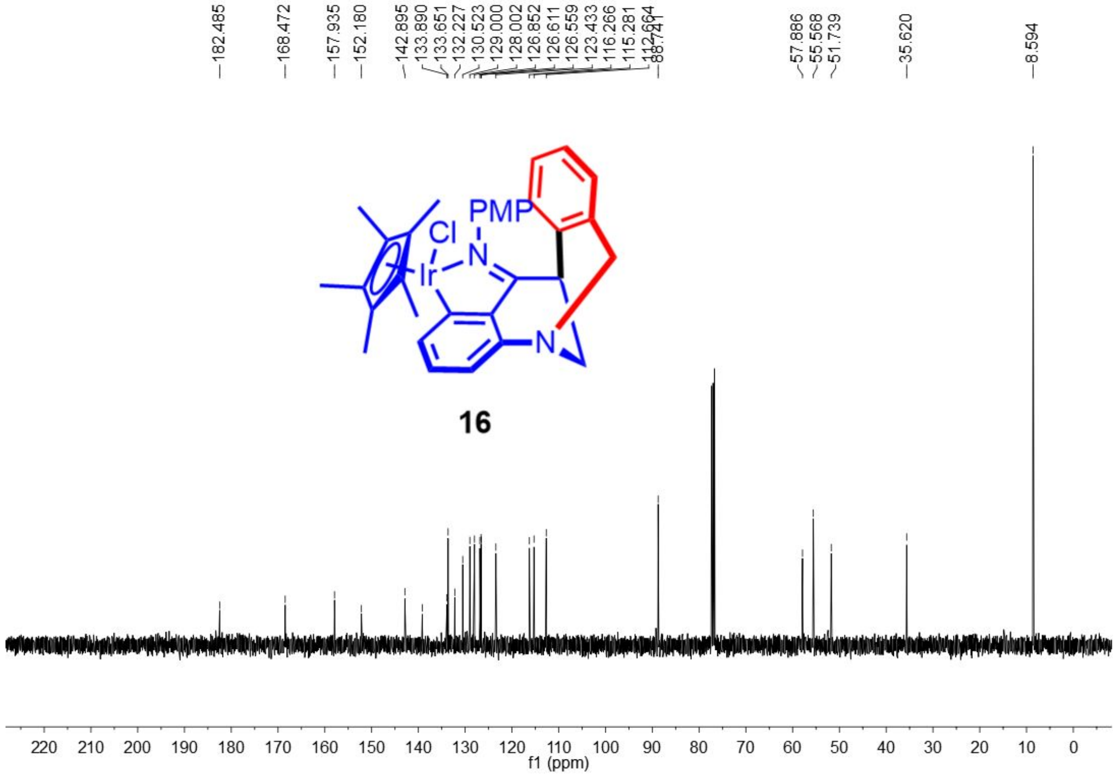

^{1}H NMR (400 MHz, $CDCl_3$) of compound **17:**

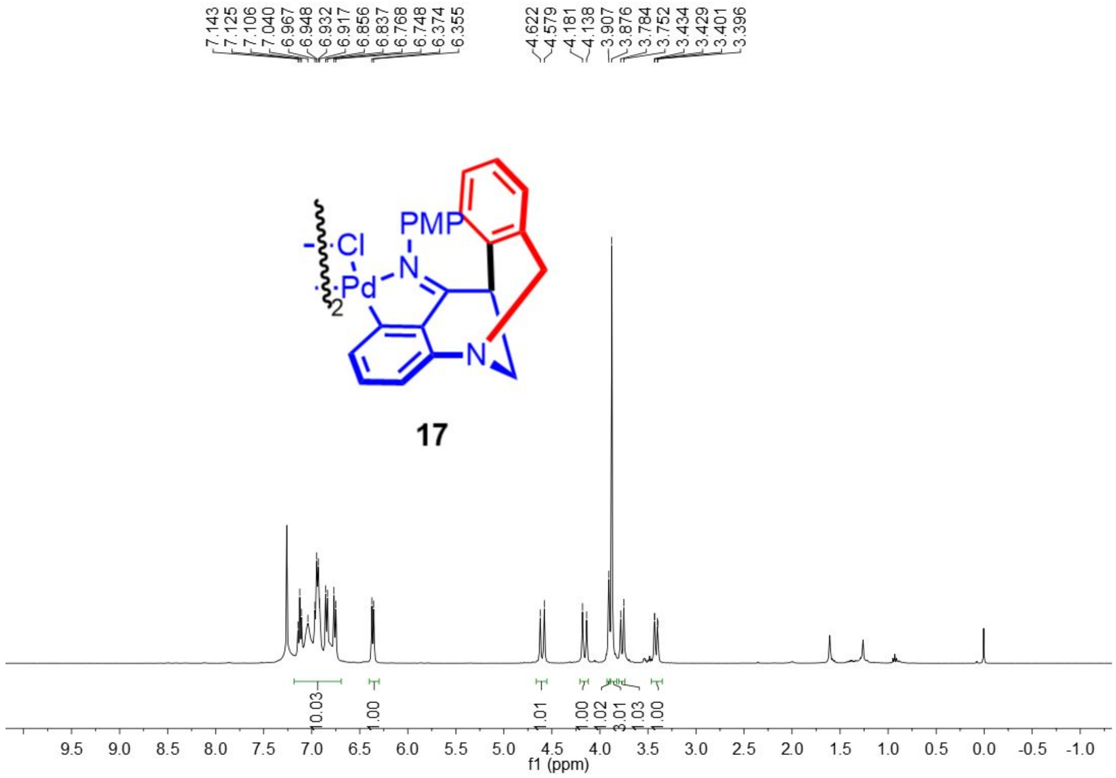

^{13}C NMR (100 MHz, $CDCl_3$) of compound **17:**

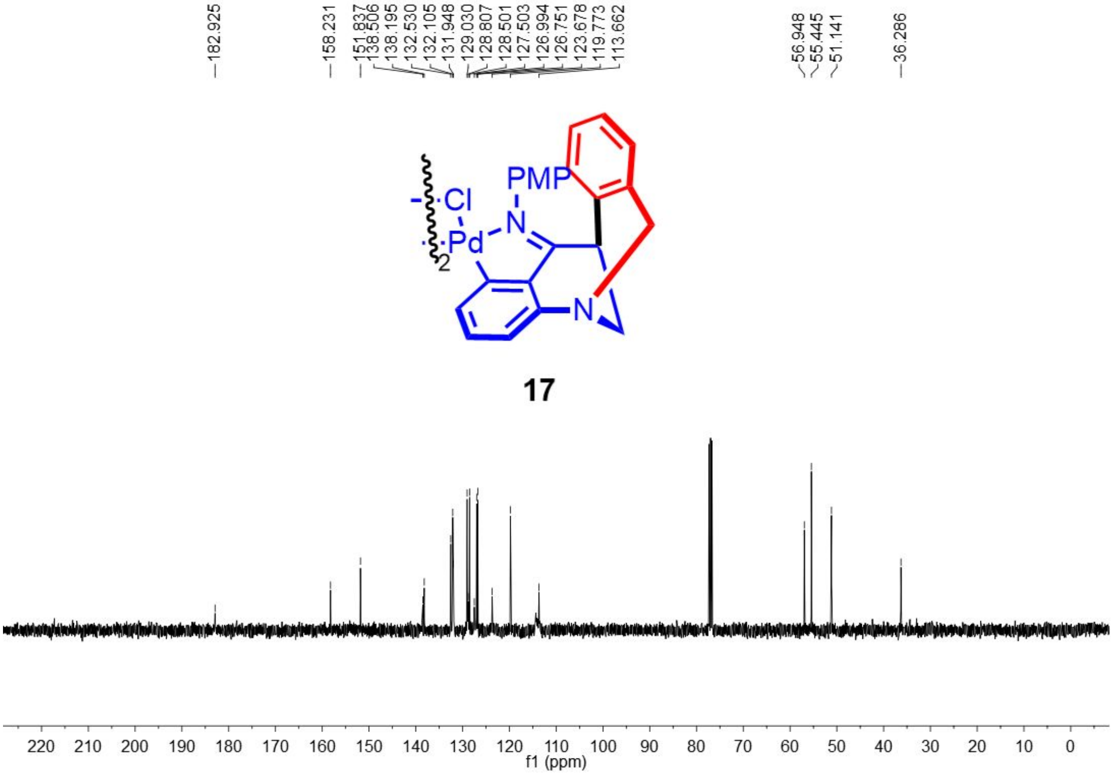

^{1}H NMR (400 MHz, $CDCl_3$) of compound **20:**

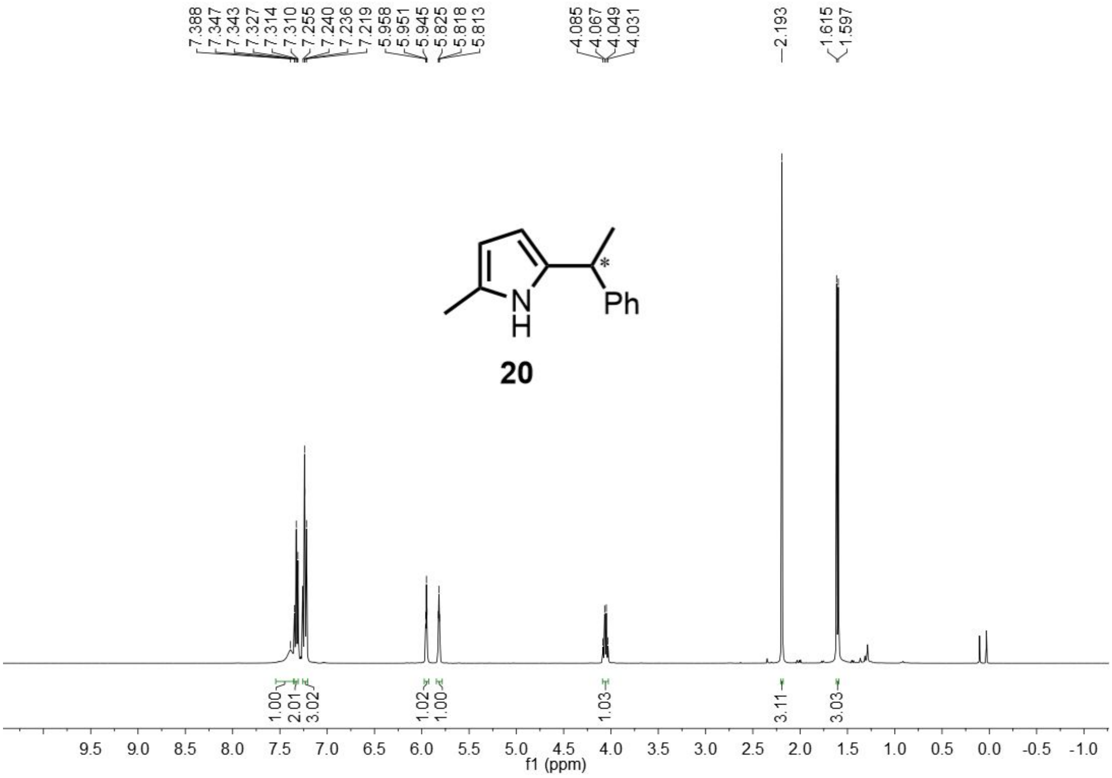

^{13}C NMR (100 MHz, $CDCl_3$) of compound **20:**

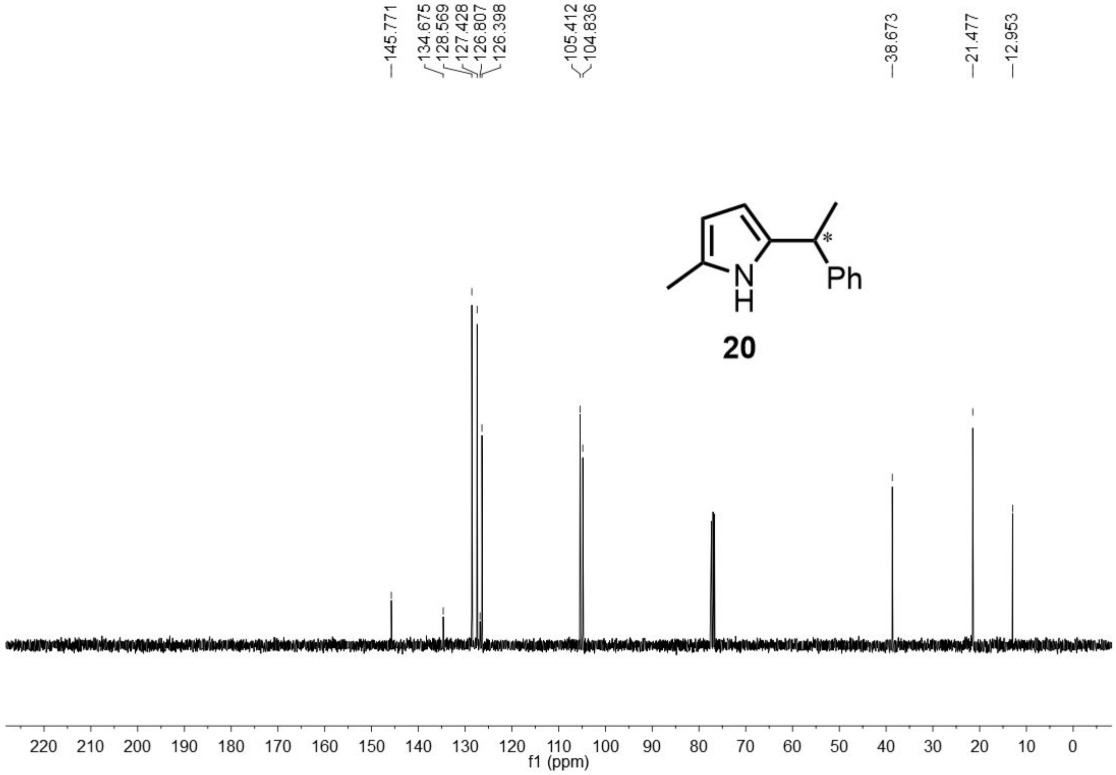

^{1}H NMR (400 MHz, $CDCl_3$) of compound **23:**

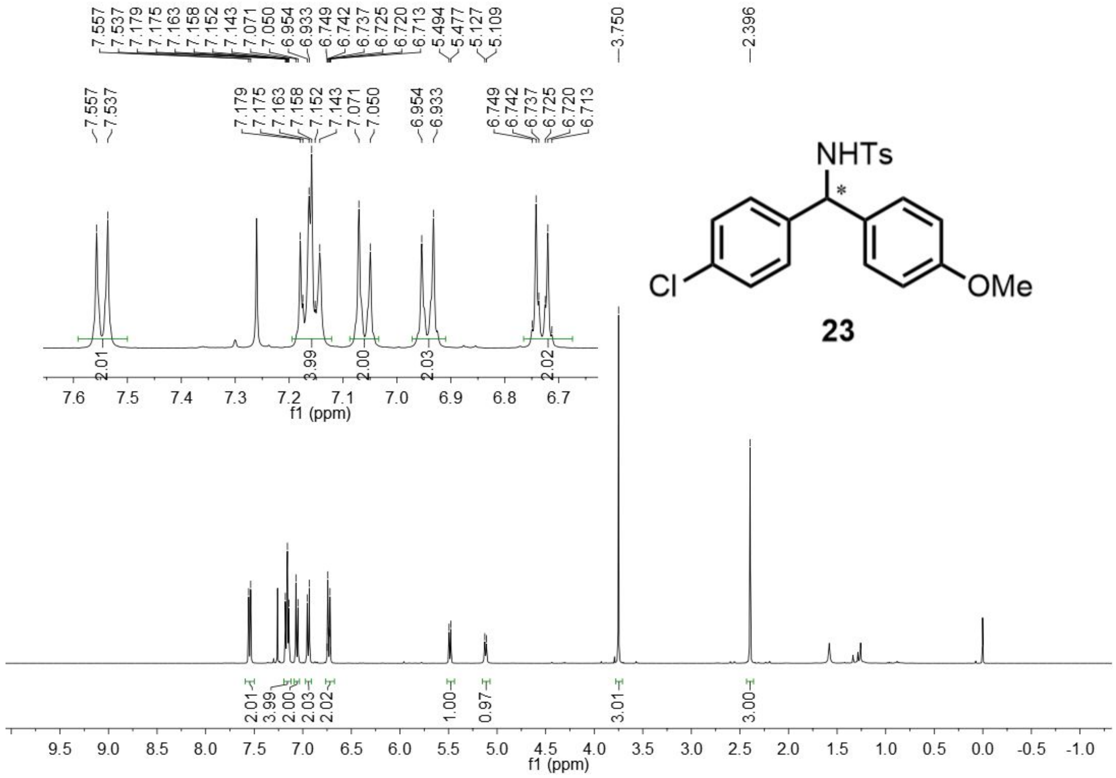

^{13}C NMR (100 MHz, $CDCl_3$) of compound **23:**

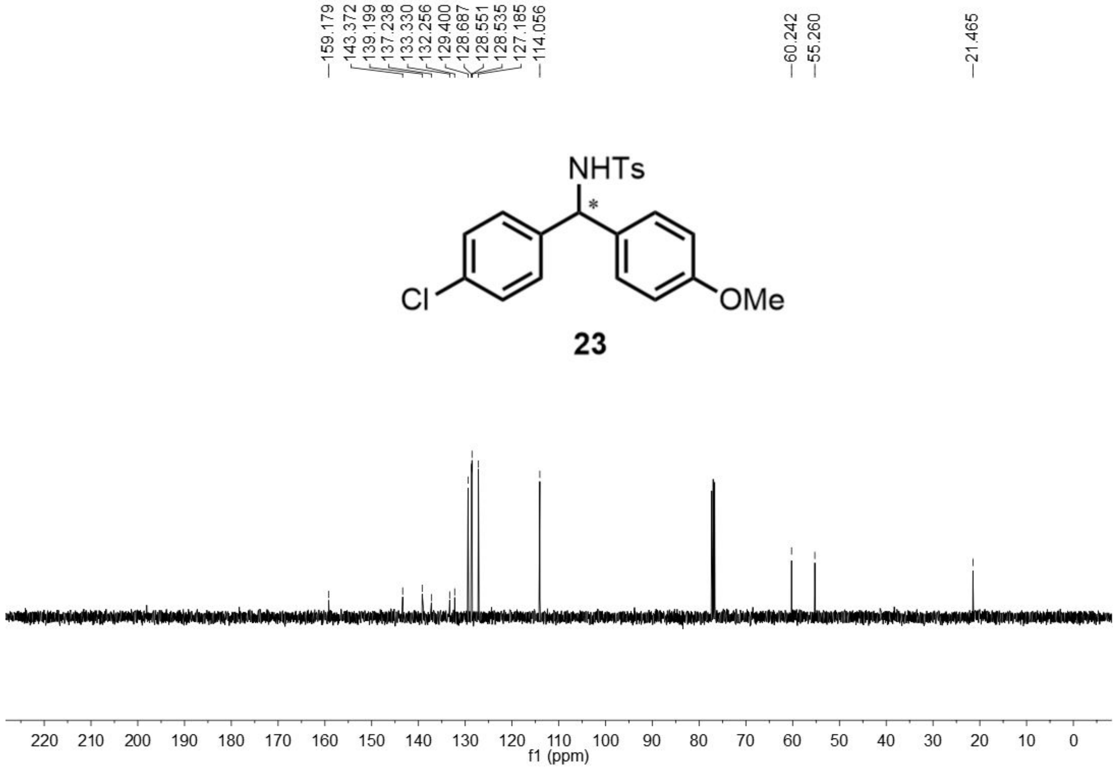

^{1}H NMR (400 MHz, $CDCl_3$) of compound **GF2:**

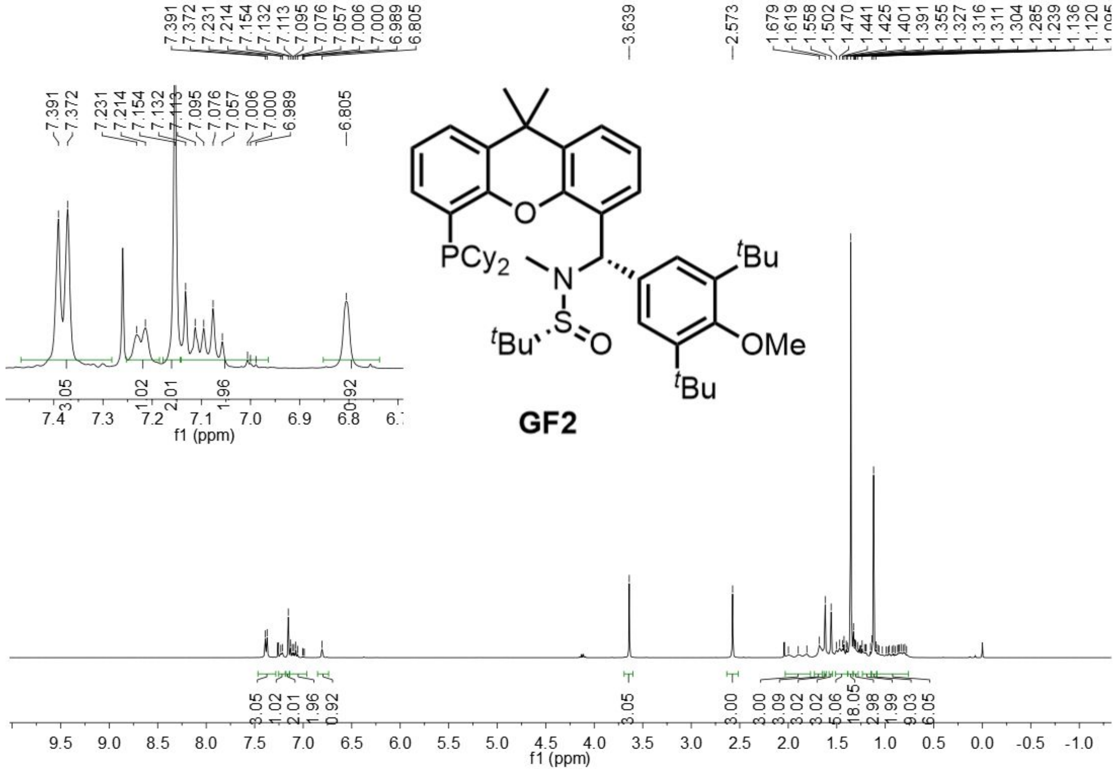

^{13}C NMR (100 MHz, $CDCl_3$) of compound **GF2:**

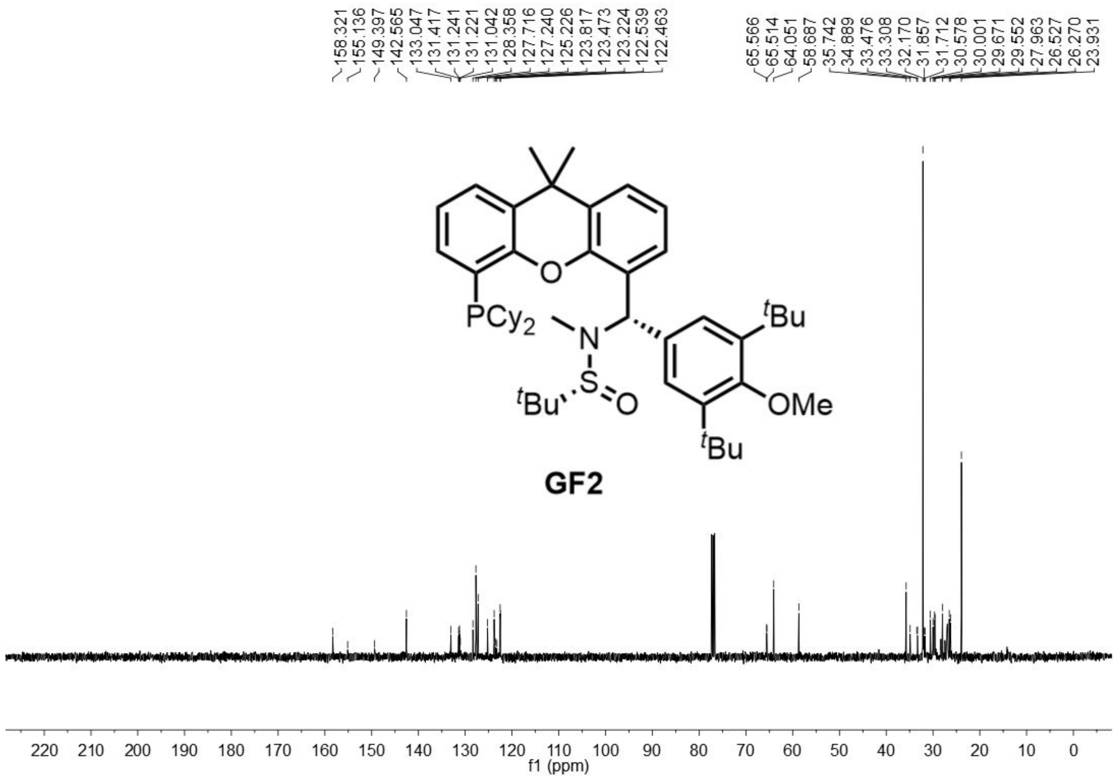

^{31}P NMR (162 MHz, $CDCl_3$) of compound **GF2:**

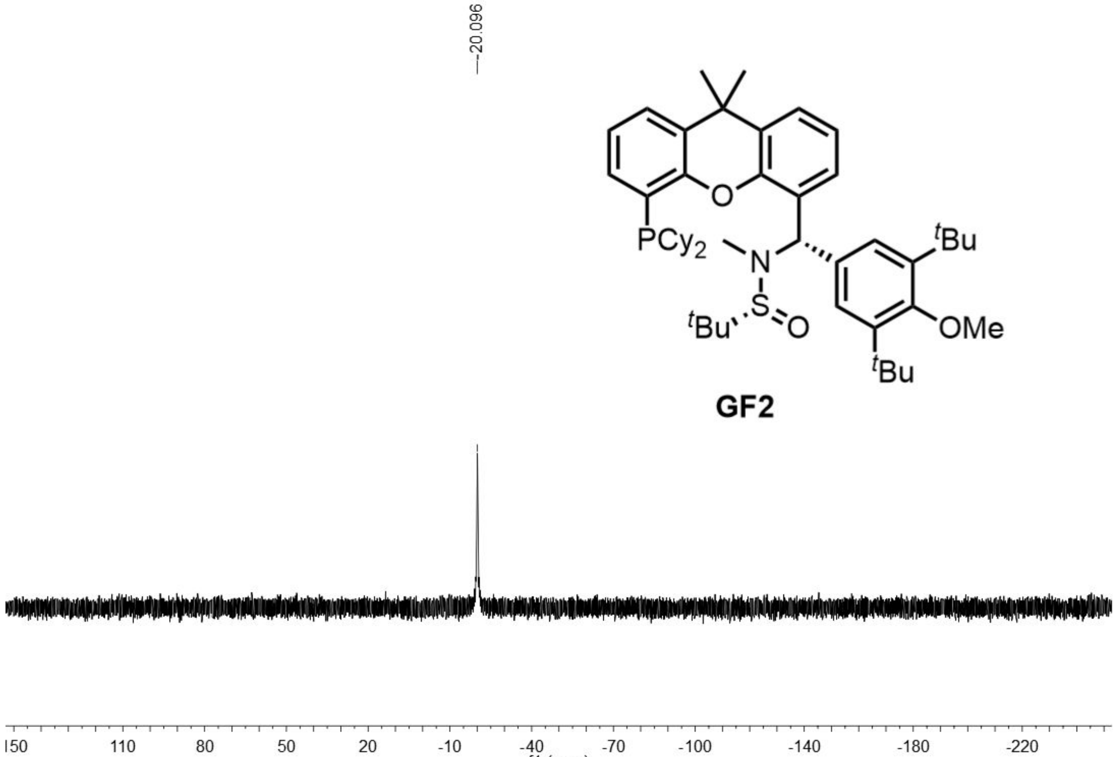

14. HPLC Spectra

Racemic:

<Chromatogram>

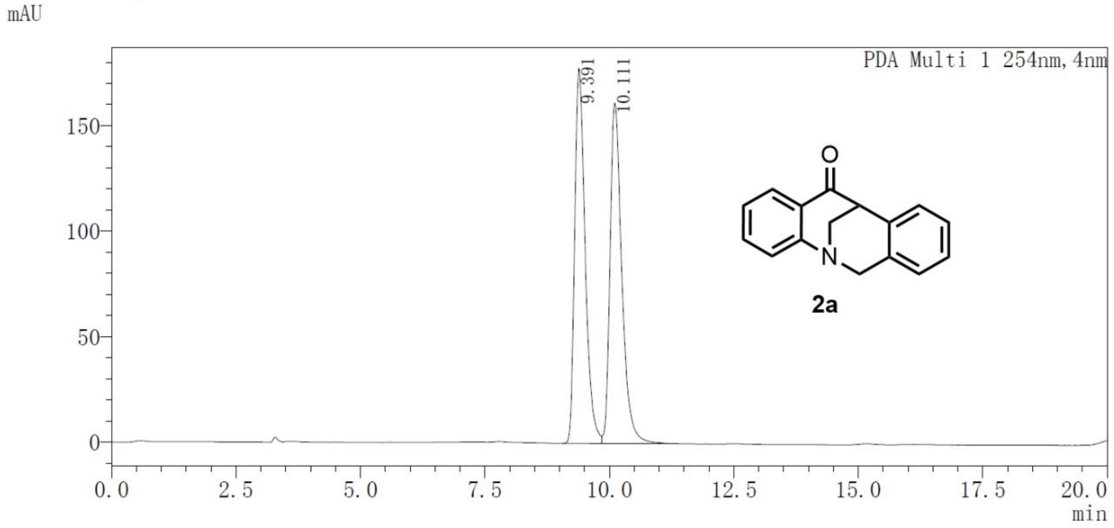

<Peak Table>

PDA Ch1 254nm

No.	Ret.Time(min)	Height(uAU)	Height%	Area(uAU*min)	Area%
1	9.391	177617	52.393	2602172	49.443
2	10.111	161391	47.607	2660757	50.557
Total		339008	100.000	5262929	100.000

Enantioselective:

<Chromatogram>

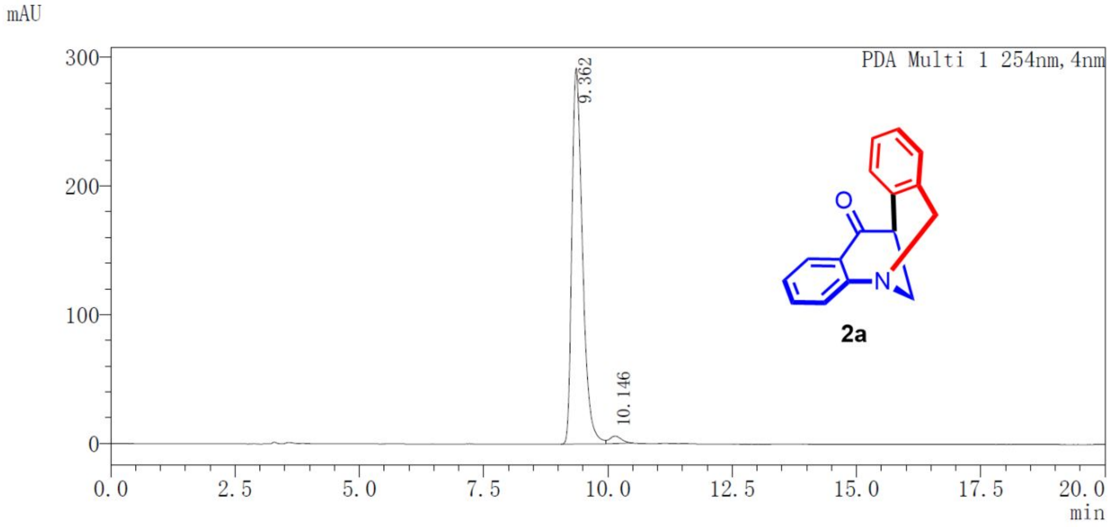

<Peak Table>

PDA Ch1 254nm

No.	Ret.Time(min)	Height(uAU)	Height%	Area(uAU*min)	Area%
1	9.362	291805	98.062	4344831	97.813
2	10.146	5767	1.938	97133	2.187
Total		297572	100.000	4441964	100.000

Racemic:

〈Chromatogram〉
mAU

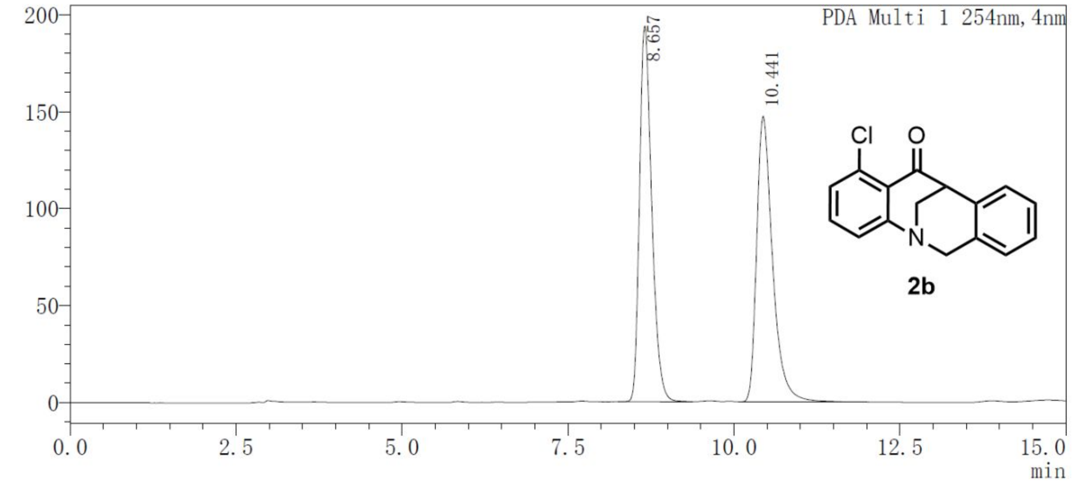

〈Peak Table〉

PDA Ch1 254nm

No.	Ret.Time(min)	Height(uAU)	Height%	Area(uAU*min)	Area%
1	8.657	193591	56.800	2490581	50.325
2	10.441	147236	43.200	2458455	49.675
Total		340827	100.000	4949036	100.000

Enantioselective:

〈Chromatogram〉
mAU

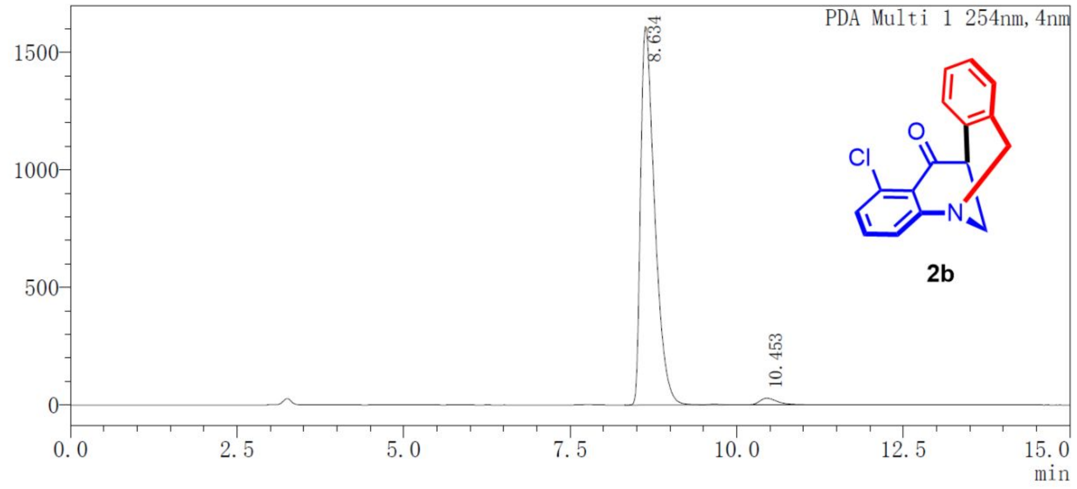

〈Peak Table〉

PDA Ch1 254nm

No.	Ret.Time(min)	Height(uAU)	Height%	Area(uAU*min)	Area%
1	8.634	1608989	98.257	23723358	97.755
2	10.453	28549	1.743	544700	2.245
Total		1637538	100.000	24268058	100.000

Racemic:

<Chromatogram>

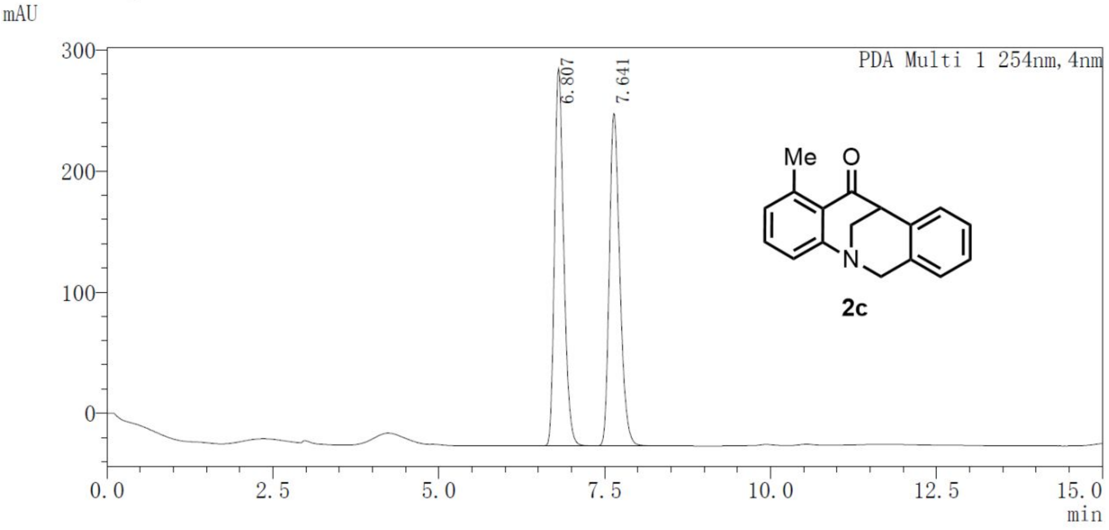

<Peak Table>

PDA Ch1 254nm

No.	Ret. Time (min)	Height (uAU)	Height%	Area (uAU*min)	Area%
1	6.807	311568	53.174	3012457	49.988
2	7.641	274370	46.826	3013897	50.012
Total		585938	100.000	6026354	100.000

Enantioselective:

<Chromatogram>

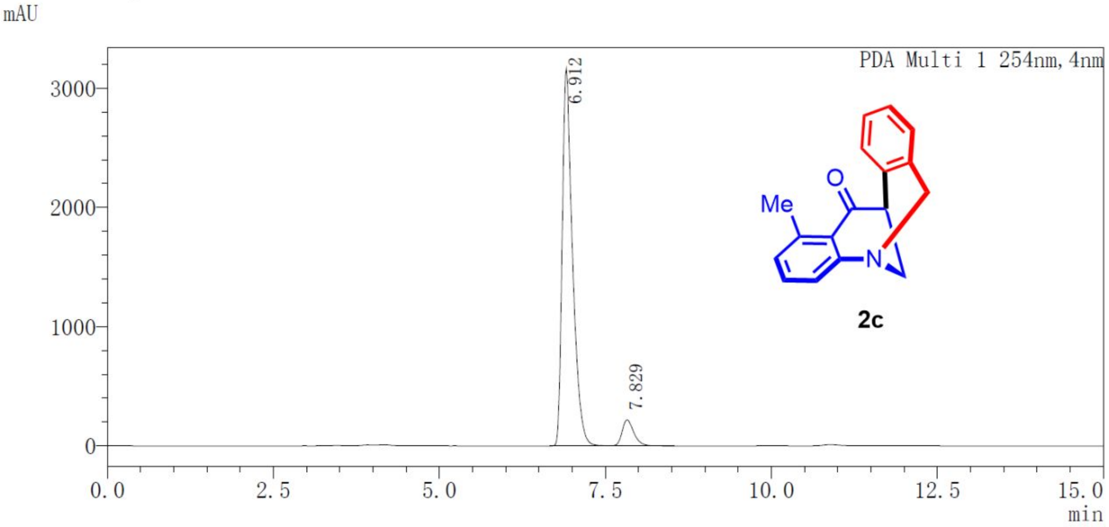

<Peak Table>

PDA Ch1 254nm

No.	Ret. Time (min)	Height (uAU)	Height%	Area (uAU*min)	Area%
1	6.912	3165282	93.537	34125615	92.716
2	7.829	218691	6.463	2680942	7.284
Total		3383973	100.000	36806557	100.000

Racemic:

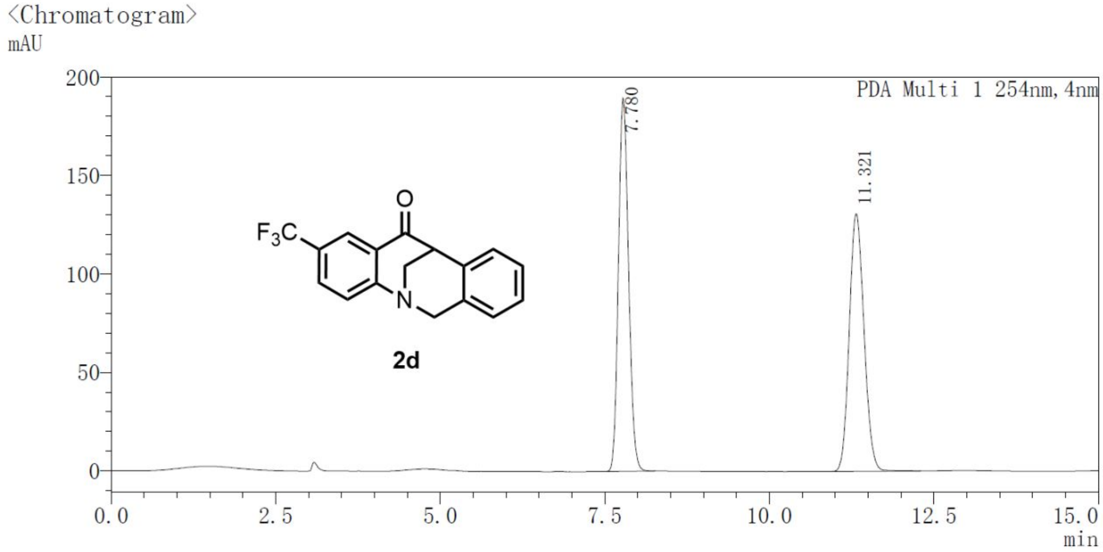

<Peak Table>

PDA Ch1 254nm

No.	Ret. Time (min)	Height (uAU)	Height%	Area (uAU*min)	Area%
1	7.780	189636	59.172	2036414	49.970
2	11.321	130848	40.828	2038842	50.030
Total		320483	100.000	4075256	100.000

Enantioselective:

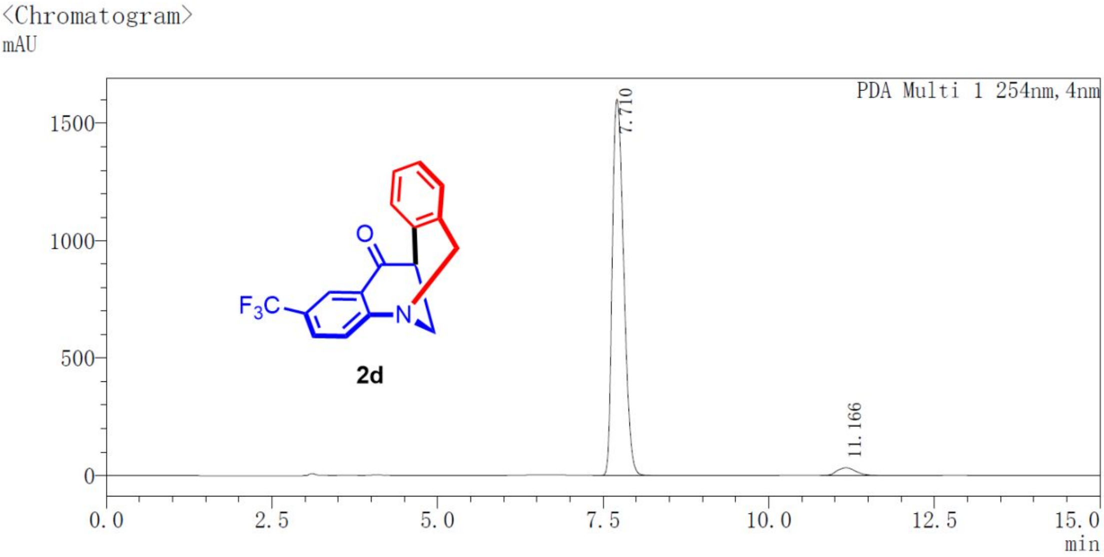

<Peak Table>

PDA Ch1 254nm

No.	Ret. Time (min)	Height (uAU)	Height%	Area (uAU*min)	Area%
1	7.710	1603160	97.998	19245777	96.727
2	11.166	32757	2.002	651316	3.273
Total		1635917	100.000	19897093	100.000

Racemic:

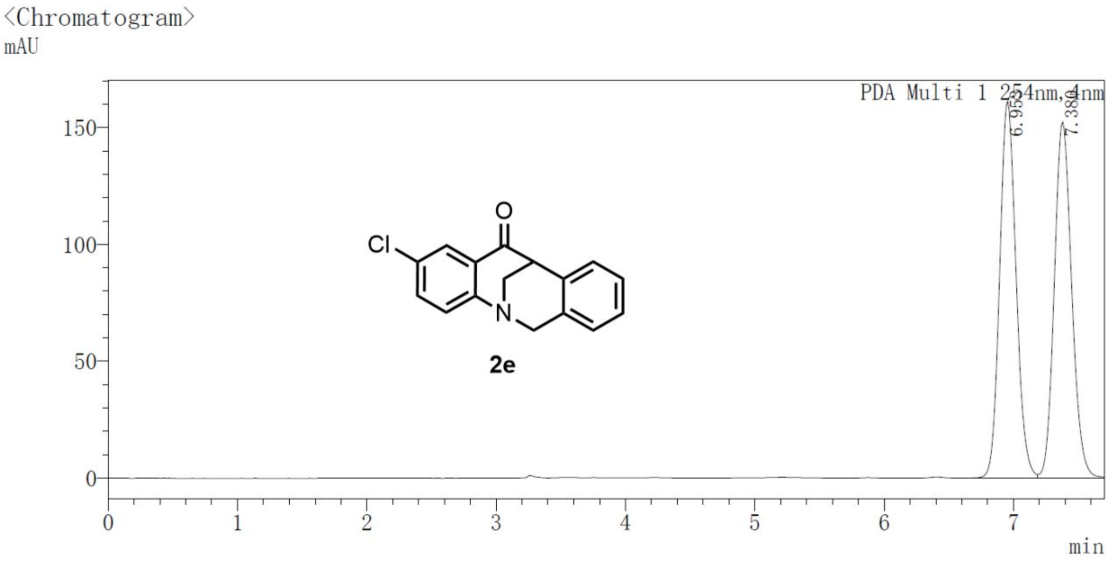

<Peak Table>

PDA Ch1 254nm

No.	Ret. Time (min)	Height (uAU)	Height%	Area (uAU*min)	Area%
1	6.953	161167	51.405	1427398	50.070
2	7.380	152360	48.595	1423417	49.930
Total		313526	100.000	2850815	100.000

Enantioselective:

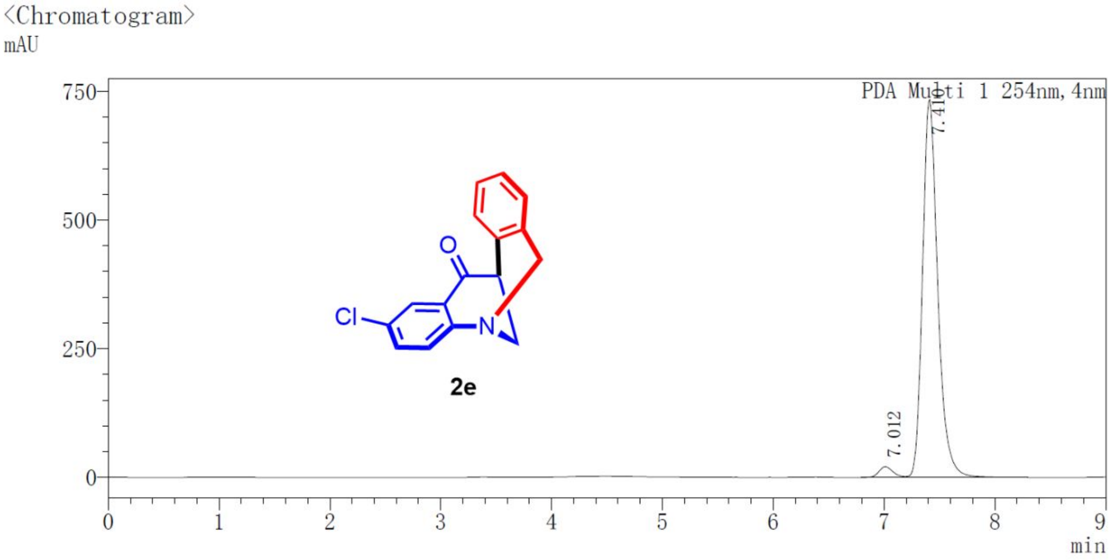

<Peak Table>

PDA Ch1 254nm

No.	Ret. Time (min)	Height (uAU)	Height%	Area (uAU*min)	Area%
1	7.012	20756	2.752	184524	2.571
2	7.410	733514	97.248	6992834	97.429
Total		754270	100.000	7177358	100.000

Racemic:

<Chromatogram>
mAU

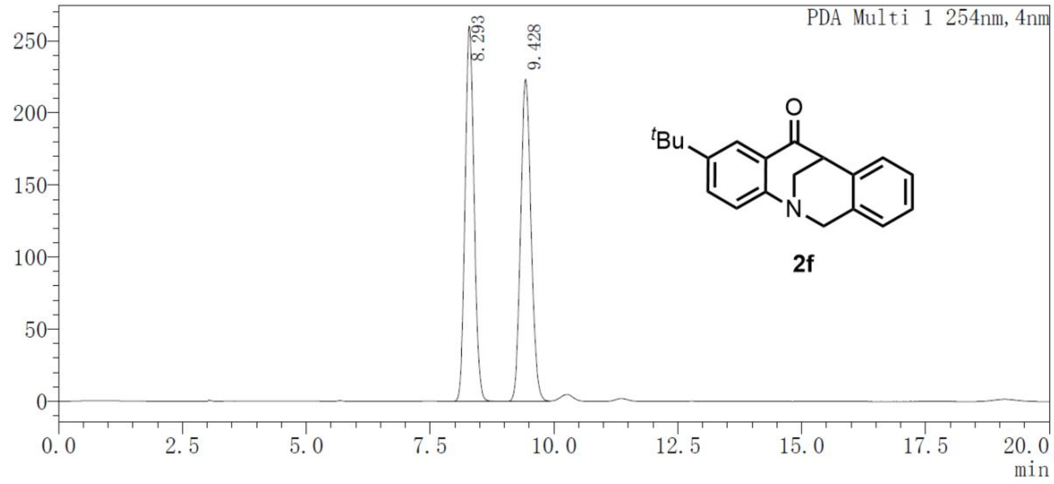

<Peak Table>

PDA Ch1 254nm

No.	Ret. Time (min)	Height (uAU)	Height%	Area (uAU*min)	Area%
1	8.293	260135	53.795	3283668	49.979
2	9.428	223431	46.205	3286425	50.021
Total		483567	100.000	6570093	100.000

Enantioselective:

<Chromatogram>
mAU

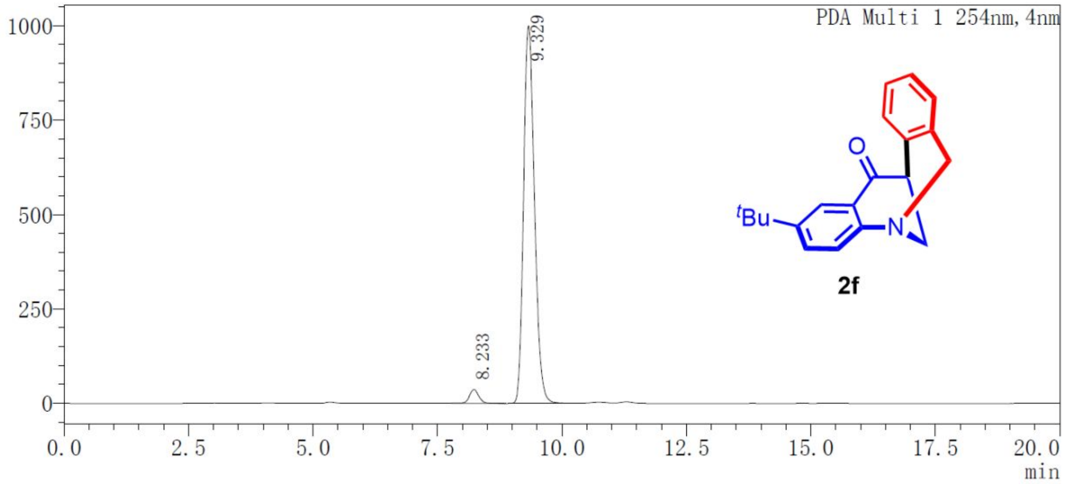

<Peak Table>

PDA Ch1 254nm

No.	Ret. Time (min)	Height (uAU)	Height%	Area (uAU*min)	Area%
1	8.233	36823	3.556	492682	3.137
2	9.329	998727	96.444	15213086	96.863
Total		1035550	100.000	15705769	100.000

Racemic:

<Chromatogram>

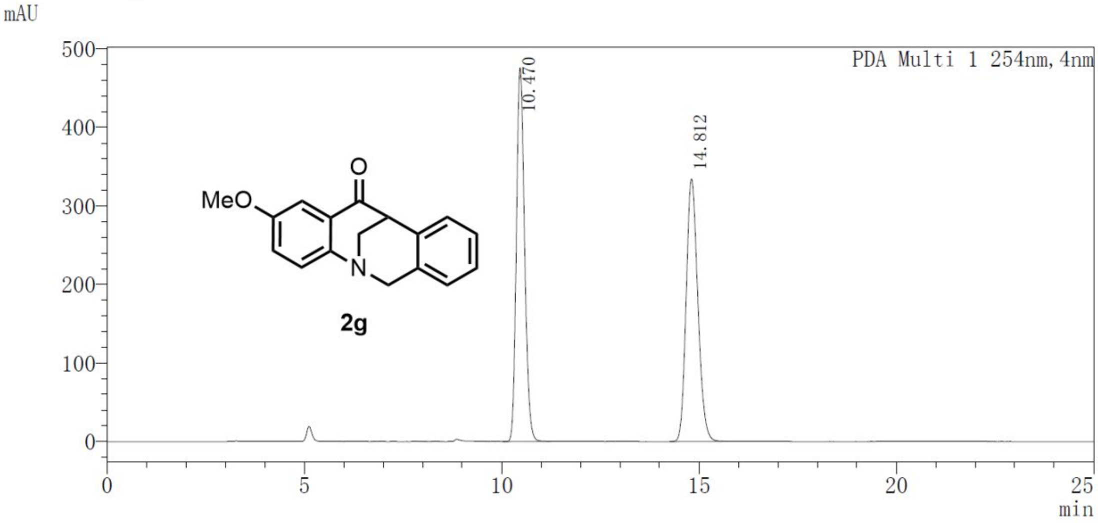

<Peak Table>

PDA Ch1 254nm

No.	Ret. Time (min)	Height (uAU)	Height%	Area (uAU*min)	Area%
1	10.470	475614	58.734	6876885	49.989
2	14.812	334162	41.266	6879977	50.011
Total		809776	100.000	13756862	100.000

Enantioselective:

<Chromatogram>

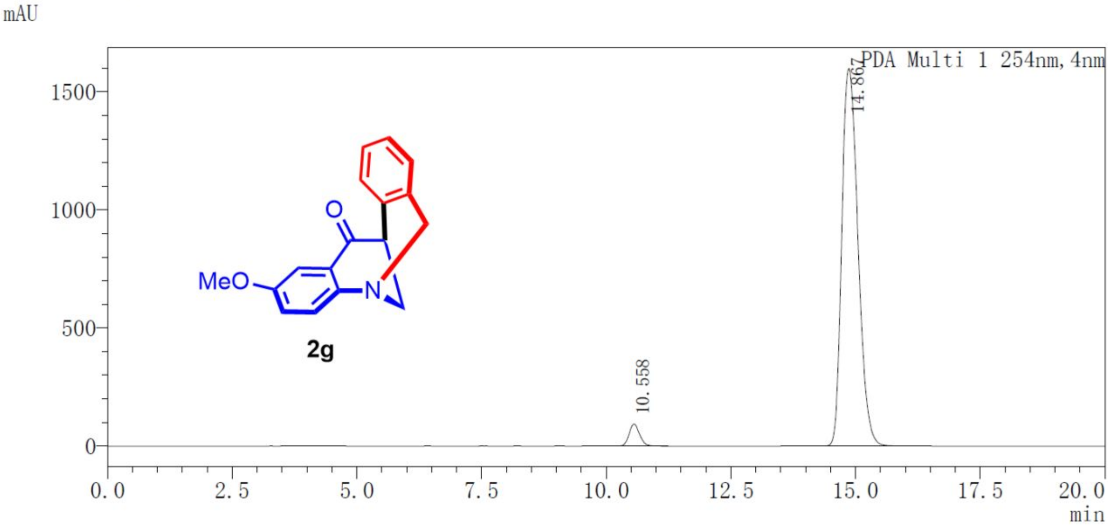

<Peak Table>

PDA Ch1 254nm

No.	Ret. Time (min)	Height (uAU)	Height%	Area (uAU*min)	Area%
1	10.558	93130	5.507	1369905	3.659
2	14.867	1598059	94.493	36069710	96.341
Total		1691189	100.000	37439614	100.000

Racemic:

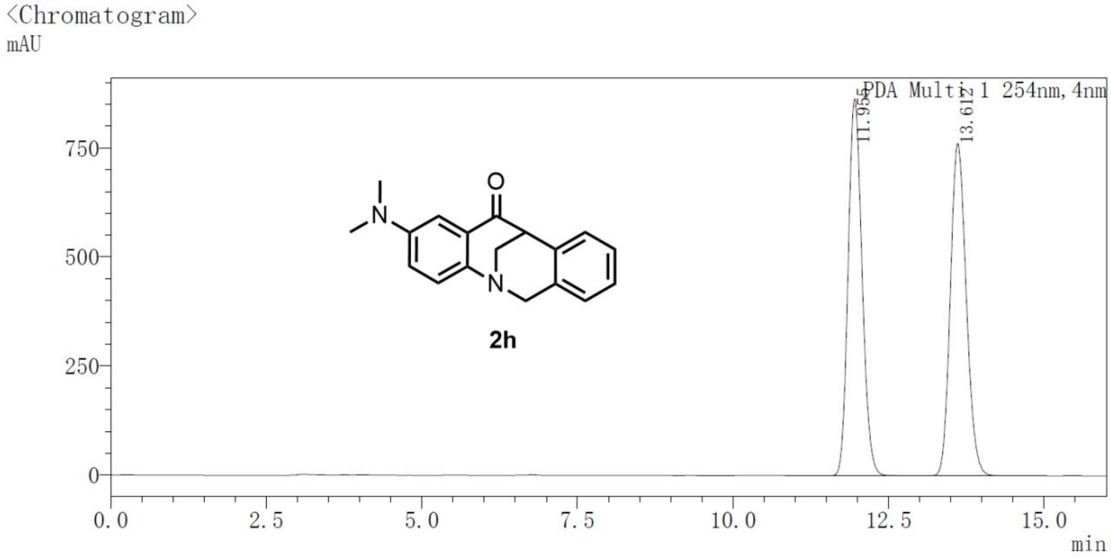

〈Peak Table〉

PDA Ch1 254nm

No.	Ret.Time(min)	Height(uAU)	Height%	Area(uAU*min)	Area%
1	11.955	863973	53.134	13500280	49.867
2	13.612	762064	46.866	13572208	50.133
Total		1626038	100.000	27072488	100.000

Enantioselective:

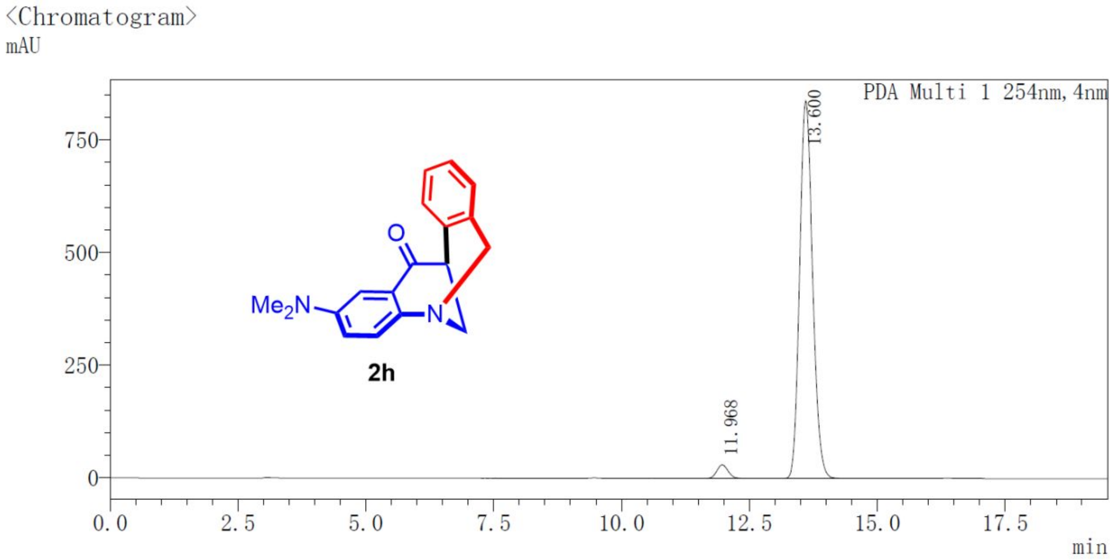

〈Peak Table〉

PDA Ch1 254nm

No.	Ret.Time(min)	Height(uAU)	Height%	Area(uAU*min)	Area%
1	11.968	30322	3.493	457773	3.009
2	13.600	837691	96.507	14754912	96.991
Total		868013	100.000	15212685	100.000

Racemic:

〈Chromatogram〉

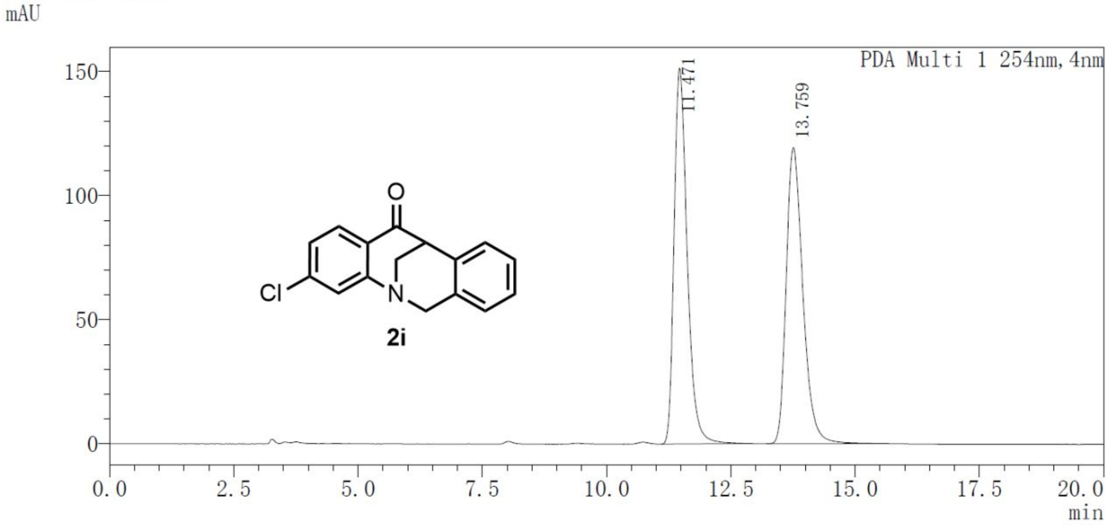

〈Peak Table〉

PDA Ch1 254nm

No.	Ret. Time (min)	Height (uAU)	Height%	Area (uAU*min)	Area%
1	11.471	151362	55.922	2778876	50.004
2	13.759	119306	44.078	2778458	49.996
Total		270668	100.000	5557334	100.000

Enantioselective:

〈Chromatogram〉

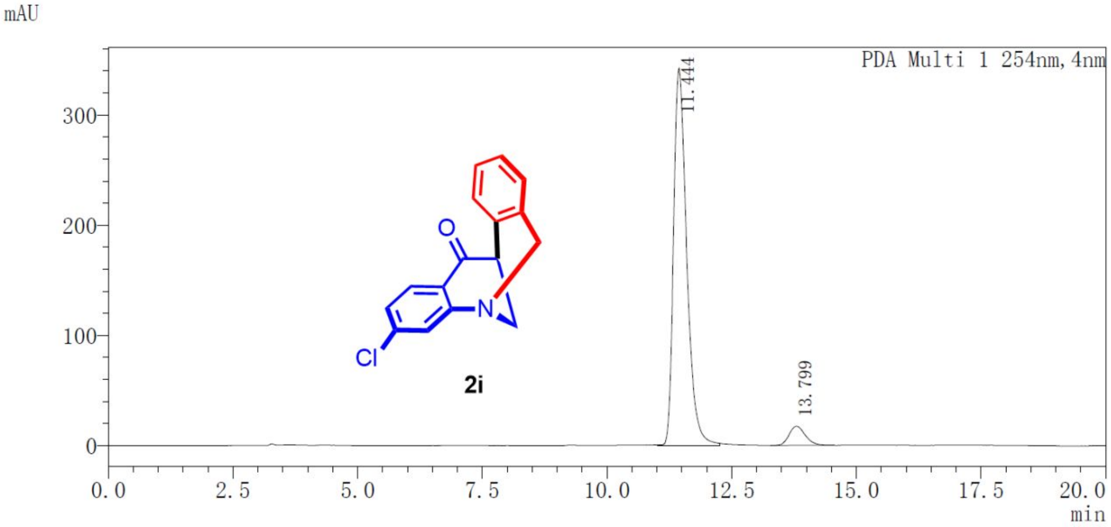

〈Peak Table〉

PDA Ch1 254nm

No.	Ret. Time (min)	Height (uAU)	Height%	Area (uAU*min)	Area%
1	11.444	341930	95.154	6262636	94.099
2	13.799	17412	4.846	392711	5.901
Total		359343	100.000	6655347	100.000

Racemic:

<Chromatogram>

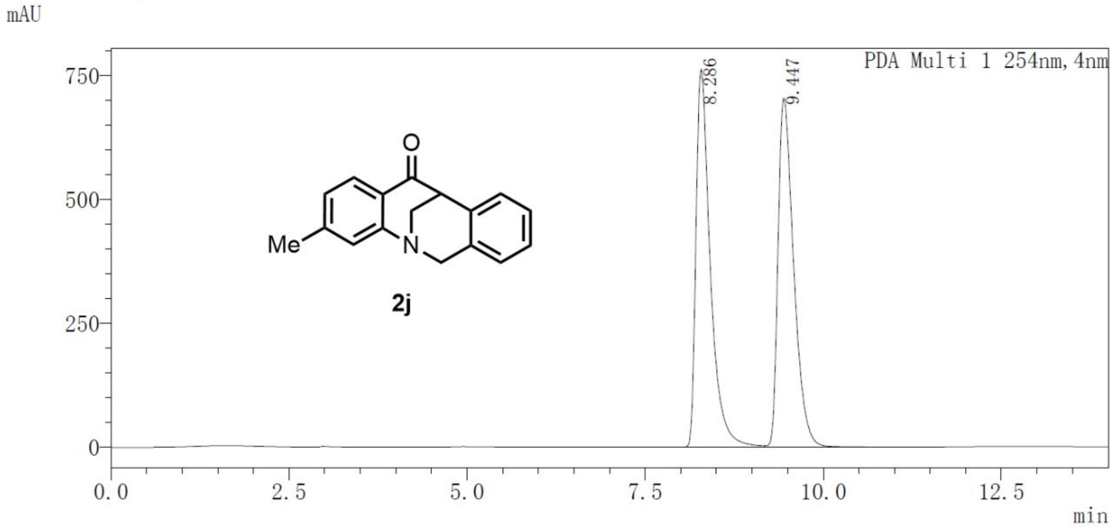

<Peak Table>

PDA Ch1 254nm

No.	Ret. Time (min)	Height (uAU)	Height%	Area (uAU*min)	Area%
1	8.286	761451	51.975	10662060	49.739
2	9.447	703575	48.025	10774073	50.261
Total		1465026	100.000	21436133	100.000

Enantioselective:

<Chromatogram>

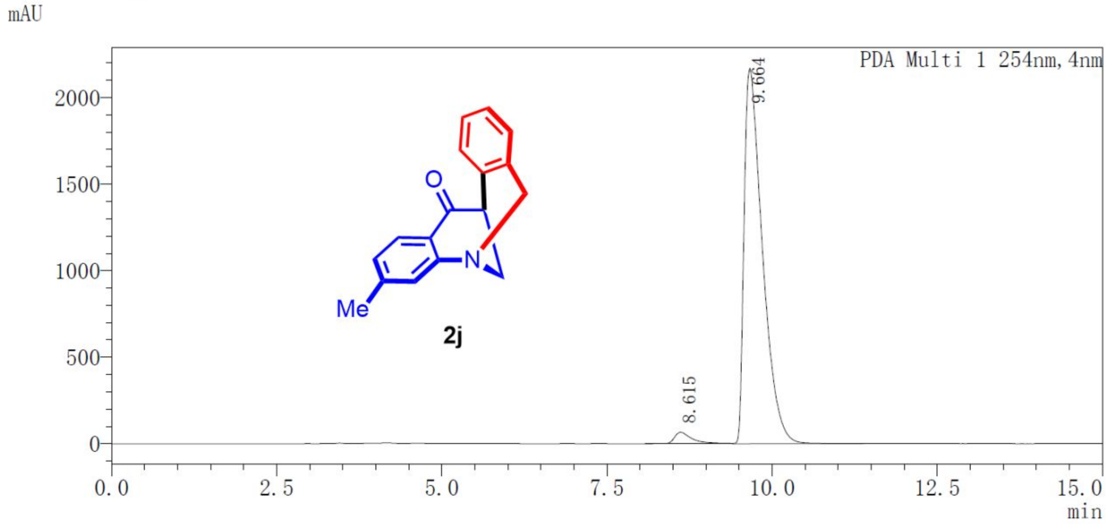

<Peak Table>

PDA Ch1 254nm

No.	Ret. Time (min)	Height (uAU)	Height%	Area (uAU*min)	Area%
1	8.615	65819	2.946	1209255	2.807
2	9.664	2168013	97.054	41873458	97.193
Total		2233832	100.000	43082713	100.000

Racemic:

<Chromatogram>
mAU

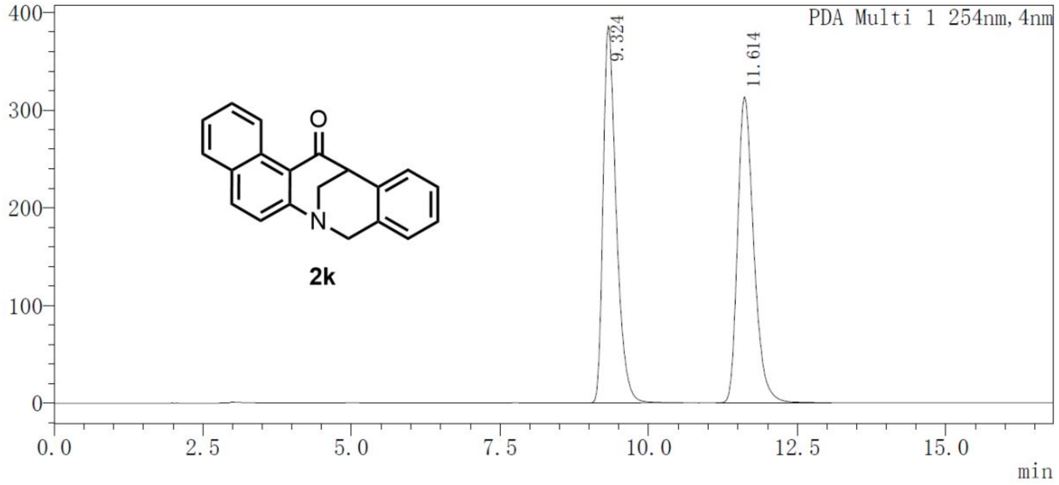

<Peak Table>

PDA Ch1 254nm

No.	Ret. Time(min)	Height(uAU)	Height%	Area(uAU*min)	Area%
1	9.324	385668	55.188	5942768	50.024
2	11.614	313157	44.812	5937102	49.976
Total		698825	100.000	11879871	100.000

Enantioselective:

<Chromatogram>
mAU

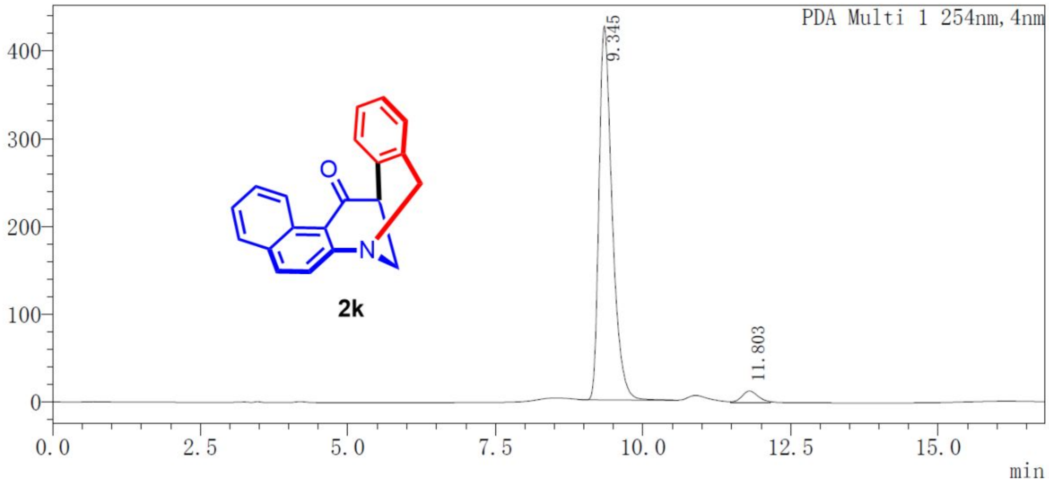

<Peak Table>

PDA Ch1 254nm

No.	Ret. Time(min)	Height(uAU)	Height%	Area(uAU*min)	Area%
1	9.345	425614	96.937	6613317	96.164
2	11.803	13449	3.063	263815	3.836
Total		439063	100.000	6877132	100.000

Racemic:

<Chromatogram>

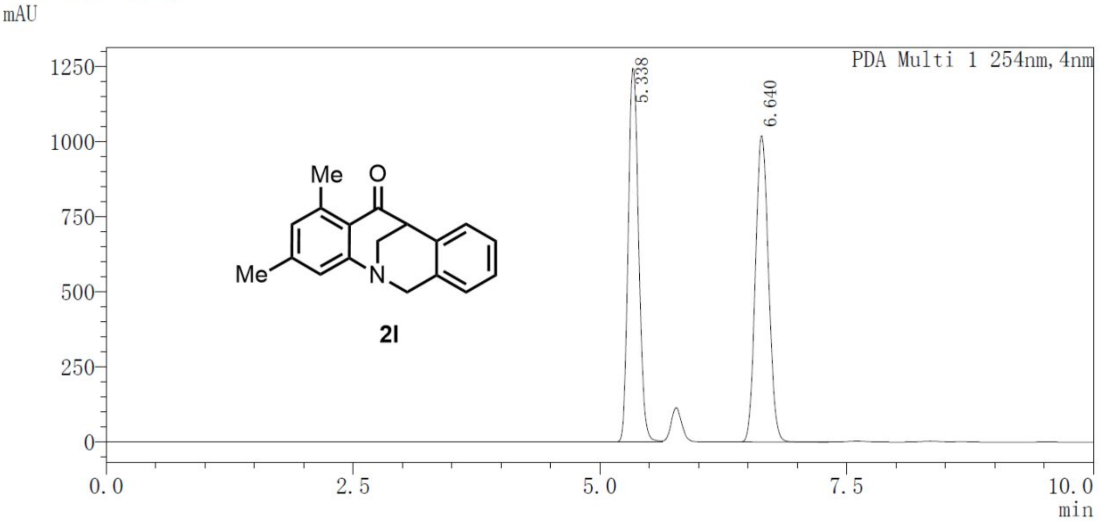

<Peak Table>

PDA Ch1 254nm

No.	Ret.Time(min)	Height(uAU)	Height%	Area(uAU*min)	Area%
1	5.338	1243913	54.968	9172922	49.298
2	6.640	1019063	45.032	9434300	50.702
Total		2262976	100.000	18607222	100.000

Enantioselective:

<Chromatogram>

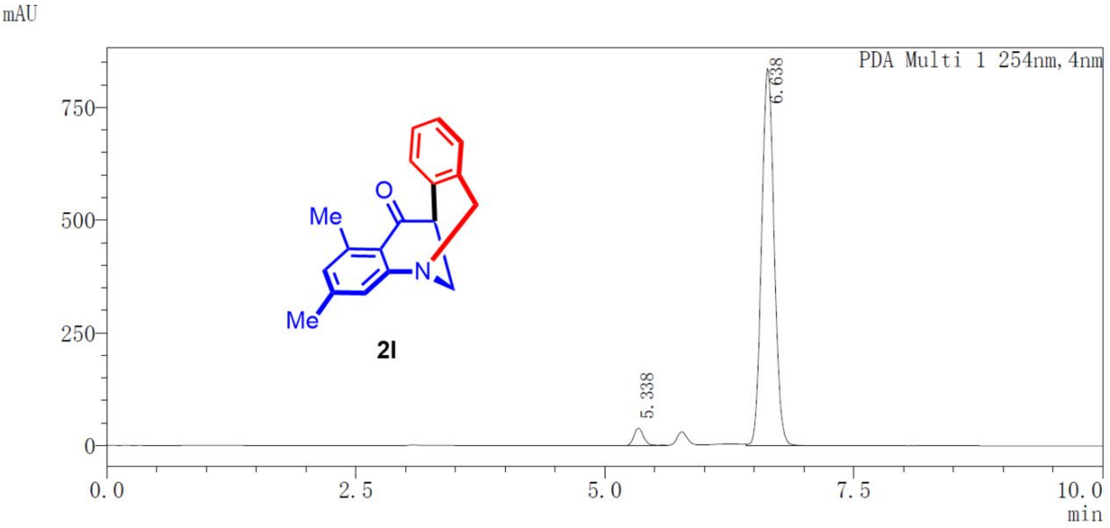

<Peak Table>

PDA Ch1 254nm

No.	Ret.Time(min)	Height(uAU)	Height%	Area(uAU*min)	Area%
1	5.338	38644	4.417	272444	3.587
2	6.638	836329	95.583	7323923	96.413
Total		874973	100.000	7596366	100.000

Racemic:

<Chromatogram>

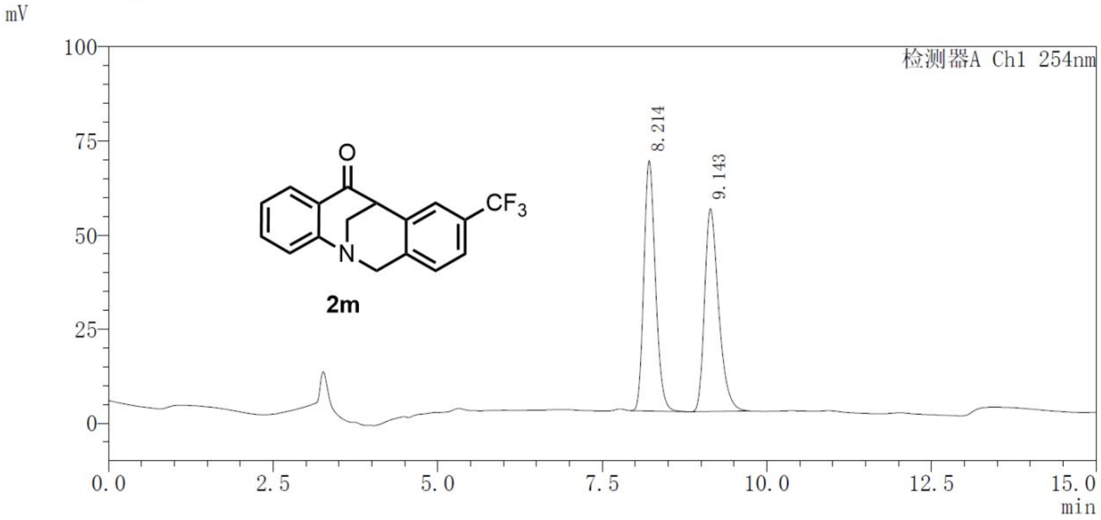

<Peak Table>

检测器A Ch1 254nm

No.	Ret. Time (min)	Area (uAU*min)	Height (uAU)	Height%	Area%
1	8. 214	817129	66547	55. 229	50. 460
2	9. 143	802237	53947	44. 771	49. 540
Total		1619367	120494	100. 000	100. 000

Enantioselective:

<Chromatogram>

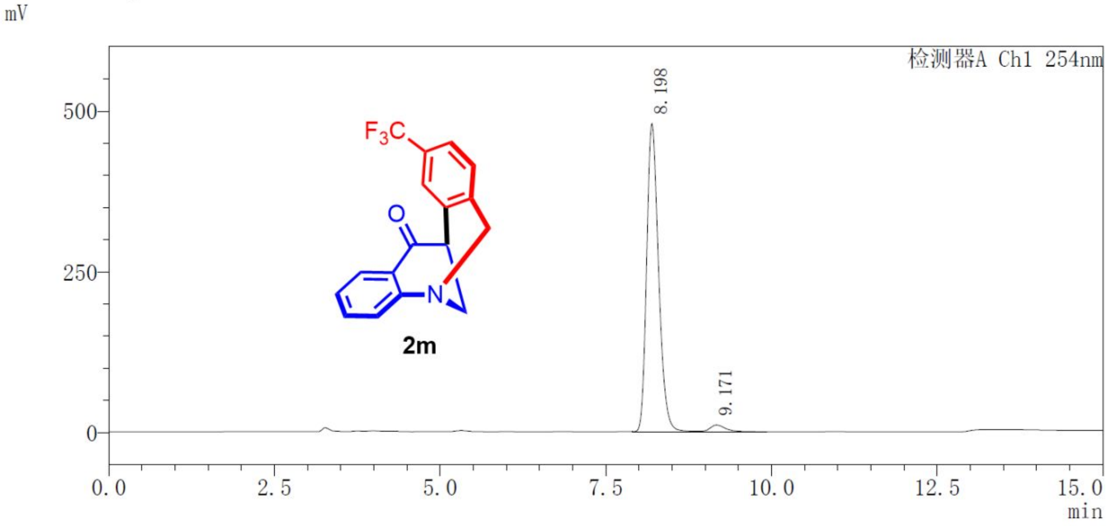

<Peak Table>

检测器A Ch1 254nm

No.	Ret. Time (min)	Area (uAU*min)	Height (uAU)	Height%	Area%
1	8. 198	6007211	479205	97. 866	97. 238
2	9. 171	170616	10451	2. 134	2. 762
Total		6177826	489656	100. 000	100. 000

Racemic:

<Chromatogram>

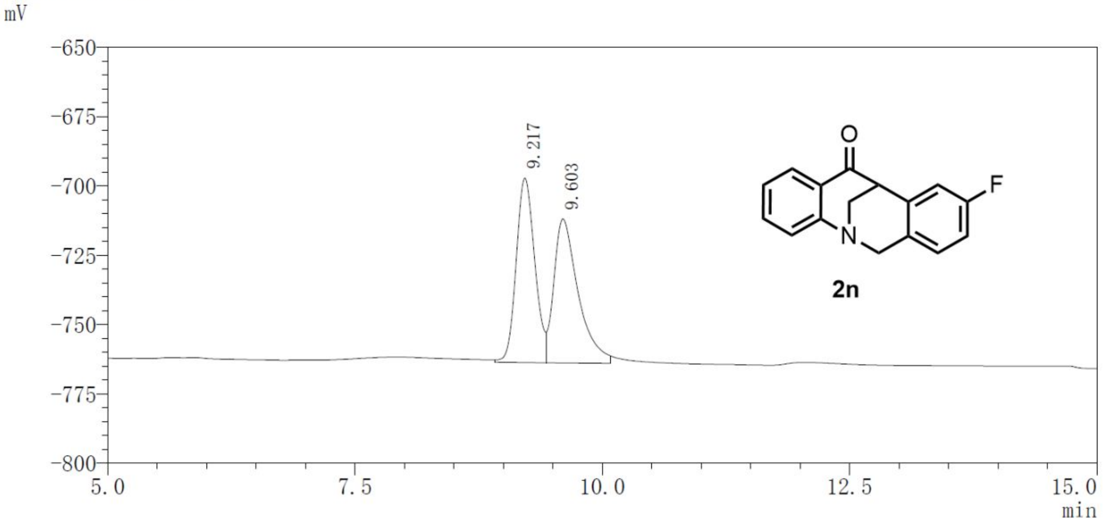

<Peak Table>

检测器A Ch1 254nm

No.	Ret. Time (min)	Area (uAU*min)	Height (uAU)	Height%	Area%
1	9.217	871857	66492	56.190	49.634
2	9.603	884724	51842	43.810	50.366
Total		1756581	118334	100.000	100.000

Enantioselective:

<Chromatogram>

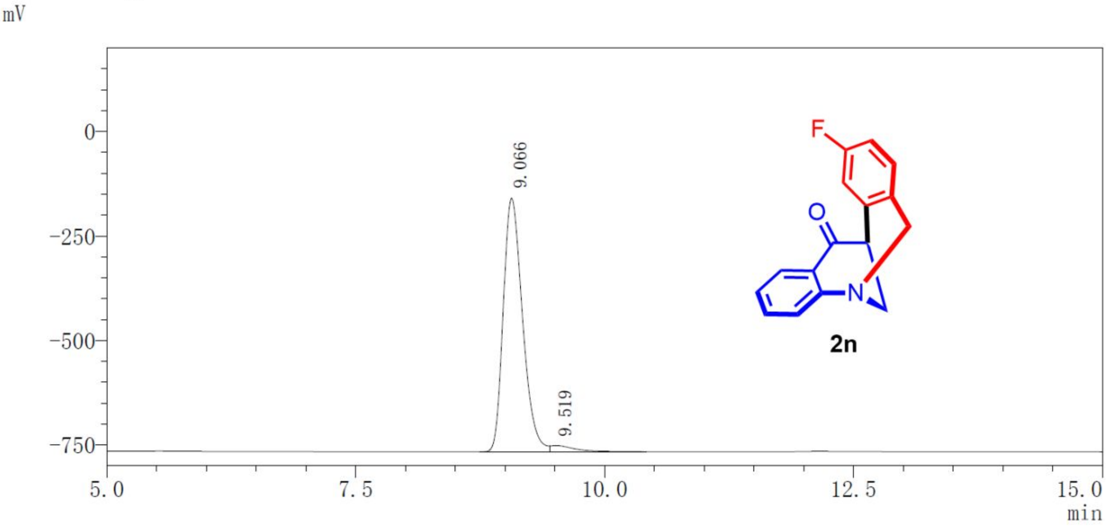

<Peak Table>

检测器A Ch1 254nm

No.	Ret. Time (min)	Area (uAU*min)	Height (uAU)	Height%	Area%
1	9.066	8104857	606523	97.645	97.103
2	9.519	241824	14630	2.355	2.897
Total		8346681	621153	100.000	100.000

Racemic:

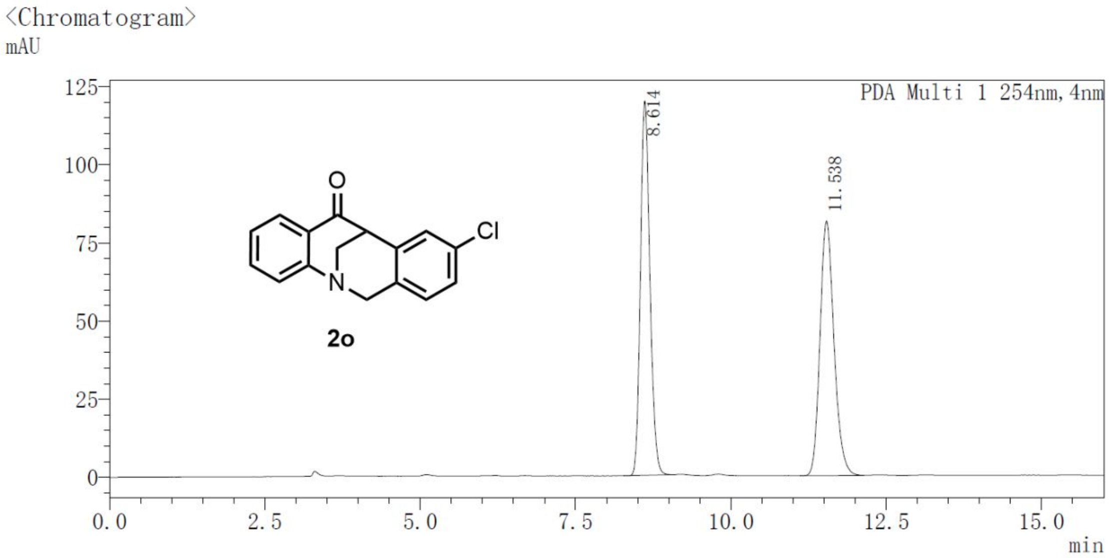

<Peak Table>

PDA Ch1 254nm

No.	Ret. Time (min)	Height (uAU)	Height%	Area (uAU*min)	Area%
1	8.614	119729	59.492	1272538	49.922
2	11.538	81523	40.508	1276540	50.078
Total		201252	100.000	2549077	100.000

Enantioselective:

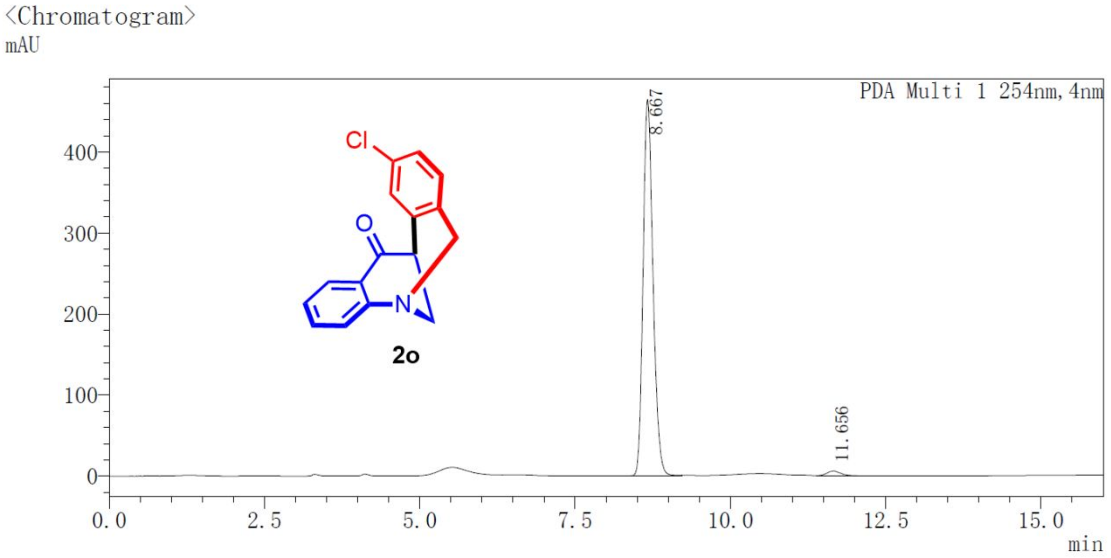

<Peak Table>

PDA Ch1 254nm

No.	Ret. Time (min)	Height (uAU)	Height%	Area (uAU*min)	Area%
1	8.667	464121	98.723	5169821	98.173
2	11.656	6004	1.277	96214	1.827
Total		470126	100.000	5266035	100.000

Racemic:

〈Chromatogram〉

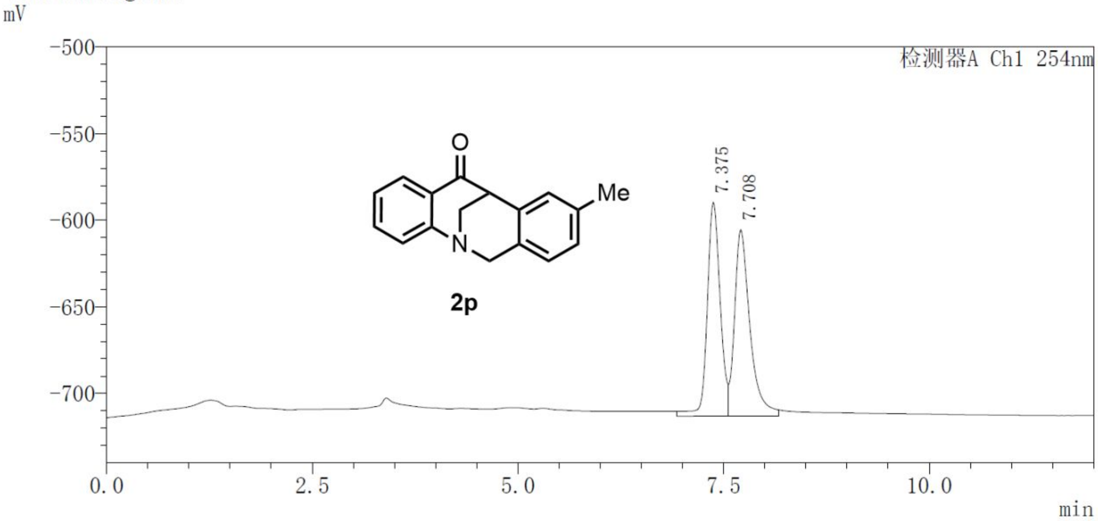

〈Peak Table〉

检测器A Ch1 254nm

No.	Ret. Time (min)	Area (uAU*min)	Height (uAU)	Height%	Area%
1	7.375	1361682	123354	53.428	49.367
2	7.708	1396623	107523	46.572	50.633
Total		2758306	230877	100.000	100.000

Enantioselective:

〈Chromatogram〉

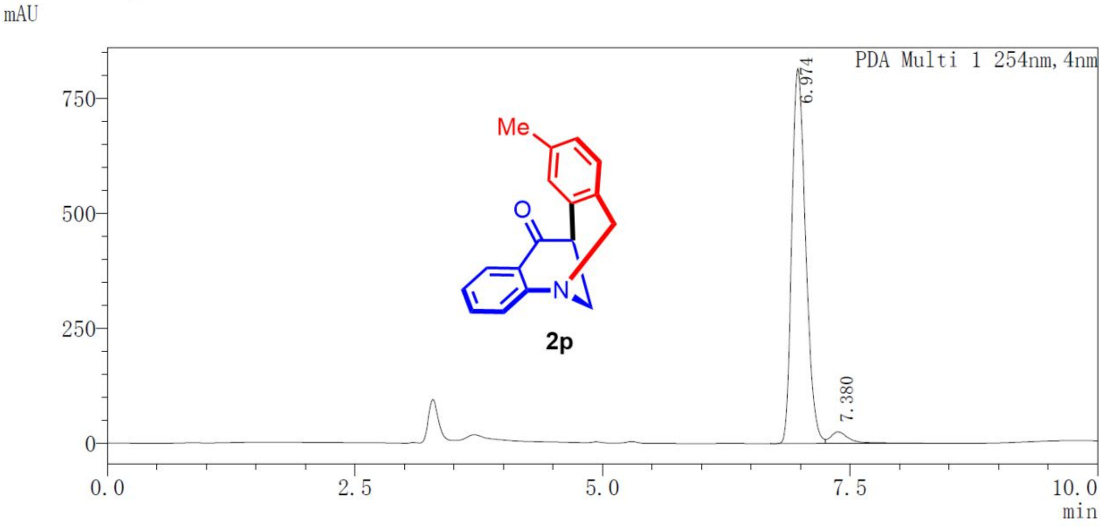

〈Peak Table〉

PDA Ch1 254nm

No.	Ret. Time (min)	Height (uAU)	Height%	Area (uAU*min)	Area%
1	6.974	816427	96.992	7937994	95.873
2	7.380	25319	3.008	341706	4.127
Total		841746	100.000	8279701	100.000

Racemic:

〈Chromatogram〉

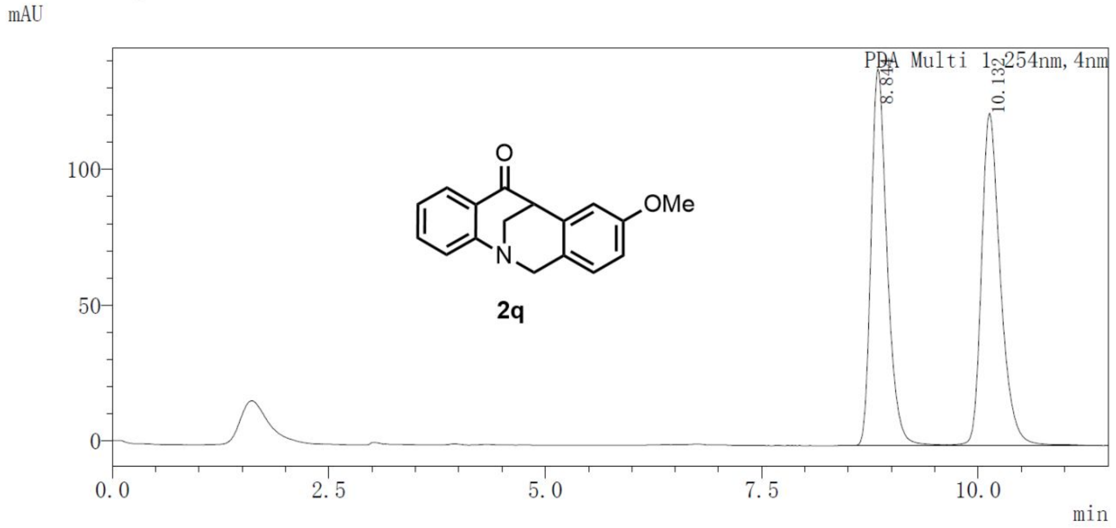

〈Peak Table〉

PDA Ch1 254nm

No.	Ret. Time (min)	Height (uAU)	Height%	Area (uAU*min)	Area%
1	8.844	138944	53.126	1786818	48.832
2	10.132	122593	46.874	1872307	51.168
Total		261538	100.000	3659125	100.000

Enantioselective:

〈Chromatogram〉

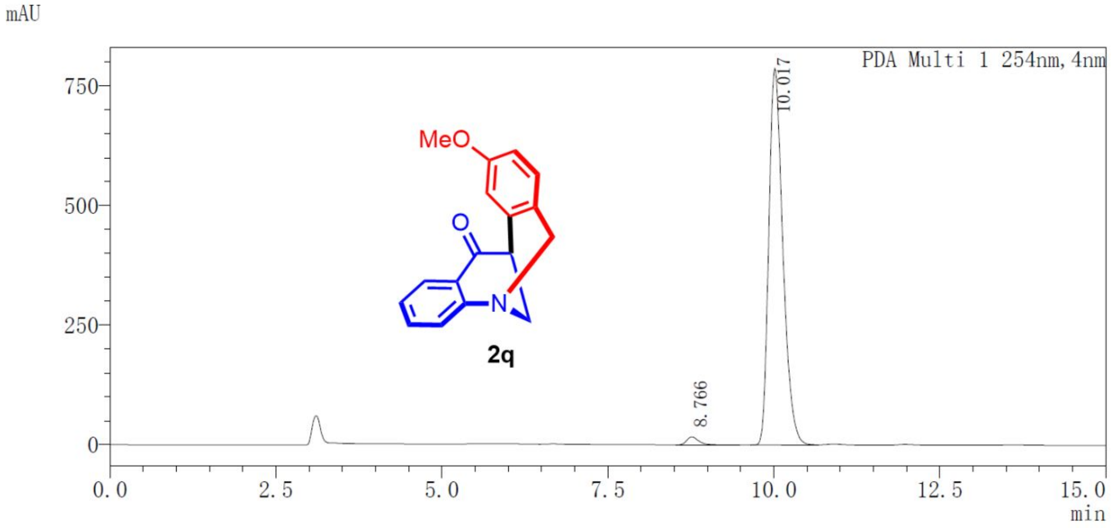

〈Peak Table〉

PDA Ch1 254nm

No.	Ret. Time (min)	Height (uAU)	Height%	Area (uAU*min)	Area%
1	8.766	16830	2.093	214117	1.804
2	10.017	787424	97.907	11651936	98.196
Total		804254	100.000	11866053	100.000

Racemic:

〈Chromatogram〉
mAU

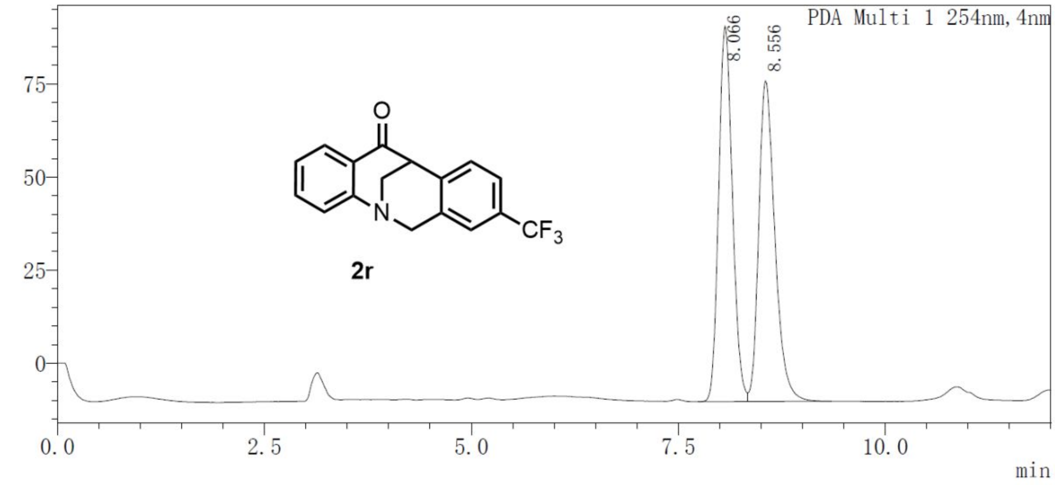

〈Peak Table〉

PDA Ch1 254nm

No.	Ret. Time (min)	Height (uAU)	Height%	Area (uAU*min)	Area%
1	8.066	100821	53.931	1170285	50.048
2	8.556	86122	46.069	1168037	49.952
Total		186943	100.000	2338322	100.000

Enantioselective:

〈Chromatogram〉
mAU

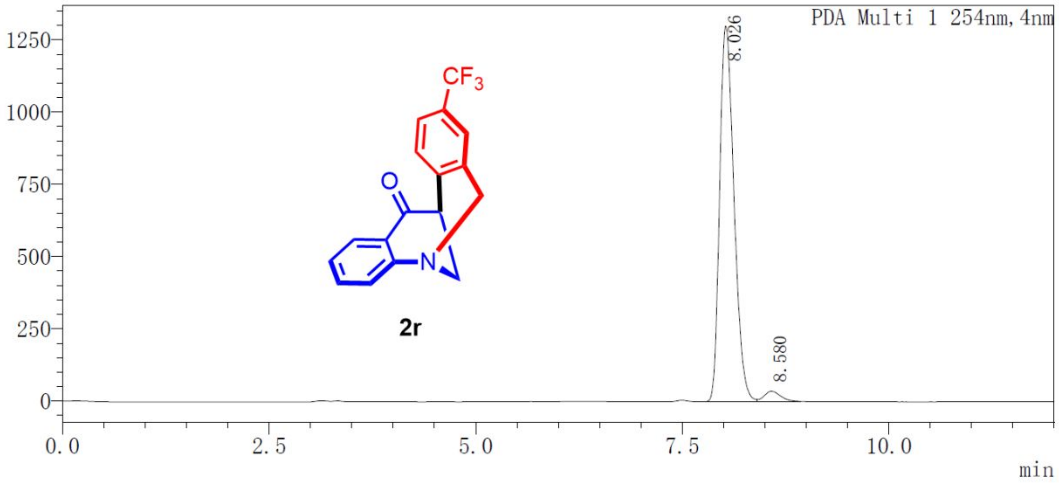

〈Peak Table〉

PDA Ch1 254nm

No.	Ret. Time (min)	Height (uAU)	Height%	Area (uAU*min)	Area%
1	8.026	1299092	97.259	15745109	96.806
2	8.580	36612	2.741	519480	3.194
Total		1335704	100.000	16264588	100.000

Racemic:

<Chromatogram>

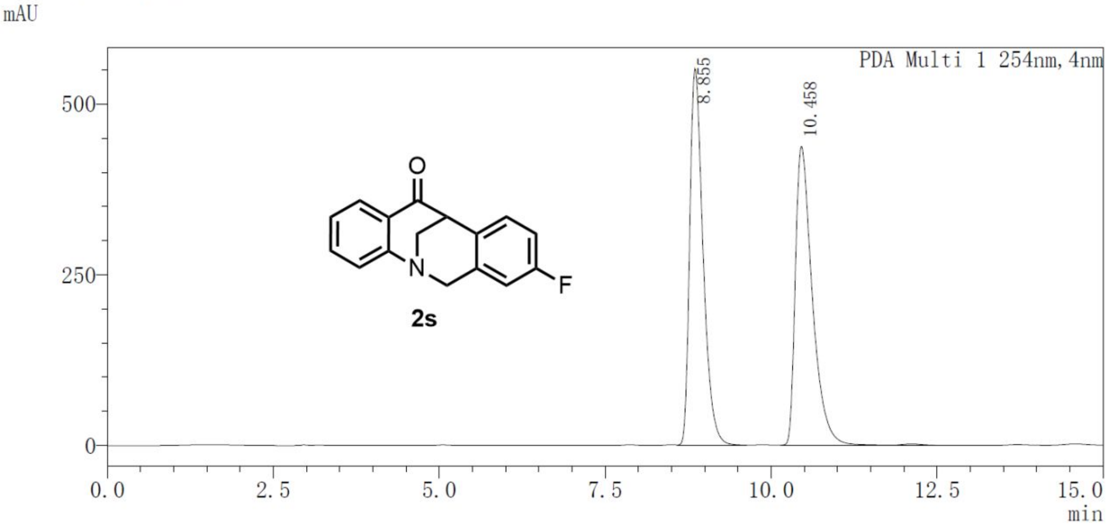

<Peak Table>

PDA Ch1 254nm

No.	Ret.Time(min)	Height(uAU)	Height%	Area(uAU*min)	Area%
1	8.855	551713	55.764	7730224	49.651
2	10.458	437662	44.236	7838748	50.349
Total		989375	100.000	15568972	100.000

Enantioselective:

<Chromatogram>

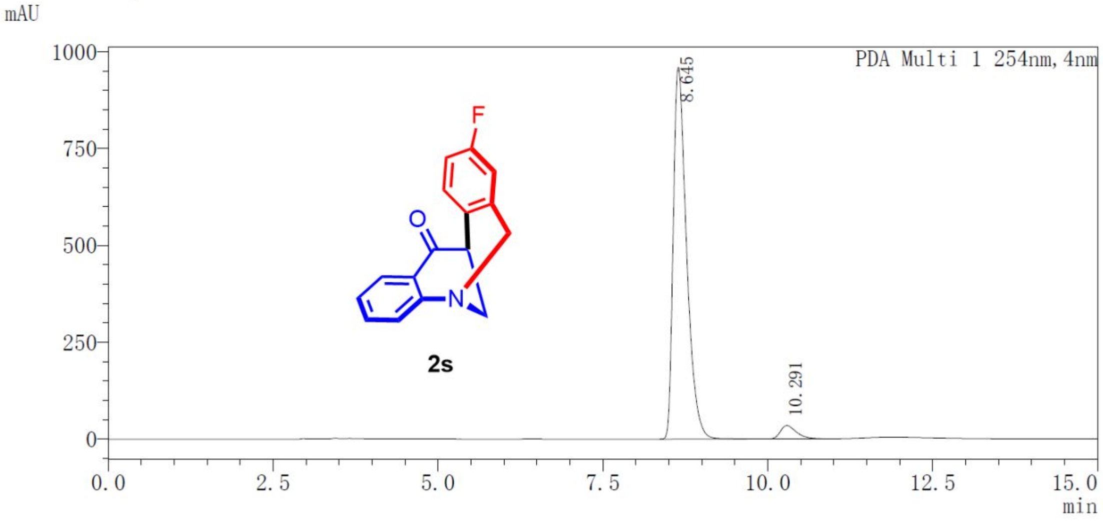

<Peak Table>

PDA Ch1 254nm

No.	Ret.Time(min)	Height(uAU)	Height%	Area(uAU*min)	Area%
1	8.645	958862	96.519	13472901	95.770
2	10.291	34583	3.481	595097	4.230
Total		993445	100.000	14067998	100.000

Racemic:

<Chromatogram>

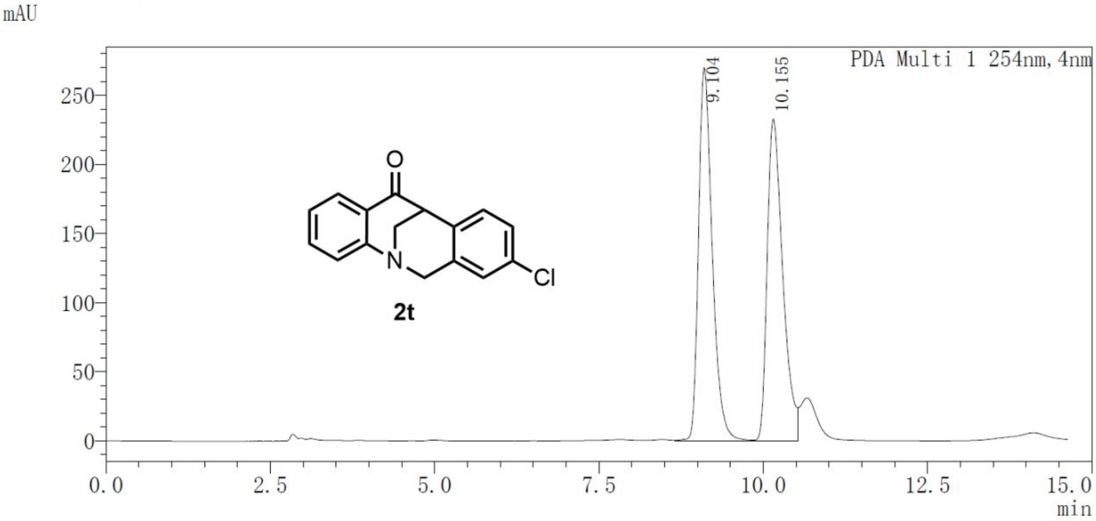

<Peak Table>

PDA Ch1 254nm

No.	Ret. Time (min)	Height (uAU)	Height%	Area (uAU*min)	Area%
1	9.104	269780	53.704	3928584	50.654
2	10.155	232565	46.296	3827075	49.346
Total		502346	100.000	7755659	100.000

Enantioselective:

<Chromatogram>

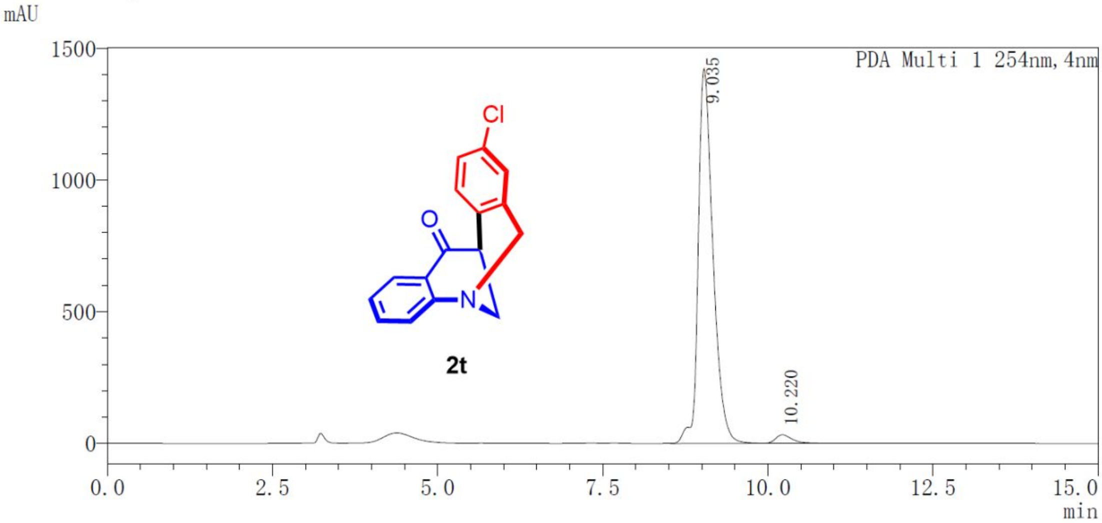

<Peak Table>

PDA Ch1 254nm

No.	Ret. Time (min)	Height (uAU)	Height%	Area (uAU*min)	Area%
1	9.035	1423667	97.756	22095249	97.471
2	10.220	32680	2.244	573379	2.529
Total		1456347	100.000	22668629	100.000

Racemic:

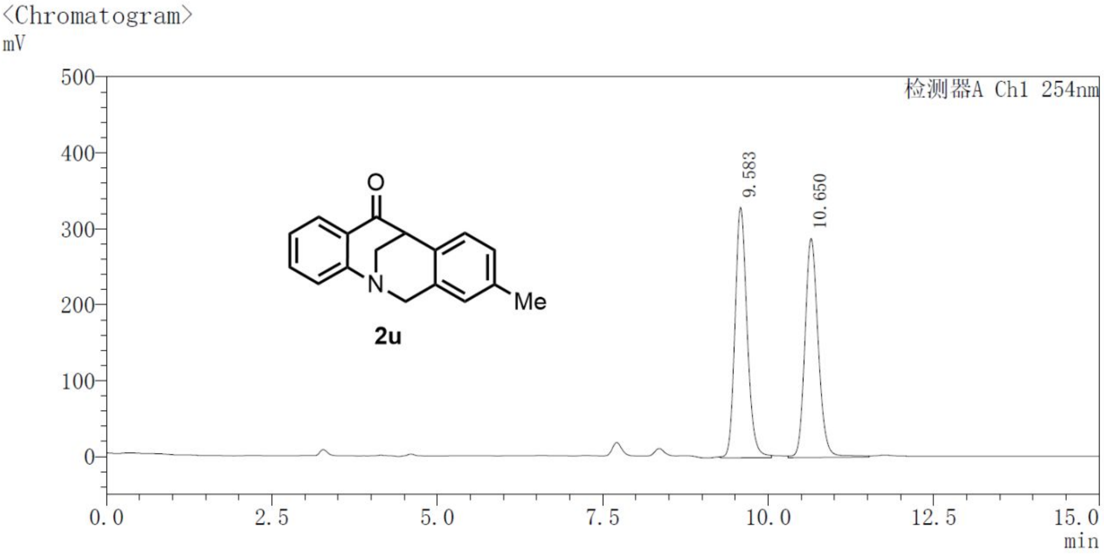

<Peak Table>

检测器A Ch1 254nm

No.	Ret. Time (min)	Area (uAU*min)	Height (uAU)	Height%	Area%
1	9.583	4208567	330034	53.357	50.931
2	10.650	4054639	288501	46.643	49.069
Total		8263206	618535	100.000	100.000

Enantioselective:

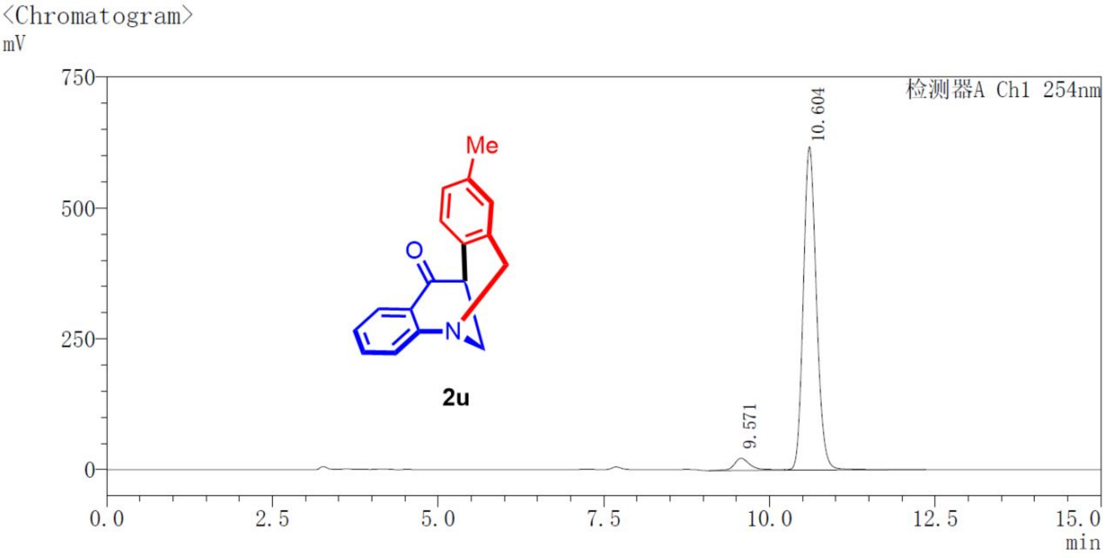

<Peak Table>

检测器A Ch1 254nm

No.	Ret. Time (min)	Area (uAU*min)	Height (uAU)	Height%	Area%
1	9.571	416367	23558	3.670	4.645
2	10.604	8546568	618308	96.330	95.355
Total		8962935	641866	100.000	100.000

Racemic:

<Chromatogram>

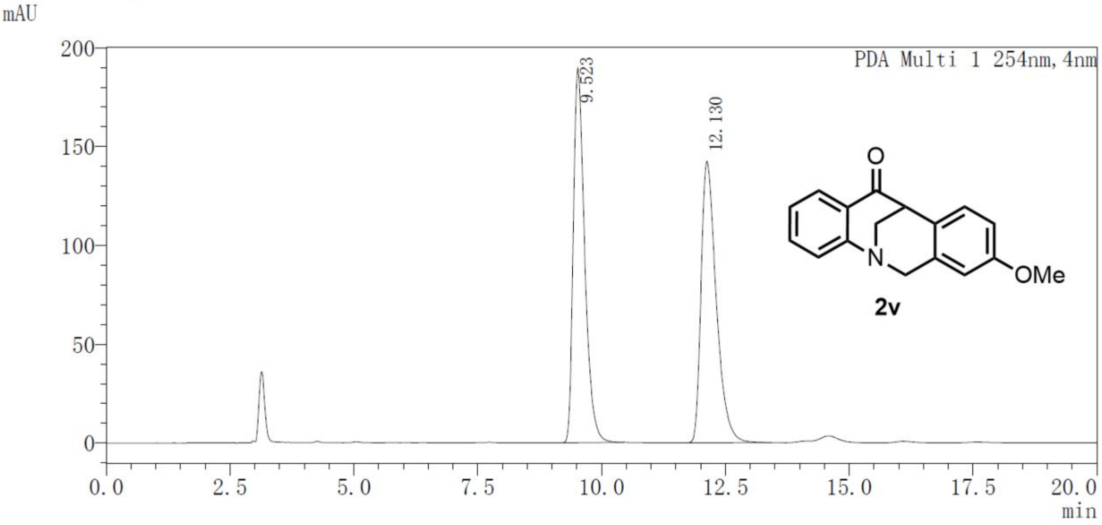

<Peak Table>

PDA Ch1 254nm

No.	Ret. Time (min)	Height (uAU)	Height%	Area (uAU*min)	Area%
1	9.523	189616	57.083	3037027	49.950
2	12.130	142562	42.917	3043145	50.050
Total		332179	100.000	6080172	100.000

Enantioselective:

<Chromatogram>

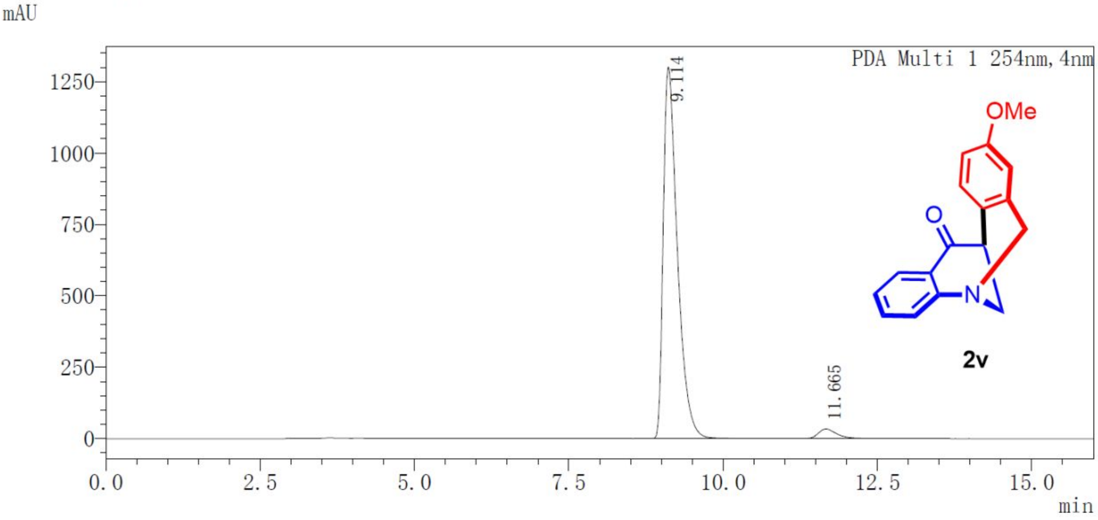

<Peak Table>

PDA Ch1 254nm

No.	Ret. Time (min)	Height (uAU)	Height%	Area (uAU*min)	Area%
1	9.114	1300354	97.500	20706703	96.815
2	11.665	33349	2.500	681280	3.185
Total		1333703	100.000	21387983	100.000

Racemic:

<Chromatogram>

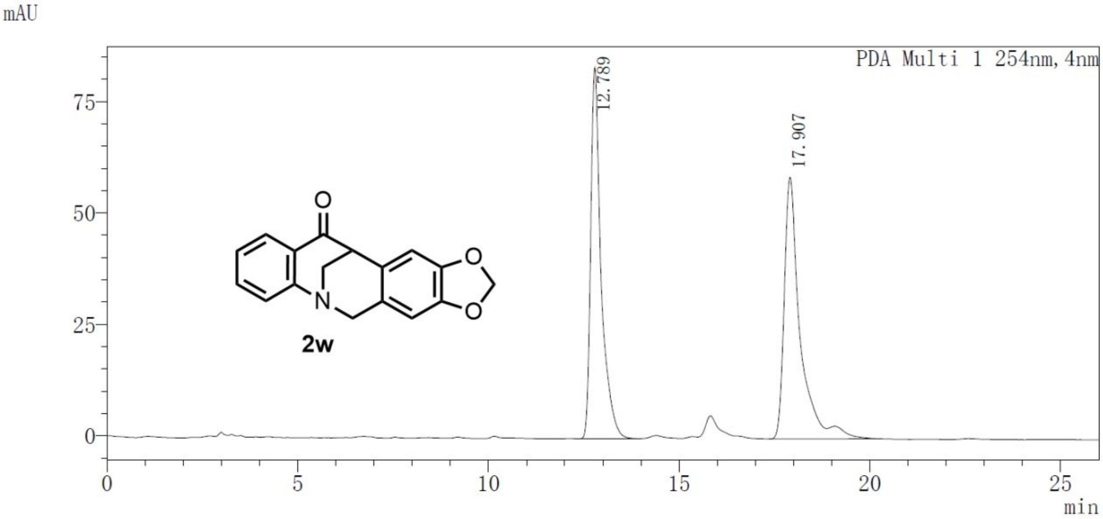

<Peak Table>

PDA Ch1 254nm

No.	Ret. Time (min)	Height (uAU)	Height%	Area (uAU*min)	Area%
1	12.789	83340	58.657	1580480	48.082
2	17.907	58741	41.343	1706561	51.918
Total		142081	100.000	3287041	100.000

Enantioselective:

<Chromatogram>

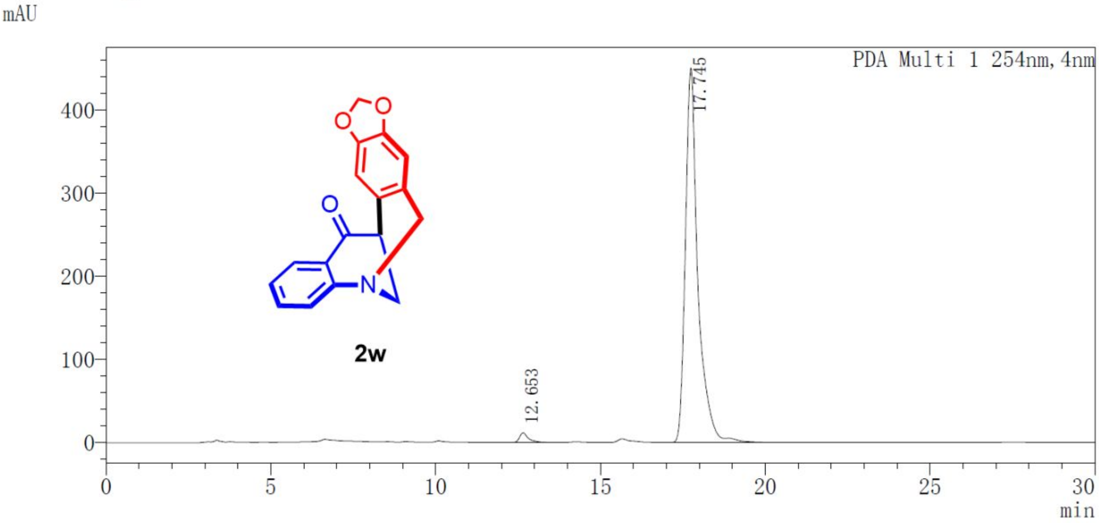

<Peak Table>

PDA Ch1 254nm

No.	Ret. Time (min)	Height (uAU)	Height%	Area (uAU*min)	Area%
1	12.653	11600	2.511	215022	1.775
2	17.745	450384	97.489	11897813	98.225
Total		461984	100.000	12112835	100.000

Racemic:

〈Chromatogram〉
mAU

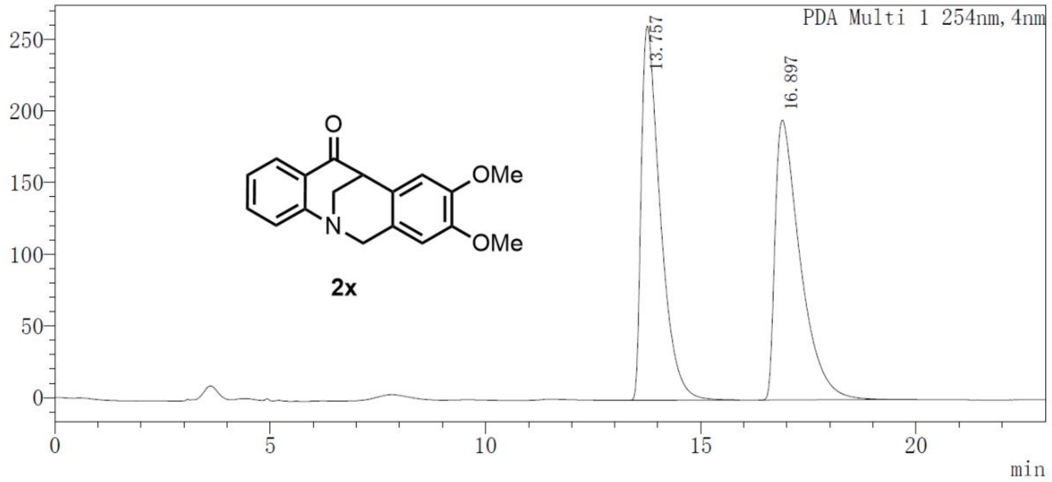

〈Peak Table〉

PDA Ch1 254nm

No.	Ret.Time(min)	Height(uAU)	Height%	Area(uAU*min)	Area%
1	13.757	261240	57.207	8033091	50.233
2	16.897	195416	42.793	7958595	49.767
Total		456655	100.000	15991686	100.000

Enantioselective:

〈Chromatogram〉
mAU

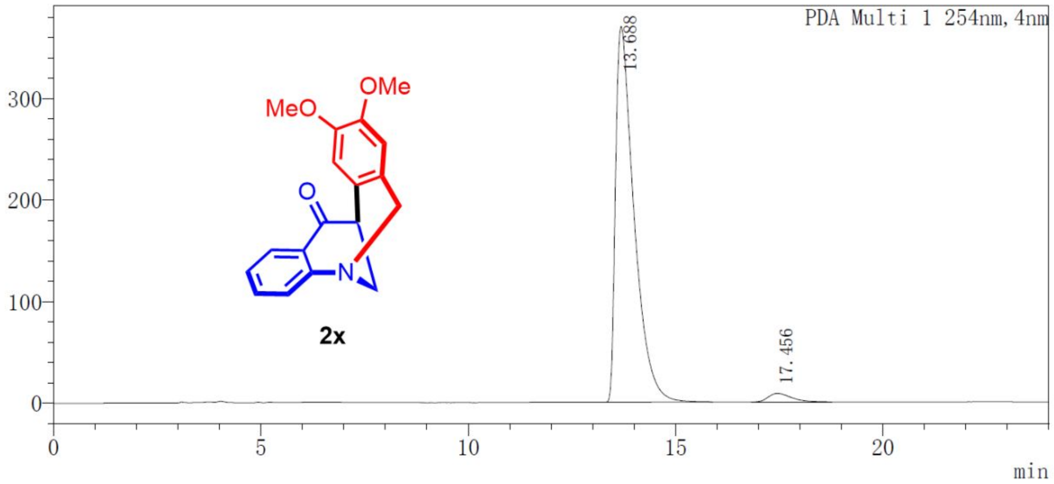

〈Peak Table〉

PDA Ch1 254nm

No.	Ret.Time(min)	Height(uAU)	Height%	Area(uAU*min)	Area%
1	13.688	369943	97.754	11482173	97.202
2	17.456	8499	2.246	330490	2.798
Total		378442	100.000	11812664	100.000

Racemic:

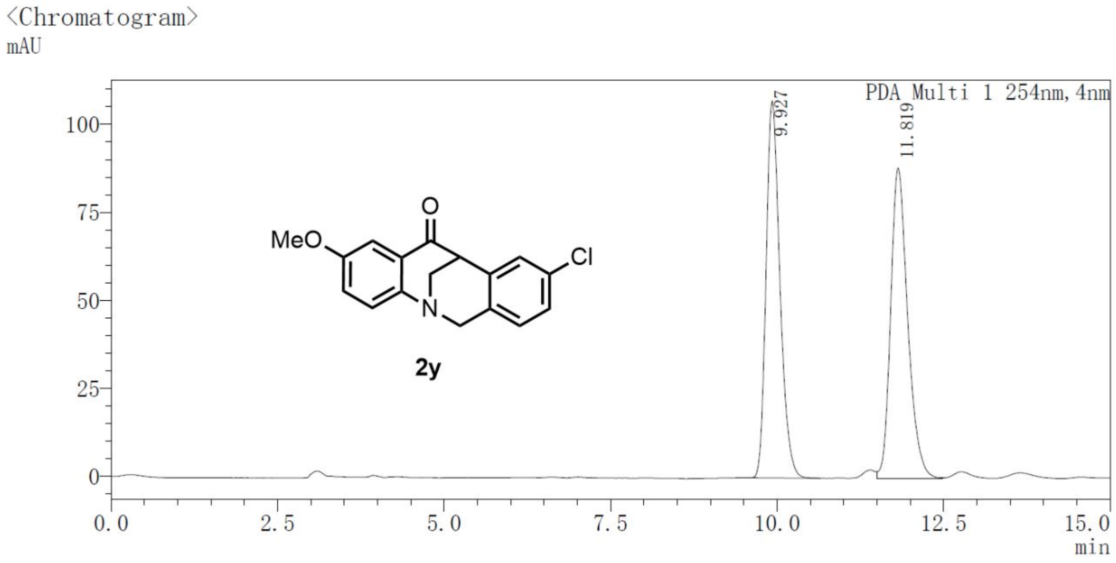

⟨Peak Table⟩

PDA Ch1 254nm

No.	Ret.Time(min)	Height(uAU)	Height%	Area(uAU*min)	Area%
1	9.927	107096	54.841	1553739	49.775
2	11.819	88189	45.159	1567755	50.225
Total		195285	100.000	3121494	100.000

Enantioselective:

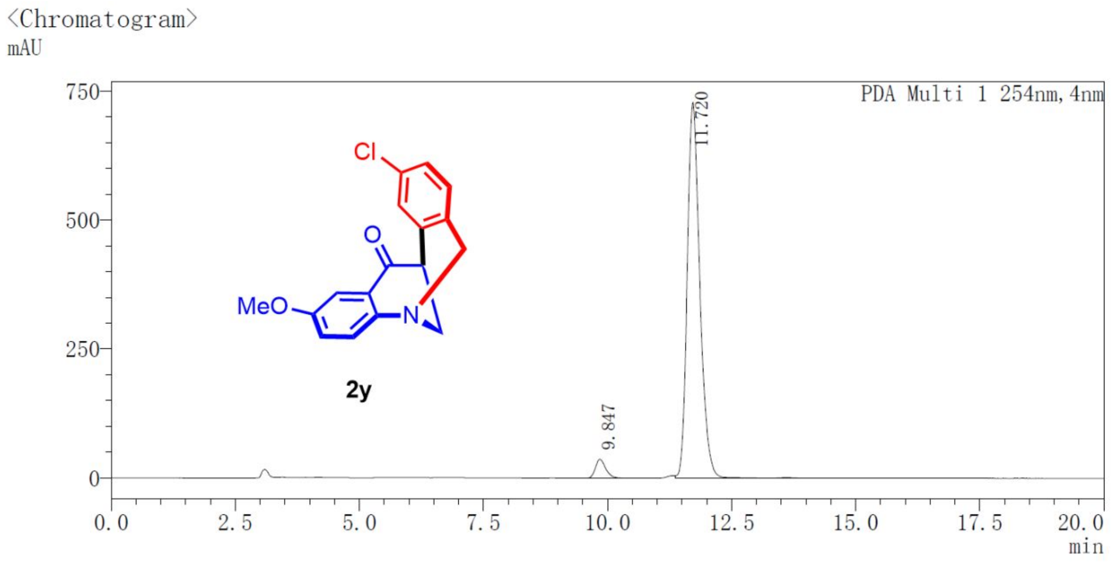

⟨Peak Table⟩

PDA Ch1 254nm

No.	Ret.Time(min)	Height(uAU)	Height%	Area(uAU*min)	Area%
1	9.847	36671	4.794	526839	3.935
2	11.720	728215	95.206	12861559	96.065
Total		764886	100.000	13388399	100.000

Racemic:

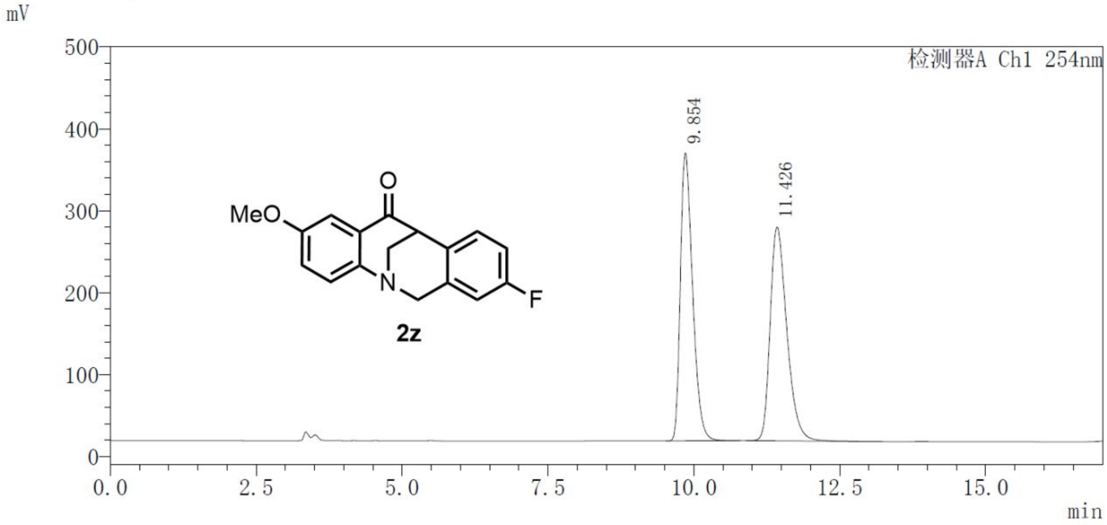

〈Peak Table〉

检测器A Ch1 254nm

No.	Ret.Time(min)	Area(uAU*min)	Height(uAU)	Height%	Area%
1	9.854	5122189	351390	57.365	49.692
2	11.426	5185689	261160	42.635	50.308
Total		10307878	612549	100.000	100.000

Enantioselective:

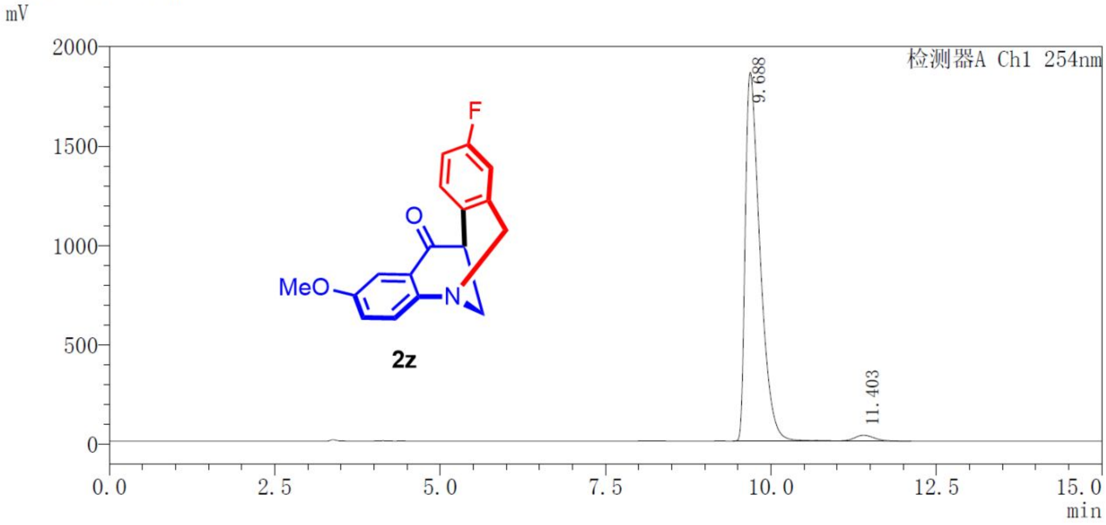

〈Peak Table〉

检测器A Ch1 254nm

No.	Ret.Time(min)	Area(uAU*min)	Height(uAU)	Height%	Area%
1	9.688	28865274	1855494	98.462	98.004
2	11.403	587804	28980	1.538	1.996
Total		29453078	1884474	100.000	100.000

Racemic:

<Chromatogram>

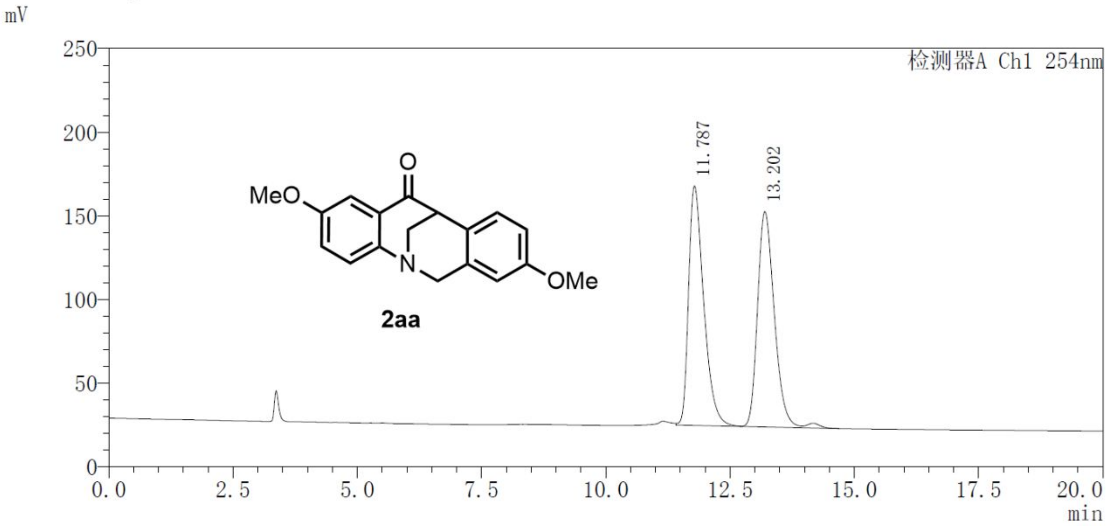

<Peak Table>

检测器A Ch1 254nm

No.	Ret. Time (min)	Area (uAU*min)	Height (uAU)	Height%	Area%
1	11.787	3023276	143176	52.662	49.749
2	13.202	3053781	128701	47.338	50.251
Total		6077058	271877	100.000	100.000

Enantioselective:

<Chromatogram>

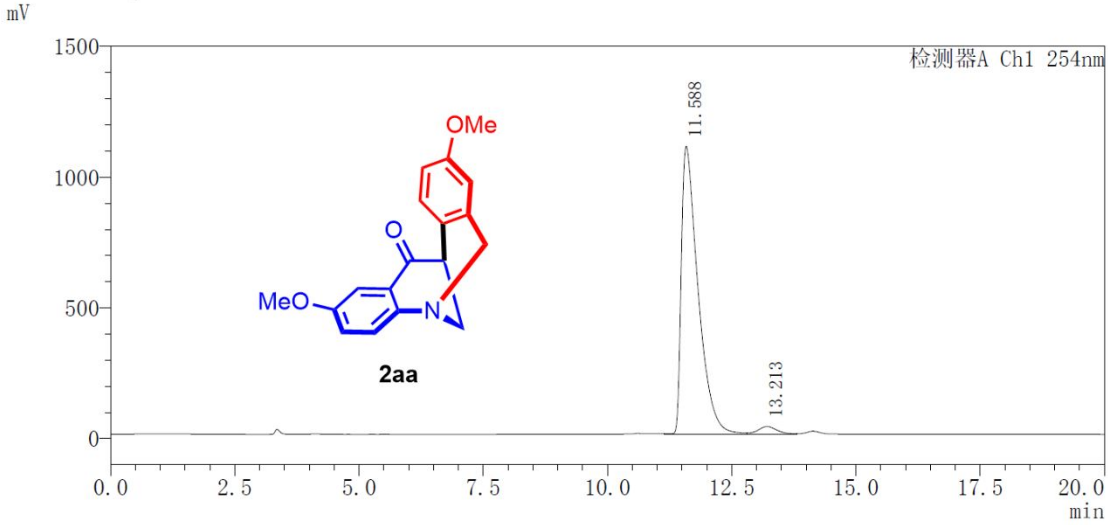

<Peak Table>

检测器A Ch1 254nm

No.	Ret. Time (min)	Area (uAU*min)	Height (uAU)	Height%	Area%
1	11.588	25888175	1102201	97.332	96.854
2	13.213	841004	30217	2.668	3.146
Total		26729179	1132418	100.000	100.000

Racemic:

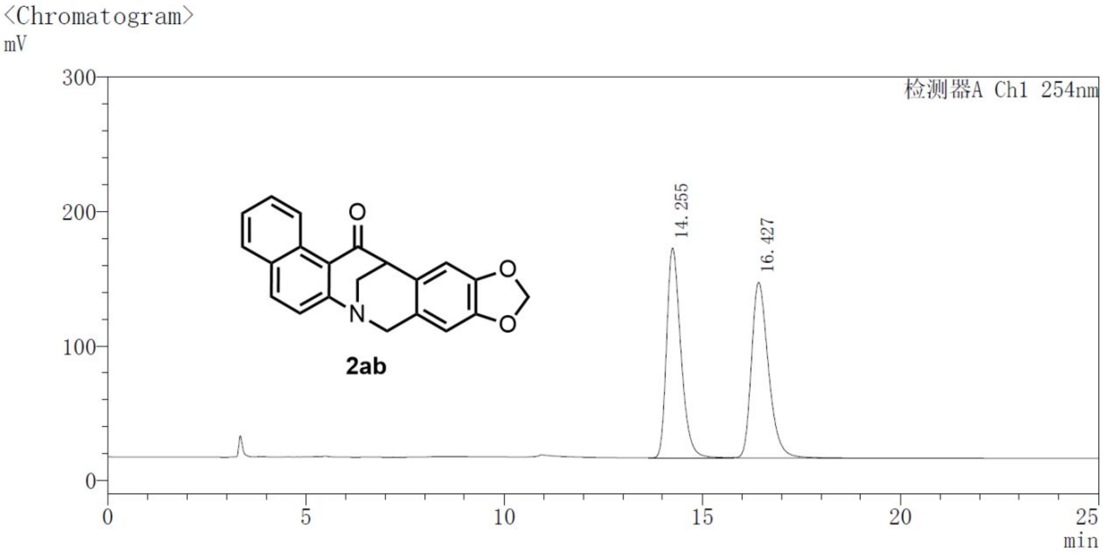

<Peak Table>

检测器A Ch1 254nm

No.	Ret. Time (min)	Area (uAU*min)	Height (uAU)	Height%	Area%
1	14.255	3905067	156132	54.435	50.005
2	16.427	3904268	130690	45.565	49.995
Total		7809335	286822	100.000	100.000

Enantioselective:

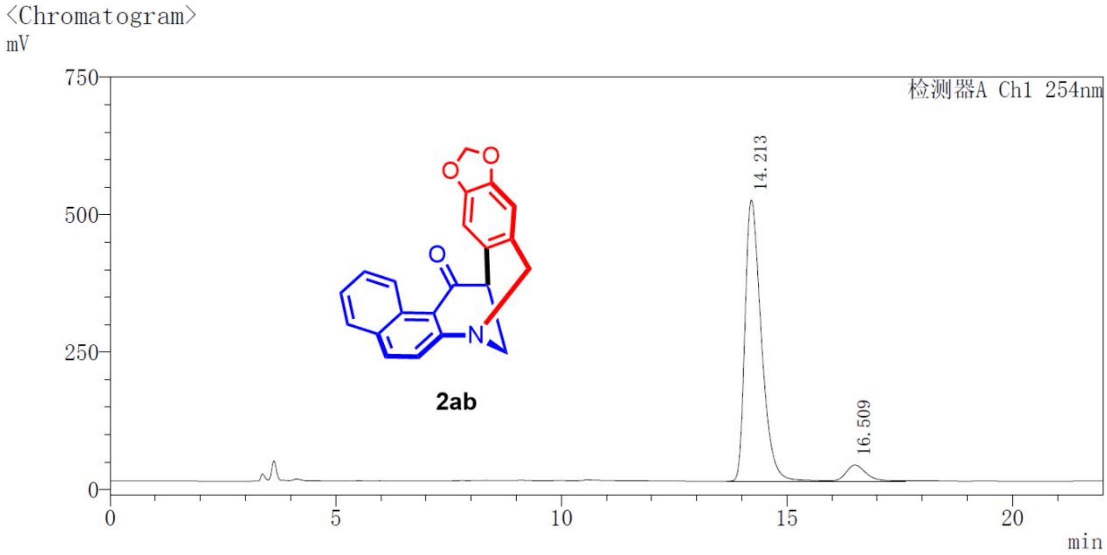

<Peak Table>

检测器A Ch1 254nm

No.	Ret. Time (min)	Area (uAU*min)	Height (uAU)	Height%	Area%
1	14.213	12883879	511290	94.565	93.284
2	16.509	927622	29383	5.435	6.716
Total		13811500	540673	100.000	100.000

Racemic:

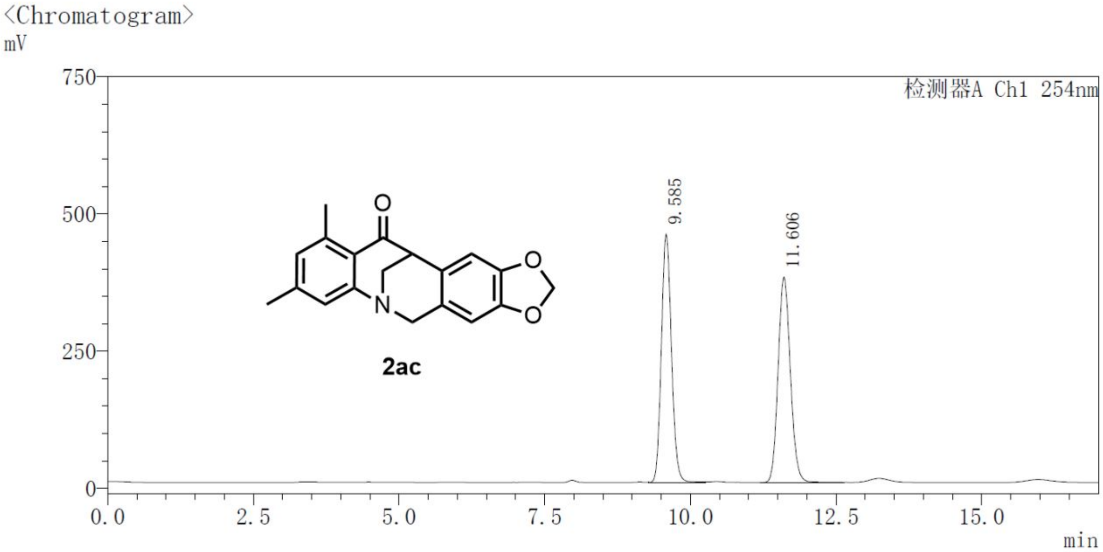

<Peak Table>

检测器A Ch1 254nm

No.	Ret. Time (min)	Area (uAU*min)	Height (uAU)	Height%	Area%
1	9.585	5455208	452461	54.736	49.865
2	11.606	5484835	374161	45.264	50.135
Total		10940043	826622	100.000	100.000

Enantioselective:

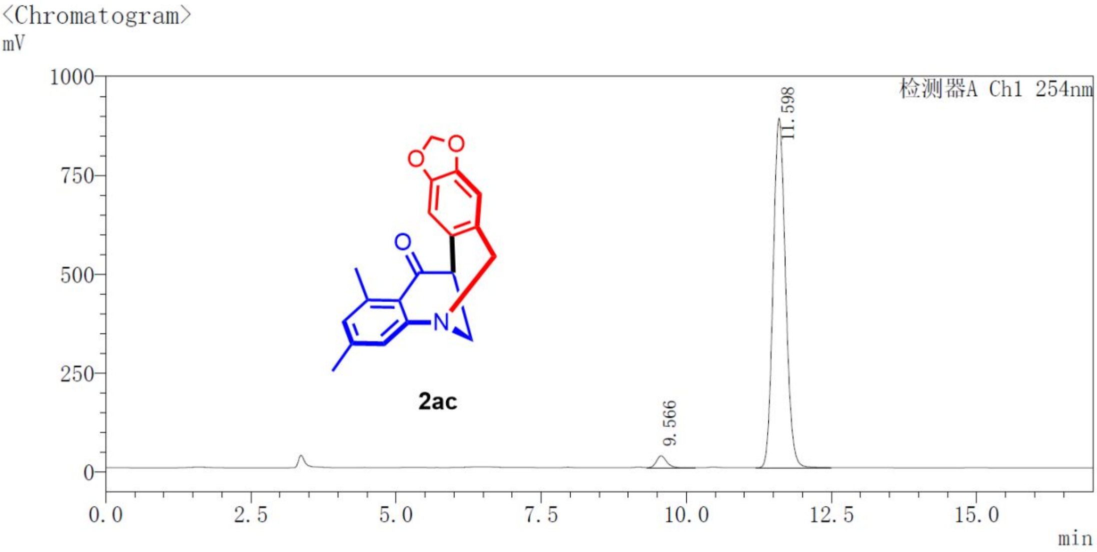

<Peak Table>

检测器A Ch1 254nm

No.	Ret. Time (min)	Area (uAU*min)	Height (uAU)	Height%	Area%
1	9.566	379802	30258	3.309	2.808
2	11.598	13147479	884157	96.691	97.192
Total		13527281	914416	100.000	100.000

Racemic:

<Chromatogram>

mAU

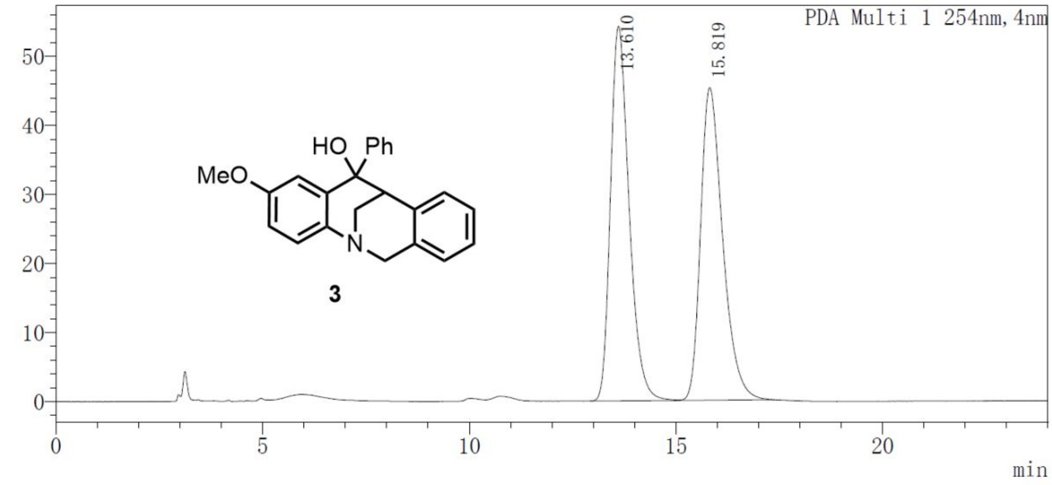

<Peak Table>

PDA Ch1 254nm

No.	Ret.Time(min)	Height(uAU)	Height%	Area(uAU*min)	Area%
1	13.610	54291	54.509	1700673	50.221
2	15.819	45310	45.491	1685710	49.779
Total		99601	100.000	3386383	100.000

Enantioselective:

<Chromatogram>

mAU

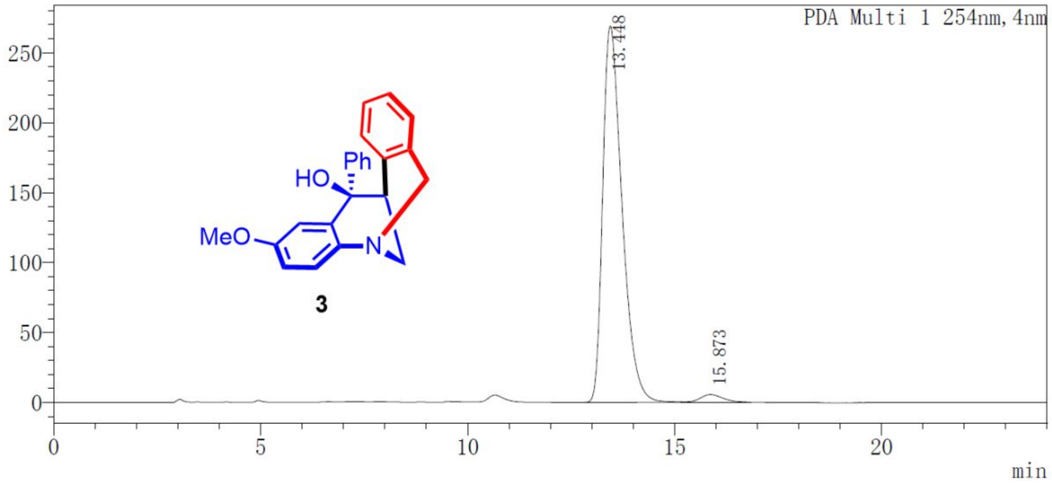

<Peak Table>

PDA Ch1 254nm

No.	Ret.Time(min)	Height(uAU)	Height%	Area(uAU*min)	Area%
1	13.448	268976	97.983	8725506	97.611
2	15.873	5536	2.017	213546	2.389
Total		274513	100.000	8939053	100.000

Racemic:

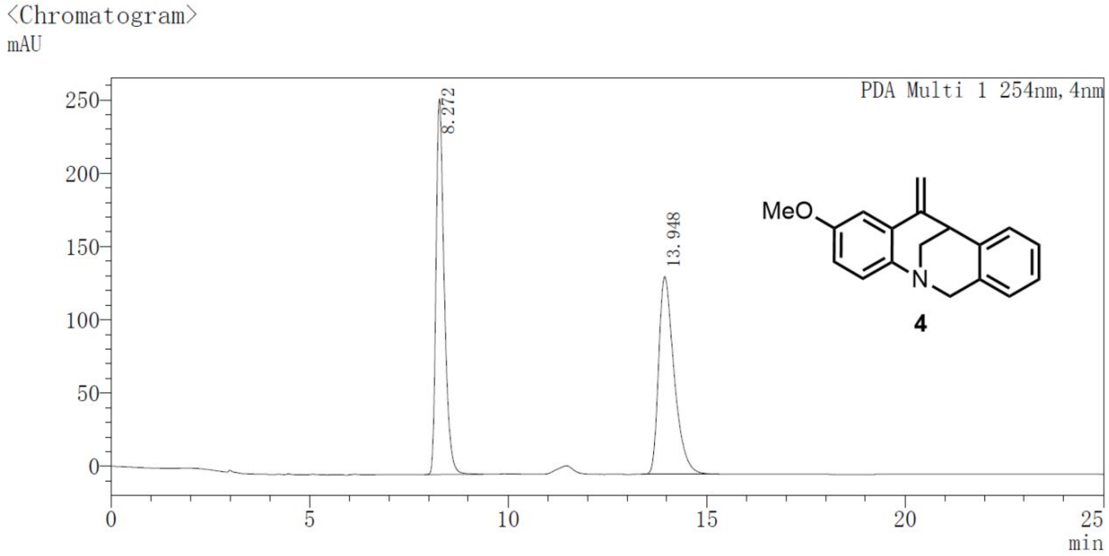

<Peak Table>

PDA Ch1 254nm

No.	Ret. Time (min)	Height (uAU)	Height%	Area (uAU*min)	Area%
1	8.272	256096	65.530	3682152	50.137
2	13.948	134709	34.470	3662020	49.863
Total		390805	100.000	7344172	100.000

Enantioselective:

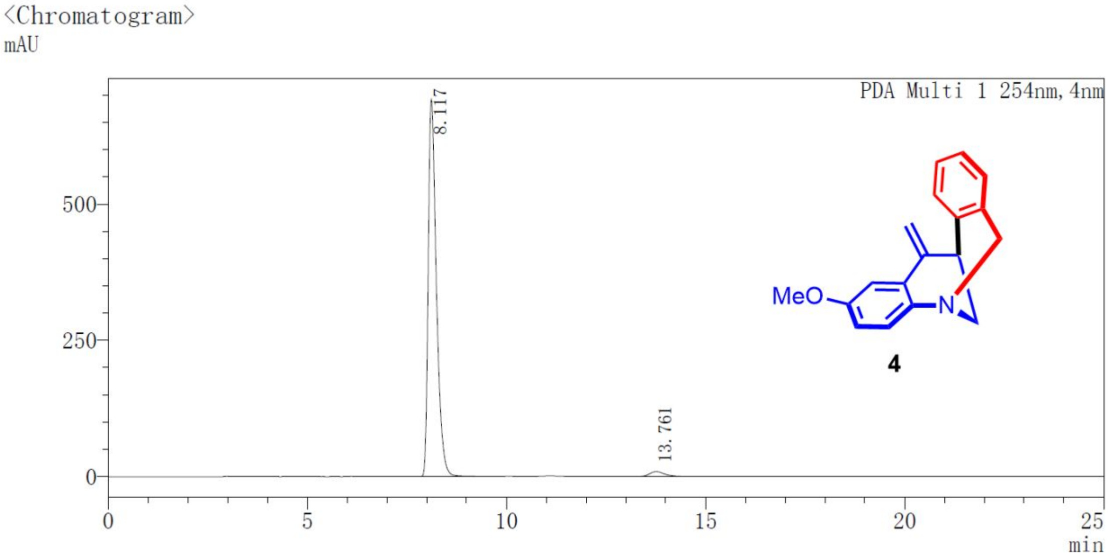

<Peak Table>

PDA Ch1 254nm

No.	Ret. Time (min)	Height (uAU)	Height%	Area (uAU*min)	Area%
1	8.117	691910	98.707	9852762	97.611
2	13.761	9065	1.293	241177	2.389
Total		700974	100.000	10093939	100.000

Racemic:

〈Chromatogram〉
mAU

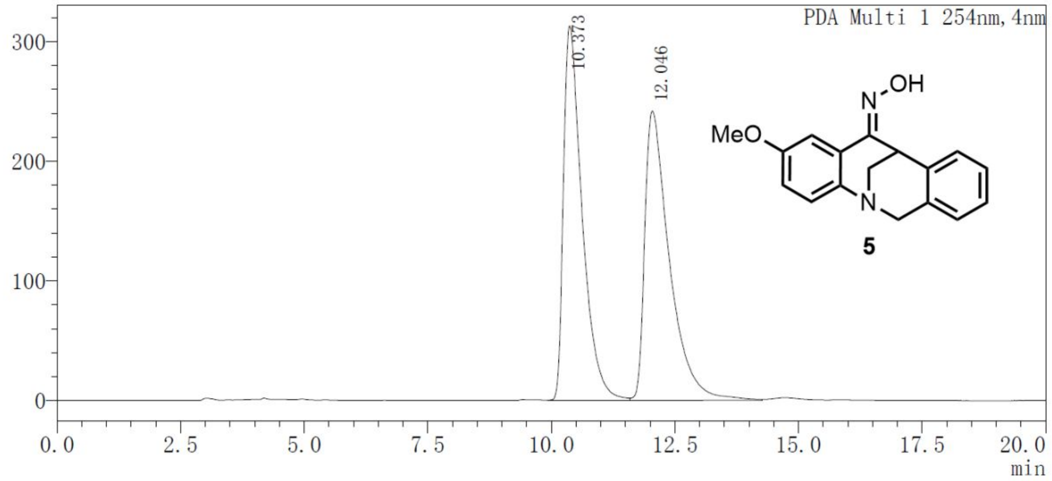

〈Peak Table〉

PDA Ch1 254nm

No.	Ret. Time(min)	Height(uAU)	Height%	Area(uAU*min)	Area%
1	10.373	313081	56.420	8242589	49.475
2	12.046	241835	43.580	8417601	50.525
Total		554916	100.000	16660189	100.000

Enantioselective:

〈Chromatogram〉
mAU

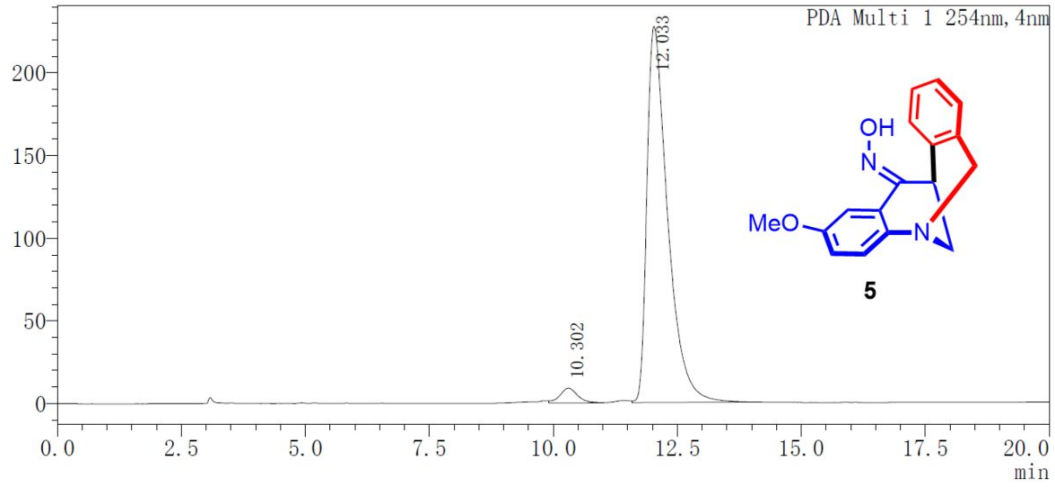

〈Peak Table〉

PDA Ch1 254nm

No.	Ret. Time(min)	Height(uAU)	Height%	Area(uAU*min)	Area%
1	10.302	8916	3.771	221200	3.226
2	12.033	227478	96.229	6636415	96.774
Total		236394	100.000	6857615	100.000

Racemic:

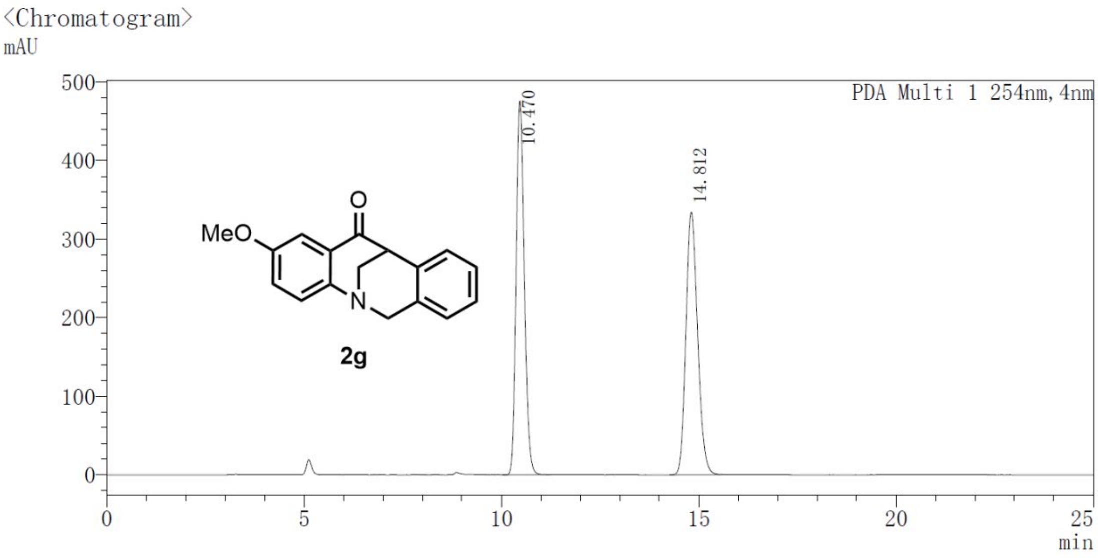

<Peak Table>

PDA Ch1 254nm

No.	Ret. Time (min)	Height (uAU)	Height%	Area (uAU*min)	Area%
1	10.470	475614	58.734	6876885	49.989
2	14.812	334162	41.266	6879977	50.011
Total		809776	100.000	13756862	100.000

Enantioselective:

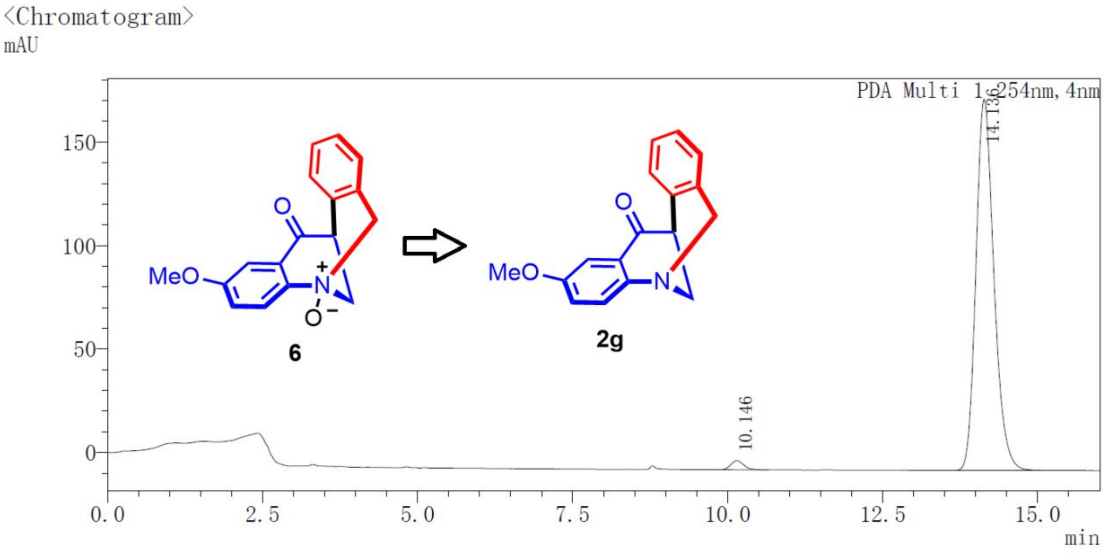

<Peak Table>

PDA Ch1 254nm

No.	Ret. Time (min)	Height (uAU)	Height%	Area (uAU*min)	Area%
1	10.146	4469	2.432	64841	1.762
2	14.136	179296	97.568	3615630	98.238
Total		183765	100.000	3680471	100.000

Racemic:

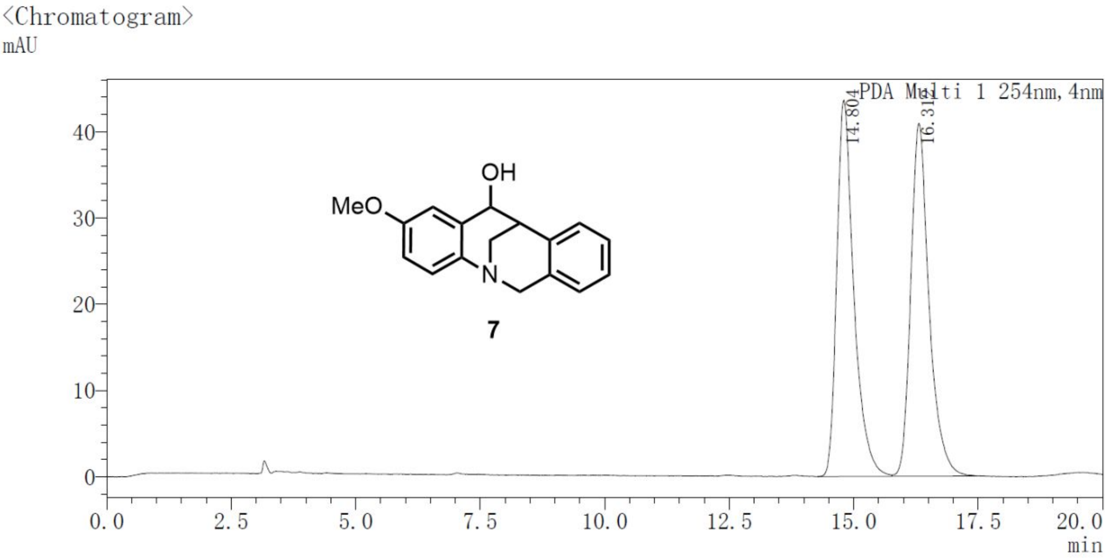

<Peak Table>

PDA Ch1 254nm

No.	Ret. Time (min)	Height (uAU)	Height%	Area (uAU*min)	Area%
1	14.804	43617	51.603	1040833	49.869
2	16.312	40907	48.397	1046294	50.131
Total		84524	100.000	2087127	100.000

Enantioselective:

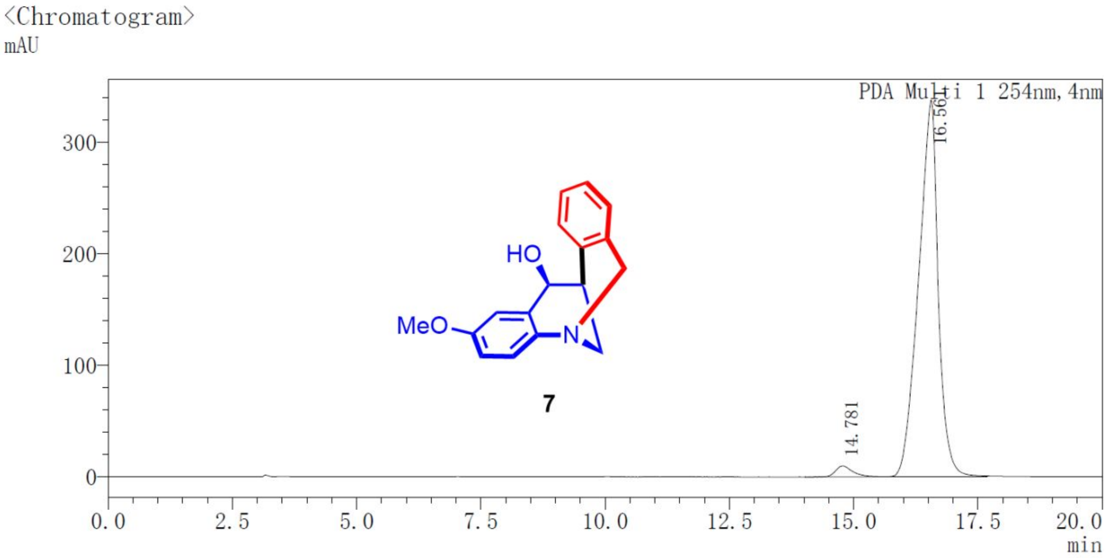

<Peak Table>

PDA Ch1 254nm

No.	Ret. Time (min)	Height (uAU)	Height%	Area (uAU*min)	Area%
1	14.781	9836	2.828	240739	2.515
2	16.561	337992	97.172	9330573	97.485
Total		347828	100.000	9571312	100.000

Racemic:

<Chromatogram>

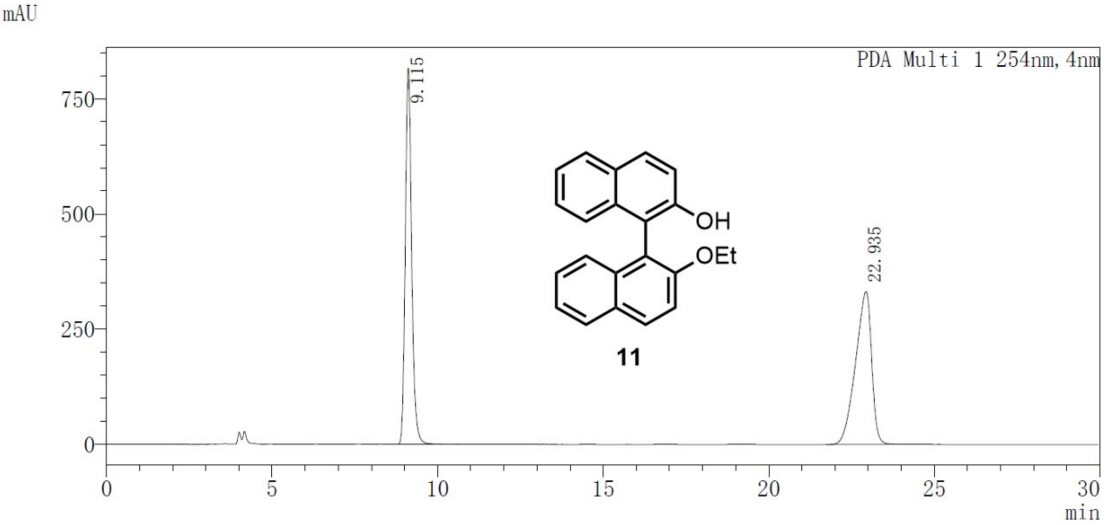

<Peak Table>

PDA Ch1 254nm

No.	Ret.Time(min)	Height(uAU)	Height%	Area(uAU*min)	Area%
1	9.115	816167	71.089	10935143	49.413
2	22.935	331925	28.911	11194953	50.587
Total		1148092	100.000	22130097	100.000

Enantioselective:

<Chromatogram>

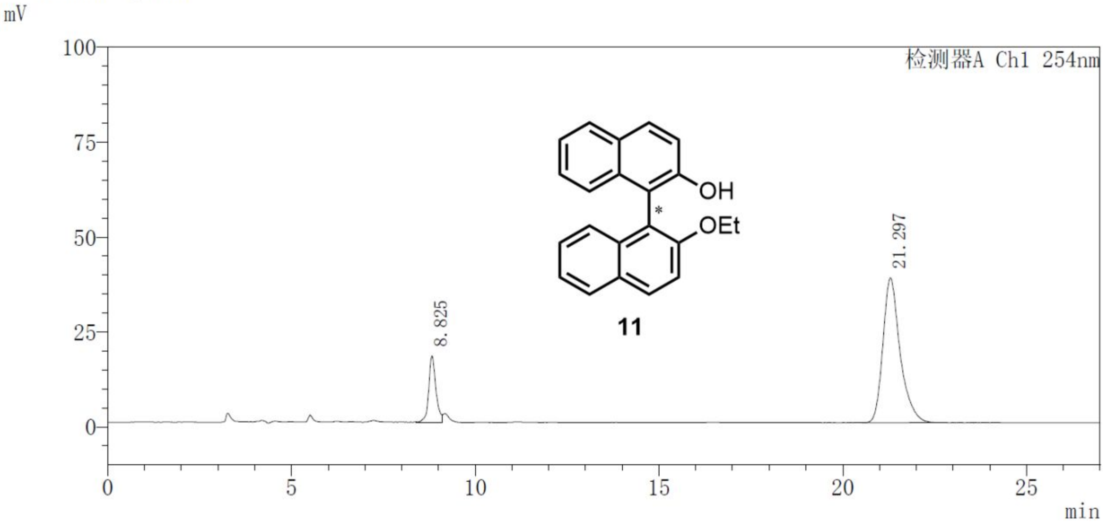

<Peak Table>

检测器A Ch1 254nm

No.	Ret.Time(min)	Area(uAU*min)	Height(uAU)	Height%	Area%
1	8.825	237825	17529	31.501	16.086
2	21.297	1240641	38117	68.499	83.914
Total		1478465	55646	100.000	100.000

Racemic:

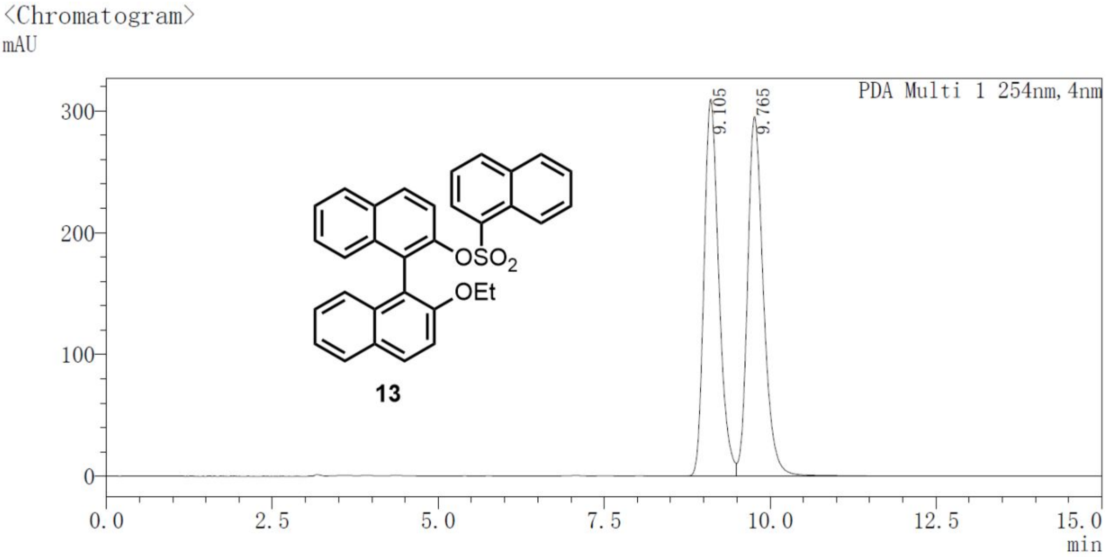

<Peak Table>

PDA Ch1 254nm

No.	Ret. Time (min)	Height (uAU)	Height%	Area (uAU*min)	Area%
1	9.105	309382	51.198	4799153	49.361
2	9.765	294907	48.802	4923467	50.639
Total		604289	100.000	9722620	100.000

Enantioselective:

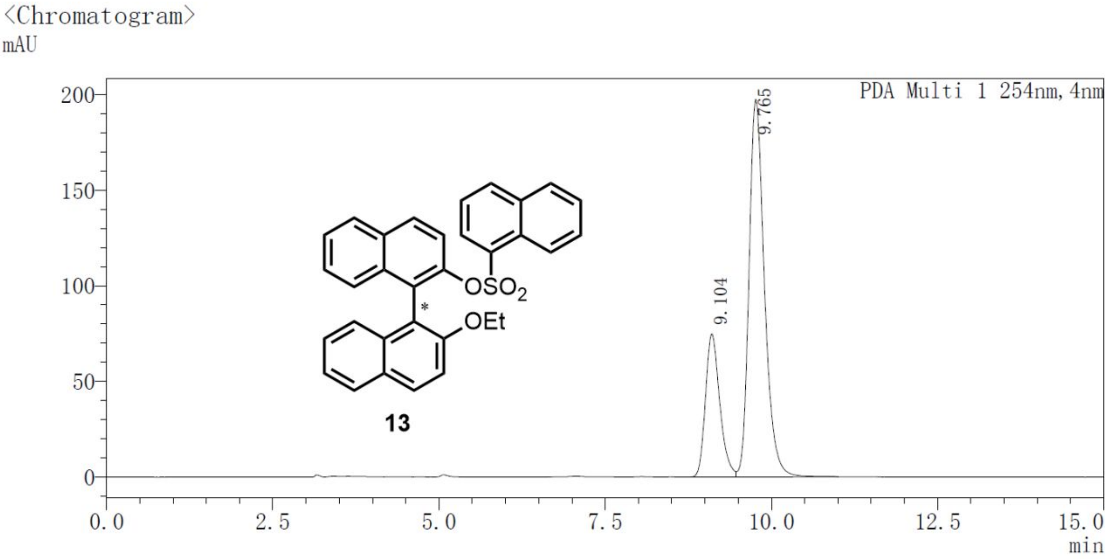

<Peak Table>

PDA Ch1 254nm

No.	Ret. Time (min)	Height (uAU)	Height%	Area (uAU*min)	Area%
1	9.104	74790	27.485	1121426	26.031
2	9.765	197320	72.515	3186562	73.969
Total		272110	100.000	4307988	100.000

Racemic:

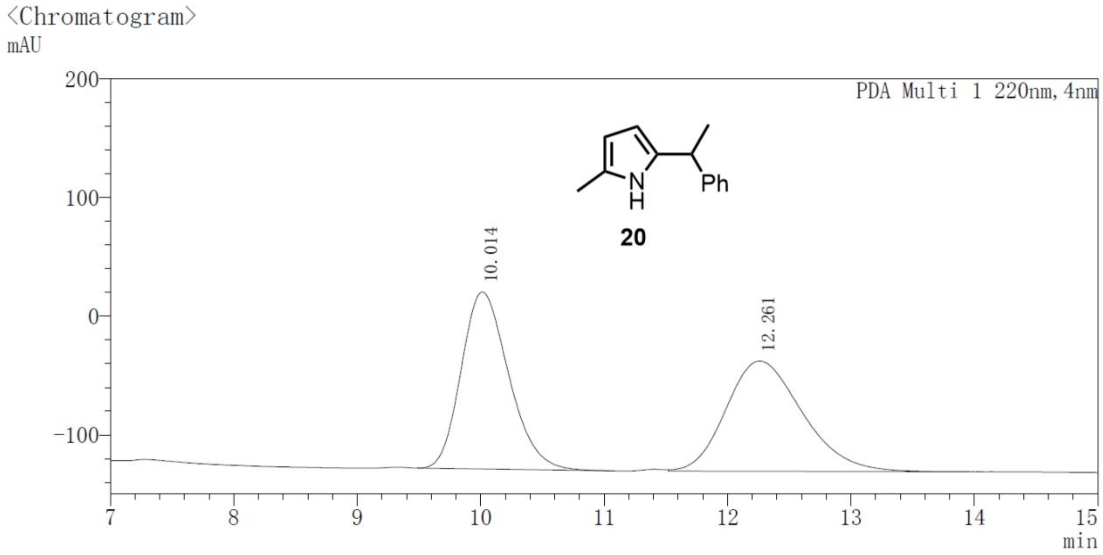

〈Peak Table〉

PDA Ch1 220nm

No.	Ret. Time (min)	Height (uAU)	Height%	Area (uAU*min)	Area%
1	10.014	149213	61.604	3996694	49.974
2	12.261	92999	38.396	4000792	50.026
Total		242212	100.000	7997487	100.000

Enantioselective:

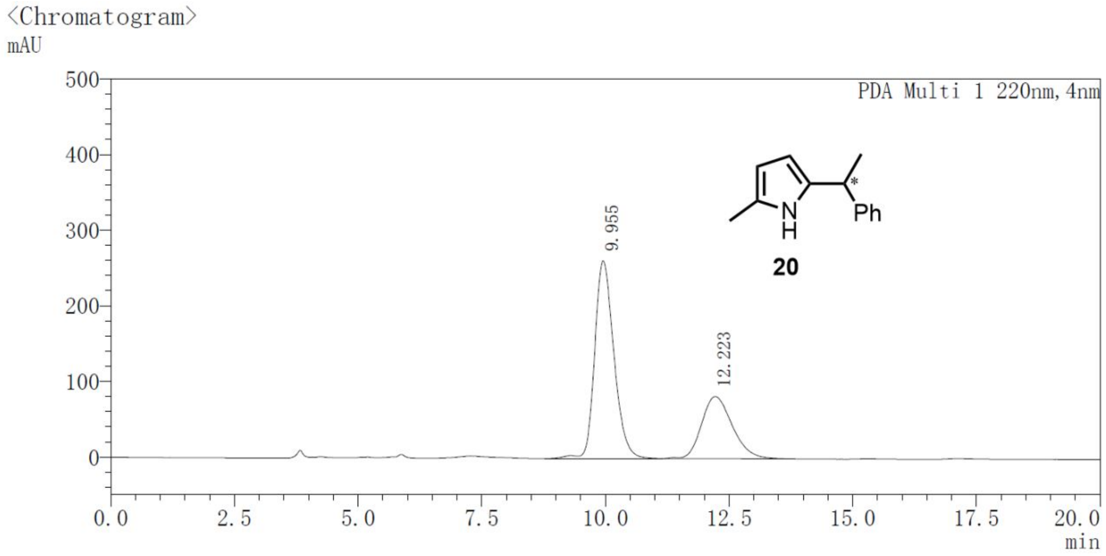

〈Peak Table〉

PDA Ch1 220nm

No.	Ret. Time (min)	Height (uAU)	Height%	Area (uAU*min)	Area%
1	9.955	261697	76.098	7100527	66.596
2	12.223	82199	23.902	3561642	33.404
Total		343896	100.000	10662169	100.000

Racemic:

<Chromatogram>
mV

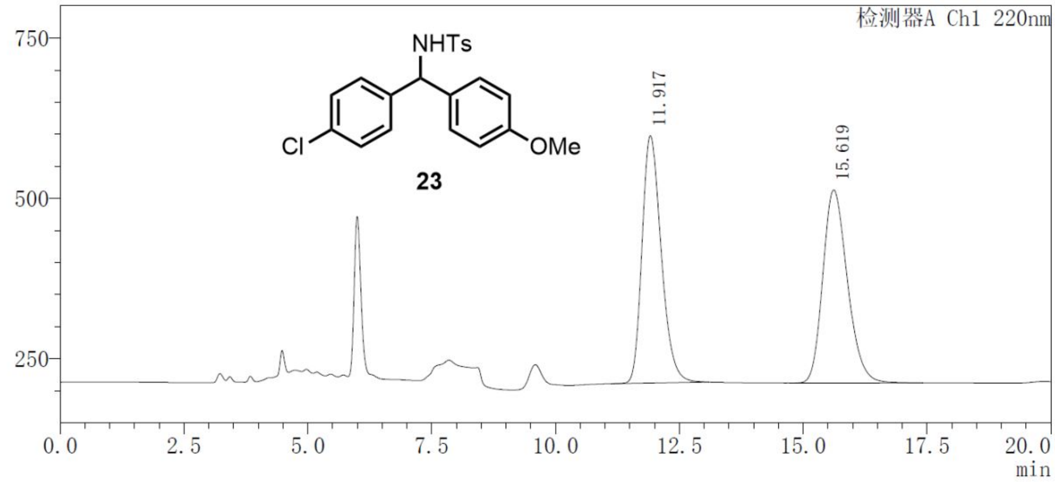

<Peak Table>

检测器A Ch1 220nm

No.	Ret.Time(min)	Area(uAU*min)	Height(uAU)	Height%	Area%
1	11.917	10384850	385862	56.163	49.934
2	15.619	10412432	301176	43.837	50.066
Total		20797282	687039	100.000	100.000

Enantioselective:

<Chromatogram>
mV

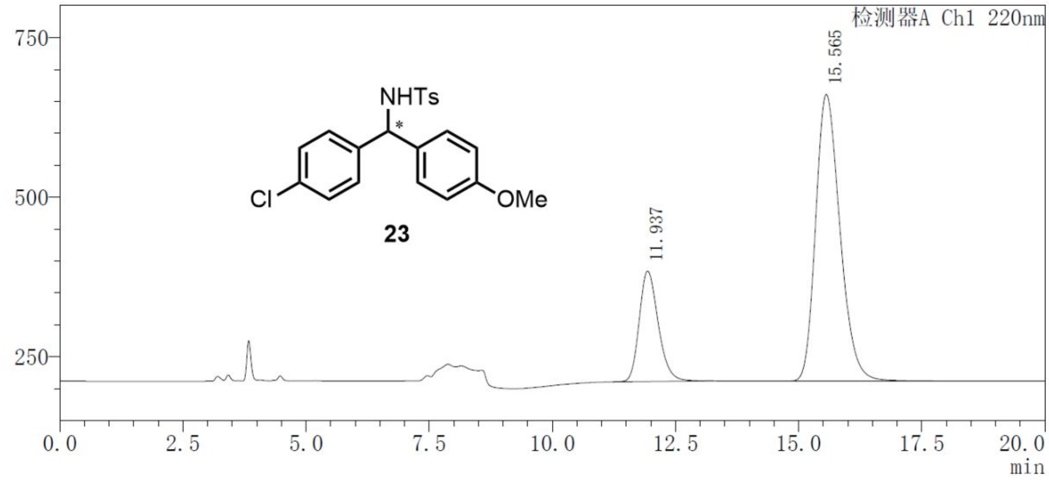

<Peak Table>

检测器A Ch1 220nm

No.	Ret.Time(min)	Area(uAU*min)	Height(uAU)	Height%	Area%
1	11.937	4637652	173110	27.823	23.080
2	15.565	15455968	449066	72.177	76.920
Total		20093620	622176	100.000	100.000

15. References

1. Zhao, G.; Wu, Y.; Wu, H.-H.; Yang, J.; Zhang, J. Pd/GF-Phos-catalyzed asymmetric three-component coupling reaction to access chiral diarylmethyl alkynes. *J. Am. Chem. Soc.* **2021**, *143*, 17983-17988.

2. a) Luan, L.-B.; Song, Z.-J.; Li, Z.-M.; Wang, Q.-R. Synthesis of new tricyclic 5,6-dihydro-4*H*-benzo[*b*][1,2,4]triazolo[1,5-*d*][1,4]diazepine derivatives by [3^+ + 2]-cycloaddition/rearrangement reactions. *Beilstein J. Org. Chem.* **2018,** *14,* 1826-1833; b) Schmidt, R. G.; Bayburt, E. K.; Latshaw, S. P.; Koenig, J. R.; Daanen, J. F.; McDonald, H. A.; Bianchi, B. R.; Zhong, C.; Joshi, S.; Honore, P. et al. Chroman and tetrahydroquinoline ureas as potent TRPV1 antagonists. *Bioorg. Med. Chem. Lett.* **2011**, *21*, 1338-1341; c) Anderson, K. W.; Tepe, J. J. Trifluoromethanesulfonic acid catalyzed Friedel–Crafts acylation of aromatics with β-lactams. *Tetrahedron* **2002**, *58*, 8475-8481.

3. a) Friberg, A.; Olsson, C.; Ek, F.; Berga, U.; Frejd, T. Cleft molecules as organocatalysts in an asymmetric hetero-Diels–Alder reaction. *Tetrahedron: Asymmetry* **2007**, 18, 885-891; b) Koichiro, N.; Fukunaga, R, Komatsu M.; Yamanaka, M.; Chikamatsu, H. Synthesis and enantiomer recognition of dipodands and crown ethers containing the 2,3:6,7-Dibenzobicyclo[3.3.1]nona-2,6-diene Residue as the Chiral Subunit. *Bull. Chem. Soc. Jpn.* **1989**, *62*, 83-88; c) He, C.; Wang, B.; Gao, M.; Gu, Z. Synthesis of chiral cleft *C, N*-palladium and iridium complexes from 2,3:6,7-dibenzobicyclo[3.3.1]nona-2,6-diene-4,8-dione and their synthetic applications. *Organometallics* **2017**, *36*, 1073-1078; d) Huang, X.; Huang, J.; Du, C.; Zhang, X.; Song, F.; You, J. *N*-oxide as a traceless oxidizing directing group: mild rhodium(III)-catalyzed C-H olefination for the synthesis of *ortho*-alkenylated tertiary anilines. *Angew. Chem. Int. Ed.* **2013**, *52*, 12970-12974; e) Slater, N. H.; Buckley, B. R.; Elsegood, M. R. J.; Teat, S. J.; Kimber, M. C. Controlling the assembly of C2-symmetric molecular tectons using a thiocarbamate appended carbocyclic cleft molecule analogous to Tröger's base. *Cryst. Growth Des.* **2016**, *16*, 3846-3852.

4. a) Jones, B. A.; Balan, T.; Jolliffe, J. D.; Campbell, C. D.; Smith, M. D. Practical and scalable kinetic resolution of BINOLs mediated by a chiral counterion. *Angew. Chem. Int. Ed.* **2019**, *58*, 4596-4600. b) Liu, Y.; Tao, R.; Lin, Z.-K.; Yang, G.; Zhao, Y. Redox-enabled direct stereoconvergent heteroarylation of simple alcohols. *Nat. Commun.* **2021**, *12*, 5035-5042; c) Schrapel, C.; Frey, W.; Garnier, D.; Peters, R. Highly enantioselective ferrocenyl palladacycle-acetate catalysed arylation of aldimines and ketimines with arylboroxines. *Chem. Eur. J.* **2017**, *23*, 2448-2460.

5. Frisch, M. J.; Trucks, G. W.; Schlegel, H. B.; Scuseria, G. E.; Robb, M. A.; Cheeseman, J.; R. Cooke, S.; Scalmani, G.; Barone, V.; Mennucci, B. et al. Revision D.01, Gaussian, Inc., Wallingford CT, **2010**.

6. a) Zhao, Y.; Truhlar, D. G. Density functional for spectroscopy: no long-range self-interaction error, good performance for rydberg and charge-transfer states, and better performance on average than B3LYP for ground states. *J. Phys. Chem. A.* **2006**, *110*, 13126-13130. b) Zhao, Y.; Truhlar, D. G. Density functionals with broad applicability in chemistry. *Acc. Chem. Res.* **2008**, *41*, 157-167. c) Zhao, Y.; Truhlar, D. G. Applications and validations of the Minnesota density functionals. *Chem. Phys. Lett.* **2011**, *502*, 1-13.

7. Hay, P. J.; Wadt, W. R. Ab initio effective core potentials for molecular calculations. Potentials for the transition metal atoms Sc to Hg. *J. Chem. Phys.* **1985**, *82*, 270-283.

8. Hehre, W.; Radom, L., P. v. R. Schleyer and JA Pople, Ab initio Molecular Orbital Theory. Wiley, New York: 1986.
9. Zhao, Y.; Truhlar, D. G. The M06 suite of density functionals for main group thermochemistry, thermochemical kinetics, noncovalent interactions, excited states, and transition elements: two new functionals and systematic testing of four M06 functionals and 12 other functionals. *Theor. Chem. Acc.* **2008**, *119*, 525-525.
10. Tomasi, J.; Mennucci, B.; Cammi, R. Quantum mechanical continuum solvation models. *Chem. Rev.* **2005**, *105*, 2999-3094.
11. a) Johnson, E. R.; Keinan, S.; Mori-Sánchez, P.; Contreras-García, J.; Cohen, A. J.; Yang, W. Revealing noncovalent interactions. *J. Am. Chem. Soc.* **2010**, *132*, 6498-6506. b) Lu, T.; Chen, F. Multiwfn: A multifunctional wavefunction analyzer. *J. Comput. Chem.* **2012**, *33*, 580-592. c) Humphrey, W.; Dalke, A.; Schulten, K. VMD: Visual molecular dynamics. *J. Mol. Graphics.* **1996**, *14*, 33-38.
12. Legault, C. Y. C., 1.0b; Universitéde Sherbrooke: Québec, Montreal, Canada; **2009**, http://www.cylview.org.
